# Topiroxostat versus allopurinol in patients with chronic heart failure complicated by hyperuricemia: A prospective, randomized, open-label, blinded-end-point clinical trial

**DOI:** 10.1371/journal.pone.0261445

**Published:** 2022-01-25

**Authors:** Masashi Sakuma, Shigeru Toyoda, Takuo Arikawa, Yota Koyabu, Toru Kato, Taichi Adachi, Hideaki Suwa, Jun-ichi Narita, Koetsu Anraku, Kimihiko Ishimura, Fumitake Yamauchi, Yasunori Sato, Teruo Inoue

**Affiliations:** 1 Department of Cardiovascular Medicine, Dokkyo Medical University, Mibu, Japan; 2 Department of Clinical Research, National Hospital Organization Tochigi Medical Center, Utsunomiya, Japan; 3 Department of Cardiology, National Hospital Organization Tochigi Medical Center, Utsunomiya, Japan; 4 Department of Neurosurgery, Tochigi Medical Center Tochinoki, Tochigi, Japan; 5 Department of Internal Medicine, Tochigi Medical Center Tochinoki, Tochigi, Japan; 6 Department of Cardiology, Yuai Memorial Hospital, Koga, Japan; 7 Department of Preventive Medicine and Public Health, Keio University School of Medicine, Shinanomachi, Japan; VU University Medical Center, NETHERLANDS

## Abstract

**Background:**

The benefits of xanthine oxidase inhibitors to chronic heart failure (CHF) patients is controversial. We investigated the beneficial effects of a novel xanthine oxidoreductase inhibitor, topiroxostat, in patients with CHF and hyperuricemia (HU), in comparison to allopurinol.

**Methods and results:**

The prospective, randomized open-label, blinded-end-point study was performed in 141 patients with CHF and HU at 4 centers. Patients were randomly assigned to either topiroxostat or allopurinol group to achieve target uric acid level ≤6.0 mg/dL. According to the protocol, 140 patients were followed up for 24 weeks. Percent change in ln (N-terminal-proB-type natriuretic peptide) at week 24 (primary endpoint) was comparable between topiroxostat and allopurinol groups (1.6±8.2 versus -0.4±8.0%; P = 0.17). In the limited number of patients with heart failure with reduced ejection fraction (HFrEF) (left ventricle ejection fraction <45%), ratio of peak early diastolic flow velocity at mitral valve leaflet to early diastolic mitral annular motion velocity (E/e’) decreased in topiroxostat group, but not in allopurinol group. Urinary 8-hydroxy-2’-deoxyguanosine and L-type fatty acid-binding protein levels increased and osmolality decreased significantly in allopurinol group, while these changes were less or absent in topiroxostat group. In allopurinol group HFrEF patients, additional to the increases in these urinary marker levels, urinary creatinine levels decreased, with no change in clearance, but not in topiroxostat group.

**Conclusions:**

Compared with allopurinol, topiroxostat did not show great benefits in patients with CHF and HU. However, topiroxostat might have potential advantages of reducing left ventricular end-diastolic pressure, not worsening oxidative stress in proximal renal tubule, and renoprotection over allopurinol in HFrEF patients.

## Introduction

Hyperuricemia is known to be a risk factor not only for gout and nephropathy, but also for cardiovascular events [1, 2]. In addition, hyperuricemia has been reported to be associated with the development and worsening of chronic heart failure (CHF) [3, 4]. Considering this mechanism, xanthine oxidoreductase (XOR) is known to be one of the sources of reactive oxygen species (ROS) production, and is thought to potentially be involved in myocardial remodeling and the formation or progression of heart failure as a result of increased ROS in heart failure [5]. Furthermore, it has been reported that XOR activity in the vascular endothelium is significantly increased in patients with heart failure. In addition, protein expressions of xanthine dehydrogenase (XDH) and xanthine oxidase (XO) are increased in the myocardium of patients with dilated cardiomyopathy [6, 7]. These reports suggest that hyperuricemia and XOR in heart failure may not only represent a prognostic factor, but also serve as a therapeutic target. However, whether or not intervention with respect to XOR can suppress the formation and progression of heart failure has not been adequately investigated.

There are currently three XOR inhibitors (XORi) that are clinically used in Japan: allopurinol, febuxostat and topiroxostat. While allopurinol is limited to the treatment of hyperuricemia complicated by hypertension, febuxostat and topiroxostat are used for treatment of hyperuricemia in patients with or without hypertension. It has been shown that febuxostat exhibits superior anti-inflammatory and anti-oxidative effects; thus, febuxostat is expected to attenuate atherosclerosis [8–10]. However, in 2018, however, a large clinical trial [Cardiovascular Safety of Febuxostat and Allopurinol in Participants With Gout and Cardiovascular Comorbidities (CARES)] demonstrated that febuxostat increased cardiovascular mortality and all-cause mortality, compared to allopurinol [11]. On the other hand, the FAST trial, which was conducted mainly in Europe, reported that febuxostat is non-inferior to allopurinol therapy with respect to the cardiovascular endpoint, and that its long-term use is not associated with an increased risk of death or serious adverse events, compared with allopurinol [12]. Therefore, the relative effects of these drugs remain controversial. Recently, topiroxostat has also shown renoprotective effects, as demonstrated by a reduction in urinary albumin excretion. In Addition, topiroxostat improves vascular endothelial function in patients with hyperuricemia [13]. However, the effects of topiroxostat in patients with cardiovascular disease have not been established. Furthermore, there have been no reports indicating the adverse effects of topiroxostat on the cardiovascular system.

We hypothesized that topiroxostat has superior effects, compared to allopurinol, in patients with chronic heart failure and hyperuricemia. The “Effect of Xanthine Oxidase Inhibitor in Chronic Heart Failure Patients Complicated with Hyperuricemia study (Excited-UA study)” was designed to test our hypothesis.

## Methods

### Study design and oversight

Excited-UA is a prospective, randomized (1:1), open-label, blinded-end-point clinical trial designed to investigate the effectiveness of topiroxistat in patients with chronic heart failure and hyperuricemia, in comparison with allopurinol. This study was designed by the Excited-UA investigators and performed at 4 centers (Dokkyo Medical University Hospital, National Hospital Organization Tochigi Medical Center, Tochigi Medical Center Tochinoki, and Yuai Memorial Hospital). The study design, detailed protocol, and rationale have been previously published [14].

The protocol was approved at Institutional Review Board for Clinical Research ofeach participating center by the respective institutional review boards or ethics committees Dokkyo Medical University Hospital (approval number 27087, Oct 13, 2015), Ethics Review Committee of National Hospital Organization Tochigi Medical Center (approval number 28–2, Jun 14, 2016), Ethics Committee of Tochigi Medical Center Tochinoki (approval number 2, Aug 22, 2016), and Ethics Committee of Yuai Memorial Hospital (May 26, 2016). An independent data and safety monitoring committee oversaw the trial. A contract research organization (Soiken, Inc., Osaka, Japan) was responsible for data collection, database establishment, and statistical analyses. The first author and an academic statistician at Dokkyo Medical University had full access to the trial databases, generated trial analyses, prepared the first draft manuscript, and made the decision to submit the manuscript for publication. The authors assume responsibility for the accuracy and completeness of the data, analyses, and fidelity of the trial to the protocol. The trial was conducted in full compliance with the Declaration of Helsinki and Ethical Guidelines for Medical and Health Research Involving Human Subjects established by the Ministry of Health, Labor, and Welfare. The information of this study was registered with the University Hospital Medical Information Network clinical trial registry (UMIN-CTR: UMIN000020939 URL: https://upload.umin.ac.jp/cgi-open-bin/ctr_e/ctr_view.cgi?recptno=R000024159).

### Study population

Patients who had chronic heart failure and hyperuricemia were enrolled in this study. Eligible patients were aged ≥20 and <85 years and have chronic heart failure, defined as plasma brain natriuretic peptide (BNP) level ≥40 pg/mL, based on previous reports [15, 16], hyperuricemia (serum uric acid level ≥7.0 mg/dL), or on treatment with anti-hyperuricemic agents. Prior to assessing eligibility, the participants were required to receive an adequate explanation of the study plan and written informed consent was obtained from each patient. Detailed inclusion and exclusion criteria were previously listed [14].

### Randomization

Eligible patients were randomly assigned to either the topiroxostat (40–160 mg/day) or allopurinol (100–200 mg/day) groups to achieve the target uric acid level of 6.0 mg/dL and below. According to the protocol, 140 patients were followed up for 24 weeks. In this study, patient registration and randomization were performed dynamic allocation by the contract research organization using the electric data capturing (EDC) system. Randomization was performed using a minimization method with a biased-coin assignment balancing on plasma BNP level at registration (<200 and ≥200 pg/mL) and echocardiography-based left ventricular ejection fraction (LVEF: <45% and ≥45%). The daily topiroxostat and allopurinol dosages were 40–160 and 100–300 mg, respectively, in Japan. Thus, 70 participants assigned to the topiroxostat group received topiroxostat at an initial dose of 40 mg/day; the dose was increased to 160 mg/day every 4 weeks to achieve the appropriate uric acid level of ≤6.0 mg/dL. Seventy patients assigned to the allopurinol group receive an initial allopurinol dose of 100 mg/day. In the allopurinol group, patients with renal dysfunction (estimated glomerular filtration rate: eGFR<50 mL/min/1.73m^2^ or creatinine clearance: CCr<50 mL/min) received a fixed dose of 100 mg/day during the observation period. Conversely, in patients without renal dysfunction, allopurinol dose was increased to 200 mg/day every 4 weeks to achieve the target uric acid level of ≤6.0 mg/dL. If the uric acid level decreased to ≤2.0 mg/dL during the study interval, the dose of each agent was reduced (10–80 mg/day reduction in topiroxostat and 100 mg/day in allopurinol).

### Observations/Measurements

Detailed observations or measurements are shown in S1 Table. Additional to general blood and urine tests performed in each center, specific biomarkers such as blood N-terminal pro-brain natriuretic peptide (NT-proBNP), malondialdehyde-modified low density lipoprotein (MDA-LDL), troponin I, xanthine oxidoreductase (XOR) activity levels, and urinary 8-hydroxy-2’-deoxyguanosine (8-OHdG), liver-type fatty acid-binding protein (L-FABP), and excreted albumin levels were collectively measured in core laboratories. The results of these biomarkers were not disclosed until the end of the follow-up period.

Echocardiography was performed in each center and the LVEF, peak early diastolic flow velocity at mitral valve leaflet (E), early diastolic mitral annular motion velocity (e’), E to e’ ratio (E/e’), and transtricuspid pressure gradient (TRPG) measured. Vascular endothelial function tests such as brachial artery flow-mediated dilation (FMD) and/or reactive hyperemia peripheral arterial tonometry (RH-PAT) were also performed in each center.

### Study endpoint

The primary endpoint is the percent change in serum NT-proBNP level 24 weeks from baseline. The secondary endpoints are as follows: 1) percent change in serum NT-proBNP level 12 weeks from baseline; 2) percent change in plasma BNP level 12 and 24 weeks from baseline; 3) correlation between the percent change in the NT-proBNP and BNP levels at week 24; 4) change in FMD and RH-PAT values 24 weeks from baseline; 5) correlation between the change in FMD and RH-PAT values at week 24; 6) change in serum uric acid level 24 weeks from baseline; 7) change in specific biomarker values 24 weeks from baseline; and 8) change in echocardiographic parameter values 24 weeks from baseline. With regard to the primary endpoint, serum NT-proBNP level was natural logarithm transformed to approximate data to a normal distribution, and the difference between baseline and week 24 weeks from baseline in naturalized logarithm is shown as a percent change. BNP, troponin I, L-FABP, and albumin excretion were also calculated after natural logarithmic transformation of the same background. Additionally, FMD is reported to be impaired by chronic atrial fibrillation [17], so FMD was analyzed only in patients without chronic atrial fibrillation.

As for safety endpoints, we assessed the frequency and proportion of serious adverse events and adverse events whose causal relationship with the study drug cannot be ruled out such as gout arthritis and hepatic dysfunction.

### Statistical analyses

As described in the protocol paper [14], based on the data of NT-proBNP values converted from BNP values in previous reports, we assumed for percent change of NT-proBNP level that the average is -39.0±75.0% in the topiroxostat group and assumed no change in the allopurinol group. With regard to the number of the patients, on the assumption of analytical power 80% with a two-sided significance level of 5%, the number of participants needed to detect statistical significance of topiroxostat is 60 patients per group. Additionally, in consideration of dropout, the target sample size was finally estimated to be a total of 140 patients, with 70 patients per group.

Primary and secondary endpoint analyses performed in the full analysis set (FAS) included all participants, except patients who rescinded consent to the trial. The analyses in the per-protocol set (PPS) were also performed to verify significant changes obtained in FAS analyses. Additionally, subgroup analyses in heart failure with reduced ejection fraction (HFrEF: LVEF<45%) and heart failure with preserved ejection fraction (HFpEF: LVEF≥50%) patients were performed on evaluation items with significant differences in FAS or PPS. NT-proBNP and BNP were analyzed by logarithmic transformation according to the statistical analysis plan. For the others, the distribution of each parameter was checked, and the results were logarithmically transformed in case of these deviated from the normal distribution.

Patient characteristics were compared using the chi-square test for categorical variables, and unpaired Student’s *t*-test for continuous variables, as appropriate, between the two study groups. For the primary endpoint, analysis of covariance (ANCOVA) analysis was used to compare the changes in NT-proBNP. ANCOVA included the randomized study group, time point (0, 12, and 24 weeks), interaction between the study group and time points as fixed effects, and covariance adjusted for BNP and EF at enrollment as allocation factors. Furthermore, mixed-effects model repeated measures (MMRM) analysis was used to compare the changes in NT-proBNP from baseline to week 12 and week 24. MMRM included the randomized study group, time point (0, 12, and 24 weeks), interaction between the study group and time points as fixed effects, and BNP and EF at enrollment as covariates. Regarding the primary and secondary endpoint analysis, changes in the values 24 weeks from baseline were assessed using the paired Student’s *t*-test for continuous variables and Wilcoxon signed-rank test for categorical outcomes in each group. For the continuous variables, percent change in values was calculated and the difference between both groups analyzed, using the unpaired *t*-test or Wilcoxon rank-sum test. For correlation analyses, Pearson’s product-moment correlation coefficient or Spearman’s’ rank-correlation coefficient was calculated to assess significance. All comparisons were planned, and all p-values two-sided. P-values <0.05 were considered statistically significant. All statistical analyses were performed using SAS V.9.4 and are described in the statistical analysis plan, which was fixed prior to database lock.

## Results

### Study patients

**Fig 1:**
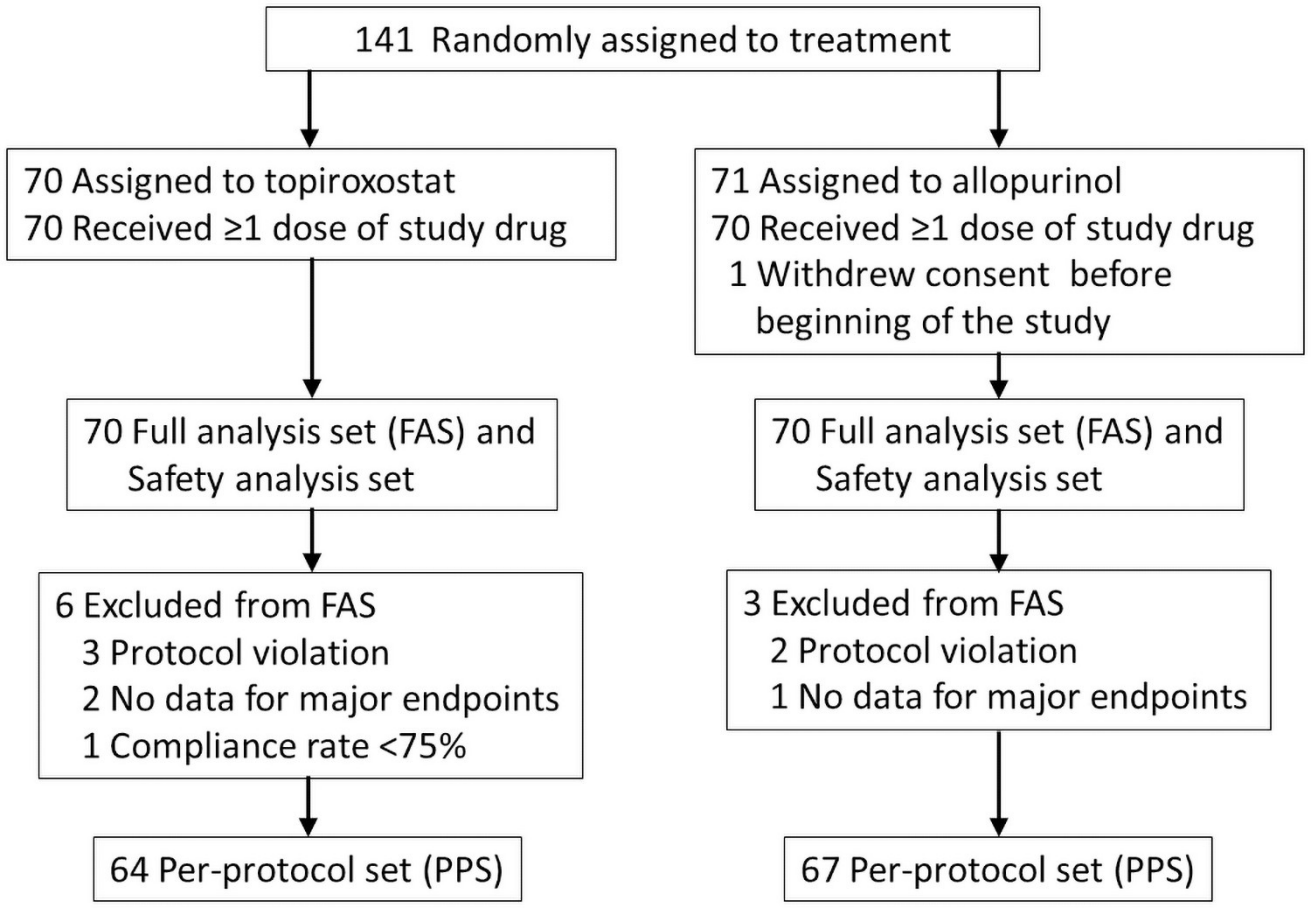
Flowchart of patients enrolled in the excited-UA study. Abbreviations: FAS, full analysis set; PPS, per-protocol set.

For this study, a total of 141 patients were scheduled to be enrolled from December 2015 to April 2018 from 4 centers. After randomization, 70 patients were assigned into the topiroxostat group and the remaining 71 into the allopurinol group. One patient in the allopurinol group was excluded because of withdrawal of consent before the study began. As a result, a total of 140 patients (topiroxostat group; n = 70, allopurinol group; n = 70) were included as a FAS and safety analyses set. In these patients, the New York Heart Association (NYHA) class was only class I or II, so this study included mild heart failure population. During the study period, 6 patients (protocol violation; n = 3, no data for major endpoints and n = 2, compliance rate less than 75%; n = 1) in the topiroxostat and 3 (protocol violation; n = 2, no data for major endpoints; n = 1) in the allopurinol groups were excluded. Finally, 131 patients (topiroxostat group; n = 64 and allopurinol group; n = 67) were analyzed as a PPS (Fig 1).

### Baseline characteristics

The baseline characteristics of the FAS are shown in Table 1. Hypertension prevalence was lower and the echocardiographic parameter and transtricuspid pressure gradient (TRPG) were higher in the topiroxostat group, compared with the allopurinol group. The other parameters for baseline characteristics, including NYHA class, were comparable between both the topiroxostat and allopurinol groups.

**Table 1:** Baseline characteristics (FAS analysis).

	Topiroxostat (n = 70)	Allopurinol (n = 70)	P Value
Age, yr	71.7±9.7	69.9±9.1	0.26
Male gender, n (%)	57 (81.4)	56 (80.0)	0.83
BMI, kg/m^2^	25.1±4.2	25.0±3.6	0.84
Hypertension, n (%)	42 (60.0)	55 (78.6)	0.017
Diabetes mellitus, n (%)	26 (37.1)	21 (30.0)	0.37
Dyslipidemia, n (%)	41 (58.6)	41 (58.6)	1.00
Chronic kidney disease, n (%)	12 (17.1)	13 (18.6)	0.83
Atrial fibrillation, n (%)	30 (42.9)	28 (40.0)	0.73
Arrhythmia episode, n (%)	13 (18.6)	13 (18.6)	1.00
NYHA I/II	45/25	49/21	0.47
Etiology of heart failure, n (%)			
Ischemic heart disease	29 (41.4)	32 (45.7)	0.61
Cardiomyopathy	18 (25.7)	13 (18.6)	0.31
Valvular disease	12 (17.1)	12 (17.1)	1.00
Hypertensive heart failure	8 (11.4)	9 (12.9)	0.80
Others	15 (21.4)	18 (25.7)	0.55
Biochemical data			
NT-proBNP, pg/mL	640.5 (325.0, 1370.0)	568.5 (313.0, 1160.0)	0.33
BNP, pg/mL	127.5 (84.9, 251.9) (n = 69)	126.5 (80.9, 191.0) (n = 69)	0.38
Troponin I, pg/mL	7.4 (3.9, 17.1)	6.8 (4.4, 14.6)	0.63
eGFR, mL/min/1.73/m^2^	54.4±15.6 (n = 69)	56.5±16.8 (n = 68)	0.46
Urinary albumin, mg/g·Cr	14.3 (6.0, 79.6) (n = 69)	19.7 (6.9, 57.2) (n = 70)	0.56
Urinary 8-OHdG, ng/mg·Cr	7.7±3.5	7.2±4.0	0.47
Urinary L-FABP,μg/g·Cr	3.1 (1.9, 6.2)	3.3 (1.9, 5.7)	0.94
MDA-LDL, U/L	95.6±31.8	96.2±29.5	0.90
Uric acid, mg/dL	8.3±1.4 (n = 69)	8.2±1.4 (n = 69)	0.63
XOR activity, pmol/h/mL	33.8 (25.5, 61.5)	29.2 (18.1, 57.5)	0.48
Echocardiographic data			
LVEF, %	52.4±12.9 (n = 70)	50.6±13.3 (n = 68)	0.42
E, cm/sec	81.8±41.1 (n = 69)	75.7±37.1 (n = 70)	0.36
E/e’	13.9±6.7 (n = 58)	13.1±7.1 (n = 67)	0.50
TRPG, mmHg	23.7±9.7 (n = 60)	20.4±7.4 (n = 55)	0.047
Vascular endothelial function data			
FMD, %	4.88±2.38 (n = 32)	4.73±2.29 (n = 33)	0.80
RHI	1.76±0.56 (n = 68)	1.80±0.65 (n = 68)	0.69
Gout episode, n (%)	1 (1.4)	2 (2.9)	0.56
Medication, n (%)			
ARBs	32 (45.7)	38 (54.3)	0.31
ACEIs	14 (20.0)	20 (28.6)	0.24
Ca antagonists	31 (44.3)	31 (44.3)	1.00
β-blockers	33 (47.1)	35 (50.0)	0.74
Diuretics	48 (68.6)	48 (68.6)	1.00
V2 receptor antagonist	2 (2.9%)	3 (4.3%)	0.65
Statins	39 (55.7)	40 (57.1)	0.86

Values are mean ± standard deviation, median (interquartile ranges), or n (percentage of patients). P-values are for the between-groups at baseline by chi-square test for categorical variables, unpaired Student’s t-test or Wilcoxon rank sum test for continuous variables. Abbreviations: BMI, body mass index; NYHA, New York Heart Association class; NT-proBNP, N-terminal pro-brain natriuretic peptide; BNP, brain natriuretic peptide; eGFR, estimated glomerular filtration rate; 8-OHdG, 8-hydroxy-2’-deoxyguanosine; L-FABP, liver-type fatty acid-binding protein; MDA-LDL, malondialdehyde-modified low density lipoprotein; XOR, xanthine oxidoreductase; LVEF, left ventricular ejection fraction; E, peak early diastolic flow velocity at mitral valve leaflet; e’, early diastolic mitral annular motion velocity; E/e’, ratio of peak early diastolic flow velocity at mitral valve leaflet by early diastolic mitral annular motion velocity; TRPG, transtricuspid pressure gradient; FMD, flow-mediated dilation; RHI, reactive hyperemia index; ARBs, angiotensin receptor blockers; ACEs, angiotensin-converting enzyme inhibitors; V2 receptor antagonist, vasopressin V2 receptor antagonist.

### Study endpoint analyses

An ANCOVA analysis was performed for the percent change in the NT-proBNP level at week 24, which is the primary endpoint, was comparable between the topiroxostat and allopurinol groups in both FAS (adjusted mean ± standard error: 0.6 ± 1.1 versus 1.2 ± 1.1%; P = 0.20, n = 132) and PPS (adjusted mean ± standard error: 0.4 ± 1.2 versus 0.8 ± 1.1%; P = 0.20, P = 0.42, n = 124). In addition, a sensitivity analysis was performed using MMRM for, the percent change in the NT-proBNP level at week 24 was also comparable between the topiroxostat and allopurinol groups in both FAS (adjusted mean ± standard error: 1.1 ± 1.1 versus 0.5 ± 1.1%; P = 0.25, n = 137) and PPS (adjusted mean ± standard error: 0.9 ± 1.1 versus 0.2 ± 1.1; P = 0.42, n = 129). Furthermore, Table 2 and S2 Table show changes in study endpoints in FAS and PPS, respectively using the unpaired *t*-test analysis. The percent change in the NT-proBNP level at week 24 was comparable between the topiroxostat and allopurinol groups in both FAS (1.6±8.2 versus -0.4±8.0%, P = 0.17) and PPS (1.3±8.1 versus 0.1±7.6%, P = 0.39) analyses.

**Table 2:** Changes in study endpoints in FAS analysis.

	Topiroxostat		Allopurinol		P Value
	n	Mean±SD	n	Mean±SD	(T versus A)
**Primary endpoint**					
Percent change in ln (NT-proBNP) at week 24, %	66	1.6±8.2	66	-0.4±8.0	0.17
**Secondary endpoint**					
Percent change in ln (NT-proBNP) at week 12, %	65	1.0±7.0	68	-0.3±7.1	0.28
Percent changes in ln (BNP), %					
At week 12	67	1.1±9.7	69	0.0±10.7	0.55
At week 24	65	1.7±12.3	66	-0.2±10.8	0.34
Change in FMD at week 24, %	30	-0.01±1.40	30	-0.22±1.53	0.59
Change in RHI at week 24	64	-0.04±0.47	62	-0.01±0.66	0.78
Change in uric acid level at week 24, mg/dL	65	-2.6±1.5	66	-2.2±1.3	0.08
Changes in specific biomarkers at week 24					
Troponin I, ln (pg/mL)	66	0.11±0.46	66	0.03±0.46	0.30
Urinary 8-OHdG, ng/mg•Cr	66	1.0±3.6	66	3.0±3.1	<0.001
Urinary L-FABP, ln (μg/g•Cr)	66	0.02±0.68	66	0.20±0.74	0.17
Urinary albumin, ln (μg/g•Cr)	65	-0.13±0.85	66	0.04±1.03	0.29
MDA-LDL, U/L	66	-4.6±26.9	66	-8.1±24.1	0.43
XOR activity, ln (pmol/h/mL)	66	-1.0±0.8	66	-0.8±0.7	0.10
Changes in echocardiographic parameters					
LVEF, %	65	-0.4±5.6	63	1.6±5.3	0.040
E, cm/sec	66	-4.2±16.7	66	5.3±21.9	0.006
E/e’	54	-1.2±4.4	62	0.3±5.4	0.10
TRPG, mmHg	52	1.3±5.5	45	2.7±8.0	0.31

Values are mean ± standard deviation. P values are analyzed for differences between the two groups by the unpaired t-test.

As for the secondary endpoint, the reduction in uric acid level at week 24 was greater in the topiroxostat group, compared to the allopurinol group, in the PPS analysis (-2.7±1.5 versus -2.2±1.2 mg/dL, P = 0.042), although it was comparable between both groups in the FAS analysis (-2.6±1.5 versus -2.2±1.3 mg/dL, P = 0.08). The change in the urinary 8-OHdG level at week 24 was less in the topiroxostat group, compared to the allopurinol group, in both FAS (1.0±3.6 versus 3.0±3.1 ng/mg·Cr, P<0.001) and PPS (0.8±3.7 versus 3.1±3.1 ng/mg·Cr, P<0.001) analyses. Regarding echocardiographic parameters, the change in LVEF at week 24 showed a significant difference between both the topiroxostat and allopurinol groups in the FAS analysis (-0.4±5.6 versus 1.6±5.3%, P = 0.040), but not in the PPS analysis (-0.1±5.5 versus 1.4±5.2%, P = 0.13). The change in E value at week 24 showed significant differences between both groups in both FAS (-4.2±16.7 versus 5.3±21.9 cm/sec, P = 0.006) and PPS (-3.4±17.0 versus 6.1±22.0 cm/sec, P = 0.008) analyses. The change in other parameters for endpoint analyses, including FMD and RHI values changes, showed no significant intergroup differences in both FAS and PPS analyses. In all patients, there was a strong correlation between percent change in NT-proBNP and BNP levels at week 24 in both FAS and PPS analyses. There was a correlation trend between the FMD and RHI values in the FAS analysis, but not in the PPS analysis (S3 Table).

Regarding the safety endpoint, there was no significant difference in each parameter between the topiroxostat and allopurinol groups (Table 3).

**Table 3:** Safety endpoint.

	Topiroxostat (n = 70)	%	Allopurinol (n = 70)	%	P Value
Adverse events, n (%)	21	30.0	14	20.0	0.17
Serious adverse events, n (%)	6	8.6	5	7.1	0.75
Unknown adverse events, n (%)	5	7.1	4	5.7	–
Probably related adverse events, n (%)	1	1.4	0	0.0	–
Serious adverse events necessitating withdrawal from the study, n (%)	1	1.4	2	2.9	–
Serious cardiovascular adverse events, n (%)	3	4.3	3	4.3	–
Gouty arthritis, n (%)	1	1.4	0	0.0	–
Hepatic function abnormality, n (%)	1	1.4	1	1.4	–

Values are n (percentage of patients). P-values are for the between-groups difference by Wilcoxon rank-sum test.

### Other secondary endpoint analyses

**Fig 2:**
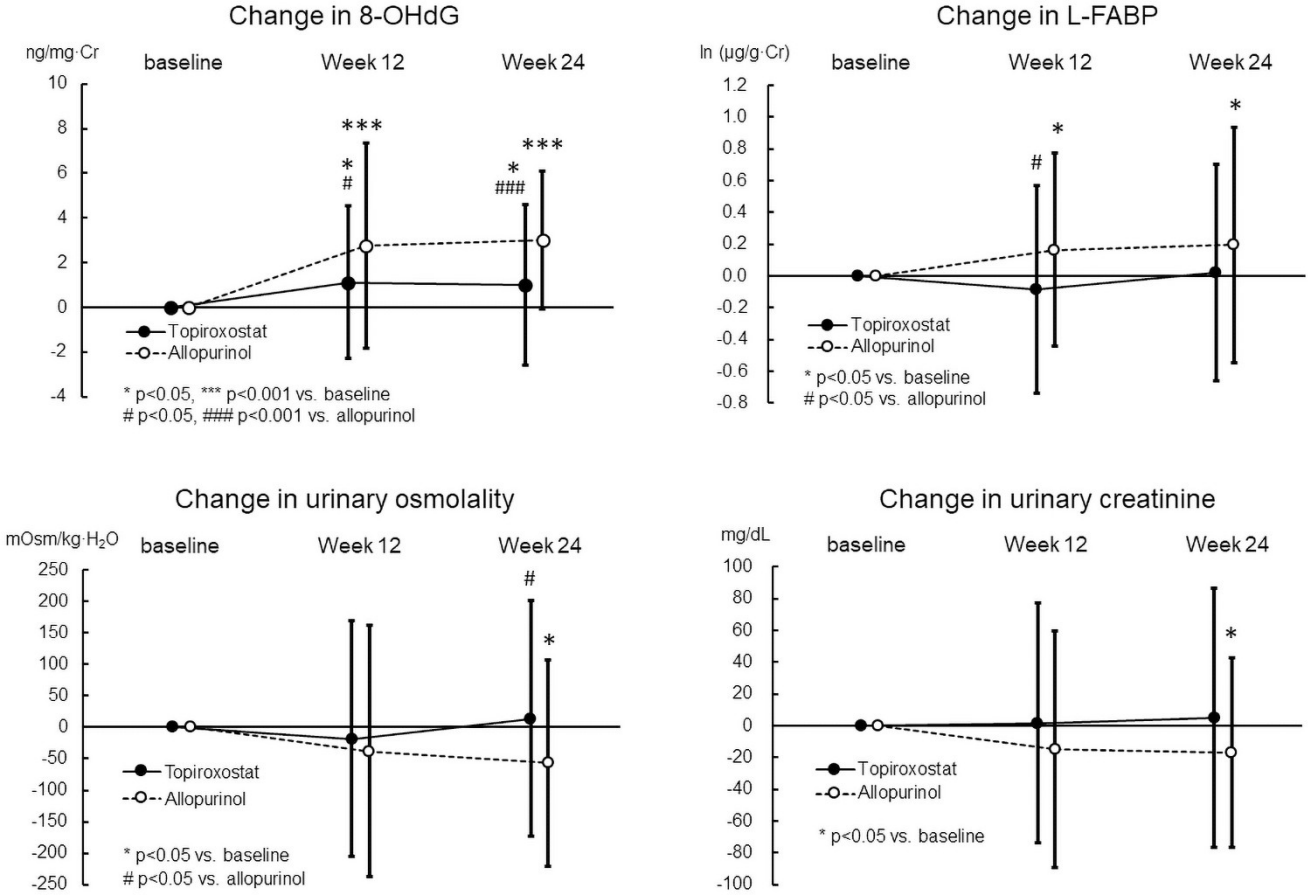
Serial changes of urinary 8-OHdG, L-FABP, osmolality and creatinine levels at weeks 12 and 24. Values are mean ± standard deviation. For the changes in the values 24 weeks from baseline were assessed using the paired Student’s t-test in each group. For the difference between both groups analyzed using the unpaired t-test.

Fig 2 shows serial changes in the value of urinary 8-OHdG, L-FABP, osmolality, and creatinine levels at weeks 12 and 24 from the baseline, which was zero, in the FAS analysis. Urinary 8-OHdG level increased significantly at weeks 12 (2.8±4.6 ng/mg·Cr, P<0.001) and 24 (3.0±3.1 ng/mg·Cr, P<0.001) in the allopurinol group, while it increased less significantly at weeks 12 (1.1±3.4 ng/mg·Cr, P = 0.011) and 24 (1.0±3.6 ng/mg·Cr, P = 0.028) in the topiroxostat group. The difference in the change in urinary 8-OHdG level between both groups was statistically significant at weeks 12 (P = 0.021) and 24 (P<0.001). Urinary L-FABP level increased significantly at weeks 12 [0.16±0.61 ln (μg/g·Cr), P = 0.029] and 24 [0.20±0.74 ln (μg/g·Cr), P = 0.036] in the allopurinol group, while it did not change in the topiroxostat group. The difference in the change in urinary L-FABP level between both groups was statistically significant at week 12 (P = 0.026). Urinary osmolality and creatinine level decreased significantly at week 24 in the allopurinol group (n = 54, -57±164 mOsm/kg·H_2_O, P = 0.015, n = 54, -17±60 mg/dL, P = 0.042, respectively), while they did not change in the topiroxostat group. Differences in the change at week 24 between both groups were statistically significant for the urinary osmolality (P = 0.038), but not for the creatinine level.

### Subgroup analyses

**Fig 3:**
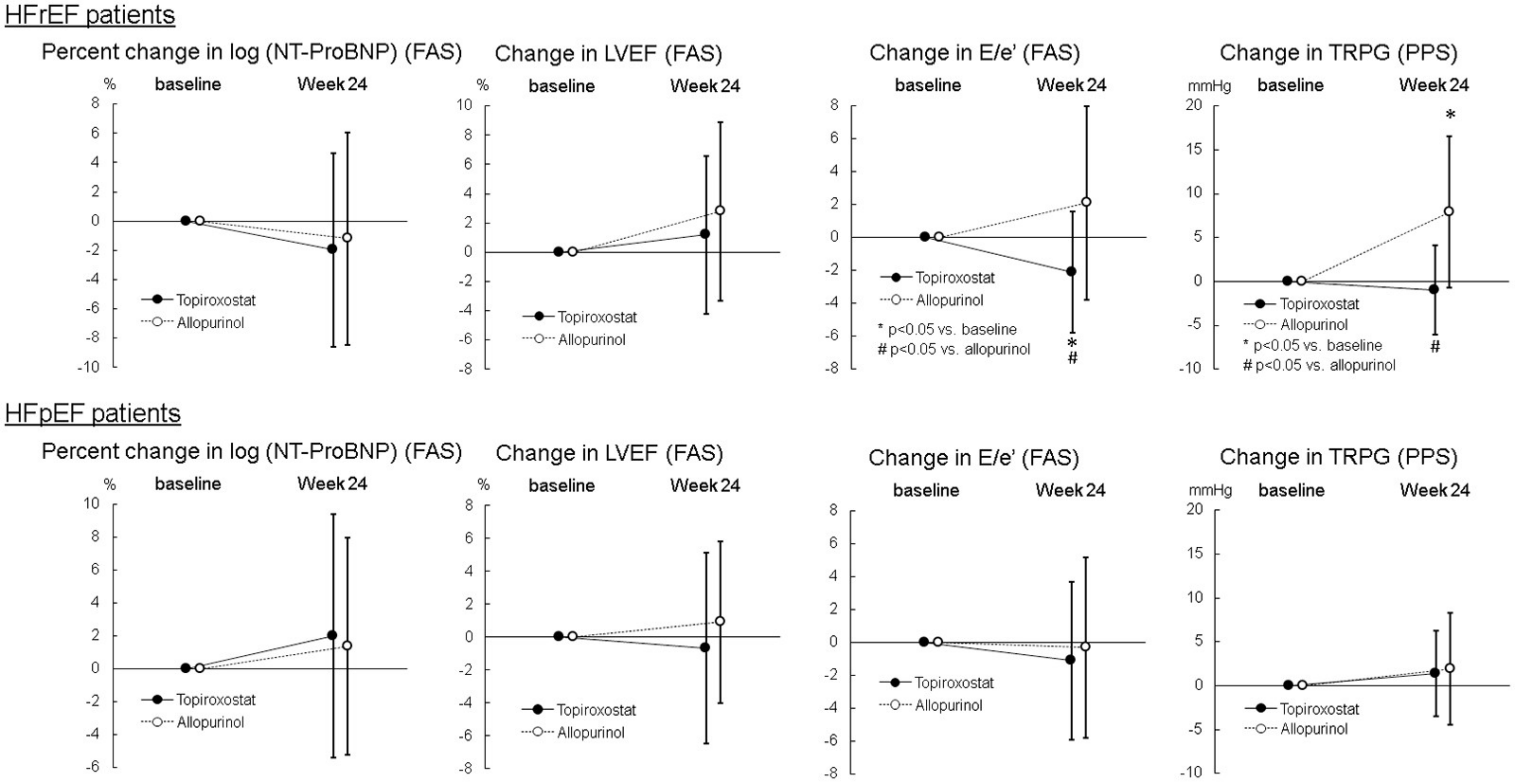
Changes in NT-proBNP, LVEF, E/e’ (FAS), and TRPG (PPS) in each HFrEF and HFpEF patients. Values are mean ± standard deviation. For the changes in the values 24 weeks from baseline were assessed using the paired Student’s t-test in each group. For the difference between both groups analyzed using the unpaired t-test. Abbreviations: HFrEF, heart failure with preserved ejection fraction; HFpEF, heart failure with preserved ejection fraction.

With regard to primary endpoint, echocardiographic parameters and urinary levels of 8-OHdG, L-FABP, osmolality and creatinine, post-hoc analyses were performed in each of HFrEF (n = 39, LVEF; 34±7%, NYHA class I/II; n = 24/15) and HFpEF (n = 87, LVEF; 60±6%, NYHA class I/II; n = 59/28) patient. Changes in NT-proBNP level and echocardiographic parameters at week 24 in subgroup analyses are shown in S4 and S5 Tables and Fig 3. In HFrEF patients, NT-proBNP did not change significantly at week 24 in both topiroxostat and allopurinol groups in FAS analysis, and the percent change in NT-proBNP level at week 24 was comparable between the topiroxostat and allopurinol groups in both FAS and PPS analyses. LVEF did not change significantly at week 24 in both groups of topiroxostat and allopurinol in FAS analysis, and the change in LVEF was comparable between both groups in both FAS and PPS analyses. E/e’ decreased significantly at week 24 from the baseline value (-2.1±3.7, P = 0.04997) in the topiroxostat group, while it did not change in the allopurinol group (2.1±5.9), in the FAS analysis. Significant differences in E/e’ change was observed between both groups in both FAS (P = 0.026) and PPS analyses (P = 0.022). Additionally, TRPG increased significantly at week 24 (7.9±8.6 mmHg, P = 0.018) in the allopurinol group, while it did not change in the topiroxostat group (-1.0±5.1 mmHg), in the PPS analysis. Significant differences in the change in TRPG were present between both groups in the PPS analysis (P = 0.012). In HFpEF patients, NT-proBNP did not change significantly at week 24 in both topiroxostat and allopurinol groups in FAS analysis, and the percent change in NT-proBNP level at week 24 was comparable between the topiroxostat and allopurinol groups in both FAS and PPS analyses. LVEF did not change significantly at week 24 in both topiroxostat and allopurinol groups in FAS analysis, and the change in LVEF was comparable between both groups in both FAS and PPS analyses. A significant difference between the topiroxostat and allopurinol groups was observed in the change in echocardiographic E value in both FAS (P = 0.022) and PPS (P = 0.030) analyses. E/e’ did not change in both groups of topiroxostat and allopurinol in FAS analysis, and the change in E/e’ was between both groups in both FAS and PPS analyses.

**Fig 4:**
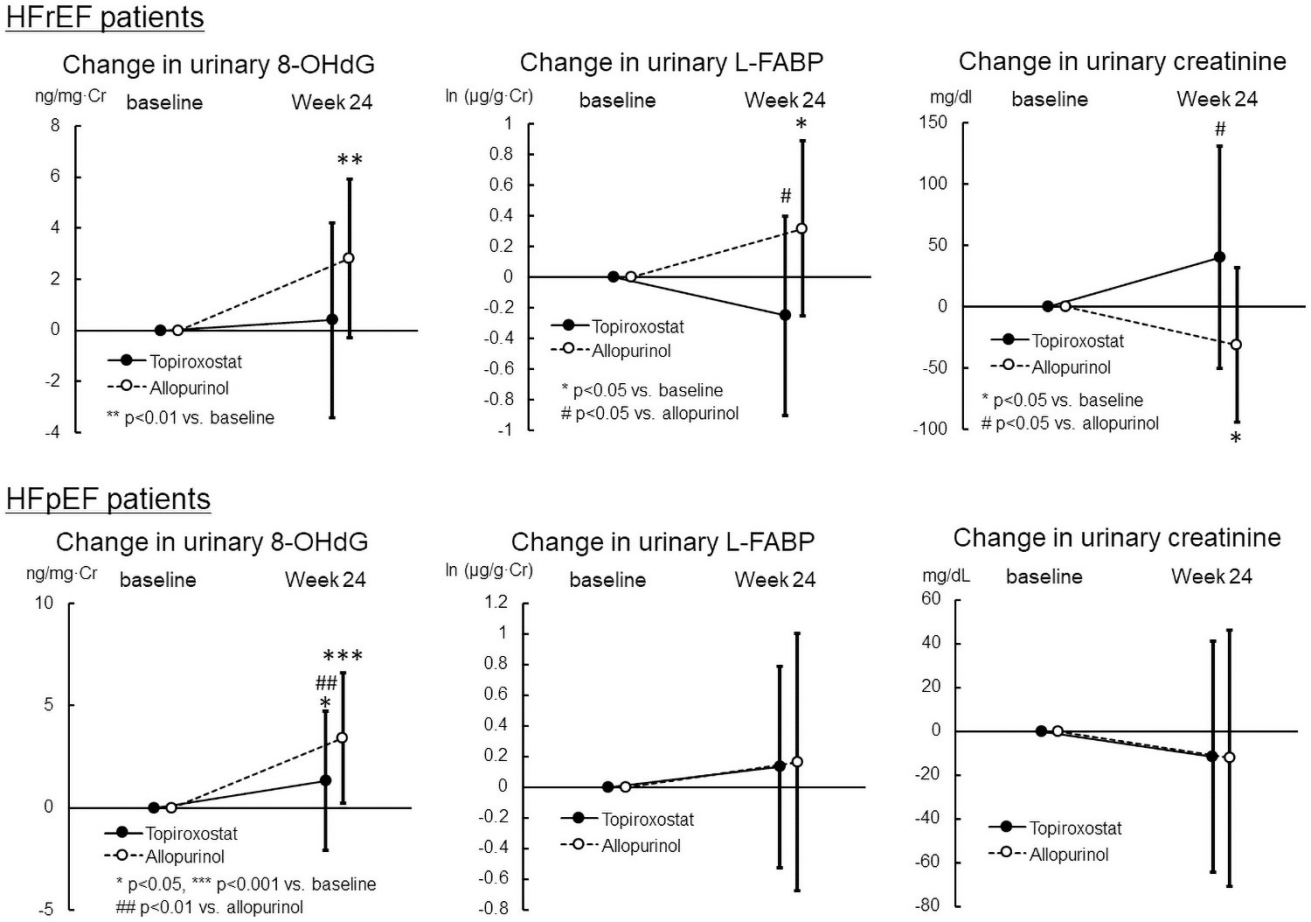
Changes in urinary 8-OHdG, L-FABP and creatinine levels in each HFrEF and HFpEF patients (FAS). Values are mean ± standard deviation. For the changes in the values 24 weeks from baseline were assessed using the paired Student’s t-test in each group. For the difference between both groups analyzed using the unpaired t-test.

Next, we assessed the changes in urinary 8-OHdG, L-FABP, osmolarity, and creatinine levels 24 weeks from the baseline in each HFrEF and HFpEF patient (S6 and S7 Tables, Fig 4). In the HFrEF patients, the urinary 8-OHdG level increased significantly in the allopurinol group (2.8±3.1 ng/mg·Cr, P = 0.001), but not in the topiroxostat group (0.4±3.8 ng/mg·Cr) in the FAS analysis. The urinary L-FABP increased significantly in the allopurinol group [0.32±0.57 ln (μg/g·Cr), P = 0.024], but not in the topiroxostat group [-0.25±0.65 ln (μg/g·Cr)] in the FAS analysis. The difference in the change was statistically significant in both FAS (P = 0.011) and PPS (P = 0.017) analyses. The urinary creatinine level decreased significantly in the allopurinol group (-31±63 mg/dL, P = 0.044), but not in the topiroxostat group (40±91 mg/dL), in the FAS analysis. The difference in the change was statistically significant in both FAS (P = 0.011) and PPS (P = 0.026) analyses. On the other hand, no intergroup difference in the change in creatinine clearance was found between the topiroxostat and allopurinol groups in both FAS (n = 14, -4.0±7.6 versus n = 19, -2.6±8.5 mL/min, P = 0.62) and PPS (n = 13, -3.5±7.7 versus n = 17, -2.7±7.3 mL/min, P = 0.76) analyses. In the HFpEF patients, FAS analysis showed that the urinary 8-OHdG level increased significantly in the topiroxostat group (1.3±3.4 ng/mg·Cr, P = 0.011), and more significantly in the allopurinol group (3.4±3.2 ng/mg·Cr, P<0.001). The intergroup difference in the change was statistically significant in both FAS (P = 0.005) and PPS (P = 0.003) analyses. Urinary L-FABP levels and osmolality did not change in both topiroxostat and allopurinol groups. Urinary osmolality did not change significantly in the topiroxostat and allopurinol groups, and there was no intergroup difference in the changes in both HFpEF and HFrEF patients in both FAS and PPS analyses.

## Discussion

In the present study, the main finding was the primary endpoint result; the percent change in NT-ProBNP level at week 24 was comparable between the topiroxostat and allopurinol groups. The primary endpoint setting was based on prior studies, which demonstrated that topiroxostat, but not allopurinol, reduced BNP or NT-proBNP levels in patients with chronic heart failure and hyperuricemia [18, 19]. Therefore, our result is contrary to our hypothesis that topiroxostat could have superior effects to allopurinol in patients with chronic heart failure and hyperuricemia. One of the reasons might be that the major population of the present study belonged to mild heart failure because of the definition of heart failure as the BNP level≥40 pg/mL. Considering the result of the CARES trial, febuxostat increased cardiovascular mortality and all-cause mortality, compared with allopurinol; however, the result that topiroxostat was not inferior to allopurinol in percent change in the NT-ProBNP level, in addition to the safety endpoint analysis results, might suggest its safety in patients with chronic heart failure.

Regarding secondary endpoints, in FAS analysis, change in LVEF, one echocardiographic parameter result, at week 24 showed a significant difference between both groups and may rather suggest topiroxostat inferiority to allopurinol. In the subgroup analyses for each of the HFrEF and HFpEF patient, the change in LVEF was comparable between the topiroxostat and allopurinol groups. In the limited HFrEF patients, however, the E/e’ decreased significantly at week 24 in the topiroxostat group, while it did not change in the allopurinol group. Additionally, the change in E/e’ was significantly different between both groups. It seems difficult to explain the mechanism for this phenomenon clearly. Generally, it was believed that E/e’ represents left ventricular end-diastolic pressure (LVEDP) in heart failure patients. However, reportedly the correlation between E/e’ and LVEDP is absent in patients with severe HFrEF such as NYHA class III or IV [20, 21]. Since the HFrEF patients in the present study showed only mild heart failure such as NYHA class I or II, our results suggest that topiroxostat, but not allopurinol, might reduce LVEDP, i.e., left ventricular pre-load in mild HFrEF patients. Additional to left ventricular systolic function, LVEDP is an important measure of left ventricular performance and may identify patients at increased risk for developing late clinical symptoms of heart failure [22, 23]; so topiroxostat might have a potential advantage over allopurinol in HFrEF patients. On the other hand, E/e’ is a very important determinant factor for pathophysiology of HFpEF. Nevertheless, in the present study, topiroxostat did not affect E/e’ in the HFpEF patients. It is unclear why the E/e’ reduction by topiroxostat was shown in the HFrEF patients but not in the HFpEF patients. It is generally expected that NT-proBNP or BNP will also decrease due to a reduction of preload associated with the improvement of LVEDP; however, no clear effect was observed in this study. This may be due to the possibility that the relationship between E/e’ and NT-proBNP or BNP may differ in HFrEF and HFpEF. In previous reports, a correlation between E/e’ and BNP was observed in diastolic dysfunction, while no correlation was observed between E/e’ and BNP in systolic dysfunction [24]. In the present study, a decrease in E/e’ was observed in HFrEF, while no decrease in E/e’ was observed in HFpEF, suggesting that a decrease in BNP could not be observed.

Additionally, TRPG, which represents the right cardiac function [25], increased significantly at week 24 in the allopurinol group, while it did not change in the topiroxostat group. Significant differences in the change in TRPG were present between both groups. The result suggests a possibility that topiroxostat, but not allopurinol, could prevent right cardiac failure aggravation in HFrEF patients.

A noteworthy finding of the present study is the advantage of topiroxostat over allopurinol on renal oxidative stress and renal tubular damages. Renoprotective effects of allopurinol have been well known [26]. Also, allopurinol has been believed to act as an anti-oxidant [27], but on the other hand, its adverse impact on redox balance has also been reported [28]. Moreover, the impact of allopurinol on renal oxidative stress in humans has so far not been reported. In the present study, urinary levels of an oxidative stress marker, 8-OHdG, and a renal proximal tubular oxidative stress marker, L-FABP, increased significantly in the allopurinol group. On the other hand, the levels increased less or not significantly in the topiroxostat group. The difference in the change in both levels was significant between both groups. These results suggest that allopurinol, but not topiroxostat, accelerated renal oxidative stress, resulting in renal tubular damage. The purine-analog xanthine oxidoreductase inhibitor allopurinol is metabolized to allopurinol-1-ribotide, with the consumption of 5-phosphoribosyl-1-pyrophosphate, decreasing the production of the energy charger inosine monophosphate, in the purine salvage pathway in cells. Consequently, adenosine triphosphate, which is formed from inosine monophosphate via adenosine monophosphate, is reduced so that functions in oxidative stress elimination in cells such as glutathione synthesis and the ubiquitin-proteasome system, are decreased. Therefore, allopurinol cannot suppress accumulation of oxidative stress in cells, as shown in the urinary 8-OHdG level. On the other hand, a nonpurine-analog xanthine oxidoreductase inhibitor, topiroxostat, can enhance the purine salvage pathway, thereby helping to promote the removal of oxidative stress [29]. Aquaporin 1, a water channel protein, and claudin-2, a component of the tight junction, play roles in water transport in the proximal renal tubule [30, 31]. Oxidative stress in proximal tubular epithelial cells partially contributes to water reabsorption dysfunction in proximal renal tubule possibly through the reduction of aquaporin 1 and/or claudin-2 [32, 33], which leads to decrease in urinary osmolality. In the present study, urinary osmolality decreased significantly at week 24 in the allopurinol group, while it did not change in the topiroxostat group. The result suggests that allopurinol-induced oxidative stress might decrease urinary osmolality, possibly via the reduction of aquaporin 1 and/or claudin-2 in proximal renal tubule in patients with heart failure, and topiroxostat might have potential advantages of not worsening oxidative stress. Additionally, the intergroup difference in the change in L-FABP and urinary creatinine levels was prominent in the HFrEF patients, but not the HFpEF patients. Therefore, undesirable effects of allopurinol such as oxidative stress-related renal tubular damages and topiroxostat advantages in same terms might be stronger in the HFrEF patients, compared with HFpEF patients. Since the renoprotective and anti-oxidant effects of topiroxostat have been suggested in previous reports [34, 35], our results might merely support previous data. However, it is a novel data of our study that these effects of topiroxostat were shown in patients with heart failure, especially in HFrEF patients. In addition, it may be suggested the beneficial effect that topiroxostat decreased E/e’ in HFrEF patients but not in HFpEF patients is associated with its renoprotective effect dominantly existing in HFrEF patients, from a perspective of cardio-renal connection. Taken together, we believe our data could provide a novel therapeutic approach for chronic heart failure with hyperuricemia.

### Study limitations

The present study has several potential limitations. In this study, topiroxostat was not inferior to allopurinol in terms of the change in NT-ProBNP level, but the noninferiority has not been proven. The sample size calculated to determine topiroxostat superiority was too small to prove its noninferiority. Likewise, our results of subgroup analyses have a limitation of small sample size. Also, the small sample size limited a discussion regarding dose response of topiroxostat and allopurinol, i.e., constant dose or increased dose, which would be a very important issue, because the number of the patients treated at gradually increased dose was too small in both treatment groups. In addition, comparatively a large number of patients with atrial fibrillation in the background population of this study may have been one of the factors that influenced the results. Finally, in this study, all the endpoint analyses were performed at weeks 12 and 24, so we could assess only the short-term effects of xanthine oxidoreductase inhibitors using surrogate endpoints alone, and we cannot predict long-term effects only from our results. Therefore, larger size event-driven trials targeting long-term drug effects are needed.

### Clinical implication/conclusion

Since the establishment of beta-blockers and renin-angiotensin-aldosterone system inhibitors as chronic heart failure treatment agents that can improve long-term prognosis, no novel drugs as a breakthrough for chronic heart failure have emerged. Recently, new agents such as neprilysin inhibitor, sacubitril, or hyperpolarization-activated cyclic nucleotide-gated channel inhibitor, ivabradine, have emerged, and their efficacy for improving long-term prognosis in patients with chronic heart failure is expected. On the other hand, therapeutic approaches targeting complications of chronic heart failure such as hypertension, diabetes, hyperuricemia, etc., which also have beneficial effects on heart failure, are rational.

Regarding the primary endpoint in the present study, we could not show the superiority of topiroxostat to allopurinol on NTproBNP levels in patients with chronic heart failure and hyperuricemia. In a previous study, which demonstrated the effect of topiroxostat on BNP level reduction, it is described that the BNP level decreased more potently by treatment with topiroxostat in patients with higher baseline level. Especially in patients with BNP level≥500 pg/mL at baseline, the level decreased by over 200 pg/mL [18]. In contrast, in the present study, the majority of patients exhibited mild heart failure. Therefore, we need to compare the effect of topiroxostat on NTproBNP level with that of allopurinol in patients with higher BNP level at baseline, in the future. Although the primary endpoint result was contrary to expectation, subgroup analysis results showed that topiroxostat might have potential advantages of reducing LVEDP, not worsening renal oxidative stress, and renoprotection over allopurinol in patients with chronic heart failure, especially in HFrEF patients. From this perspective, topiroxostat would be promising for the treatment of chronic heart failure complicated with hyperuricemia, although further investigations are needed.

## Supporting information

S1 Table Detailed observations/measurements. (DOCX)

S2 Table Changes in study endpoints in PPS analysis. Values are mean ± standard deviation. P values are analyzed for differences between the two groups by the unpaired t-test.
(DOCX)

S3 Table Correlations between percent change in NT-proBNP and BNP levels and between FMD and RHI values in FAS and PPS analyses. For correlation analyses, Pearson’s product-moment correlation coefficient or Spearman’s’ rank-correlation coefficient was calculated to assess significance.
(DOCX)

S4 Table Changes in NT-proBNP level and echocardiographic parameters in patients with HFrEF in FAS and PPS analyses. Values are mean ± standard deviation. P values are analyzed for differences between the two groups by the unpaired t-test.
(DOCX)

S5 Table Changes in NT-proBNP level and echocardiographic parameters in patients with HFpEF in FAS and PPS analyses. Values are mean ± standard deviation. P values are analyzed for differences between the two groups by the unpaired t-test.
(DOCX)

S6 Table Changes in urinary 8-OHdG, L-FABP, osmolality and creatinine in patients with HFrEF in FAS and PPS analyses. Values are mean ± standard deviation. P values are analyzed for differences between the two groups by the unpaired t-test.
(DOCX)

S7 Table Changes in urinary 8-OHdG, L-FABP, osmolality and creatinine in patients with HFpEF in FAS and PPS analyses. Values are mean ± standard deviation. P values are analyzed for differences between the two groups by the unpaired t-test.
(DOCX)

S1 Appendix Analysis report 1. (PDF)

S2 Appendix Analysis report 2. (PDF)

S3 Appendix Analysis report 3. (PDF)

S4 Appendix Graphical inspection of the residuals plot in FAS. (PDF)

S1 Checklist (PDF)
