# Supplementary material for: Topiroxostat versus allopurinol in patients with chronic heart failure complicated by hyperuricemia: A prospective, randomized, open-label, blinded-end-point clinical trial
Source: PLoS One. 2022 Jan 25;17(1):e0261445. doi: 10.1371/journal.pone.0261445 (PMC8789120; doi:10.1371/journal.pone.0261445)
Supplement: S1 Table — (DOCX) [file pone.0261445.s001.docx]

**S1 Table. Detailed observations/measurements.**

- Baseline

Height, body mass index, complications such as hypertension, diabetes mellitus, dyslipidemia, chronic kidney disease, atrial fibrillation, arrhythmia episodes, NYHA classification, heart failure etiology, and gout episodes, and medications

- Each visit

Body weight, medication status, blood pressure, and heart rate

- Baseline, weeks 12 and 24

General blood and urine tests and BNP (each hospital)

NT-proBNP and MDA-LDL (LSI Medience Corp., Tokyo, Japan)

Troponin I (Abbott Japan Co., Ltd., Tokyo, Japan)

Urinary 8-OHdG, L-FABP, and albumin excretion (LSI Medience Corp., Tokyo, Japan)

- Baseline, week 24

Xanthine oxidoreductase activity (Mie Research Laboratories, Sanwa Kagaku Kennkyusho Co. Ltd., Inabe, Japan)

Chest X-ray, electrocardiogram, and echocardiography (each hospital)

FMD and/or RH-PAT (each hospital)

Echocardiographic data, including LVEF, E, E/e’, and TRPG (each hospital)

NYHA, New York Heart Association; BNP, brain natriuretic peptide; NT-pro BNP, N-terminal pro-brain natriuretic peptide; hsCRP, high-sensitivity C-reactive protein; MDA-LDL, malondialdehyde-modified low density lipoprotein; 8-OHdG, 8-hydroxy-2'-deoxyguanosine; L-FABP, liver type fatty acid-binding protein; FMD, flow-mediated dilation; RH-PAT, reactive hyperemia peripheral arterial tonometry; LVEF, left ventricular ejection fraction; E, peak early diastolic flow velocity at mitral valve leaflet; e’, early diastolic mitral annular motion velocity; E/e’, E to e' ratio.
