# Supplementary material for: Topiroxostat versus allopurinol in patients with chronic heart failure complicated by hyperuricemia: A prospective, randomized, open-label, blinded-end-point clinical trial
Source: PLoS One. 2022 Jan 25;17(1):e0261445. doi: 10.1371/journal.pone.0261445 (PMC8789120; doi:10.1371/journal.pone.0261445)
Supplement: S2 Table — Values are mean ± standard deviation. P values are analyzed for differences between the two groups by the unpaired t-test. (DOCX) [file pone.0261445.s002.docx]

| **S2 Table. Changes in Study Endpoints in PPS analysis.** | | | | | | | | | |
| --- | --- | --- | --- | --- | --- | --- | --- | --- | --- |
|  | Topiroxostat | |  | Allopurinol | |  | P Value  (T versus A) | |  |
|  | n | Mean±SD |  | n | Mean±SD |  |  |  |  |
| **Primary Endpoint** |  |  |  |  |  |  | |  | |
| Percent change in log (NT-proBNP) at week 24, % | 61 | 1.3±8.1 |  | 63 | 0.1±7.6 |  | 0.39 | |  |
|  |  |  |  |  |  |  |  | |  |
| **Secondary Endpoint** |  |  |  |  |  |  |  | |  |
| Percent change in log (NT-proBNP) at week 12, % | 61 | 0.9±7.0 |  | 65 | -0.3±7.3 |  | 0.36 | |  |
| Percent changes in log (BNP), % |  |  |  |  |  |  |  | |  |
| At week 12 | 61 | 1.0±9.8 |  | 65 | -0.6±10.1 |  | 0.36 | |  |
| At week 24 | 60 | 1.5±12.3 |  | 62 | -0.2±10.2 |  | 0.38 | |  |
| Change in FMD at week 24, % | 28 | 0.13±1.18 |  | 28 | -0.35±1.49 |  | 0.19 | |  |
| Change in RHI at week 24 | 60 | -0.03±0.47 |  | 59 | -0.02±0.67 |  | 0.94 | |  |
| Change in uric acid level at week 24, mg/dL | 60 | -2.7±1.5 |  | 62 | -2.2±1.2 |  | 0.042 | |  |
| Changes in specific biomarkers at week 24 |  |  |  |  |  |  |  | |  |
| Troponin I, log (pg/mL) | 61 | 0.10±0.46 |  | 63 | 0.02±0.46 |  | 0.34 | |  |
| Urinary 8-OHdG, ng/mg·Cr | 61 | 0.8±3.7 |  | 63 | 3.1±3.1 |  | <0.001 | |  |
| Urinary L-FABP, log (μg/g·Cr) | 61 | 0.03±0.71 |  | 63 | 0.20±0.76 |  | 0.21 | |  |
| Urinary albumin, log (μg/g·Cr) | 60 | -0.12±0.87 |  | 63 | 0.06±1.05 |  | 0.30 | |  |
| MDA-LDL, U/L | 61 | -3.0±26.4 |  | 63 | -9.2±21.6 |  | 0.15 | |  |
| XOR activity, log (pmol/h/mL) | 61 | -1.1±0.8 |  | 63 | -0.8±0.7 |  | 0.10 | |  |
| Changes in echocardiographic parameters |  |  |  |  |  |  |  | |  |
| LVEF, % | 60 | -0.1±5.5 |  | 61 | 1.4±5.2 |  | 0.13 | |  |
| E, cm/sec | 61 | -3.4±17.0 |  | 63 | 6.1±22.0 |  | 0.008 | |  |
| E/e' | 49 | -1.3±4.6 |  | 59 | 0.4±5.5 |  | 0.08 | |  |
| TRPG, mmHg | 49 | 0.9±5.3 |  | 43 | 3.1±7.3 |  | 0.10 | |  |

PPS, per-protocol set; NT-proBNP, N-terminal pro-brain natriuretic peptide; BNP, brain natriuretic peptide; FMD, flow-mediated dilation; RHI, reactive hyperemia index; 8-OHdG, 8-hydroxy-2'-deoxyguanosine; L-FABP, liver-type fatty acid-binding protein; MDA-LDL, malondialdehyde-modified low density lipoprotein; XOR, xanthine oxidoreductase; LVEF, left ventricular ejection fraction; E, peak early diastolic flow velocity at mitral valve leaflet; e’, early diastolic mitral annular motion velocity; E/eacid-binding E to e' ratio; TRPG, transtricuspid pressure gradient.
