# Supplementary material for: Topiroxostat versus allopurinol in patients with chronic heart failure complicated by hyperuricemia: A prospective, randomized, open-label, blinded-end-point clinical trial
Source: PLoS One. 2022 Jan 25;17(1):e0261445. doi: 10.1371/journal.pone.0261445 (PMC8789120; doi:10.1371/journal.pone.0261445)
Supplement: S3 Table — For correlation analyses, Pearson’s product-moment correlation coefficient or Spearman’s’ rank-correlation coefficient was calculated to assess significance. (DOCX) [file pone.0261445.s003.docx]

| **S3 Table. Correlations** **Between Percent Change in NT-proBNP and BNP Levels and Between FMD and RHI Values** **in FAS and PPS Analyses.** | | | | | | | | | | | |  |
| --- | --- | --- | --- | --- | --- | --- | --- | --- | --- | --- | --- | --- |
|  |  | |  | |  |  | |  |  |  |  | |
| **FAS Analysis** |  | |  | |  |  | |  |  |  |  | |
|  | n | | Pearson’s method | | | | |  | Spearman’s method | |  | |
|  |  |  | Correlation coefficient (95% CI) | | | P Value | |  | Correlation coefficient (95% CI) | | P Value | |
| Correlation between percent change in  NT-proBNP and BNP levels at week 24 | 130 | | 0.84 (0.77, 0.88) | | | <0.001 | |  | 0.72 (0.63, 0.79) | | <0.001 | |
| Correlation between change in FMD and  RHI values at week 24 | 60 | | 0.25 (-0.01, 0.47) | | | 0.06 | |  | 0.26 (0.00, 0.48) | | 0.048 | |
|  | |  | |  |  |  | |  |  |  |  | |
| **PPS Analysis** |  | |  | |  |  |  | |  |  |  | |
|  | n | | Pearson’s method | | | | |  | Spearman’s method | |  | |
|  |  |  | Correlation coefficient (95% CI) | | | P Value | |  | Correlation coefficient (95% CI) | | P Value | |
| Correlation between percent change in NT-proBNP and BNP levels at week 24 | 122 | | 0.83 (0.76, 0.88) | | | <0.001 | | 0.69 (0.59, 0.77) | | | <0.001 | |
| Correlation between change in FMD and  RHI values at week 24 | 56 | | 0.18 (-0.09, 0.42) | | | 0.18 | | 0.21 (-0.06, 0.45) | | | 0.12 | |

FAS, full analysis set; PPS, per-protocol set; CI, confidence interval; NT-proBNP, N-terminal pro-brain natriuretic peptide; BNP, brain natriuretic peptide; FMD, flow-mediated dilation; RHI, reactive hyperemia index.
