# Supplementary material for: Topiroxostat versus allopurinol in patients with chronic heart failure complicated by hyperuricemia: A prospective, randomized, open-label, blinded-end-point clinical trial
Source: PLoS One. 2022 Jan 25;17(1):e0261445. doi: 10.1371/journal.pone.0261445 (PMC8789120; doi:10.1371/journal.pone.0261445)
Supplement: S4 Table — Values are mean ± standard deviation. P values are analyzed for differences between the two groups by the unpaired t-test. (DOCX) [file pone.0261445.s004.docx]

| **S4 Table. Changes in NT-proBNP Level and Echocardiographic Parameters in Patients with HFrEF in FAS and PPS Analyses.** | | | | | | | |  |
| --- | --- | --- | --- | --- | --- | --- | --- | --- |
|  | Topiroxostat | |  | Allopurinol | |  | P Value  (T versus A) | |
|  | n | Mean±SD |  | n | Mean±SD |  |  |  |
| **FAS Analysis** |  |  |  |  |  |  |  | |
| Percent change in log (NT-proBNP) at week 24, % | 15 | -2.0±6.6 |  | 19 | -1.2±7.3 |  | 0.75 | |
| Changes in echocardiographic parameters at week 24 |  |  |  |  |  |  |  | |
| LVEF, % | 15 | 1.2±5.4 |  | 19 | 2.8±6.1 |  | 0.45 | |
| E, cm/sec | 15 | -4.5±18.3 |  | 19 | 9.5±30.7 |  | 0.13 | |
| E/e' | 15 | -2.1±3.7 |  | 18 | 2.1±5.9 |  | 0.026 | |
| TRPG, mmHg | 11 | -0.7±5.0 |  | 11 | 5.3±11.9 |  | 0.14 | |
| **PPS Analysis** |  |  |  |  |  |  |  | |
| Percent change in log (NT-proBNP) at week 24, % | 14 | -1.7±6.8 |  | 17 | -0.9±7.6 |  | 0.74 | |
| Changes in echocardiographic parameters at week 24 |  |  |  |  |  |  |  | |
| LVEF, % | 14 | 1.3±5.6 |  | 17 | 2.4±6.2 |  | 0.62 | |
| E, cm/sec | 14 | -3.3±18.9 |  | 17 | 12.8±30.8 |  | 0.10 | |
| E/e' | 14 | -1.9±3.8 |  | 16 | 2.5±5.8 |  | 0.022 | |
| TRPG, mmHg | 10 | -1.0±5.1 |  | 10 | 7.9±8.6 |  | 0.012 | |

HFrEF, heart failure with reduced ejection fraction; FAS, full analysis set; PPS, per-protocol set; NT-proBNP, N-terminal pro-brain natriuretic peptide; LVEF, left ventricular ejection fraction; E, peak early diastolic flow velocity at mitral valve leaflet; e’, early diastolic mitral annular motion velocity; E/e, E to e' ratio; TRPG, transtricuspid pressure gradient.
