# Supplementary material for: Topiroxostat versus allopurinol in patients with chronic heart failure complicated by hyperuricemia: A prospective, randomized, open-label, blinded-end-point clinical trial
Source: PLoS One. 2022 Jan 25;17(1):e0261445. doi: 10.1371/journal.pone.0261445 (PMC8789120; doi:10.1371/journal.pone.0261445)
Supplement: S5 Table — Values are mean ± standard deviation. P values are analyzed for differences between the two groups by the unpaired t-test. (DOCX) [file pone.0261445.s005.docx]

| **S5 Table. Changes in NT-proBNP Level and Echocardiographic Parameters in Patients with HFpEF in FAS and PPS Analyses.** | | | | | | | |
| --- | --- | --- | --- | --- | --- | --- | --- |
|  | Topiroxostat | |  | Allopurinol | |  | P Value  (T versus A) |
|  | n | Mean±SD |  | n | Mean±SD |  |  |
| **FAS Analysis** |  |  |  |  |  |  |  |
| Percent change in log (NT-proBNP) at week 24, % | 44 | 2.0±7.4 |  | 40 | 1.4±6.6 |  | 0.71 |
| Changes in echocardiographic parameters at week24 |  |  |  |  |  |  |  |
| LVEF, % | 43 | -0.7±5.8 |  | 39 | 0.9±4.9 |  | 0.21 |
| E, cm/sec | 44 | -5.0±16.0 |  | 40 | 3.8±18.4 |  | 0.022 |
| E/e' | 34 | -1.1±4.8 |  | 37 | -0.3±5.5 |  | 0.53 |
| TRPG, mmHg | 36 | 1.9±5.3 |  | 30 | 1.9±6.4 |  | 0.99 |
| **PPS Analysis** |  |  |  |  |  |  |  |
| Percent change in log (NT-proBNP) at week 24 % | 41 | 1.8±7.4 |  | 40 | 1.4±6.6 |  | 0.79 |
| Changes in echocardiographic parameters at week 24 |  |  |  |  |  |  |  |
| LVEF, % | 40 | -0.2±5.5 |  | 39 | 0.9±4.9 |  | 0.36 |
| E, cm/sec | 41 | -4.8±16.4 |  | 40 | 3.8±18.4 |  | 0.030 |
| E/e' | 31 | -1.4±5.0 |  | 37 | -0.3±5.5 |  | 0.43 |
| TRPG, mmHg | 34 | 1.4±4.9 |  | 30 | 1.9±6.4 |  | 0.70 |

HFpEF, heart failure with preserved ejection fraction; FAS, full analysis set; PPS, per-protocol set; NT-proBNP, N-terminal pro-brain natriuretic peptide; LVEF, left ventricular ejection fraction; E, peak early diastolic flow velocity at mitral valve leaflet; e’, early diastolic mitral annular motion velocity; E/e, E to e' ratio; TRPG, transtricuspid pressure gradient.
