# Supplementary material for: Topiroxostat versus allopurinol in patients with chronic heart failure complicated by hyperuricemia: A prospective, randomized, open-label, blinded-end-point clinical trial
Source: PLoS One. 2022 Jan 25;17(1):e0261445. doi: 10.1371/journal.pone.0261445 (PMC8789120; doi:10.1371/journal.pone.0261445)
Supplement: S6 Table — Values are mean ± standard deviation. P values are analyzed for differences between the two groups by the unpaired t-test. (DOCX) [file pone.0261445.s006.docx]

| **S6 Table. Changes in Urinary 8-OHdG, L-FABP, Osmolality and Creatinine in Patients with HFrEF in FAS and PPS Analyses.** | | | | | | | |
| --- | --- | --- | --- | --- | --- | --- | --- |
|  | Topiroxostat | |  | Allopurinol | |  | P Value  (T versus A) |
|  | n | Mean±SD |  | n | Mean±SD |  |  |
| **FAS Analysis** |  |  |  |  |  |  |  |
| Change in urinary 8-OHdG, ng/mg·Cr | 15 | 0.4±3.8 |  | 19 | 2.8±3.1 |  | 0.053 |
| Change of urinary L-FABP, log (μg/g·Cr) | 15 | -0.25±0.65 |  | 19 | 0.32±0.57 |  | 0.011 |
| Change in urinary osmolality, mOsm/kg·H_2_O | 12 | 18±148 |  | 15 | -100±194 |  | 0.09 |
| Change in urinary creatinine, mg/dL | 15 | 40±91 |  | 19 | -31±63 |  | 0.011 |
|  |  |  |  |  |  |  |  |
| **PPS Analysis** |  |  |  |  |  |  |  |
| Change in urinary 8-OHdG, ng/mg·Cr | 14 | 0.2±3.8 |  | 17 | 2.8±3.2 |  | 0.051 |
| Change of urinary L-FABP, log (μg/g·Cr) | 14 | -0.25±0.68 |  | 17 | 0.33±0.60 |  | 0.017 |
| Change in urinary osmolality, mOsm/kg·H_2_O | 11 | 2±143 |  | 13 | -102±186 |  | 0.14 |
| Change in urinary creatinine, mg/dL | 14 | 39±94 |  | 17 | -29±66 |  | 0.026 |
|  |  |  |  |  |  |  |  |

HFrEF, heart failure with reduced ejection fraction; FAS, full analysis set; PPS, per-protocol set; 8-OHdG, 8-hydroxy-2'-deoxyguanosine; L-FABP, liver-type fatty acid-binding protein.
