# Supplementary material for: Topiroxostat versus allopurinol in patients with chronic heart failure complicated by hyperuricemia: A prospective, randomized, open-label, blinded-end-point clinical trial
Source: PLoS One. 2022 Jan 25;17(1):e0261445. doi: 10.1371/journal.pone.0261445 (PMC8789120; doi:10.1371/journal.pone.0261445)
Supplement: S7 Table — Values are mean ± standard deviation. P values are analyzed for differences between the two groups by the unpaired t-test. (DOCX) [file pone.0261445.s007.docx]

| **S7 Table. Changes in Urinary 8-OHdG, L-FABP, Osmolality and Creatinine in Patients with HFpEF in FAS and PPS Analyses.** | | | | | | | |
| --- | --- | --- | --- | --- | --- | --- | --- |
|  | Topiroxostat | |  | Allopurinol | |  | P Value  (T versus A) |
|  | n | Mean±SD |  | n | Mean±SD |  |  |
| **FAS Analysis** |  |  |  |  |  |  |  |
| Change in urinary 8-OHdG, ng/mg·Cr | 44 | 1.3±3.4 |  | 40 | 3.4±3.2 |  | 0.005 |
| Change of urinary L-FABP, log (μg/g·Cr) | 44 | 0.13±0.66 |  | 40 | 0.16±0.84 |  | 0.87 |
| Change in urinary osmolality, mOsm/kg·H_2_O | 36 | 3±190 |  | 34 | -27±151 |  | 0.46 |
| Change in urinary creatinine, mg/dL | 44 | -12±53 |  | 40 | -12±59 |  | 0.95 |
|  |  |  |  |  |  |  |  |
| **PPS Analysis** |  |  |  |  |  |  |  |
| Change in urinary 8-OHdG, ng/mg·Cr | 41 | 1.2±3.4 |  | 40 | 3.4±3.2 |  | 0.003 |
| Change of urinary L-FABP, log (μg/g·Cr) | 41 | 0.13±0.68 |  | 40 | 0.16±0.84 |  | 0.88 |
| Change in urinary osmolality, mOsm/kg·H_2_O | 34 | -0±191 |  | 34 | -27±151 |  | 0.52 |
| Change in urinary creatinine, mg/dL | 41 | -15±53 |  | 40 | -12±59 |  | 0.84 |
|  |  |  |  |  |  |  |  |

HFpEF, heart failure with preserved ejection fraction; FAS, full analysis set; PPS, per-protocol set; 8-OHdG, 8-hydroxy-2'-deoxyguanosine; L-FABP, liver-type fatty acid-binding protein.
