# Supplementary material for: Topiroxostat versus allopurinol in patients with chronic heart failure complicated by hyperuricemia: A prospective, randomized, open-label, blinded-end-point clinical trial
Source: PLoS One. 2022 Jan 25;17(1):e0261445. doi: 10.1371/journal.pone.0261445 (PMC8789120; doi:10.1371/journal.pone.0261445)
Supplement: S1 Appendix — (PDF) [file pone.0261445.s008.pdf]

高尿酸血症を合併した心不全患者における
トピロキソスタットとアロプリノールのランダム化比較試験

The **E**ffect of **X**anthine Oxidase Inhibitor
in **C**hron**I**c heart failure patients
complica**TED** with hyper-**U**ricemi**A**

Excited-UA study

統計解析報告書
Ver 1.1

研究代表者 ：
獨協医科大学病院 心臓･血管内科 教授 井上 晃男

統計解析責任者 ：
株式会社 総合医科学研究所 医薬臨床研究支援事業部 山田 博万

統計解析報告書の作成･変更履歴

日付	版番号	変更点
2019/05/20	1.0	初版作成
2019/05/24	1.1	統計解析報告書 Ver1.1 に対応した解析結果の追加

目次

1. 研究のフローチャート........1
図 1.1. 研究のフローチャート........1

2. 研究対象者の内訳........2
表 2.1. 研究対象者の内訳........2

3. 研究対象者背景........3
表 3.1. [FAS] 研究対象者背景(連続変数)........3
表 3.2. [FAS] 研究対象者背景(カテゴリ変数)........3
表 3.3. [FAS] 心不全の原疾患 / その他の心不全 の詳細........5
表 3.4. [FAS] 既往歴の詳細........6
表 3.5. [FAS] 各合併症の詳細........8

4. 主要評価項目........10

4.1. FAS........10
表 4.1.1. [FAS] 要約統計量と t 検定、Wilcoxon 検定........10
表 4.1.2. [FAS] 共分散分析........10
表 4.1.3. [FAS] MMRM........10

4.2. PPS........11
表 4.2.1. [PPS] 要約統計量と t 検定、Wilcoxon 検定........11
表 4.2.2. [PPS] 共分散分析........11
表 4.2.3. [PPS] MMRM........11

5. 副次評価項目........12

5.1. FAS........12
表 5.1.1. [FAS] 24 週変化率以外も含めた NT-proBNP........12
表 5.1.2. [FAS] BNP........13
表 5.1.3. [FAS] NT-proBNP の変化率と BNP 変化率の相関........14
表 5.1.4. [FAS] FMD........15
表 5.1.5. [FAS] EndoPAT........16
表 5.1.6. [FAS] FMD の変化量と EndoPAT の変化量の相関........17
表 5.1.7. [FAS] 血中尿酸値........18
表 5.1.8. [FAS] バイタルサイン、一般血液・尿検査........19
表 5.1.9. [FAS] 心臓超音波検査パラメーター........34
表 5.1.10. [FAS] 特殊血液検査........37
表 5.1.11. [FAS] 特殊尿検査........41

5.2. PPS........50
表 5.2.1. [PPS] 24 週変化率以外も含めた NT-proBNP........50
表 5.2.2. [PPS] BNP........51
表 5.2.3. [PPS] NT-proBNP の変化率と BNP 変化率の相関........52
表 5.2.4. [PPS] FMD........53
表 5.2.5. [PPS] EndoPAT........54
表 5.2.6. [PPS] FMD の変化量と EndoPAT の変化量の相関........55
表 5.2.7. [PPS] 血中尿酸値........56
表 5.2.8. [PPS] バイタルサイン、一般血液・尿検査........57
表 5.2.9. [PPS] 心臓超音波検査パラメーター........72
表 5.2.10. [PPS] 特殊血液検査........75
表 5.2.11. [PPS] 特殊尿検査........79

6. 安全性評価項目........88

6.1. 有害事象 88
表 6.1.1. [安全性解析対象集団] 有害事象の発現件数........ 88
表 6.1.2. [安全性解析対象集団] 有害事象の発現例数と発現率(%)........ 90

6.2. 痛風関節炎(副作用) 92
6.2.1. FAS/安全性解析対象集団 92
表 6.2.1.1. [FAS/安全性解析対象集団] 痛風関節炎(副作用)の発現件数........ 92
表 6.2.1.2. [FAS/安全性解析対象集団] 痛風関節炎(副作用)の発現例数と発現率(%)........ 92
6.2.2. PPS 93
表 6.2.2.1. [PPS] 痛風関節炎(副作用)の発現件数........ 93
表 6.2.2.2. [PPS] 痛風関節炎(副作用)の発現例数と発現率(%)........ 93

6.3. 肝機能検査値異常 (副作用) 94
6.3.1. FAS/安全性解析対象集団 94
表 6.3.1.1. [FAS/安全性解析対象集団] 肝機能検査値異常(副作用)の発現件数........ 94
表 6.3.1.2. [FAS/安全性解析対象集団] 肝機能検査値異常(副作用)の発現例数と発現率(%) 94
6.3.2. PPS 95
表 6.3.2.1. [PPS] 肝機能検査値異常(副作用)の発現件数........ 95
表 6.3.2.2. [PPS] 肝機能検査値異常(副作用)の発現例数と発現率(%)........ 95

7. 部分集団解析 96

7.1. FAS 96
7.1.1. 部分集団 1 96
表 7.1.1.1. [FAS] 要約統計量と t 検定、Wilcoxon 検定........ 96
表 7.1.1.2. [FAS] 共分散分析........ 96
表 7.1.1.3. [FAS] MMRM........ 96
7.1.2. 部分集団 2 97
表 7.1.2.1. [FAS] 要約統計量と t 検定、Wilcoxon 検定........ 97
表 7.1.2.2. [FAS] 共分散分析........ 97
表 7.1.2.3. [FAS] MMRM........ 97
7.1.3. 部分集団 3 98
表 7.1.3.1. [FAS] 要約統計量と t 検定、Wilcoxon 検定........ 98
表 7.1.3.2. [FAS] 共分散分析........ 98
表 7.1.3.3. [FAS] MMRM........ 98

7.2. PPS 99
7.2.1. 部分集団 1 99
表 7.2.1.1. [PPS] 要約統計量と t 検定、Wilcoxon 検定........ 99
表 7.2.1.2. [PPS] 共分散分析........ 99
表 7.2.1.3. [PPS] MMRM........ 99
7.2.2. 部分集団 2 100
表 7.2.2.1. [PPS] 要約統計量と t 検定、Wilcoxon 検定........ 100
表 7.2.2.2. [PPS] 共分散分析........ 100
表 7.2.2.3. [PPS] MMRM........ 100
7.2.3. 部分集団 3 101
表 7.2.3.1. [PPS] 要約統計量と t 検定、Wilcoxon 検定........ 101
表 7.2.3.2. [PPS] 共分散分析........ 101
表 7.2.3.3. [PPS] MMRM........ 101

8. 薬剤情報 102
表 8.1. [FAS] 研究対象薬の服薬の有無........ 102
表 8.2. [FAS] 研究対象薬トピロキソスタットの服薬用量........ 102
表 8.3. [FAS] 研究対象薬アロプリノールの服薬用量........ 103

表 8.4. [FAS] 併用薬 ベースライン時 104
表 8.5. [FAS] 併用薬 12 週時 107
表 8.6. [FAS] 併用薬 24 週時 110

1. 研究のフローチャート

研究のフローチャートを図 1.1 に示す。

図 1.1. 研究のフローチャート
カッコ内の数字は該当症例数を示す。

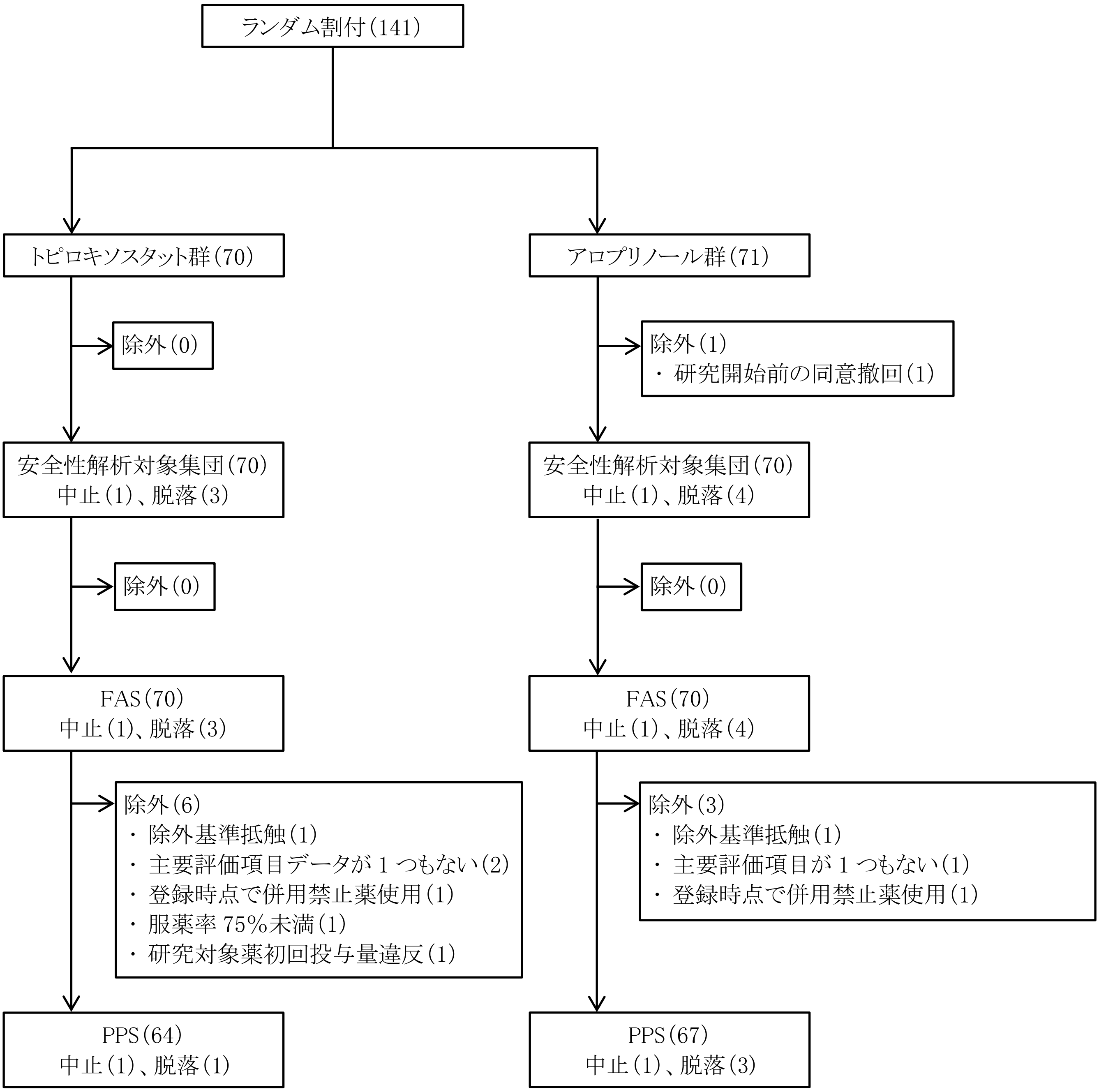

2. 研究対象者の内訳

研究対象者の内訳を表 2.1 に示す。

表 2.1. 研究対象者の内訳

対象	症例数	
	トピロキソスタット群	アロプリノール群
本研究に登録された症例	70	71
安全性解析集団の症例	70	70
FAS 集団の症例	70	70
PPS 集団の症例	64	67
同意を撤回した症例	0	1
ランダム化後に 1 回も研究対象薬を投薬されていない症例	0	1
主要評価項目のデータ（NT-proBNP 変化率）を少なくとも 1 つを有する症例	68	69
選択基準に合致しない症例	0	0
除外基準に抵触する症例	2	2

※ 除外基準に抵触する症例には、0 週から併用禁止薬を使用していた 2 症例を含む

3. 研究対象者背景

表 3.1. [FAS] 研究対象者背景(連続変数)

変数	統計量	トピロキソスタット群	アロプリノール群	群間比較 P 値
年齢	n	70	70	
	Mean±SD	71.7±9.7	69.9±9.1	0.26
	Median [Q1, Q3]	75.0 [66.0, 79.0]	70.5 [65.0, 77.0]	0.13
	Min, Max	39.0, 84.0	38.0, 85.0	

表 3.2. [FAS] 研究対象者背景(カテゴリ変数)

変数	水準	症例数(%)		群間比較 P 値	
		トピロキソスタット群 n = 70	アロプリノール群 n = 70	χ 二乗検定	Fisher 検定
性別	男性	57 (81.4)	56 (80.0)	0.83	1.00
	女性	13 (18.6)	14 (20.0)		
NYHA 分類	I 度	45 (64.3)	49 (70.0)	0.47	0.59
	II 度	25 (35.7)	21 (30.0)		
	III 度	0 (0.0)	0 (0.0)		
	IV 度	0 (0.0)	0 (0.0)		
心不全の原疾患 / 虚血性心疾患	あり	29 (41.4)	32 (45.7)	0.61	0.73
心不全の原疾患 / 心筋症	あり	18 (25.7)	13 (18.6)	0.31	0.42
心不全の原疾患 / 弁膜症	あり	12 (17.1)	12 (17.1)	1.00	1.00
心不全の原疾患 / 高血圧性心不全	あり	8 (11.4)	9 (12.9)	0.80	1.00
心不全の原疾患 / その他の心不全	あり	15 (21.4)	18 (25.7)	0.55	0.69
治療歴	あり	28 (40.0)	36 (51.4)	0.17	0.23
治療歴 / CABG 歴	あり	9 (12.9)	7 (10.0)	0.60	0.79
治療歴 / 弁置換術あるいは弁形成術	あり	5 (7.1)	9 (12.9)	0.26	0.40
治療歴 / PCI 歴	あり	20 (28.6)	23 (32.9)	0.58	0.71
治療歴 / PCI 歴 / POBA	あり	5 (7.1)	5 (7.1)	1.00	1.00
治療歴 / PCI 歴 / DES	あり	9 (12.9)	12 (17.1)	0.48	0.64
治療歴 / PCI 歴 / BMS	あり	9 (12.9)	10 (14.3)	0.81	1.00
治療歴 / PCI 歴 / DEB	あり	0 (0.0)	0 (0.0)	-	-
治療歴 / PCI 歴 / BVS	あり	0 (0.0)	0 (0.0)	-	-
治療歴 / PCI 歴 / その他	あり	1 (1.4)	0 (0.0)	0.32*	1.00
既往歴	あり	26 (37.1)	27 (38.6)	0.86	1.00
合併症	あり	67 (95.7)	67 (95.7)	1.00*	1.00
合併症 / 心房細動	あり	30 (42.9)	28 (40.0)	0.73	0.86
合併症 / その他不整脈	あり	13 (18.6)	13 (18.6)	1.00	1.00
合併症 / 細小血管合併症	あり	1 (1.4)	1 (1.4)	1.00*	1.00
合併症 / 腎疾患	あり	19 (27.1)	13 (18.6)	0.23	0.31
合併症 / 肝疾患	あり	3 (4.3)	0 (0.0)	0.08*	0.24
合併症 / 脳心血管系疾患	あり	6 (8.6)	2 (2.9)	0.15*	0.27
合併症 / 糖尿病	あり	26 (37.1)	21 (30.0)	0.37	0.47

合併症 / 高血圧	あり	42 (60.0)	55 (78.6)	0.017	0.027
合併症 / 脂質異常症	あり	41 (58.6)	41 (58.6)	1.00	1.00
合併症 / 痛風発作	あり	1 (1.4)	2 (2.9)	0.56*	1.00
合併症 / その他	あり	32 (45.7)	27 (38.6)	0.39	0.49

χ二乗検定のＰ値において、アスタリスクがついているものは、χ二乗検定の実施要件を満たしていないため、結果に信頼性がないことを示します。

表 3.3. [FAS] 心不全の原疾患 / その他の心不全 の詳細

変数	症例数(%)		群間比較 P 値	
	トピロキソスタット群 n = 70	アロプリノール群 n = 70	χ 二乗検定	Fisher 検定
心不全の原疾患 / その他の心不全 / I 度房室ブロック	1 (1.4)	0 (0.0)	0.32*	1.00
心不全の原疾患 / その他の心不全 / 拡張障害	1 (1.4)	0 (0.0)	0.32*	1.00
心不全の原疾患 / その他の心不全 / 収縮性心膜炎	1 (1.4)	1 (1.4)	1.00*	1.00
心不全の原疾患 / その他の心不全 / 徐脈	1 (1.4)	0 (0.0)	0.32*	1.00
心不全の原疾患 / その他の心不全 / 上室性期外収縮	0 (0.0)	1 (1.4)	0.32*	1.00
心不全の原疾患 / その他の心不全 / 心室期外収縮	0 (0.0)	3 (4.3)	0.08*	0.24
心不全の原疾患 / その他の心不全 / 心不全	1 (1.4)	0 (0.0)	0.32*	1.00
心不全の原疾患 / その他の心不全 / 心房細動	11 (15.7)	12 (17.1)	0.82	1.00
心不全の原疾患 / その他の心不全 / 心房粗動	0 (0.0)	1 (1.4)	0.32*	1.00
心不全の原疾患 / その他の心不全 / 肺血栓症	1 (1.4)	0 (0.0)	0.32*	1.00

χ 二乗検定の P 値において、アスタリスクがついているものは、χ 二乗検定の実施要件を満たしていないため、結果に信頼性がないことを示します。

表 3.4. [FAS] 既往歴の詳細

変数	症例数(%)		群間比較 P 値	
	トピロキソスタット群<br>n = 70	アロプリノール群<br>n = 70	χ 二乗<br>検定	Fisher<br>検定
既往歴 / ASD	1 (1.4)	0 (0.0)	0.32*	1.00
既往歴 / C 型肝炎	1 (1.4)	2 (2.9)	0.56*	1.00
既往歴 / S 状結腸癌	1 (1.4)	0 (0.0)	0.32*	1.00
既往歴 / アレルギー性鼻炎	0 (0.0)	1 (1.4)	0.32*	1.00
既往歴 / うつ病	1 (1.4)	0 (0.0)	0.32*	1.00
既往歴 / ダンピング症候群	1 (1.4)	0 (0.0)	0.32*	1.00
既往歴 / 胃癌	1 (1.4)	0 (0.0)	0.32*	1.00
既往歴 / 胃潰瘍	2 (2.9)	0 (0.0)	0.15*	0.50
既往歴 / 一過性脳虚血発作	1 (1.4)	0 (0.0)	0.32*	1.00
既往歴 / 肝機能障害	1 (1.4)	0 (0.0)	0.32*	1.00
既往歴 / 気管支喘息	1 (1.4)	2 (2.9)	0.56*	1.00
既往歴 / 逆流性食道炎	1 (1.4)	0 (0.0)	0.32*	1.00
既往歴 / 狭心症	1 (1.4)	0 (0.0)	0.32*	1.00
既往歴 / 胸部大動脈瘤	0 (0.0)	1 (1.4)	0.32*	1.00
既往歴 / 頚椎症性脊髄症	1 (1.4)	0 (0.0)	0.32*	1.00
既往歴 / 甲状腺機能低下症	0 (0.0)	1 (1.4)	0.32*	1.00
既往歴 / 甲状腺腫	0 (0.0)	1 (1.4)	0.32*	1.00
既往歴 / 高血圧	0 (0.0)	1 (1.4)	0.32*	1.00
既往歴 / 腰椎ヘルニア	0 (0.0)	1 (1.4)	0.32*	1.00
既往歴 / 腰部脊柱管狭窄症	1 (1.4)	0 (0.0)	0.32*	1.00
既往歴 / 左室心尖部壁在血栓	1 (1.4)	0 (0.0)	0.32*	1.00
既往歴 / 十二指腸潰瘍	1 (1.4)	0 (0.0)	0.32*	1.00
既往歴 / 縦隔腫瘍	0 (0.0)	1 (1.4)	0.32*	1.00
既往歴 / 徐脈性不整脈	0 (0.0)	1 (1.4)	0.32*	1.00
既往歴 / 消化管出血	1 (1.4)	0 (0.0)	0.32*	1.00
既往歴 / 硝子体出血	0 (0.0)	1 (1.4)	0.32*	1.00
既往歴 / 心原性脳塞栓症	1 (1.4)	0 (0.0)	0.32*	1.00
既往歴 / 心室細動・心室頻拍	0 (0.0)	1 (1.4)	0.32*	1.00
既往歴 / 心室性期外収縮	0 (0.0)	1 (1.4)	0.32*	1.00
既往歴 / 心臓粘液腫	1 (1.4)	0 (0.0)	0.32*	1.00
既往歴 / 心内血栓	1 (1.4)	0 (0.0)	0.32*	1.00
既往歴 / 心不全	1 (1.4)	0 (0.0)	0.32*	1.00
既往歴 / 心房細動	1 (1.4)	0 (0.0)	0.32*	1.00
既往歴 / 心房粗動	1 (1.4)	0 (0.0)	0.32*	1.00
既往歴 / 深部静脈血栓症	0 (0.0)	1 (1.4)	0.32*	1.00
既往歴 / 腎結石	1 (1.4)	0 (0.0)	0.32*	1.00
既往歴 / 水晶体再建術	1 (1.4)	0 (0.0)	0.32*	1.00
既往歴 / 水腎症	1 (1.4)	0 (0.0)	0.32*	1.00
既往歴 / 睡眠時無呼吸症候群	1 (1.4)	0 (0.0)	0.32*	1.00
既往歴 / 前立腺癌	1 (1.4)	1 (1.4)	1.00*	1.00
既往歴 / 前立腺肥大症	1 (1.4)	0 (0.0)	0.32*	1.00
既往歴 / 鼠径ヘルニア	1 (1.4)	0 (0.0)	0.32*	1.00
既往歴 / 僧帽弁閉鎖不全症	0 (0.0)	1 (1.4)	0.32*	1.00

既往歴の詳細

既往歴 / 大腿骨頚部骨折	1 (1.4)	0 (0.0)	0.32*	1.00
既往歴 / 大腸ポリープ	1 (1.4)	0 (0.0)	0.32*	1.00
既往歴 / 大腸癌	1 (1.4)	0 (0.0)	0.32*	1.00
既往歴 / 大動脈弁閉鎖不全症	0 (0.0)	2 (2.9)	0.15*	0.50
既往歴 / 大動脈弁輪拡張症	0 (0.0)	1 (1.4)	0.32*	1.00
既往歴 / 胆のう炎	1 (1.4)	0 (0.0)	0.32*	1.00
既往歴 / 胆石	0 (0.0)	1 (1.4)	0.32*	1.00
既往歴 / 蓄膿症	0 (0.0)	1 (1.4)	0.32*	1.00
既往歴 / 中咽頭腫瘍	0 (0.0)	1 (1.4)	0.32*	1.00
既往歴 / 虫垂炎	2 (2.9)	0 (0.0)	0.15*	0.50
既往歴 / 直腸癌	0 (0.0)	1 (1.4)	0.32*	1.00
既往歴 / 陳旧性心筋梗塞	2 (2.9)	2 (2.9)	1.00*	1.00
既往歴 / 糖尿病	0 (0.0)	1 (1.4)	0.32*	1.00
既往歴 / 頭部外傷・脳挫傷	1 (1.4)	0 (0.0)	0.32*	1.00
既往歴 / 洞不全症候群	0 (0.0)	1 (1.4)	0.32*	1.00
既往歴 / 乳癌	1 (1.4)	0 (0.0)	0.32*	1.00
既往歴 / 尿管結石	2 (2.9)	0 (0.0)	0.15*	0.50
既往歴 / 脳梗塞	4 (5.7)	7 (10.0)	0.35	0.53
既往歴 / 脳出血	0 (0.0)	1 (1.4)	0.32*	1.00
既往歴 / 脳動脈瘤	1 (1.4)	0 (0.0)	0.32*	1.00
既往歴 / 肺結核	0 (0.0)	1 (1.4)	0.32*	1.00
既往歴 / 頻脈	1 (1.4)	0 (0.0)	0.32*	1.00
既往歴 / 変形性股関節症	0 (0.0)	1 (1.4)	0.32*	1.00
既往歴 / 変形性腰椎症	0 (0.0)	1 (1.4)	0.32*	1.00
既往歴 / 変形性膝関節症	1 (1.4)	0 (0.0)	0.32*	1.00
既往歴 / 慢性硬膜下血腫	2 (2.9)	0 (0.0)	0.15*	0.50
既往歴 / 膵炎	0 (0.0)	1 (1.4)	0.32*	1.00
既往歴 / 蕁麻疹	1 (1.4)	0 (0.0)	0.32*	1.00

χ二乗検定のＰ値において、アスタリスクがついているものは、χ二乗検定の実施要件を満たしていないため、結果に信頼性がないことを示します。

表 3.5. [FAS] 各合併症の詳細

変数	症例数(%)		群間比較 P 値	
	トピロキソスタット群 n = 70	アロプリノール群 n = 70	χ 二乗検定	Fisher 検定
合併症 / その他不整脈 / WPW 症候群	1 (1.4)	0 (0.0)	0.32*	1.00
合併症 / その他不整脈 / 完全房室ブロック	1 (1.4)	0 (0.0)	0.32*	1.00
合併症 / その他不整脈 / 徐脈	1 (1.4)	0 (0.0)	0.32*	1.00
合併症 / その他不整脈 / 上室性期外収縮	1 (1.4)	0 (0.0)	0.32*	1.00
合併症 / その他不整脈 / 心室期外収縮	5 (7.1)	9 (12.9)	0.26	0.40
合併症 / その他不整脈 / 心室頻拍	3 (4.3)	3 (4.3)	1.00*	1.00
合併症 / その他不整脈 / 心房粗動	1 (1.4)	0 (0.0)	0.32*	1.00
合併症 / その他不整脈 / 洞性徐脈	0 (0.0)	1 (1.4)	0.32*	1.00
合併症 / その他不整脈 / 洞不全症候群	1 (1.4)	0 (0.0)	0.32*	1.00
合併症 / 細小血管合併症 / 糖尿病性網膜症	1 (1.4)	1 (1.4)	1.00*	1.00
合併症 / 腎疾患 / CKD	12 (17.1)	13 (18.6)	0.83	1.00
合併症 / 腎疾患 / 高血圧性腎疾患	1 (1.4)	0 (0.0)	0.32*	1.00
合併症 / 腎疾患 / 糸球体腎炎	0 (0.0)	1 (1.4)	0.32*	1.00
合併症 / 腎疾患 / 腎梗塞	1 (1.4)	0 (0.0)	0.32*	1.00
合併症 / 腎疾患 / 慢性腎不全	5 (7.1)	0 (0.0)	0.023*	0.06
合併症 / 肝疾患 / B 型肝炎	1 (1.4)	0 (0.0)	0.32*	1.00
合併症 / 肝疾患 / アルコール性肝障害	1 (1.4)	0 (0.0)	0.32*	1.00
合併症 / 肝疾患 / 肝機能障害	1 (1.4)	0 (0.0)	0.32*	1.00
合併症 / 脳心血管系疾患 / 狭心症	2 (2.9)	0 (0.0)	0.15*	0.50
合併症 / 脳心血管系疾患 / 心原性脳塞栓症	1 (1.4)	0 (0.0)	0.32*	1.00
合併症 / 脳心血管系疾患 / 大動脈弁閉鎖不全症	1 (1.4)	0 (0.0)	0.32*	1.00
合併症 / 脳心血管系疾患 / 陳旧性心筋梗塞	0 (0.0)	1 (1.4)	0.32*	1.00
合併症 / 脳心血管系疾患 / 内頚動脈狭窄症	1 (1.4)	0 (0.0)	0.32*	1.00
合併症 / 脳心血管系疾患 / 脳梗塞後遺症	0 (0.0)	1 (1.4)	0.32*	1.00
合併症 / 脳心血管系疾患 / 未破裂脳動脈瘤	1 (1.4)	0 (0.0)	0.32*	1.00
合併症 / その他 / COPD	1 (1.4)	3 (4.3)	0.31*	0.62
合併症 / その他 / leriche 症候群	0 (0.0)	1 (1.4)	0.32*	1.00
合併症 / その他 / アレルギー性皮膚炎	1 (1.4)	0 (0.0)	0.32*	1.00
合併症 / その他 / アレルギー性鼻炎	1 (1.4)	0 (0.0)	0.32*	1.00
合併症 / その他 / てんかん	4 (5.7)	0 (0.0)	0.042*	0.12
合併症 / その他 / レム睡眠行動異常症	1 (1.4)	0 (0.0)	0.32*	1.00
合併症 / その他 / 胃潰瘍	1 (1.4)	0 (0.0)	0.32*	1.00
合併症 / その他 / 下肢静脈瘤	1 (1.4)	0 (0.0)	0.32*	1.00
合併症 / その他 / 下肢閉塞性動脈硬化症	1 (1.4)	3 (4.3)	0.31*	0.62
合併症 / その他 / 加齢黄斑変性症	0 (0.0)	1 (1.4)	0.32*	1.00
合併症 / その他 / 感音難聴	0 (0.0)	1 (1.4)	0.32*	1.00
合併症 / その他 / 感冒	1 (1.4)	0 (0.0)	0.32*	1.00
合併症 / その他 / 間質性肺炎	0 (0.0)	1 (1.4)	0.32*	1.00
合併症 / その他 / 気管支喘息	1 (1.4)	1 (1.4)	1.00*	1.00
合併症 / その他 / 逆流性食道炎	3 (4.3)	2 (2.9)	0.65*	1.00
合併症 / その他 / 橋本病	0 (0.0)	1 (1.4)	0.32*	1.00
合併症 / その他 / 肩関節症	0 (0.0)	1 (1.4)	0.32*	1.00
合併症 / その他 / 甲状腺機能低下症	1 (1.4)	1 (1.4)	1.00*	1.00

合併症 / その他 / 甲状腺機能亢進症	2 (2.9)	1 (1.4)	0.56*	1.00
合併症 / その他 / 高カリウム血症	0 (0.0)	2 (2.9)	0.15*	0.50
合併症 / その他 / 高位正中神経麻痺	0 (0.0)	1 (1.4)	0.32*	1.00
合併症 / その他 / 腰痛症	2 (2.9)	1 (1.4)	0.56*	1.00
合併症 / その他 / 心身症	1 (1.4)	1 (1.4)	1.00*	1.00
合併症 / その他 / 神経症	3 (4.3)	0 (0.0)	0.08*	0.24
合併症 / その他 / 睡眠時無呼吸症候群	3 (4.3)	1 (1.4)	0.31*	0.62
合併症 / その他 / 前立腺肥大	3 (4.3)	3 (4.3)	1.00*	1.00
合併症 / その他 / 双極性障害	1 (1.4)	0 (0.0)	0.32*	1.00
合併症 / その他 / 打ち身	1 (1.4)	0 (0.0)	0.32*	1.00
合併症 / その他 / 胆石症	1 (1.4)	0 (0.0)	0.32*	1.00
合併症 / その他 / 鉄欠乏性貧血	0 (0.0)	3 (4.3)	0.08*	0.24
合併症 / その他 / 特発性レストレスレッグス症候群	1 (1.4)	0 (0.0)	0.32*	1.00
合併症 / その他 / 認知症	0 (0.0)	1 (1.4)	0.32*	1.00
合併症 / その他 / 白内障	1 (1.4)	1 (1.4)	1.00*	1.00
合併症 / その他 / 不眠症	2 (2.9)	1 (1.4)	0.56*	1.00
合併症 / その他 / 変形性膝関節症	0 (0.0)	1 (1.4)	0.32*	1.00
合併症 / その他 / 便秘症	2 (2.9)	2 (2.9)	1.00*	1.00
合併症 / その他 / 弁膜症	0 (0.0)	1 (1.4)	0.32*	1.00
合併症 / その他 / 末梢性めまい	1 (1.4)	0 (0.0)	0.32*	1.00
合併症 / その他 / 慢性萎縮性胃炎	1 (1.4)	0 (0.0)	0.32*	1.00
合併症 / その他 / 慢性気管支炎	1 (1.4)	0 (0.0)	0.32*	1.00
合併症 / その他 / 慢性閉塞性肺疾患	2 (2.9)	0 (0.0)	0.15*	0.50
合併症 / その他 / 網膜格子状変性	0 (0.0)	1 (1.4)	0.32*	1.00
合併症 / その他 / 緑内障	1 (1.4)	0 (0.0)	0.32*	1.00

χ二乗検定のP値において、アスタリスクがついているものは、χ二乗検定の実施要件を満たしていないため、結果に信頼性がないことを示します。

4. 主要評価項目

4.1. FAS

表 4.1.1. [FAS] 要約統計量と t 検定、Wilcoxon 検定

変数		観察ポイント	統計量	トピロキソスタット群	アロプリノール群	群間比較 P 値
対数変換 NT-proBNP (ln(pg/mL))	測定値	24 週	n	66	66	
			Mean±SD	6.5±0.9	6.3±1.0	0.16
			Median [Q1, Q3]	6.4 [5.9, 7.2]	6.2 [5.5, 7.0]	0.14
			Min, Max	4.7, 8.9	4.2, 9.4	
	変化率	24 週	n	66	66	
			Mean±SD	1.6±8.2	-0.4±8.0	0.17
			Median [Q1, Q3]	0.4 [-3.1, 4.6]	-0.4 [-4.3, 5.0]	0.42
			Min, Max	-15.5, 30.5	-27.6, 16.9	
			One-sample t-test	0.13	0.69	
			Wilcoxon signed-rank test	0.29	0.92	

表 4.1.2. [FAS] 共分散分析

変数		観察ポイント	n	調整済み平均値(SE)		調整済み平均値の差(95%CI)	群間比較 P 値
				トピロキソスタット群	アロプリノール群		
対数変換 NT-proBNP (ln(pg/mL))	変化率	24 週	132	0.6 (1.1)	-1.2 (1.1)	1.8 (-1.0, 4.6)	0.20

群を固定効果、割付調整因子（BNP 200 pg/ml 未満／200 pg/ml 以上、心臓超音波検査 EF 45%未満／45%以上）を共変量とした共分散分析を実施。

表 4.1.3. [FAS] MMRM

変数		観察ポイント	n	調整済み平均値(SE)		調整済み平均値の差(95%CI)	群間比較 P 値
				トピロキソスタット群	アロプリノール群		
対数変換 NT-proBNP (ln(pg/mL))	変化率	24 週	137	1.1 (1.1)	-0.5 (1.1)	1.6 (-1.2, 4.5)	0.25

群および時期効果、群と時期の交互作用、割付調整因子（BNP 200 pg/ml 未満／200 pg/ml 以上、心臓超音波検査 EF 45%未満／45%以上）を固定効果、研究対象者を変量効果としたモデルにて MMRM を実施。

4.2. PPS

表 4.2.1. [PPS] 要約統計量と t 検定、Wilcoxon 検定

変数		観察ポイント	統計量	トピロキソスタット群	アロプリノール群	群間比較 P 値
対数変換 NT-proBNP (ln(pg/mL))	測定値	24 週	n	61	63	
			Mean±SD	6.5±0.9	6.3±1.0	0.33
			Median [Q1, Q3]	6.4 [5.9, 7.1]	6.2 [5.5, 7.0]	0.24
			Min, Max	4.7, 8.4	4.6, 9.4	
	変化率	24 週	n	61	63	
			Mean±SD	1.3±8.1	0.1±7.6	0.39
			Median [Q1, Q3]	0.3 [-2.8, 4.1]	0.5 [-4.0, 5.0]	0.71
			Min, Max	-15.5, 30.5	-27.6, 16.9	
			One-sample t-test	0.21	0.93	
			Wilcoxon signed-rank test	0.37	0.62	

表 4.2.2. [PPS] 共分散分析

変数		観察ポイント	n	調整済み平均値(SE)		調整済み平均値の差(95%CI)	群間比較 P 値
				トピロキソスタット群	アロプリノール群		
対数変換 NT-proBNP (ln(pg/mL))	変化率	24 週	124	0.4 (1.2)	-0.8 (1.1)	1.1 (-1.6, 3.9)	0.42

群を固定効果、割付調整因子(BNP 200 pg/ml 未満/200 pg/ml 以上、心臓超音波検査 EF 45%未満/45%以上)を共変量とした共分散分析を実施。

表 4.2.3. [PPS] MMRM

変数		観察ポイント	n	調整済み平均値(SE)		調整済み平均値の差(95%CI)	群間比較 P 値
				トピロキソスタット群	アロプリノール群		
対数変換 NT-proBNP (ln(pg/mL))	変化率	24 週	129	0.9 (1.1)	-0.2 (1.1)	1.2 (-1.7, 4.0)	0.42

群および時期効果、群と時期の交互作用、割付調整因子(BNP 200 pg/ml 未満/200 pg/ml 以上、心臓超音波検査 EF 45%未満/45%以上)を固定効果、研究対象者を変量効果としたモデルにて MMRM を実施。

5. 副次評価項目

副次評価項目のうち、以下の特殊血液検査項目はデータが収集されなかったため解析結果に含まない。

- 炎症バイオマーカー（IL-1β、IL6、IL8、IL-12、IFN-γ、SDF1α、TNFα、MCP-1）、FACS、EPC（CD34+/CD133+/CD45low+）、EPC（CD34+/KDR）、d-ROMs、BAP、トロポニンI

5.1. FAS

表 5.1.1. [FAS] 24 週変化率以外も含めた NT-proBNP

変数		観察ポイント	統計量	トピロキソスタット群	アロプリノール群	群間比較 P値
対数変換 NT-proBNP (ln(pg/mL))	測定値	0 週	n	70	70	
			Mean±SD	6.56±1.02	6.36±0.90	0.23
			Median [Q1, Q3]	6.46 [5.78, 7.22]	6.34 [5.75, 7.06]	0.33
			Min, Max	4.68, 10.22	4.58, 8.77	
		12 週	n	65	68	
			Mean±SD	6.53±0.94	6.32±1.04	0.23
			Median [Q1, Q3]	6.43 [5.84, 7.27]	6.45 [5.53, 7.02]	0.36
			Min, Max	4.62, 9.17	4.35, 9.53	
		24 週	n	66	66	
			Mean±SD	6.53±0.90	6.29±1.03	0.16
			Median [Q1, Q3]	6.36 [5.92, 7.20]	6.20 [5.52, 7.01]	0.14
			Min, Max	4.70, 8.86	4.23, 9.36	
	変化量	12 週	n	65	68	
			Mean±SD	0.05±0.44	-0.01±0.45	0.50
			Median [Q1, Q3]	-0.03 [-0.20, 0.34]	-0.02 [-0.30, 0.30]	0.41
			Min, Max	-1.05, 1.31	-1.10, 1.91	
			One-sample t-test	0.41	0.89	
			Wilcoxon signed-rank test	0.48	0.69	
		24 週	n	66	66	
			Mean±SD	0.08±0.48	-0.03±0.51	0.22
			Median [Q1, Q3]	0.02 [-0.23, 0.27]	-0.03 [-0.24, 0.28]	0.47
			Min, Max	-0.87, 1.53	-1.77, 1.06	
			One-sample t-test	0.18	0.66	
			Wilcoxon signed-rank test	0.32	0.98	
	変化率	12 週	n	65	68	
			Mean±SD	1.0±7.0	-0.3±7.1	0.28
			Median [Q1, Q3]	-0.4 [-3.5, 5.8]	-0.3 [-4.5, 4.8]	0.36
			Min, Max	-14.3, 24.2	-17.3, 25.2	
			One-sample t-test	0.24	0.74	
			Wilcoxon signed-rank test	0.42	0.70	
		24 週	n	66	66	
			Mean±SD	1.6±8.2	-0.4±8.0	0.17
			Median [Q1, Q3]	0.4 [-3.1, 4.6]	-0.4 [-4.3, 5.0]	0.42
			Min, Max	-15.5, 30.5	-27.6, 16.9	
			One-sample t-test	0.13	0.69	
			Wilcoxon signed-rank test	0.29	0.92	

表 5.1.2. [FAS] BNP

変数		観察ポイント	統計量	トピロキソスタット群	アロプリノール群	群間比較 P 値
対数変換 BNP (ln(pg/mL))	測定値	0 週	n	69	69	
			Mean±SD	4.99±0.79	4.83±0.67	0.21
			Median [Q1, Q3]	4.85 [4.44, 5.53]	4.84 [4.39, 5.25]	0.38
			Min, Max	3.33, 7.47	3.18, 6.52	
		12 週	n	68	70	
			Mean±SD	5.01±0.76	4.84±0.87	0.24
			Median [Q1, Q3]	4.87 [4.53, 5.36]	4.95 [4.28, 5.35]	0.43
			Min, Max	3.03, 7.22	2.79, 7.25	
		24 週	n	66	67	
			Mean±SD	4.97±0.76	4.82±0.81	0.27
			Median [Q1, Q3]	4.95 [4.57, 5.40]	4.87 [4.24, 5.39]	0.37
			Min, Max	2.65, 7.15	3.10, 6.98	
	変化量	12 週	n	67	69	
			Mean±SD	0.02±0.45	0.01±0.52	0.84
			Median [Q1, Q3]	0.06 [−0.22, 0.27]	0.00 [−0.32, 0.31]	0.51
			Min, Max	−1.29, 1.13	−1.04, 1.72	
			One-sample t-test	0.65	0.91	
			Wilcoxon signed-rank test	0.52	0.83	
		24 週	n	65	66	
			Mean±SD	0.06±0.55	−0.02±0.53	0.43
			Median [Q1, Q3]	0.07 [−0.26, 0.31]	0.04 [−0.20, 0.27]	0.74
			Min, Max	−1.68, 1.57	−1.87, 1.33	
			One-sample t-test	0.40	0.80	
			Wilcoxon signed-rank test	0.36	0.65	
	変化率	12 週	n	67	69	
			Mean±SD	1.1±9.7	0.0±10.7	0.55
			Median [Q1, Q3]	1.0 [−4.1, 5.8]	0.1 [−6.4, 6.2]	0.46
			Min, Max	−29.9, 32.2	−22.5, 33.9	
			One-sample t-test	0.36	0.98	
			Wilcoxon signed-rank test	0.39	0.83	
		24 週	n	65	66	
			Mean±SD	1.7±12.3	−0.2±10.8	0.34
			Median [Q1, Q3]	1.4 [−5.0, 6.8]	0.7 [−4.9, 5.2]	0.65
			Min, Max	−38.8, 40.3	−36.1, 23.5	
			One-sample t-test	0.26	0.88	
			Wilcoxon signed-rank test	0.32	0.68	

NT-proBNP の変化率と BNP 変化率の相関

表 5.1.3. [FAS] NT-proBNP の変化率と BNP 変化率の相関

変数 1	変数 2	n	Pearson		Spearman	
			相関係数 (95%CI)	P 値	相関係数 (95%CI)	P 値
対数変換 NT-proBNP (ln(pg/mL)) 12 週変化率	対数変換 BNP (ln(pg/mL)) 12 週変化率	130	0.75 (0.66, 0.81)	<0.001	0.69 (0.59, 0.77)	<0.001
対数変換 NT-proBNP (ln(pg/mL)) 24 週変化率	対数変換 BNP (ln(pg/mL)) 24 週変化率	130	0.84 (0.77, 0.88)	<0.001	0.72 (0.63, 0.79)	<0.001

表 5.1.4. [FAS] FMD

変数		観察ポイント	統計量	トピロキソスタット群	アロプリノール群	群間比較P値
FMD(%)	測定値	0 週	n	49	50	
			Mean±SD	4.77±2.37	4.49±2.14	0.53
			Median [Q1, Q3]	4.60 [3.00, 5.80]	4.50 [2.90, 5.50]	0.74
			Min, Max	1.10, 11.50	1.10, 10.80	
		24 週	n	48	48	
			Mean±SD	4.89±2.41	4.22±1.58	0.11
			Median [Q1, Q3]	4.15 [3.45, 6.10]	4.00 [3.20, 5.20]	0.28
			Min, Max	1.30, 12.60	1.20, 9.20	
	変化量	24 週	n	47	47	
			Mean±SD	0.04±1.81	−0.27±1.39	0.35
			Median [Q1, Q3]	0.30 [−0.80, 1.10]	−0.20 [−0.90, 0.50]	0.17
			Min, Max	−6.60, 4.40	−5.60, 2.40	
			One-sample t-test	0.87	0.19	
			Wilcoxon signed-rank test	0.47	0.21	

表 5.1.5. [FAS] EndoPAT

変数		観察ポイント	統計量	トピロキソスタット群	アロプリノール群	群間比較P値
EndoPAT（RHI指数）	測定値	0 週	n	68	68	
			Mean±SD	1.76±0.56	1.80±0.65	0.69
			Median [Q1, Q3]	1.64 [1.39, 2.03]	1.67 [1.44, 1.96]	0.73
			Min, Max	0.92, 3.66	0.75, 4.56	
		24 週	n	65	63	
			Mean±SD	1.74±0.50	1.79±0.57	0.58
			Median [Q1, Q3]	1.62 [1.39, 1.93]	1.70 [1.39, 2.02]	0.65
			Min, Max	0.78, 2.87	0.86, 3.52	
	変化量	24 週	n	64	62	
			Mean±SD	−0.04±0.47	−0.01±0.66	0.78
			Median [Q1, Q3]	−0.02 [−0.25, 0.21]	−0.04 [−0.31, 0.31]	0.92
			Min, Max	−1.52, 0.98	−2.48, 1.66	
			One-sample t-test	0.50	0.89	
			Wilcoxon signed-rank test	0.55	0.90	

FMDの変化量とEndoPATの変化量の相関

表5.1.6. [FAS] FMDの変化量とEndoPATの変化量の相関

変数1	変数2	n	Pearson		Spearman	
			相関係数<br>(95%CI)	P値	相関係数<br>(95%CI)	P値
FMD（%） 24週変化量	EndoPAT（RHI指数）<br>24週変化量	94	0.09 (−0.11, 0.29)	0.37	0.12 (−0.09, 0.31)	0.27

5. 副次評価項目

5.1. FAS

血中尿酸値

表 5.1.7. [FAS] 血中尿酸値

変数		観察ポイント	統計量	トピロキソスタット群	アロプリノール群	群間比較P値
血中尿酸値(mg/dL)	測定値	0 週	n	69	69	
			Mean±SD	8.3±1.4	8.2±1.4	0.63
			Median [Q1, Q3]	8.0 [7.2, 9.1]	7.9 [7.3, 8.7]	0.71
			Min, Max	5.7, 11.9	5.4, 12.4	
		12 週	n	69	70	
			Mean±SD	5.4±1.1	5.9±1.1	0.033
			Median [Q1, Q3]	5.5 [4.9, 6.1]	5.9 [5.1, 6.4]	0.031
			Min, Max	3.2, 8.7	3.6, 9.0	
		24 週	n	66	67	
			Mean±SD	5.6±1.2	5.9±1.2	0.10
			Median [Q1, Q3]	5.5 [4.6, 6.6]	6.0 [5.1, 6.4]	0.18
			Min, Max	3.1, 7.8	3.1, 9.7	
	変化量	12 週	n	68	69	
			Mean±SD	−2.8±1.2	−2.3±1.3	0.023
			Median [Q1, Q3]	−2.6 [−3.7, −1.9]	−2.1 [−2.9, −1.5]	0.024
			Min, Max	−5.5, −0.9	−6.2, 0.7	
			One-sample t-test	<0.001	<0.001	
			Wilcoxon signed-rank test	<0.001	<0.001	
		24 週	n	65	66	
			Mean±SD	−2.6±1.5	−2.2±1.3	0.08
			Median [Q1, Q3]	−2.5 [−3.7, −1.8]	−2.1 [−3.1, −1.4]	0.05
			Min, Max	−6.6, 0.6	−5.7, 1.2	
			One-sample t-test	<0.001	<0.001	
			Wilcoxon signed-rank test	<0.001	<0.001	

表 5.1.8. [FAS] バイタルサイン、一般血液・尿検査

変数		観察ポイント	統計量	トピロキソスタット群	アロプリノール群	群間比較 P 値
体重(kg)	測定値	0 週	n	70	70	
			Mean±SD	67.0±13.7	66.3±12.7	0.74
			Median [Q1, Q3]	64.0 [56.0, 74.0]	66.0 [59.8, 74.0]	0.99
			Min, Max	44.5, 107.6	41.0, 104.0	
		12 週	n	62	65	
			Mean±SD	66.7±13.8	66.2±12.9	0.81
			Median [Q1, Q3]	64.3 [57.8, 75.0]	65.0 [59.7, 73.3]	0.94
			Min, Max	42.0, 111.0	44.0, 108.0	
		24 週	n	64	66	
			Mean±SD	67.2±14.1	65.7±14.1	0.53
			Median [Q1, Q3]	65.4 [57.9, 76.0]	64.0 [59.0, 74.0]	0.65
			Min, Max	42.0, 113.9	37.8, 110.0	
	変化量	12 週	n	62	65	
			Mean±SD	0.1±2.4	-0.4±4.4	0.44
			Median [Q1, Q3]	0.0 [-1.0, 1.0]	0.0 [-0.5, 1.0]	0.84
			Min, Max	-8.6, 8.0	-30.0, 6.9	
			One-sample t-test	0.80	0.45	
			Wilcoxon signed-rank test	0.91	0.66	
		24 週	n	64	66	
			Mean±SD	0.2±2.5	-0.7±5.1	0.20
			Median [Q1, Q3]	0.0 [-1.6, 1.4]	0.0 [-1.6, 1.2]	0.51
			Min, Max	-6.0, 8.0	-31.0, 11.6	
			One-sample t-test	0.49	0.27	
			Wilcoxon signed-rank test	0.68	0.64	
BMI(kg/m^2)	測定値	0 週	n	70	70	
			Mean±SD	25.1±4.2	25.0±3.6	0.84
			Median [Q1, Q3]	24.6 [21.6, 28.0]	24.6 [22.2, 27.0]	1.00
			Min, Max	17.1, 39.0	19.4, 38.2	
		12 週	n	62	65	
			Mean±SD	25.0±4.5	24.8±3.9	0.77
			Median [Q1, Q3]	24.3 [21.5, 27.9]	24.6 [22.1, 26.7]	0.90
			Min, Max	16.0, 40.3	19.0, 39.7	
		24 週	n	64	66	
			Mean±SD	25.2±4.6	24.7±4.2	0.51
			Median [Q1, Q3]	24.8 [21.8, 27.4]	24.4 [22.0, 26.7]	0.54
			Min, Max	16.0, 41.3	15.9, 40.4	
	変化量	12 週	n	62	65	
			Mean±SD	0.0±0.8	-0.2±1.6	0.41
			Median [Q1, Q3]	0.0 [-0.4, 0.4]	0.0 [-0.2, 0.4]	0.86
			Min, Max	-2.6, 2.9	-11.2, 2.4	
			One-sample t-test	0.69	0.46	
			Wilcoxon signed-rank test	0.87	0.72	
		24 週	n	64	66	
			Mean±SD	0.1±0.9	-0.3±1.9	0.16
			Median [Q1, Q3]	0.0 [-0.5, 0.6]	0.0 [-0.6, 0.6]	0.45
			Min, Max	-2.0, 2.9	-11.5, 4.1	

			One-sample t-test	0.38	0.25	
			Wilcoxon signed-rank test	0.55	0.63	
収縮期血圧 (mmHg)	測定値	0 週	n	70	70	
			Mean±SD	124.3±19.7	125.7±18.0	0.65
			Median [Q1, Q3]	120.5 [110.0, 137.0]	124.0 [113.0, 138.0]	0.60
			Min, Max	91.0, 171.0	90.0, 163.0	
		12 週	n	68	70	
			Mean±SD	123.2±18.2	125.1±17.0	0.52
			Median [Q1, Q3]	120.0 [110.0, 134.5]	123.5 [116.0, 140.0]	0.35
			Min, Max	96.0, 172.0	93.0, 158.0	
		24 週	n	66	67	
			Mean±SD	125.9±19.1	124.0±19.2	0.57
			Median [Q1, Q3]	123.0 [114.0, 136.0]	124.0 [113.0, 135.0]	0.71
			Min, Max	95.0, 179.0	84.0, 164.0	
	変化量	12 週	n	68	70	
			Mean±SD	-1.1±16.8	-0.6±16.0	0.86
			Median [Q1, Q3]	1.5 [-12.5, 10.0]	2.0 [-9.0, 7.0]	0.83
			Min, Max	-53.0, 37.0	-53.0, 32.0	
			One-sample t-test	0.59	0.76	
			Wilcoxon signed-rank test	0.82	0.96	
		24 週	n	66	67	
			Mean±SD	1.6±16.7	-0.8±18.2	0.43
			Median [Q1, Q3]	2.0 [-8.0, 11.0]	1.0 [-12.0, 9.0]	0.47
			Min, Max	-47.0, 53.0	-60.0, 40.0	
			One-sample t-test	0.44	0.71	
			Wilcoxon signed-rank test	0.28	0.93	
拡張期血圧 (mmHg)	測定値	0 週	n	70	70	
			Mean±SD	73.6±15.3	73.1±12.7	0.84
			Median [Q1, Q3]	73.0 [60.0, 83.0]	73.5 [66.0, 79.0]	0.78
			Min, Max	47.0, 116.0	42.0, 118.0	
		12 週	n	68	70	
			Mean±SD	71.9±16.3	72.9±13.6	0.69
			Median [Q1, Q3]	70.0 [60.0, 81.5]	74.0 [63.0, 82.0]	0.41
			Min, Max	46.0, 123.0	40.0, 108.0	
		24 週	n	66	67	
			Mean±SD	72.0±15.5	73.9±15.8	0.50
			Median [Q1, Q3]	71.0 [60.0, 83.0]	70.0 [64.0, 83.0]	0.51
			Min, Max	40.0, 122.0	44.0, 124.0	
	変化量	12 週	n	68	70	
			Mean±SD	-1.5±14.8	-0.2±12.4	0.59
			Median [Q1, Q3]	-2.0 [-11.0, 5.0]	0.0 [-8.0, 8.0]	0.32
			Min, Max	-45.0, 45.0	-34.0, 35.0	
			One-sample t-test	0.42	0.89	
			Wilcoxon signed-rank test	0.17	0.87	
		24 週	n	66	67	
			Mean±SD	-1.0±13.8	0.7±13.8	0.46
			Median [Q1, Q3]	-0.5 [-8.0, 6.0]	0.0 [-8.0, 8.0]	0.46
			Min, Max	-34.0, 52.0	-28.0, 34.0	
			One-sample t-test	0.54	0.67	

			Wilcoxon signed-rank test	0.33	0.83	
脈拍(bpm)	測定値	0 週	n	69	70	
			Mean±SD	72.3±14.0	72.0±13.4	0.91
			Median [Q1, Q3]	71.0 [64.0, 80.0]	72.0 [62.0, 83.0]	0.81
			Min, Max	44.0, 114.0	41.0, 98.0	
		12 週	n	64	69	
			Mean±SD	75.3±15.3	73.3±12.5	0.41
			Median [Q1, Q3]	74.5 [64.0, 84.5]	74.0 [63.0, 83.0]	0.61
			Min, Max	47.0, 134.0	47.0, 106.0	
		24 週	n	64	67	
			Mean±SD	73.4±15.1	75.0±16.2	0.57
			Median [Q1, Q3]	73.0 [63.0, 82.5]	72.0 [62.0, 86.0]	0.76
			Min, Max	43.0, 120.0	51.0, 130.0	
	変化量	12 週	n	64	69	
			Mean±SD	2.8±10.1	1.1±13.2	0.39
			Median [Q1, Q3]	0.5 [-4.0, 7.5]	0.0 [-6.0, 10.0]	0.70
			Min, Max	-14.0, 33.0	-40.0, 29.0	
			One-sample t-test	0.029	0.50	
			Wilcoxon signed-rank test	0.12	0.36	
		24 週	n	64	67	
			Mean±SD	2.2±16.0	2.9±16.6	0.82
			Median [Q1, Q3]	0.0 [-6.5, 10.0]	1.0 [-8.0, 9.0]	0.92
			Min, Max	-47.0, 58.0	-31.0, 56.0	
			One-sample t-test	0.27	0.16	
			Wilcoxon signed-rank test	0.30	0.39	
NYHA 分類	測定値	0 週	n	70	70	
			Median [Q1, Q3]	1.0 [1.0, 2.0]	1.0 [1.0, 2.0]	0.47
			Min, Max	1.0, 2.0	1.0, 2.0	
		12 週	n	68	70	
			Median [Q1, Q3]	1.0 [1.0, 2.0]	1.0 [1.0, 2.0]	0.90
			Min, Max	1.0, 2.0	1.0, 2.0	
		24 週	n	66	66	
			Median [Q1, Q3]	1.0 [1.0, 2.0]	1.0 [1.0, 2.0]	0.59
			Min, Max	1.0, 3.0	1.0, 4.0	
	変化量	12 週	n	68	70	
			Median [Q1, Q3]	0.0 [0.0, 0.0]	0.0 [0.0, 0.0]	0.41
			Min, Max	-1.0, 1.0	-1.0, 1.0	
			Wilcoxon signed-rank test	1.00	0.51	
		24 週	n	66	66	
			Median [Q1, Q3]	0.0 [0.0, 0.0]	0.0 [0.0, 0.0]	0.08
			Min, Max	-1.0, 1.0	-1.0, 2.0	
			Wilcoxon signed-rank test	1.00	0.07	
赤血球数(× 10^4/μL)	測定値	0 週	n	69	69	
			Mean±SD	444.6±46.3	432.8±65.4	0.23
			Median [Q1, Q3]	448.0 [411.0, 472.0]	432.0 [384.0, 478.0]	0.21
			Min, Max	355.0, 568.0	274.0, 577.0	
		12 週	n	69	70	
			Mean±SD	443.2±52.2	426.6±71.2	0.12
			Median [Q1, Q3]	433.0 [406.0, 485.0]	422.5 [378.0, 484.0]	0.18

			Min, Max	303.0, 565.0	234.0, 570.0	
		24 週	n	67	67	
			Mean±SD	443.4±56.7	423.5±69.7	0.07
			Median [Q1, Q3]	448.0 [410.0, 481.0]	412.0 [381.0, 469.0]	0.05
			Min, Max	313.0, 598.0	284.0, 580.0	
	変化量	12 週	n	68	69	
			Mean±SD	-0.9±25.8	-5.6±34.4	0.37
			Median [Q1, Q3]	1.0 [-20.5, 15.0]	-3.0 [-24.0, 18.0]	0.58
			Min, Max	-55.0, 73.0	-135.0, 70.0	
			One-sample t-test	0.76	0.18	
			Wilcoxon signed-rank test	0.88	0.36	
		24 週	n	66	66	
			Mean±SD	-2.2±30.2	-10.2±31.2	0.13
			Median [Q1, Q3]	-1.5 [-17.0, 20.0]	-11.0 [-26.0, 10.0]	0.09
			Min, Max	-125.0, 62.0	-154.0, 53.0	
			One-sample t-test	0.56	0.010	
			Wilcoxon signed-rank test	0.82	0.011	
白血球数 (/μL)	測定値	0 週	n	69	69	
			Mean±SD	5688.4±1312.9	6140.0±1813.8	0.10
			Median [Q1, Q3]	5500.0 [4900.0, 6600.0]	5800.0 [5000.0, 7000.0]	0.17
			Min, Max	2900.0, 9400.0	2500.0, 11700.0	
		12 週	n	69	70	
			Mean±SD	5802.9±1480.8	6072.9±1742.6	0.33
			Median [Q1, Q3]	5600.0 [4800.0, 6900.0]	6100.0 [4700.0, 7100.0]	0.43
			Min, Max	3200.0, 9800.0	3100.0, 11400.0	
		24 週	n	67	67	
			Mean±SD	5694.0±1412.1	5965.7±1605.2	0.30
			Median [Q1, Q3]	5600.0 [4600.0, 6400.0]	5800.0 [4900.0, 6400.0]	0.40
			Min, Max	3000.0, 10100.0	3100.0, 11100.0	
	変化量	12 週	n	68	69	
			Mean±SD	142.6±1121.2	-51.6±1282.7	0.35
			Median [Q1, Q3]	200.0 [-600.0, 1000.0]	0.0 [-700.0, 400.0]	0.39
			Min, Max	-2100.0, 2600.0	-5900.0, 2600.0	
			One-sample t-test	0.30	0.74	
			Wilcoxon signed-rank test	0.35	0.68	
		24 週	n	66	66	
			Mean±SD	78.8±973.9	-84.2±1190.9	0.39
			Median [Q1, Q3]	100.0 [-300.0, 600.0]	-100.0 [-600.0, 400.0]	0.15
			Min, Max	-3400.0, 2900.0	-4200.0, 3400.0	
			One-sample t-test	0.51	0.57	
			Wilcoxon signed-rank test	0.27	0.35	
ヘモグロビン (g/dL)	測定値	0 週	n	69	69	
			Mean±SD	13.6±1.5	13.3±1.9	0.25
			Median [Q1, Q3]	13.8 [12.7, 14.7]	13.1 [12.1, 14.7]	0.22

			Min, Max	9.0, 16.6	8.8, 17.1	
		12 週	n	69	70	
			Mean±SD	13.5±1.6	13.2±2.2	0.29
			Median [Q1, Q3]	13.5 [12.4, 14.9]	13.1 [11.4, 14.8]	0.36
			Min, Max	9.2, 16.5	7.4, 16.9	
		24 週	n	67	67	
			Mean±SD	13.4±1.9	13.1±2.1	0.38
			Median [Q1, Q3]	13.7 [12.1, 15.0]	12.9 [11.7, 14.8]	0.32
			Min, Max	8.5, 17.0	8.5, 17.5	
	変化量	12 週	n	68	69	
			Mean±SD	-0.1±0.8	-0.1±1.0	0.91
			Median [Q1, Q3]	-0.1 [-0.6, 0.3]	0.0 [-0.7, 0.5]	0.77
			Min, Max	-1.9, 2.2	-4.0, 1.7	
			One-sample t-test	0.24	0.26	
			Wilcoxon signed-rank test	0.25	0.49	
		24 週	n	66	66	
			Mean±SD	-0.2±0.9	-0.2±1.0	0.99
			Median [Q1, Q3]	-0.1 [-0.7, 0.4]	-0.3 [-0.8, 0.3]	0.76
			Min, Max	-3.6, 1.5	-4.8, 2.6	
			One-sample t-test	0.06	0.08	
			Wilcoxon signed-rank test	0.13	0.049	
ヘマトクリット (%)	測定値	0 週	n	69	69	
			Mean±SD	41.3±4.2	40.2±5.2	0.18
			Median [Q1, Q3]	41.6 [38.7, 43.8]	40.1 [36.5, 43.5]	0.13
			Min, Max	28.3, 51.6	27.4, 51.1	
		12 週	n	69	70	
			Mean±SD	41.1±4.7	40.0±6.0	0.24
			Median [Q1, Q3]	41.0 [38.0, 44.4]	39.6 [35.6, 44.8]	0.30
			Min, Max	28.5, 51.9	24.6, 50.4	
		24 週	n	67	67	
			Mean±SD	40.8±5.4	39.7±5.9	0.26
			Median [Q1, Q3]	41.1 [37.0, 44.6]	39.1 [35.5, 43.3]	0.21
			Min, Max	28.0, 53.5	26.9, 52.2	
	変化量	12 週	n	68	69	
			Mean±SD	-0.2±2.3	-0.2±3.0	0.99
			Median [Q1, Q3]	0.1 [-1.3, 1.4]	-0.1 [-1.6, 1.6]	0.74
			Min, Max	-6.3, 5.9	-13.1, 5.1	
			One-sample t-test	0.58	0.67	
			Wilcoxon signed-rank test	0.77	0.95	
		24 週	n	66	66	
			Mean±SD	-0.5±2.9	-0.6±2.9	0.84
			Median [Q1, Q3]	-0.3 [-1.8, 1.6]	-0.6 [-2.4, 1.2]	0.53
			Min, Max	-12.1, 4.9	-15.0, 5.0	
			One-sample t-test	0.20	0.12	
			Wilcoxon signed-rank test	0.48	0.11	
血小板数(× 10^4/μL)	測定値	0 週	n	69	69	
			Mean±SD	19.8±7.9	19.8±6.5	0.95
			Median [Q1, Q3]	19.3 [15.2, 22.1]	19.1 [15.7, 22.6]	0.92
			Min, Max	8.6, 68.6	9.9, 46.8	

		12 週	n	69	70	
			Mean±SD	20.3±6.3	20.2±5.8	0.94
			Median [Q1, Q3]	19.6 [16.6, 22.8]	20.1 [15.7, 23.5]	0.92
			Min, Max	8.0, 37.1	10.1, 37.1	
		24 週	n	67	67	
			Mean±SD	20.1±5.6	19.4±5.3	0.41
			Median [Q1, Q3]	19.8 [16.6, 23.4]	18.8 [16.0, 22.5]	0.41
			Min, Max	8.6, 39.4	8.0, 37.0	
	変化量	12 週	n	68	69	
			Mean±SD	0.5±5.8	0.5±4.1	0.93
			Median [Q1, Q3]	0.6 [-0.5, 2.7]	0.3 [-0.9, 1.8]	0.37
			Min, Max	-35.2, 14.3	-13.1, 21.7	
			One-sample t-test	0.52	0.29	
			Wilcoxon signed-rank test	0.016	0.16	
		24 週	n	66	66	
			Mean±SD	0.9±2.9	0.1±2.7	0.08
			Median [Q1, Q3]	0.9 [-0.5, 2.5]	-0.2 [-1.5, 1.1]	0.021
			Min, Max	-6.4, 8.6	-6.0, 11.5	
			One-sample t-test	0.010	0.80	
			Wilcoxon signed-rank test	0.006	0.69	
AST(IU/L)	測定値	0 週	n	69	69	
			Mean±SD	26.3±12.2	23.6±10.1	0.16
			Median [Q1, Q3]	24.0 [20.0, 29.0]	22.0 [17.0, 27.0]	0.040
			Min, Max	13.0, 106.0	12.0, 85.0	
		12 週	n	69	70	
			Mean±SD	26.5±9.0	24.8±8.8	0.27
			Median [Q1, Q3]	25.0 [21.0, 31.0]	23.0 [19.0, 28.0]	0.17
			Min, Max	14.0, 63.0	11.0, 57.0	
		24 週	n	67	67	
			Mean±SD	25.3±6.6	24.9±9.0	0.74
			Median [Q1, Q3]	24.0 [20.0, 30.0]	22.0 [19.0, 27.0]	0.28
			Min, Max	16.0, 45.0	13.0, 55.0	
	変化量	12 週	n	68	69	
			Mean±SD	0.3±11.5	1.3±7.3	0.52
			Median [Q1, Q3]	1.0 [-2.0, 3.5]	1.0 [-2.0, 5.0]	0.45
			Min, Max	-73.0, 35.0	-28.0, 23.0	
			One-sample t-test	0.85	0.13	
			Wilcoxon signed-rank test	0.21	0.023	
		24 週	n	66	66	
			Mean±SD	-0.5±10.5	1.2±7.7	0.28
			Median [Q1, Q3]	1.0 [-3.0, 4.0]	1.0 [-2.0, 4.0]	0.44
			Min, Max	-73.0, 11.0	-30.0, 27.0	
			One-sample t-test	0.71	0.19	
			Wilcoxon signed-rank test	0.31	0.048	
ALT(IU/L)	測定値	0 週	n	69	69	
			Mean±SD	20.3±11.3	18.0±10.3	0.21
			Median [Q1, Q3]	18.0 [14.0, 24.0]	16.0 [12.0, 21.0]	0.037
			Min, Max	8.0, 87.0	6.0, 63.0	
		12 週	n	69	70	

			Mean±SD	20.8±10.1	18.5±9.0	0.16
			Median [Q1, Q3]	18.0 [14.0, 24.0]	16.0 [13.0, 23.0]	0.14
			Min, Max	8.0, 60.0	6.0, 48.0	
		24 週	n	67	67	
			Mean±SD	20.2±9.7	19.2±10.8	0.57
			Median [Q1, Q3]	19.0 [14.0, 24.0]	16.0 [11.0, 25.0]	0.28
			Min, Max	8.0, 60.0	7.0, 61.0	
	変化量	12 週	n	68	69	
			Mean±SD	0.3±10.1	0.6±7.2	0.81
			Median [Q1, Q3]	0.5 [-2.0, 4.0]	1.0 [-1.0, 3.0]	0.78
			Min, Max	-66.0, 21.0	-27.0, 25.0	
			One-sample t-test	0.84	0.49	
			Wilcoxon signed-rank test	0.23	0.09	
		24 週	n	66	66	
			Mean±SD	0.0±10.9	1.2±8.3	0.47
			Median [Q1, Q3]	0.0 [-2.0, 3.0]	0.0 [-2.0, 4.0]	0.86
			Min, Max	-72.0, 32.0	-20.0, 47.0	
			One-sample t-test	0.99	0.24	
			Wilcoxon signed-rank test	0.48	0.27	
ALP(IU/L)	測定値	0 週	n	66	68	
			Mean±SD	229.7±93.9	222.4±76.3	0.62
			Median [Q1, Q3]	202.5 [176.0, 241.0]	206.5 [172.5, 267.0]	0.96
			Min, Max	107.0, 636.0	86.0, 473.0	
		12 週	n	65	69	
			Mean±SD	240.9±93.1	233.8±81.0	0.64
			Median [Q1, Q3]	218.0 [186.0, 265.0]	216.0 [182.0, 289.0]	0.79
			Min, Max	110.0, 658.0	94.0, 492.0	
		24 週	n	66	67	
			Mean±SD	239.6±90.8	237.0±91.1	0.87
			Median [Q1, Q3]	223.0 [169.0, 273.0]	220.0 [179.0, 266.0]	0.98
			Min, Max	122.0, 525.0	96.0, 583.0	
	変化量	12 週	n	62	67	
			Mean±SD	6.6±37.7	12.5±43.4	0.41
			Median [Q1, Q3]	4.0 [-11.0, 22.0]	19.0 [1.0, 30.0]	0.05
			Min, Max	-91.0, 101.0	-182.0, 141.0	
			One-sample t-test	0.17	0.021	
			Wilcoxon signed-rank test	0.20	<0.001	
		24 週	n	62	65	
			Mean±SD	7.5±53.2	15.8±53.0	0.38
			Median [Q1, Q3]	3.0 [-21.0, 33.0]	15.0 [-7.0, 33.0]	0.18
			Min, Max	-152.0, 222.0	-173.0, 264.0	
			One-sample t-test	0.27	0.019	
			Wilcoxon signed-rank test	0.31	0.002	
T-Bil(mg/dL)	測定値	0 週	n	69	69	
			Mean±SD	0.90±0.38	0.82±0.42	0.23
			Median [Q1, Q3]	0.80 [0.70, 1.01]	0.70 [0.60, 0.90]	0.06
			Min, Max	0.27, 2.50	0.30, 2.79	
		12 週	n	68	69	
			Mean±SD	0.82±0.35	0.77±0.36	0.42

			Median [Q1, Q3]	0.76 [0.56, 1.00]	0.70 [0.50, 0.90]	0.26
			Min, Max	0.26, 1.70	0.30, 2.01	
		24 週	n	67	67	
			Mean±SD	0.83±0.34	0.78±0.37	0.38
			Median [Q1, Q3]	0.80 [0.60, 1.01]	0.70 [0.50, 0.90]	0.21
			Min, Max	0.29, 1.80	0.30, 2.30	
	変化量	12 週	n	67	68	
			Mean±SD	−0.08±0.31	−0.04±0.32	0.47
			Median [Q1, Q3]	0.00 [−0.20, 0.10]	−0.10 [−0.20, 0.10]	0.92
			Min, Max	−1.00, 0.48	−0.78, 1.21	
			One-sample t-test	0.030	0.26	
			Wilcoxon signed-rank test	0.05	0.043	
		24 週	n	66	66	
			Mean±SD	−0.07±0.30	−0.05±0.27	0.68
			Median [Q1, Q3]	−0.05 [−0.20, 0.10]	−0.10 [−0.20, 0.14]	0.80
			Min, Max	−0.80, 0.94	−0.60, 1.00	
			One-sample t-test	0.08	0.18	
			Wilcoxon signed-rank test	0.06	0.14	
γ GTP(U/L)	測定値	0 週	n	68	68	
			Mean±SD	51.4±46.6	39.9±38.6	0.12
			Median [Q1, Q3]	35.0 [23.0, 60.0]	26.0 [19.5, 42.5]	0.028
			Min, Max	10.0, 283.0	9.0, 235.0	
		12 週	n	68	69	
			Mean±SD	56.6±52.1	48.7±70.5	0.46
			Median [Q1, Q3]	36.5 [24.5, 70.0]	26.0 [19.0, 41.0]	0.019
			Min, Max	9.0, 265.0	10.0, 522.0	
		24 週	n	66	67	
			Mean±SD	56.4±53.5	41.9±40.3	0.08
			Median [Q1, Q3]	36.5 [23.0, 65.0]	28.0 [17.0, 42.0]	0.024
			Min, Max	8.0, 247.0	11.0, 209.0	
	変化量	12 週	n	66	67	
			Mean±SD	4.7±24.9	9.6±61.8	0.55
			Median [Q1, Q3]	1.5 [−3.0, 10.0]	1.0 [−3.0, 5.0]	0.39
			Min, Max	−71.0, 88.0	−33.0, 493.0	
			One-sample t-test	0.13	0.21	
			Wilcoxon signed-rank test	0.046	0.29	
		24 週	n	64	65	
			Mean±SD	7.3±28.6	2.2±18.5	0.23
			Median [Q1, Q3]	1.0 [−3.5, 10.5]	1.0 [−3.0, 4.0]	0.55
			Min, Max	−50.0, 145.0	−61.0, 68.0	
			One-sample t-test	0.045	0.34	
			Wilcoxon signed-rank test	0.10	0.44	
対数変換 γ GTP(ln (U/L))	測定値	0 週	n	68	68	
			Mean±SD	3.65±0.74	3.40±0.70	0.044
			Median [Q1, Q3]	3.56 [3.14, 4.09]	3.26 [2.97, 3.75]	0.028
			Min, Max	2.30, 5.65	2.20, 5.46	
		12 週	n	68	69	
			Mean±SD	3.73±0.76	3.46±0.80	0.048
			Median [Q1, Q3]	3.60 [3.20, 4.25]	3.26 [2.94, 3.71]	0.019

			Min, Max	2.20, 5.58	2.30, 6.26	
		24 週	n	66	67	
			Mean±SD	3.71±0.78	3.43±0.73	0.035
			Median [Q1, Q3]	3.60 [3.14, 4.17]	3.33 [2.83, 3.74]	0.024
			Min, Max	2.08, 5.51	2.40, 5.34	
	変化量	12 週	n	66	67	
			Mean±SD	0.06±0.26	0.08±0.43	0.77
			Median [Q1, Q3]	0.05 [−0.09, 0.23]	0.02 [−0.12, 0.18]	0.47
			Min, Max	−0.67, 0.67	−0.57, 2.89	
			One-sample t-test	0.07	0.14	
			Wilcoxon signed-rank test	0.040	0.31	
		24 週	n	64	65	
			Mean±SD	0.10±0.38	0.03±0.27	0.25
			Median [Q1, Q3]	0.03 [−0.10, 0.23]	0.05 [−0.13, 0.17]	0.48
			Min, Max	−0.77, 1.46	−0.59, 0.91	
			One-sample t-test	0.037	0.33	
			Wilcoxon signed-rank test	0.10	0.50	
TC(mg/dL)	測定値	0 週	n	68	69	
			Mean±SD	176.9±30.8	166.9±32.1	0.06
			Median [Q1, Q3]	179.0 [155.5, 194.5]	162.0 [146.0, 187.0]	0.05
			Min, Max	110.0, 256.0	96.0, 254.0	
		12 週	n	68	69	
			Mean±SD	171.4±38.0	168.0±39.5	0.62
			Median [Q1, Q3]	169.0 [148.0, 193.5]	164.0 [141.0, 188.0]	0.59
			Min, Max	69.0, 276.0	90.0, 264.0	
		24 週	n	67	67	
			Mean±SD	174.0±33.1	162.1±31.8	0.036
			Median [Q1, Q3]	173.0 [151.0, 193.0]	161.0 [142.0, 180.0]	0.06
			Min, Max	112.0, 249.0	97.0, 243.0	
	変化量	12 週	n	66	68	
			Mean±SD	−5.2±25.5	0.2±22.3	0.20
			Median [Q1, Q3]	−6.0 [−18.0, 9.0]	−4.0 [−15.5, 16.0]	0.40
			Min, Max	−91.0, 44.0	−49.0, 51.0	
			One-sample t-test	0.10	0.95	
			Wilcoxon signed-rank test	0.16	0.78	
		24 週	n	65	66	
			Mean±SD	−1.8±24.5	−5.8±24.5	0.35
			Median [Q1, Q3]	2.0 [−10.0, 12.0]	−6.0 [−16.0, 6.0]	0.13
			Min, Max	−69.0, 69.0	−127.0, 33.0	
			One-sample t-test	0.56	0.06	
			Wilcoxon signed-rank test	0.83	0.06	
HDL-C (mg/dL)	測定値	0 週	n	69	69	
			Mean±SD	56.3±16.1	55.4±18.3	0.75
			Median [Q1, Q3]	53.0 [47.0, 67.0]	51.0 [43.0, 63.0]	0.38
			Min, Max	30.0, 105.0	30.0, 135.0	
		12 週	n	68	69	
			Mean±SD	56.2±14.6	56.8±20.4	0.83
			Median [Q1, Q3]	56.0 [45.0, 65.0]	51.0 [44.0, 61.0]	0.44
			Min, Max	27.0, 103.0	30.0, 149.0	

		24 週	n	66	67	
			Mean±SD	55.3±14.1	54.0±17.1	0.62
			Median [Q1, Q3]	53.0 [46.0, 62.0]	51.0 [43.0, 58.0]	0.27
			Min, Max	33.0, 98.0	31.0, 139.0	
	変化量	12 週	n	67	68	
			Mean±SD	-0.7±9.4	1.7±7.7	0.11
			Median [Q1, Q3]	0.0 [-7.0, 5.0]	0.0 [-3.0, 5.5]	0.31
			Min, Max	-30.0, 29.0	-13.0, 26.0	
			One-sample t-test	0.56	0.07	
			Wilcoxon signed-rank test	0.68	0.24	
		24 週	n	65	66	
			Mean±SD	-0.8±8.5	-1.4±9.0	0.70
			Median [Q1, Q3]	-1.0 [-6.0, 4.0]	0.0 [-4.0, 4.0]	0.79
			Min, Max	-23.0, 23.0	-39.0, 15.0	
			One-sample t-test	0.43	0.20	
			Wilcoxon signed-rank test	0.44	0.64	
TG(mg/dL)	測定値	0 週	n	67	69	
			Mean±SD	111.4±49.1	118.8±64.1	0.46
			Median [Q1, Q3]	99.0 [76.0, 145.0]	104.0 [73.0, 142.0]	0.75
			Min, Max	32.0, 280.0	44.0, 408.0	
		12 週	n	65	64	
			Mean±SD	126.4±64.2	124.6±69.6	0.88
			Median [Q1, Q3]	110.0 [71.0, 167.0]	103.5 [80.0, 139.0]	0.86
			Min, Max	42.0, 338.0	22.0, 420.0	
		24 週	n	66	66	
			Mean±SD	111.0±55.8	112.2±58.4	0.90
			Median [Q1, Q3]	99.5 [70.0, 134.0]	98.5 [70.0, 138.0]	0.99
			Min, Max	46.0, 362.0	47.0, 348.0	
	変化量	12 週	n	62	64	
			Mean±SD	16.8±42.0	7.7±63.6	0.35
			Median [Q1, Q3]	13.0 [-6.0, 38.0]	1.5 [-16.0, 35.0]	0.13
			Min, Max	-101.0, 121.0	-191.0, 284.0	
			One-sample t-test	0.003	0.34	
			Wilcoxon signed-rank test	0.001	0.35	
		24 週	n	63	65	
			Mean±SD	2.1±36.6	-7.1±43.0	0.19
			Median [Q1, Q3]	4.0 [-21.0, 23.0]	-4.0 [-22.0, 18.0]	0.32
			Min, Max	-65.0, 136.0	-203.0, 113.0	
			One-sample t-test	0.65	0.19	
			Wilcoxon signed-rank test	0.86	0.30	
LDL-C (mg/dL)	測定値	0 週	n	69	69	
			Mean±SD	103.2±29.7	93.8±24.5	0.045
			Median [Q1, Q3]	101.0 [82.0, 125.0]	92.0 [79.0, 106.0]	0.048
			Min, Max	45.0, 176.0	38.0, 160.0	
		12 週	n	68	69	
			Mean±SD	98.7±31.7	93.0±30.3	0.29
			Median [Q1, Q3]	93.5 [73.0, 118.0]	88.0 [74.0, 109.0]	0.37
			Min, Max	45.0, 180.0	35.0, 174.0	
		24 週	n	66	67	

			Mean±SD	100.7±27.8	91.1±26.7	0.046
			Median [Q1, Q3]	100.5 [78.0, 121.0]	90.0 [72.0, 110.0]	0.05
			Min, Max	44.0, 164.0	35.0, 152.0	
	変化量	12 週	n	67	68	
			Mean±SD	-4.2±21.1	-1.9±17.6	0.49
			Median [Q1, Q3]	-5.0 [-12.0, 6.0]	-3.0 [-13.5, 9.0]	0.76
			Min, Max	-75.0, 37.0	-43.0, 47.0	
			One-sample t-test	0.11	0.38	
			Wilcoxon signed-rank test	0.13	0.20	
		24 週	n	65	66	
			Mean±SD	-0.9±20.1	-3.7±19.9	0.43
			Median [Q1, Q3]	0.0 [-7.0, 10.0]	-4.5 [-11.0, 6.0]	0.15
			Min, Max	-68.0, 52.0	-113.0, 45.0	
			One-sample t-test	0.71	0.14	
			Wilcoxon signed-rank test	0.88	0.10	
Na(mEq/L)	測定値	0 週	n	69	69	
			Mean±SD	140.5±3.2	140.5±2.2	0.93
			Median [Q1, Q3]	141.0 [139.0, 142.0]	141.0 [139.0, 142.0]	0.72
			Min, Max	121.0, 145.0	135.0, 146.0	
		12 週	n	69	70	
			Mean±SD	140.4±2.6	140.8±4.0	0.58
			Median [Q1, Q3]	140.0 [139.0, 142.0]	141.0 [139.0, 142.0]	0.25
			Min, Max	129.0, 146.0	114.0, 152.0	
		24 週	n	67	67	
			Mean±SD	140.5±2.5	140.3±1.9	0.67
			Median [Q1, Q3]	141.0 [139.0, 142.0]	140.0 [139.0, 142.0]	0.39
			Min, Max	134.0, 146.0	136.0, 145.0	
	変化量	12 週	n	68	69	
			Mean±SD	-0.2±2.2	0.2±4.1	0.53
			Median [Q1, Q3]	0.0 [-2.0, 1.0]	0.0 [-1.0, 2.0]	0.07
			Min, Max	-5.0, 8.0	-27.0, 9.0	
			One-sample t-test	0.55	0.70	
			Wilcoxon signed-rank test	0.36	0.14	
		24 週	n	66	66	
			Mean±SD	-0.1±2.5	-0.2±2.1	0.88
			Median [Q1, Q3]	0.0 [-1.0, 1.0]	0.0 [-2.0, 1.0]	0.86
			Min, Max	-5.0, 14.0	-6.0, 7.0	
			One-sample t-test	0.77	0.57	
			Wilcoxon signed-rank test	0.30	0.40	
血糖値(mg/dL)	測定値	0 週	n	67	69	
			Mean±SD	105.9±18.2	105.6±17.1	0.92
			Median [Q1, Q3]	102.0 [94.0, 115.0]	103.0 [95.0, 110.0]	0.97
			Min, Max	80.0, 179.0	83.0, 160.0	
		12 週	n	66	63	
			Mean±SD	112.9±24.8	118.0±36.9	0.35
			Median [Q1, Q3]	107.0 [98.0, 121.0]	107.0 [95.0, 129.0]	0.98
			Min, Max	49.0, 201.0	57.0, 301.0	
		24 週	n	66	66	
			Mean±SD	108.1±19.6	106.9±17.2	0.72

			Median [Q1, Q3]	103.0 [96.0, 117.0]	102.0 [96.0, 115.0]	0.65
			Min, Max	77.0, 186.0	83.0, 162.0	
	変化量	12 週	n	63	63	
			Mean±SD	7.0±19.1	12.2±25.8	0.20
			Median [Q1, Q3]	6.0 [-1.0, 17.0]	7.0 [-1.0, 20.0]	0.65
			Min, Max	-74.0, 55.0	-42.0, 141.0	
			One-sample t-test	0.005	<0.001	
			Wilcoxon signed-rank test	<0.001	<0.001	
		24 週	n	63	65	
			Mean±SD	1.3±12.4	2.0±11.8	0.72
			Median [Q1, Q3]	2.0 [-6.0, 8.0]	3.0 [-2.0, 6.0]	0.72
			Min, Max	-33.0, 41.0	-45.0, 53.0	
			One-sample t-test	0.41	0.17	
			Wilcoxon signed-rank test	0.39	0.045	
BUN(血中浸透圧)(mg/dL)	測定値	0 週	n	69	69	
			Mean±SD	19.7±6.6	19.7±5.3	0.96
			Median [Q1, Q3]	18.0 [15.0, 23.0]	19.0 [16.0, 22.0]	0.56
			Min, Max	10.0, 42.0	10.0, 35.0	
		12 週	n	69	70	
			Mean±SD	20.6±8.7	19.9±8.7	0.64
			Median [Q1, Q3]	18.0 [15.0, 23.5]	18.6 [15.0, 22.0]	0.60
			Min, Max	8.0, 67.0	9.0, 70.0	
		24 週	n	67	67	
			Mean±SD	20.9±8.8	19.8±7.8	0.48
			Median [Q1, Q3]	19.0 [15.2, 25.0]	19.0 [15.0, 22.0]	0.59
			Min, Max	9.0, 57.0	5.1, 48.0	
	変化量	12 週	n	68	69	
			Mean±SD	1.2±5.9	0.2±7.9	0.42
			Median [Q1, Q3]	1.0 [-2.0, 4.0]	0.0 [-3.0, 3.0]	0.30
			Min, Max	-10.9, 25.0	-18.7, 48.0	
			One-sample t-test	0.11	0.83	
			Wilcoxon signed-rank test	0.14	0.94	
		24 週	n	66	66	
			Mean±SD	1.5±6.2	0.4±6.5	0.33
			Median [Q1, Q3]	1.0 [-2.0, 4.0]	0.0 [-3.0, 3.0]	0.27
			Min, Max	-13.0, 27.0	-17.3, 25.0	
			One-sample t-test	0.06	0.63	
			Wilcoxon signed-rank test	0.10	0.95	
血清クレアチニン(mg/dL)	測定値	0 週	n	69	68	
			Mean±SD	1.05±0.21	1.03±0.24	0.58
			Median [Q1, Q3]	1.07 [0.87, 1.18]	0.98 [0.87, 1.18]	0.38
			Min, Max	0.65, 1.63	0.58, 1.71	
		12 週	n	69	70	
			Mean±SD	1.06±0.25	1.04±0.29	0.65
			Median [Q1, Q3]	1.06 [0.84, 1.17]	0.97 [0.84, 1.22]	0.42
			Min, Max	0.70, 1.88	0.53, 2.00	
		24 週	n	67	67	
			Mean±SD	1.07±0.28	1.04±0.30	0.60
			Median [Q1, Q3]	1.05 [0.83, 1.22]	0.96 [0.82, 1.25]	0.49

			Min, Max	0.57, 2.20	0.56, 2.01	
	変化量	12 週	n	68	68	
			Mean±SD	0.02±0.15	0.01±0.18	0.67
			Median [Q1, Q3]	0.02 [-0.05, 0.09]	0.02 [-0.06, 0.08]	0.78
			Min, Max	-0.33, 0.78	-0.86, 0.63	
			One-sample t-test	0.23	0.66	
			Wilcoxon signed-rank test	0.22	0.45	
		24 週	n	66	65	
			Mean±SD	0.03±0.17	0.02±0.14	0.84
			Median [Q1, Q3]	-0.01 [-0.05, 0.08]	0.02 [-0.09, 0.10]	0.61
			Min, Max	-0.30, 0.74	-0.35, 0.42	
			One-sample t-test	0.18	0.20	
			Wilcoxon signed-rank test	0.54	0.21	
クレアチニン・クリアランス (mL/min)	測定値	0 週	n	69	68	
			Mean±SD	64.1±31.6	66.8±29.7	0.61
			Median [Q1, Q3]	55.9 [43.5, 80.4]	60.8 [45.4, 76.4]	0.35
			Min, Max	25.8, 204.0	31.4, 164.6	
		12 週	n	62	65	
			Mean±SD	62.1±30.6	66.3±31.1	0.44
			Median [Q1, Q3]	53.3 [41.0, 81.5]	57.1 [44.1, 82.1]	0.43
			Min, Max	21.6, 213.3	19.3, 182.2	
		24 週	n	64	66	
			Mean±SD	63.5±32.3	65.4±32.0	0.73
			Median [Q1, Q3]	56.2 [42.1, 78.5]	56.6 [39.9, 82.6]	0.82
			Min, Max	21.3, 216.7	24.7, 172.4	
	変化量	12 週	n	62	63	
			Mean±SD	-1.0±5.3	0.1±10.6	0.45
			Median [Q1, Q3]	-1.5 [-4.7, 1.6]	-1.7 [-6.1, 5.8]	0.79
			Min, Max	-12.6, 12.2	-24.4, 45.6	
			One-sample t-test	0.14	0.92	
			Wilcoxon signed-rank test	0.07	0.72	
		24 週	n	63	64	
			Mean±SD	-0.6±9.2	-0.8±9.3	0.88
			Median [Q1, Q3]	0.1 [-3.3, 3.4]	-2.7 [-7.1, 5.4]	0.40
			Min, Max	-41.3, 30.6	-26.4, 24.4	
			One-sample t-test	0.61	0.47	
			Wilcoxon signed-rank test	0.78	0.27	
eGFR (mL/min/1.73m^2)	測定値	0 週	n	69	68	
			Mean±SD	54.4±15.6	56.5±16.8	0.46
			Median [Q1, Q3]	51.1 [43.5, 65.8]	53.5 [43.6, 67.2]	0.58
			Min, Max	29.4, 94.2	30.1, 98.3	
		12 週	n	69	70	
			Mean±SD	54.1±15.7	56.3±17.4	0.43
			Median [Q1, Q3]	51.9 [43.6, 64.6]	53.2 [42.5, 69.4]	0.62
			Min, Max	20.6, 95.7	27.3, 99.6	
		24 週	n	67	67	
			Mean±SD	54.1±16.2	56.3±18.1	0.46
			Median [Q1, Q3]	51.8 [43.4, 68.4]	52.6 [41.5, 68.8]	0.68
			Min, Max	23.1, 95.0	27.2, 106.8	

	変化量	12 週	n	68	68	
			Mean±SD	-0.6±5.9	0.3±8.6	0.50
			Median [Q1, Q3]	-0.6 [-4.0, 2.9]	-1.0 [-4.7, 4.9]	0.72
			Min, Max	-16.4, 16.2	-17.0, 40.5	
			One-sample t-test	0.41	0.79	
			Wilcoxon signed-rank test	0.39	0.84	
		24 週	n	66	65	
			Mean±SD	-0.7±7.9	-0.1±7.4	0.69
			Median [Q1, Q3]	0.3 [-4.2, 3.4]	-1.5 [-5.3, 4.2]	0.67
			Min, Max	-24.8, 28.3	-14.4, 17.6	
			One-sample t-test	0.50	0.90	
			Wilcoxon signed-rank test	0.61	0.55	
尿中クレアチン (mg/dl)	測定値	0 週	n	58	58	
			Mean±SD	97.5±55.9	105.7±58.3	0.44
			Median [Q1, Q3]	88.4 [62.7, 132.0]	99.5 [60.0, 155.0]	0.39
			Min, Max	14.0, 252.0	16.0, 277.0	
		12 週	n	56	58	
			Mean±SD	100.2±65.6	92.3±67.6	0.53
			Median [Q1, Q3]	90.0 [51.9, 126.5]	71.5 [42.0, 121.0]	0.40
			Min, Max	11.0, 334.0	7.0, 344.0	
		24 週	n	56	55	
			Mean±SD	103.8±83.1	87.0±50.3	0.20
			Median [Q1, Q3]	88.0 [53.0, 132.5]	83.0 [42.0, 130.0]	0.52
			Min, Max	14.8, 478.0	15.0, 238.0	
	変化量	12 週	n	56	57	
			Mean±SD	1.5±75.5	-14.7±74.6	0.25
			Median [Q1, Q3]	8.6 [-31.8, 40.0]	-9.0 [-56.0, 30.0]	0.25
			Min, Max	-197.0, 280.0	-214.0, 158.0	
			One-sample t-test	0.88	0.14	
			Wilcoxon signed-rank test	0.71	0.19	
		24 週	n	56	54	
			Mean±SD	4.7±81.5	-16.8±59.5	0.12
			Median [Q1, Q3]	5.0 [-42.3, 36.5]	-14.0 [-44.0, 18.0]	0.21
			Min, Max	-180.0, 328.0	-206.0, 120.0	
			One-sample t-test	0.67	0.042	
			Wilcoxon signed-rank test	0.91	0.039	
尿中浸透圧 (mOsm/Kg・H2O)	測定値	0 週	n	57	59	
			Mean±SD	517.4±191.9	555.8±194.7	0.29
			Median [Q1, Q3]	521.0 [405.0, 657.0]	588.0 [412.0, 700.0]	0.30
			Min, Max	163.0, 946.0	188.0, 1018.0	
		12 週	n	54	58	
			Mean±SD	497.2±152.8	519.8±186.1	0.49
			Median [Q1, Q3]	512.5 [395.0, 598.0]	515.0 [360.0, 652.0]	0.53
			Min, Max	144.5, 987.0	192.0, 1130.0	
		24 週	n	56	54	
			Mean±SD	537.4±193.2	496.8±194.9	0.27
			Median [Q1, Q3]	550.0 [404.5, 690.5]	476.5 [357.0, 629.0]	0.30
			Min, Max	174.0, 1039.0	130.0, 863.0	
	変化量	12 週	n	53	58	

			Mean±SD	−18.0±187.7	−37.6±200.5	0.60
			Median [Q1, Q3]	−20.0 [−147.0, 96.0]	−58.0 [−165.0, 75.0]	0.43
			Min, Max	−503.5, 432.0	−475.0, 594.0	
			One-sample t-test	0.49	0.16	
			Wilcoxon signed-rank test	0.45	0.07	
		24 週	n	55	54	
			Mean±SD	14.2±186.7	−56.5±164.4	0.038
			Median [Q1, Q3]	−8.0 [−113.0, 188.0]	−37.0 [−120.0, 15.0]	0.06
			Min, Max	−381.0, 556.0	−614.0, 447.0	
			One-sample t-test	0.57	0.015	
			Wilcoxon signed-rank test	0.79	0.006	

表 5.1.9. [FAS] 心臓超音波検査パラメーター

変数		観察ポイント	統計量	トピロキソスタット群	アロプリノール群	群間比較 P 値
左室駆出率 (EF) (M.simpson 法) (%)	測定値	0 週	n	70	68	
			Mean±SD	52.4±12.9	50.6±13.3	0.42
			Median [Q1, Q3]	55.0 [43.0, 61.8]	54.7 [41.9, 60.5]	0.52
			Min, Max	25.2, 74.0	19.3, 71.4	
		24 週	n	65	65	
			Mean±SD	53.1±12.5	52.6±14.2	0.82
			Median [Q1, Q3]	55.5 [43.0, 62.8]	54.7 [44.4, 61.5]	0.84
			Min, Max	20.7, 75.0	21.8, 77.7	
	変化量	24 週	n	65	63	
			Mean±SD	-0.4±5.6	1.6±5.3	0.040
			Median [Q1, Q3]	-0.5 [-4.0, 3.0]	1.0 [-2.5, 4.0]	0.036
			Min, Max	-13.7, 12.1	-10.0, 17.0	
			One-sample t-test	0.53	0.022	
			Wilcoxon signed-rank test	0.47	0.05	
左室拡張末期径(LVDd) (mm)	測定値	0 週	n	68	70	
			Mean±SD	53.3±8.3	53.2±8.8	0.98
			Median [Q1, Q3]	51.9 [47.8, 57.2]	52.2 [47.0, 57.4]	0.95
			Min, Max	38.1, 78.4	36.0, 76.2	
		24 週	n	64	66	
			Mean±SD	51.3±8.1	52.5±9.6	0.44
			Median [Q1, Q3]	50.3 [47.1, 56.1]	51.4 [47.4, 56.7]	0.42
			Min, Max	31.5, 72.4	10.5, 77.8	
	変化量	24 週	n	62	66	
			Mean±SD	-1.0±5.9	-0.9±6.5	0.89
			Median [Q1, Q3]	-1.0 [-4.3, 2.4]	-0.4 [-3.5, 1.9]	0.76
			Min, Max	-20.7, 16.2	-36.3, 16.0	
			One-sample t-test	0.18	0.29	
			Wilcoxon signed-rank test	0.21	0.38	
左室収縮末期径(LVDs) (mm)	測定値	0 週	n	64	68	
			Mean±SD	38.9±10.7	38.5±11.2	0.81
			Median [Q1, Q3]	36.7 [31.1, 46.5]	36.4 [31.2, 44.3]	0.89
			Min, Max	20.6, 68.2	12.7, 70.7	
		24 週	n	63	63	
			Mean±SD	36.6±9.3	38.5±11.1	0.32
			Median [Q1, Q3]	35.5 [29.7, 43.5]	36.5 [31.3, 46.3]	0.44
			Min, Max	20.0, 56.4	7.9, 69.9	
	変化量	24 週	n	57	61	
			Mean±SD	-0.6±5.4	-0.1±7.8	0.73
			Median [Q1, Q3]	-0.4 [-3.4, 2.8]	-0.8 [-2.8, 2.9]	0.95
			Min, Max	-17.2, 15.6	-26.6, 39.3	
			One-sample t-test	0.42	0.89	
			Wilcoxon signed-rank test	0.44	0.47	
僧帽弁口血流速波形(E) (cm/sec)	測定値	0 週	n	69	70	
			Mean±SD	81.8±41.1	75.7±37.1	0.36
			Median [Q1, Q3]	73.5 [55.9, 93.0]	65.6 [51.7, 87.9]	0.30
			Min, Max	37.3, 268.0	26.4, 214.8	

		24 週	n	66	66	
			Mean±SD	77.1±37.3	81.4±40.3	0.53
			Median [Q1, Q3]	68.7 [55.1, 86.2]	73.4 [53.8, 92.9]	0.60
			Min, Max	25.3, 241.0	31.0, 245.5	
	変化量	24 週	n	66	66	
			Mean±SD	-4.2±16.7	5.3±21.9	0.006
			Median [Q1, Q3]	-2.7 [-13.7, 6.0]	2.8 [-8.3, 13.3]	0.013
			Min, Max	-48.9, 44.2	-42.8, 96.1	
			One-sample t-test	0.046	0.05	
			Wilcoxon signed-rank test	0.043	0.14	
僧帽弁口血流速波形(A)(cm/sec)	測定値	0 週	n	35	42	
			Mean±SD	67.5±25.3	74.0±25.5	0.27
			Median [Q1, Q3]	70.3 [48.0, 88.0]	68.7 [58.6, 83.0]	0.62
			Min, Max	21.6, 113.0	30.9, 181.0	
		24 週	n	34	37	
			Mean±SD	62.4±25.1	73.2±23.7	0.07
			Median [Q1, Q3]	64.9 [50.7, 80.7]	70.0 [58.2, 86.4]	0.11
			Min, Max	14.6, 119.0	26.9, 122.0	
	変化量	24 週	n	32	36	
			Mean±SD	-3.5±18.0	-3.4±21.1	0.97
			Median [Q1, Q3]	-3.2 [-12.0, 6.0]	-3.5 [-9.8, 6.0]	0.83
			Min, Max	-64.3, 48.4	-100.0, 42.0	
			One-sample t-test	0.28	0.34	
			Wilcoxon signed-rank test	0.18	0.32	
僧帽弁口血流速波形(DT)(msec)	測定値	0 週	n	67	69	
			Mean±SD	204.1±51.2	220.1±66.5	0.12
			Median [Q1, Q3]	188.5 [174.1, 225.0]	204.6 [167.0, 253.5]	0.25
			Min, Max	105.6, 375.0	100.0, 400.0	
		24 週	n	65	66	
			Mean±SD	211.5±74.1	225.3±85.5	0.33
			Median [Q1, Q3]	184.0 [160.0, 240.0]	198.6 [160.0, 258.0]	0.55
			Min, Max	24.0, 531.7	104.0, 544.0	
	変化量	24 週	n	63	65	
			Mean±SD	0.7±50.2	-0.7±57.2	0.88
			Median [Q1, Q3]	0.0 [-27.0, 33.0]	-7.6 [-32.0, 33.0]	0.57
			Min, Max	-160.0, 165.0	-136.0, 156.2	
			One-sample t-test	0.91	0.92	
			Wilcoxon signed-rank test	0.67	0.78	
左室弁輪運動速波形(E/e')	測定値	0 週	n	58	67	
			Mean±SD	13.9±6.7	13.1±7.1	0.50
			Median [Q1, Q3]	12.7 [9.3, 16.4]	11.8 [8.5, 15.4]	0.38
			Min, Max	4.4, 38.2	3.6, 46.2	
		24 週	n	63	64	
			Mean±SD	12.2±5.9	13.2±6.3	0.33
			Median [Q1, Q3]	10.5 [8.6, 13.8]	11.7 [9.3, 15.5]	0.20
			Min, Max	4.9, 36.5	4.4, 34.5	
	変化量	24 週	n	54	62	
			Mean±SD	-1.2±4.4	0.3±5.4	0.10
			Median [Q1, Q3]	-0.8 [-4.0, 1.3]	0.7 [-3.2, 4.4]	0.10

			Min, Max	-10.5, 9.3	-11.7, 13.9	
			One-sample t-test	0.044	0.65	
			Wilcoxon signed-rank test	0.06	0.66	
下大静脈径 (IVCd) (mm)	測定値	0 週	n	68	65	
			Mean±SD	14.7±5.4	14.1±4.1	0.46
			Median [Q1, Q3]	14.7 [11.5, 17.0]	13.6 [11.0, 16.7]	0.56
			Min, Max	4.2, 43.0	3.2, 27.0	
		24 週	n	65	64	
			Mean±SD	16.0±6.5	15.2±5.9	0.47
			Median [Q1, Q3]	15.3 [12.4, 17.8]	14.6 [12.1, 17.2]	0.47
			Min, Max	8.0, 49.2	7.8, 51.2	
	変化量	24 週	n	63	60	
			Mean±SD	1.2±5.8	0.9±6.2	0.79
			Median [Q1, Q3]	0.8 [-3.1, 3.6]	0.7 [-2.3, 3.3]	0.85
			Min, Max	-6.8, 32.4	-10.0, 36.5	
			One-sample t-test	0.11	0.27	
			Wilcoxon signed-rank test	0.24	0.36	
左房短径 (LAD) (mm)	測定値	0 週	n	70	68	
			Mean±SD	45.5±7.4	43.4±7.9	0.11
			Median [Q1, Q3]	44.4 [40.0, 49.1]	42.7 [38.7, 47.8]	0.11
			Min, Max	30.9, 63.2	26.0, 64.8	
		24 週	n	66	66	
			Mean±SD	45.9±7.5	44.3±7.5	0.22
			Median [Q1, Q3]	45.0 [41.7, 49.5]	44.1 [38.8, 48.2]	0.18
			Min, Max	29.0, 66.8	31.8, 65.5	
	変化量	24 週	n	66	64	
			Mean±SD	0.6±4.9	0.9±5.5	0.74
			Median [Q1, Q3]	0.6 [-1.9, 3.1]	1.1 [-2.4, 4.6]	0.71
			Min, Max	-13.6, 14.6	-12.0, 20.6	
			One-sample t-test	0.31	0.18	
			Wilcoxon signed-rank test	0.21	0.15	
三尖弁圧較差 (TRPG) (mmHg)	測定値	0 週	n	60	55	
			Mean±SD	23.7±9.7	20.4±7.4	0.047
			Median [Q1, Q3]	22.2 [17.5, 27.3]	18.7 [16.2, 25.0]	0.038
			Min, Max	7.4, 70.3	4.0, 41.0	
		24 週	n	55	52	
			Mean±SD	24.3±7.6	22.4±8.9	0.23
			Median [Q1, Q3]	23.5 [19.1, 28.2]	20.6 [16.4, 27.7]	0.10
			Min, Max	10.0, 46.0	10.0, 51.0	
	変化量	24 週	n	52	45	
			Mean±SD	1.3±5.5	2.7±8.0	0.31
			Median [Q1, Q3]	1.0 [-1.8, 5.5]	2.0 [-1.0, 7.0]	0.27
			Min, Max	-12.1, 13.0	-20.7, 23.7	
			One-sample t-test	0.09	0.028	
			Wilcoxon signed-rank test	0.11	0.009	

表 5.1.10. [FAS] 特殊血液検査

変数		観察ポイント	統計量	トピロキソスタット群	アロプリノール群	群間比較 P 値
hsCRP(mg/dL)	測定値	0 週	n	69	69	
			Mean±SD	0.16±0.17	0.12±0.13	0.11
			Median [Q1, Q3]	0.09 [0.03, 0.25]	0.06 [0.03, 0.14]	0.28
			Min, Max	0.00, 0.50	0.01, 0.50	
		12 週	n	64	65	
			Mean±SD	0.16±0.17	0.14±0.14	0.43
			Median [Q1, Q3]	0.08 [0.04, 0.25]	0.09 [0.04, 0.18]	0.85
			Min, Max	0.00, 0.50	0.01, 0.50	
		24 週	n	65	62	
			Mean±SD	0.17±0.17	0.13±0.14	0.18
			Median [Q1, Q3]	0.09 [0.05, 0.22]	0.07 [0.04, 0.18]	0.16
			Min, Max	0.01, 0.50	0.01, 0.50	
	変化量	12 週	n	63	64	
			Mean±SD	0.00±0.17	0.02±0.15	0.55
			Median [Q1, Q3]	0.00 [-0.02, 0.05]	0.01 [-0.03, 0.07]	0.77
			Min, Max	-0.44, 0.39	-0.36, 0.47	
			One-sample t-test	0.93	0.33	
			Wilcoxon signed-rank test	0.40	0.24	
		24 週	n	64	61	
			Mean±SD	0.03±0.16	0.02±0.14	0.57
			Median [Q1, Q3]	0.01 [-0.01, 0.07]	0.00 [-0.01, 0.03]	0.34
			Min, Max	-0.45, 0.49	-0.37, 0.41	
			One-sample t-test	0.13	0.39	
			Wilcoxon signed-rank test	0.016	0.17	
	変化率	12 週	n	63	64	
			Mean±SD	107.7±462.6	102.9±284.2	0.94
			Median [Q1, Q3]	1.1 [-41.5, 110.0]	24.0 [-35.5, 117.5]	0.53
			Min, Max	-90.2, 3266.7	-91.5, 1751.9	
			One-sample t-test	0.07	0.005	
			Wilcoxon signed-rank test	0.05	0.006	
		24 週	n	64	61	
			Mean±SD	156.9±539.0	58.3±133.9	0.17
			Median [Q1, Q3]	26.7 [-14.0, 137.8]	16.4 [-18.8, 87.2]	0.38
			Min, Max	-93.9, 4066.7	-90.1, 497.8	
			One-sample t-test	0.023	0.001	
			Wilcoxon signed-rank test	<0.001	0.004	
対数変換 hsCRP(ln (mg/dL))	測定値	0 週	n	69	69	
			Mean±SD	-2.50±1.24	-2.72±1.10	0.28
			Median [Q1, Q3]	-2.47 [-3.41, -1.37]	-2.86 [-3.54, -1.97]	0.28
			Min, Max	-5.52, -0.69	-4.96, -0.69	
		12 週	n	64	65	
			Mean±SD	-2.47±1.20	-2.51±1.06	0.82
			Median [Q1, Q3]	-2.58 [-3.32, -1.40]	-2.47 [-3.32, -1.74]	0.85
			Min, Max	-5.52, -0.69	-4.83, -0.69	
		24 週	n	65	62	
			Mean±SD	-2.30±1.11	-2.55±1.05	0.19

			Median [Q1, Q3]	-2.38 [-3.02, -1.51]	-2.66 [-3.19, -1.74]	0.16
			Min, Max	-5.12, -0.69	-4.42, -0.69	
	変化量	12 週	n	63	64	
			Mean±SD	0.09±0.98	0.19±0.99	0.56
			Median [Q1, Q3]	0.01 [-0.54, 0.74]	0.22 [-0.44, 0.78]	0.53
			Min, Max	-2.32, 3.52	-2.47, 2.92	
			One-sample t-test	0.48	0.12	
			Wilcoxon signed-rank test	0.46	0.12	
		24 週	n	64	61	
			Mean±SD	0.32±1.02	0.16±0.80	0.35
			Median [Q1, Q3]	0.24 [-0.15, 0.87]	0.15 [-0.21, 0.63]	0.38
			Min, Max	-2.80, 3.73	-2.31, 1.79	
			One-sample t-test	0.015	0.11	
			Wilcoxon signed-rank test	0.006	0.048	
	変化率	12 週	n	63	64	
			Mean±SD	13.8±73.1	5.1±59.6	0.46
			Median [Q1, Q3]	-0.4 [-25.0, 17.2]	-7.0 [-27.2, 18.0]	0.63
			Min, Max	-79.5, 303.5	-80.8, 236.2	
			One-sample t-test	0.14	0.50	
			Wilcoxon signed-rank test	0.84	0.46	
		24 週	n	64	61	
			Mean±SD	1.8±71.4	3.5±54.7	0.88
			Median [Q1, Q3]	-8.1 [-26.7, 6.8]	-4.1 [-17.9, 5.2]	0.35
			Min, Max	-84.3, 378.3	-71.2, 263.2	
			One-sample t-test	0.84	0.61	
			Wilcoxon signed-rank test	0.023	0.18	
シスタチンC (mg/L)	測定値	0 週	n	70	70	
			Mean±SD	1.18±0.27	1.17±0.32	0.86
			Median [Q1, Q3]	1.17 [0.97, 1.34]	1.09 [0.93, 1.33]	0.62
			Min, Max	0.70, 2.02	0.69, 2.08	
		12 週	n	65	68	
			Mean±SD	1.20±0.31	1.16±0.35	0.52
			Median [Q1, Q3]	1.19 [0.98, 1.32]	1.11 [0.92, 1.30]	0.31
			Min, Max	0.70, 2.03	0.63, 2.41	
		24 週	n	66	66	
			Mean±SD	1.21±0.33	1.21±0.39	0.96
			Median [Q1, Q3]	1.17 [0.98, 1.36]	1.14 [0.91, 1.36]	0.63
			Min, Max	0.73, 2.26	0.62, 2.34	
	変化量	12 週	n	65	68	
			Mean±SD	0.04±0.15	0.01±0.15	0.28
			Median [Q1, Q3]	0.03 [-0.02, 0.08]	-0.01 [-0.06, 0.09]	0.23
			Min, Max	-0.28, 0.55	-0.60, 0.49	
			One-sample t-test	0.035	0.57	
			Wilcoxon signed-rank test	0.031	0.58	
		24 週	n	66	66	
			Mean±SD	0.05±0.18	0.05±0.17	0.88
			Median [Q1, Q3]	0.03 [-0.03, 0.13]	0.04 [-0.04, 0.12]	0.87
			Min, Max	-0.32, 0.65	-0.38, 1.03	
			One-sample t-test	0.041	0.022	

			Wilcoxon signed-rank test	0.019	0.008	
	変化率	12 週	n	65	68	
			Mean±SD	3.3±10.9	1.3±12.4	0.31
			Median [Q1, Q3]	2.8 [-1.7, 7.4]	-0.9 [-5.4, 8.2]	0.24
			Min, Max	-16.0, 37.2	-42.3, 41.4	
			One-sample t-test	0.016	0.40	
			Wilcoxon signed-rank test	0.020	0.50	
		24 週	n	66	66	
			Mean±SD	3.8±12.9	4.1±13.8	0.92
			Median [Q1, Q3]	3.5 [-2.6, 10.0]	3.8 [-3.9, 12.1]	0.90
			Min, Max	-27.0, 40.4	-24.5, 78.6	
			One-sample t-test	0.018	0.019	
			Wilcoxon signed-rank test	0.015	0.011	
MDA-LDL (U/L)	測定値	0 週	n	70	70	
			Mean±SD	95.6±31.8	96.2±29.5	0.90
			Median [Q1, Q3]	96.5 [73.0, 114.0]	96.0 [71.0, 114.0]	0.98
			Min, Max	30.0, 167.0	51.0, 170.0	
		12 週	n	65	68	
			Mean±SD	91.0±34.4	85.8±27.3	0.33
			Median [Q1, Q3]	88.0 [67.0, 108.0]	80.5 [67.5, 102.5]	0.54
			Min, Max	30.0, 188.0	31.0, 182.0	
		24 週	n	66	66	
			Mean±SD	91.3±33.1	87.4±32.5	0.49
			Median [Q1, Q3]	87.0 [69.0, 107.0]	80.5 [64.0, 103.0]	0.34
			Min, Max	36.0, 196.0	35.0, 190.0	
	変化量	12 週	n	65	68	
			Mean±SD	-4.8±27.0	-10.7±25.9	0.20
			Median [Q1, Q3]	-7.0 [-21.0, 10.0]	-7.0 [-29.0, 7.0]	0.32
			Min, Max	-72.0, 74.0	-67.0, 46.0	
			One-sample t-test	0.16	0.001	
			Wilcoxon signed-rank test	0.07	0.003	
		24 週	n	66	66	
			Mean±SD	-4.6±26.9	-8.1±24.1	0.43
			Median [Q1, Q3]	-4.5 [-19.0, 15.0]	-8.0 [-24.0, 3.0]	0.30
			Min, Max	-71.0, 54.0	-70.0, 77.0	
			One-sample t-test	0.17	0.008	
			Wilcoxon signed-rank test	0.33	0.003	
	変化率	12 週	n	65	68	
			Mean±SD	-1.3±30.2	-7.8±26.3	0.19
			Median [Q1, Q3]	-6.4 [-20.9, 12.7]	-7.8 [-29.7, 7.7]	0.27
			Min, Max	-46.2, 119.5	-47.5, 54.9	
			One-sample t-test	0.73	0.017	
			Wilcoxon signed-rank test	0.18	0.012	
		24 週	n	66	66	
			Mean±SD	0.0±31.7	-6.8±23.4	0.16
			Median [Q1, Q3]	-3.8 [-21.6, 19.7]	-7.4 [-20.0, 5.2]	0.30
			Min, Max	-48.3, 131.7	-56.5, 73.3	
			One-sample t-test	0.99	0.021	
			Wilcoxon signed-rank test	0.61	0.005	

5. 副次評価項目

5.1. FAS

特殊血液検査

XOR 活性 (pmol/h/mL plasma)	測定値	0 週	n	70	70	
			Mean±SD	55.3±53.3	66.9±125.6	0.48
			Median [Q1, Q3]	33.8 [25.5, 61.5]	29.2 [18.1, 57.5]	0.12
			Min, Max	8.0, 342.0	6.7, 946.0	
		24 週	n	66	66	
			Mean±SD	23.1±33.6	29.5±45.0	0.36
			Median [Q1, Q3]	11.5 [6.7, 27.4]	12.7 [6.8, 24.4]	0.57
			Min, Max	6.7, 250.0	6.7, 246.0	
	変化量	24 週	n	66	66	
			Mean±SD	-33.2±57.2	-40.6±114.4	0.64
			Median [Q1, Q3]	-21.8 [-43.4, -10.2]	-18.0 [-32.2, -8.2]	0.35
			Min, Max	-334.9, 182.2	-868.1, 138.0	
			One-sample t-test	<0.001	0.005	
			Wilcoxon signed-rank test	<0.001	<0.001	
	変化率	24 週	n	66	66	
			Mean±SD	-49.6±54.5	-44.1±45.5	0.53
			Median [Q1, Q3]	-65.7 [-78.8, -48.1]	-55.0 [-70.7, -36.4]	0.05
			Min, Max	-97.9, 268.7	-91.8, 130.0	
			One-sample t-test	<0.001	<0.001	
			Wilcoxon signed-rank test	<0.001	<0.001	
対数変換 XOR 活性(ln (pmol/h/mL plasma))	測定値	0 週	n	70	70	
			Mean±SD	3.7±0.7	3.6±1.0	0.28
			Median [Q1, Q3]	3.5 [3.2, 4.1]	3.4 [2.9, 4.1]	0.12
			Min, Max	2.1, 5.8	1.9, 6.9	
		24 週	n	66	66	
			Mean±SD	2.7±0.8	2.8±0.9	0.48
			Median [Q1, Q3]	2.4 [1.9, 3.3]	2.5 [1.9, 3.2]	0.57
			Min, Max	1.9, 5.5	1.9, 5.5	
	変化量	24 週	n	66	66	
			Mean±SD	-1.0±0.8	-0.8±0.7	0.10
			Median [Q1, Q3]	-1.1 [-1.5, -0.7]	-0.8 [-1.2, -0.5]	0.05
			Min, Max	-3.9, 1.3	-2.5, 0.8	
			One-sample t-test	<0.001	<0.001	
			Wilcoxon signed-rank test	<0.001	<0.001	
	変化率	24 週	n	66	66	
			Mean±SD	-27.1±20.4	-21.9±17.6	0.12
			Median [Q1, Q3]	-31.3 [-43.6, -17.1]	-23.5 [-34.5, -12.3]	0.06
			Min, Max	-66.3, 30.9	-55.8, 26.6	
			One-sample t-test	<0.001	<0.001	
			Wilcoxon signed-rank test	<0.001	<0.001	

5. 副次評価項目
5.1. FAS
特殊尿検査

表 5.1.11. [FAS] 特殊尿検査

変数		観察ポイント	統計量	トピロキソスタット群	アロプリノール群	群間比較 P 値
8-OHdG (ng/mg・Cr)	測定値	0 週	n	70	70	
			Mean±SD	7.7±3.5	7.2±4.0	0.47
			Median [Q1, Q3]	6.9 [5.4, 8.8]	6.6 [4.8, 9.0]	0.25
			Min, Max	2.9, 22.3	2.1, 27.8	
		12 週	n	65	68	
			Mean±SD	8.9±3.7	10.1±5.4	0.16
			Median [Q1, Q3]	8.2 [6.5, 10.3]	9.1 [7.0, 11.6]	0.21
			Min, Max	3.6, 20.7	4.3, 37.9	
		24 週	n	66	66	
			Mean±SD	8.6±4.1	10.2±4.5	0.031
			Median [Q1, Q3]	8.3 [5.8, 9.6]	9.3 [7.6, 11.8]	0.009
			Min, Max	1.5, 24.9	3.6, 29.0	
	変化量	12 週	n	65	68	
			Mean±SD	1.1±3.4	2.8±4.6	0.021
			Median [Q1, Q3]	1.1 [-0.4, 2.6]	2.6 [0.3, 4.6]	0.007
			Min, Max	-7.4, 12.7	-10.1, 27.3	
			One-sample t-test	0.011	<0.001	
			Wilcoxon signed-rank test	0.002	<0.001	
		24 週	n	66	66	
			Mean±SD	1.0±3.6	3.0±3.1	<0.001
			Median [Q1, Q3]	1.2 [-0.7, 2.6]	3.0 [1.0, 4.7]	<0.001
			Min, Max	-9.9, 11.9	-2.3, 10.6	
			One-sample t-test	0.028	<0.001	
			Wilcoxon signed-rank test	0.010	<0.001	
	変化率	12 週	n	65	68	
			Mean±SD	24.1±46.5	52.2±66.4	0.006
			Median [Q1, Q3]	18.0 [-4.1, 40.7]	45.3 [3.6, 80.6]	0.010
			Min, Max	-61.7, 158.8	-43.0, 275.0	
			One-sample t-test	<0.001	<0.001	
			Wilcoxon signed-rank test	<0.001	<0.001	
		24 週	n	66	66	
			Mean±SD	22.9±52.8	58.7±66.6	<0.001
			Median [Q1, Q3]	15.8 [-10.1, 39.8]	41.8 [12.7, 89.6]	<0.001
			Min, Max	-83.0, 197.6	-25.0, 296.4	
			One-sample t-test	<0.001	<0.001	
			Wilcoxon signed-rank test	<0.001	<0.001	
尿中 L-FABP (濃度) (ng/mL)	測定値	0 週	n	70	70	
			Mean±SD	3.9±3.9	5.1±7.0	0.19
			Median [Q1, Q3]	2.3 [1.5, 3.7]	2.4 [1.6, 5.3]	0.36
			Min, Max	1.5, 21.5	1.5, 36.3	
		12 週	n	65	68	
			Mean±SD	3.5±3.1	4.8±7.5	0.22
			Median [Q1, Q3]	2.0 [1.5, 4.0]	2.2 [1.5, 5.0]	0.42
			Min, Max	1.5, 12.8	1.5, 48.8	
		24 週	n	66	66	
			Mean±SD	4.1±4.9	5.3±8.1	0.35

			Median [Q1, Q3]	2.4 [1.5, 5.0]	2.1 [1.5, 6.6]	0.85
			Min, Max	1.5, 33.3	1.5, 51.8	
	変化量	12 週	n	65	68	
			Mean±SD	-0.3±3.7	0.1±7.0	0.68
			Median [Q1, Q3]	0.0 [-0.7, 0.5]	0.0 [-1.1, 0.9]	0.88
			Min, Max	-13.4, 10.5	-26.5, 32.6	
			One-sample t-test	0.46	0.95	
			Wilcoxon signed-rank test	0.89	0.76	
		24 週	n	66	66	
			Mean±SD	0.7±4.3	0.3±7.0	0.74
			Median [Q1, Q3]	0.1 [-0.6, 1.2]	0.0 [-0.9, 0.9]	0.48
			Min, Max	-9.8, 21.9	-28.3, 33.4	
			One-sample t-test	0.22	0.71	
			Wilcoxon signed-rank test	0.22	0.81	
	変化率	12 週	n	65	68	
			Mean±SD	20.6±98.4	28.3±118.0	0.69
			Median [Q1, Q3]	0.0 [-22.2, 33.3]	0.0 [-29.3, 35.8]	0.93
			Min, Max	-89.9, 552.6	-86.7, 581.3	
			One-sample t-test	0.10	0.05	
			Wilcoxon signed-rank test	0.42	0.56	
		24 週	n	66	66	
			Mean±SD	38.7±106.8	52.1±249.9	0.69
			Median [Q1, Q3]	2.2 [-20.7, 60.5]	0.0 [-30.9, 40.0]	0.35
			Min, Max	-81.9, 416.7	-85.2, 1855.6	
			One-sample t-test	0.004	0.10	
			Wilcoxon signed-rank test	0.037	0.44	
対数変換 尿中 L-FABP(濃度)(ln(ng/mL))	測定値	0 週	n	70	70	
			Mean±SD	1.03±0.72	1.17±0.84	0.30
			Median [Q1, Q3]	0.83 [0.41, 1.31]	0.85 [0.47, 1.67]	0.36
			Min, Max	0.41, 3.07	0.41, 3.59	
		12 週	n	65	68	
			Mean±SD	0.98±0.69	1.09±0.82	0.39
			Median [Q1, Q3]	0.69 [0.41, 1.39]	0.79 [0.41, 1.60]	0.42
			Min, Max	0.41, 2.55	0.41, 3.89	
		24 週	n	66	66	
			Mean±SD	1.09±0.73	1.13±0.89	0.74
			Median [Q1, Q3]	0.88 [0.41, 1.61]	0.72 [0.41, 1.89]	0.85
			Min, Max	0.41, 3.51	0.41, 3.95	
	変化量	12 週	n	65	68	
			Mean±SD	-0.04±0.71	-0.04±0.76	0.98
			Median [Q1, Q3]	0.00 [-0.25, 0.29]	0.00 [-0.35, 0.31]	0.93
			Min, Max	-2.30, 1.88	-2.02, 1.92	
			One-sample t-test	0.63	0.67	
			Wilcoxon signed-rank test	0.95	0.80	
		24 週	n	66	66	
			Mean±SD	0.10±0.68	0.01±0.79	0.49
			Median [Q1, Q3]	0.02 [-0.23, 0.47]	0.00 [-0.37, 0.34]	0.35
			Min, Max	-1.71, 1.64	-1.91, 2.97	
			One-sample t-test	0.23	0.90	

			Wilcoxon signed-rank test	0.19	0.91	
	変化率	12 週	n	65	68	
			Mean±SD	14.9±74.9	16.0±74.8	0.93
			Median [Q1, Q3]	0.0 [-25.3, 48.9]	0.0 [-31.9, 46.2]	0.97
			Min, Max	-85.0, 339.8	-82.5, 260.0	
			One-sample t-test	0.11	0.08	
			Wilcoxon signed-rank test	0.30	0.43	
		24 週	n	66	66	
			Mean±SD	32.0±89.3	24.2±104.8	0.65
			Median [Q1, Q3]	2.6 [-21.8, 49.1]	0.0 [-29.6, 36.4]	0.29
			Min, Max	-80.8, 396.9	-82.5, 505.8	
			One-sample t-test	0.005	0.07	
			Wilcoxon signed-rank test	0.032	0.52	
尿中 L-FABP (Cre 補正値) (μg/g･Cr)	測定値	0 週	n	70	70	
			Mean±SD	5.1±5.0	5.2±6.0	0.95
			Median [Q1, Q3]	3.1 [1.9, 6.2]	3.3 [1.9, 5.7]	0.94
			Min, Max	0.8, 25.1	0.8, 33.4	
		12 週	n	65	68	
			Mean±SD	4.6±5.7	5.9±7.2	0.24
			Median [Q1, Q3]	2.6 [2.0, 4.8]	3.5 [2.3, 6.8]	0.06
			Min, Max	0.6, 39.1	1.2, 47.2	
		24 週	n	66	66	
			Mean±SD	5.9±9.4	6.4±7.0	0.73
			Median [Q1, Q3]	3.0 [1.8, 5.7]	3.5 [2.2, 8.6]	0.19
			Min, Max	0.8, 55.7	0.9, 37.5	
	変化量	12 週	n	65	68	
			Mean±SD	-0.3±5.0	1.1±4.2	0.09
			Median [Q1, Q3]	0.0 [-0.7, 0.8]	0.3 [-0.8, 2.1]	0.11
			Min, Max	-17.7, 20.5	-8.7, 18.8	
			One-sample t-test	0.68	0.035	
			Wilcoxon signed-rank test	0.78	0.07	
		24 週	n	66	66	
			Mean±SD	1.1±7.7	1.3±5.5	0.92
			Median [Q1, Q3]	-0.1 [-1.3, 1.0]	0.4 [-0.8, 2.2]	0.32
			Min, Max	-16.7, 38.0	-15.8, 29.6	
			One-sample t-test	0.23	0.07	
			Wilcoxon signed-rank test	0.98	0.09	
	変化率	12 週	n	65	68	
			Mean±SD	11.8±80.4	43.9±107.4	0.05
			Median [Q1, Q3]	0.0 [-24.6, 30.8]	9.3 [-21.9, 68.8]	0.08
			Min, Max	-84.7, 490.0	-65.8, 494.7	
			One-sample t-test	0.24	0.001	
			Wilcoxon signed-rank test	0.81	0.005	
		24 週	n	66	66	
			Mean±SD	33.0±133.3	72.8±231.2	0.23
			Median [Q1, Q3]	-5.3 [-31.3, 50.0]	10.9 [-17.9, 70.5]	0.14
			Min, Max	-79.9, 866.7	-77.4, 1418.2	
			One-sample t-test	0.049	0.013	
			Wilcoxon signed-rank test	0.41	0.003	

対数変換 尿中L-FABP(Cre 補正値)(ln(μg/g・Cr))	測定値	0 週	n	70	70	
			Mean±SD	1.28±0.82	1.28±0.80	0.96
			Median [Q1, Q3]	1.13 [0.64, 1.82]	1.19 [0.64, 1.74]	0.94
			Min, Max	-0.22, 3.22	-0.22, 3.51	
		12 週	n	65	68	
			Mean±SD	1.15±0.77	1.40±0.80	0.07
			Median [Q1, Q3]	0.96 [0.69, 1.57]	1.25 [0.81, 1.92]	0.06
			Min, Max	-0.51, 3.67	0.18, 3.85	
		24 週	n	66	66	
			Mean±SD	1.26±0.90	1.45±0.87	0.22
			Median [Q1, Q3]	1.10 [0.59, 1.74]	1.24 [0.79, 2.15]	0.19
			Min, Max	-0.22, 4.02	-0.11, 3.62	
	変化量	12 週	n	65	68	
			Mean±SD	-0.08±0.65	0.16±0.61	0.026
			Median [Q1, Q3]	0.00 [-0.28, 0.27]	0.09 [-0.25, 0.52]	0.08
			Min, Max	-1.88, 1.77	-1.07, 1.78	
			One-sample t-test	0.31	0.029	
			Wilcoxon signed-rank test	0.69	0.045	
		24 週	n	66	66	
			Mean±SD	0.02±0.68	0.20±0.74	0.17
			Median [Q1, Q3]	-0.05 [-0.37, 0.41]	0.10 [-0.20, 0.53]	0.14
			Min, Max	-1.60, 2.27	-1.49, 2.72	
			One-sample t-test	0.78	0.036	
			Wilcoxon signed-rank test	0.95	0.05	
	変化率	12 週	n	65	68	
			Mean±SD	31.6±148.8	60.6±251.1	0.42
			Median [Q1, Q3]	0.0 [-24.0, 26.3]	5.8 [-19.2, 48.4]	0.30
			Min, Max	-84.7, 902.5	-349.0, 1454.5	
			One-sample t-test	0.09	0.05	
			Wilcoxon signed-rank test	0.75	0.08	
		24 週	n	66	66	
			Mean±SD	30.8±179.5	48.2±445.0	0.77
			Median [Q1, Q3]	-5.7 [-36.8, 37.5]	8.1 [-19.1, 49.4]	0.24
			Min, Max	-181.7, 1244.3	-1173.8, 2853.9	
			One-sample t-test	0.17	0.38	
			Wilcoxon signed-rank test	0.97	0.09	
尿中アルブミン(アルブミン)(μg/mL)	測定値	0 週	n	70	70	
			Mean±SD	97.3±245.9	166.8±661.0	0.41
			Median [Q1, Q3]	13.8 [5.5, 50.5]	20.7 [7.3, 67.2]	0.26
			Min, Max	0.5, 1402.6	0.6, 4566.9	
		12 週	n	65	69	
			Mean±SD	104.4±337.7	114.0±272.5	0.86
			Median [Q1, Q3]	9.4 [4.5, 37.4]	17.4 [6.6, 55.8]	0.15
			Min, Max	0.5, 2209.6	0.6, 1628.5	
		24 週	n	66	66	
			Mean±SD	68.0±175.6	145.4±696.2	0.38
			Median [Q1, Q3]	13.0 [5.6, 48.7]	16.3 [4.3, 40.7]	0.68
			Min, Max	0.7, 1024.8	0.8, 5627.4	
	変化量	12 週	n	65	69	

			Mean±SD	2.3±292.2	-54.7±601.7	0.49
			Median [Q1, Q3]	-0.2 [-5.5, 3.4]	-3.0 [-14.1, 6.8]	0.46
			Min, Max	-1102.5, 1630.7	-4135.9, 1401.0	
			One-sample t-test	0.95	0.45	
			Wilcoxon signed-rank test	0.49	0.21	
		24 週	n	66	66	
			Mean±SD	-24.6±229.3	-26.1±621.0	0.99
			Median [Q1, Q3]	0.1 [-7.7, 6.4]	-2.0 [-11.1, 4.7]	0.48
			Min, Max	-1079.0, 720.9	-4202.7, 2500.1	
			One-sample t-test	0.39	0.73	
			Wilcoxon signed-rank test	0.78	0.19	
	変化率	12 週	n	65	69	
			Mean±SD	47.6±221.9	105.1±364.1	0.28
			Median [Q1, Q3]	-1.2 [-40.0, 59.5]	-14.5 [-66.0, 86.0]	0.93
			Min, Max	-99.4, 1565.5	-95.7, 2425.0	
			One-sample t-test	0.09	0.019	
			Wilcoxon signed-rank test	0.51	0.61	
		24 週	n	66	66	
			Mean±SD	71.8±232.5	71.0±269.0	0.99
			Median [Q1, Q3]	3.6 [-49.1, 58.7]	-14.7 [-61.9, 48.5]	0.39
			Min, Max	-93.4, 1118.6	-92.0, 1366.7	
			One-sample t-test	0.015	0.036	
			Wilcoxon signed-rank test	0.36	0.75	
対数変換 尿中アルブミン(アルブミン)(ln(μg/mL))	測定値	0 週	n	70	70	
			Mean±SD	2.84±1.77	3.11±1.76	0.37
			Median [Q1, Q3]	2.62 [1.70, 3.92]	3.03 [1.99, 4.21]	0.26
			Min, Max	-0.69, 7.25	-0.51, 8.43	
		12 週	n	65	69	
			Mean±SD	2.63±1.76	3.00±1.88	0.24
			Median [Q1, Q3]	2.24 [1.50, 3.62]	2.86 [1.89, 4.02]	0.15
			Min, Max	-0.69, 7.70	-0.51, 7.40	
		24 週	n	66	66	
			Mean±SD	2.74±1.63	2.89±1.72	0.60
			Median [Q1, Q3]	2.56 [1.72, 3.89]	2.79 [1.46, 3.71]	0.68
			Min, Max	-0.36, 6.93	-0.22, 8.64	
	変化量	12 週	n	65	69	
			Mean±SD	-0.19±1.22	-0.10±1.27	0.68
			Median [Q1, Q3]	-0.01 [-0.51, 0.47]	-0.16 [-1.08, 0.62]	0.93
			Min, Max	-5.11, 2.81	-3.15, 3.23	
			One-sample t-test	0.21	0.50	
			Wilcoxon signed-rank test	0.39	0.39	
		24 週	n	66	66	
			Mean±SD	-0.05±1.10	-0.14±1.09	0.63
			Median [Q1, Q3]	0.03 [-0.68, 0.46]	-0.16 [-0.96, 0.40]	0.39
			Min, Max	-2.72, 2.50	-2.53, 2.69	
			One-sample t-test	0.72	0.31	
			Wilcoxon signed-rank test	0.74	0.14	
	変化率	12 週	n	64	69	
			Mean±SD	8.7±71.4	8.2±247.8	0.99

			Median [Q1, Q3]	-1.2 [-22.5, 21.1]	-6.1 [-37.7, 17.4]	0.45
			Min, Max	-83.9, 306.9	-848.3, 1771.0	
			One-sample t-test	0.33	0.78	
			Wilcoxon signed-rank test	0.76	0.23	
		24 週	n	65	66	
			Mean±SD	21.2±139.9	-7.7±328.3	0.51
			Median [Q1, Q3]	-1.8 [-27.4, 24.7]	-6.1 [-29.8, 10.9]	0.32
			Min, Max	-95.0, 1054.1	-2226.7, 1294.0	
			One-sample t-test	0.23	0.85	
			Wilcoxon signed-rank test	0.98	0.11	
尿中アルブミン(クレアチニン)(mg/dL)	測定値	0 週	n	70	70	
			Mean±SD	97.6±57.5	107.3±56.6	0.32
			Median [Q1, Q3]	86.5 [52.9, 139.7]	98.6 [64.8, 151.4]	0.29
			Min, Max	7.2, 251.0	14.8, 273.2	
		12 週	n	65	69	
			Mean±SD	102.3±62.2	94.9±67.7	0.51
			Median [Q1, Q3]	87.3 [57.1, 134.4]	74.3 [42.8, 122.8]	0.31
			Min, Max	11.0, 329.8	6.7, 358.9	
		24 週	n	66	66	
			Mean±SD	110.3±97.2	87.4±49.0	0.09
			Median [Q1, Q3]	86.4 [55.4, 141.8]	83.6 [54.6, 120.4]	0.32
			Min, Max	13.8, 563.6	16.6, 283.0	
	変化量	12 週	n	65	69	
			Mean±SD	3.6±71.8	-13.3±71.2	0.17
			Median [Q1, Q3]	6.7 [-29.7, 47.3]	-7.5 [-52.3, 33.0]	0.16
			Min, Max	-193.9, 276.8	-204.5, 174.8	
			One-sample t-test	0.69	0.13	
			Wilcoxon signed-rank test	0.47	0.17	
		24 週	n	66	66	
			Mean±SD	13.1±88.1	-17.3±58.4	0.021
			Median [Q1, Q3]	3.7 [-24.8, 38.4]	-12.2 [-51.5, 11.9]	0.041
			Min, Max	-174.6, 387.5	-204.8, 131.0	
			One-sample t-test	0.23	0.019	
			Wilcoxon signed-rank test	0.48	0.027	
	変化率	12 週	n	65	69	
			Mean±SD	47.2±145.8	6.1±76.8	0.041
			Median [Q1, Q3]	7.6 [-35.0, 47.4]	-7.3 [-47.2, 38.1]	0.12
			Min, Max	-93.8, 553.3	-91.3, 320.3	
			One-sample t-test	0.011	0.51	
			Wilcoxon signed-rank test	0.12	0.73	
		24 週	n	66	66	
			Mean±SD	42.0±111.8	5.2±89.6	0.039
			Median [Q1, Q3]	5.1 [-24.9, 71.7]	-16.6 [-39.0, 20.7]	0.024
			Min, Max	-86.1, 492.5	-92.3, 519.8	
			One-sample t-test	0.003	0.64	
			Wilcoxon signed-rank test	0.045	0.15	
対数変換 尿中アルブミン(クレアチニン)(ln	測定値	0 週	n	70	70	
			Mean±SD	4.35±0.77	4.50±0.65	0.22
			Median [Q1, Q3]	4.46 [3.97, 4.94]	4.59 [4.17, 5.02]	0.29

(mg/dL))			Min, Max	1.97, 5.53	2.69, 5.61	
		12 週	n	65	69	
			Mean±SD	4.43±0.68	4.28±0.80	0.25
			Median [Q1, Q3]	4.47 [4.04, 4.90]	4.31 [3.76, 4.81]	0.31
			Min, Max	2.39, 5.80	1.89, 5.88	
		24 週	n	66	66	
			Mean±SD	4.44±0.73	4.30±0.64	0.24
			Median [Q1, Q3]	4.46 [4.01, 4.95]	4.43 [4.00, 4.79]	0.32
			Min, Max	2.62, 6.33	2.81, 5.65	
	変化量	12 週	n	65	69	
			Mean±SD	0.04±0.84	-0.23±0.84	0.06
			Median [Q1, Q3]	0.07 [-0.43, 0.39]	-0.08 [-0.64, 0.32]	0.12
			Min, Max	-2.78, 1.88	-2.44, 1.44	
			One-sample t-test	0.67	0.027	
			Wilcoxon signed-rank test	0.52	0.10	
		24 週	n	66	66	
			Mean±SD	0.08±0.76	-0.18±0.69	0.037
			Median [Q1, Q3]	0.05 [-0.29, 0.54]	-0.18 [-0.49, 0.19]	0.024
			Min, Max	-1.97, 1.78	-2.57, 1.82	
			One-sample t-test	0.37	0.037	
			Wilcoxon signed-rank test	0.30	0.024	
	変化率	12 週	n	65	69	
			Mean±SD	3.7±23.2	-3.8±18.8	0.040
			Median [Q1, Q3]	1.6 [-8.7, 9.1]	-1.7 [-16.1, 7.0]	0.12
			Min, Max	-53.7, 95.2	-56.3, 45.7	
			One-sample t-test	0.20	0.09	
			Wilcoxon signed-rank test	0.43	0.16	
		24 週	n	66	66	
			Mean±SD	4.2±20.7	-2.7±16.3	0.033
			Median [Q1, Q3]	1.1 [-6.5, 13.9]	-4.2 [-11.9, 4.4]	0.018
			Min, Max	-39.0, 81.3	-47.6, 58.1	
			One-sample t-test	0.10	0.18	
			Wilcoxon signed-rank test	0.17	0.041	
尿中アルブミン(クレアチニン補正値) (mg/g Cr)	測定値	0 週	n	69	70	
			Mean±SD	129.5±348.2	156.0±550.7	0.74
			Median [Q1, Q3]	14.3 [6.0, 79.6]	19.7 [6.9, 57.2]	0.56
			Min, Max	1.6, 2366.9	1.9, 3560.1	
		12 週	n	65	69	
			Mean±SD	108.1±390.7	166.0±559.4	0.49
			Median [Q1, Q3]	12.8 [4.9, 40.0]	19.6 [9.3, 59.7]	0.045
			Min, Max	1.3, 2920.0	1.6, 4259.3	
		24 週	n	66	66	
			Mean±SD	107.6±344.5	155.2±492.3	0.52
			Median [Q1, Q3]	14.9 [5.1, 38.3]	19.9 [6.2, 54.8]	0.26
			Min, Max	2.0, 2351.7	2.0, 3452.6	
	変化量	12 週	n	64	69	
			Mean±SD	-22.5±123.0	9.0±316.4	0.46
			Median [Q1, Q3]	-1.2 [-23.8, 3.2]	-0.5 [-7.0, 16.9]	0.13
			Min, Max	-567.9, 553.1	-2030.1, 1410.1	

			One-sample t-test	0.15	0.81	
			Wilcoxon signed-rank test	0.06	0.54	
		24 週	n	65	66	
			Mean±SD	-21.0±106.4	-6.3±270.7	0.68
			Median [Q1, Q3]	0.1 [-8.3, 3.5]	-0.8 [-10.9, 13.6]	0.71
			Min, Max	-552.6, 177.9	-1843.0, 652.1	
			One-sample t-test	0.12	0.85	
			Wilcoxon signed-rank test	0.49	0.91	
	変化率	12 週	n	64	69	
			Mean±SD	8.4±88.3	71.8±183.0	0.013
			Median [Q1, Q3]	-14.7 [-40.8, 26.5]	-5.0 [-38.6, 89.0]	0.09
			Min, Max	-92.6, 326.7	-79.8, 784.2	
			One-sample t-test	0.45	0.002	
			Wilcoxon signed-rank test	0.34	0.16	
		24 週	n	65	66	
			Mean±SD	21.8±115.7	107.4±363.4	0.07
			Median [Q1, Q3]	2.0 [-47.6, 25.3]	-9.9 [-46.5, 52.4]	0.71
			Min, Max	-93.4, 509.6	-88.4, 1790.8	
			One-sample t-test	0.13	0.019	
			Wilcoxon signed-rank test	0.76	0.70	
対数変換 尿中アルブミン(クレアチニン補正値)(ln(mg/g Cr))	測定値	0 週	n	69	70	
			Mean±SD	3.13±1.73	3.22±1.63	0.76
			Median [Q1, Q3]	2.66 [1.79, 4.38]	2.98 [1.93, 4.05]	0.56
			Min, Max	0.47, 7.77	0.64, 8.18	
		12 週	n	65	69	
			Mean±SD	2.80±1.63	3.32±1.68	0.07
			Median [Q1, Q3]	2.55 [1.59, 3.69]	2.98 [2.23, 4.09]	0.045
			Min, Max	0.26, 7.98	0.47, 8.36	
		24 週	n	66	66	
			Mean±SD	2.91±1.61	3.20±1.70	0.31
			Median [Q1, Q3]	2.70 [1.63, 3.65]	2.99 [1.82, 4.00]	0.26
			Min, Max	0.69, 7.76	0.69, 8.15	
	変化量	12 週	n	64	69	
			Mean±SD	-0.24±0.89	0.12±0.89	0.019
			Median [Q1, Q3]	-0.16 [-0.52, 0.23]	-0.05 [-0.49, 0.64]	0.09
			Min, Max	-2.61, 1.45	-1.60, 2.18	
			One-sample t-test	0.032	0.25	
			Wilcoxon signed-rank test	0.06	0.57	
		24 週	n	65	66	
			Mean±SD	-0.13±0.85	0.04±1.03	0.29
			Median [Q1, Q3]	0.02 [-0.65, 0.23]	-0.10 [-0.63, 0.42]	0.71
			Min, Max	-2.71, 1.81	-2.16, 2.94	
			One-sample t-test	0.21	0.75	
			Wilcoxon signed-rank test	0.26	0.63	
	変化率	12 週	n	64	69	
			Mean±SD	-4.0±33.3	11.0±47.2	0.037
			Median [Q1, Q3]	-6.7 [-23.8, 9.6]	-2.0 [-15.3, 23.7]	0.09
			Min, Max	-68.5, 132.1	-71.4, 196.2	
			One-sample t-test	0.34	0.06	

			Wilcoxon signed-rank test	0.10	0.48	
		24 週	n	65	66	
			Mean±SD	1.3±30.0	11.1±59.2	0.24
			Median [Q1, Q3]	0.7 [-22.1, 15.8]	-2.8 [-16.3, 16.8]	0.91
			Min, Max	-50.3, 98.6	-63.0, 254.4	
			One-sample t-test	0.73	0.13	
			Wilcoxon signed-rank test	0.66	0.80	

5.2. PPS

表 5.2.1. [PPS] 24 週変化率以外も含めた NT-proBNP

変数		観察ポイント	統計量	トピロキソスタット群	アロプリノール群	群間比較P値
対数変換 NT-proBNP (ln(pg/mL))	測定値	0 週	n	64	67	
			Mean±SD	6.49±1.00	6.34±0.90	0.36
			Median [Q1, Q3]	6.43 [5.74, 7.20]	6.30 [5.72, 7.06]	0.44
			Min, Max	4.68, 10.22	4.58, 8.77	
		12 週	n	61	65	
			Mean±SD	6.49±0.92	6.28±1.04	0.24
			Median [Q1, Q3]	6.43 [5.81, 7.19]	6.45 [5.49, 6.97]	0.34
			Min, Max	4.62, 9.17	4.35, 9.53	
		24 週	n	61	63	
			Mean±SD	6.48±0.87	6.31±1.01	0.33
			Median [Q1, Q3]	6.36 [5.90, 7.14]	6.18 [5.52, 7.01]	0.24
			Min, Max	4.70, 8.39	4.62, 9.36	
	変化量	12 週	n	61	65	
			Mean±SD	0.03±0.43	-0.01±0.46	0.64
			Median [Q1, Q3]	-0.03 [-0.20, 0.34]	-0.02 [-0.30, 0.30]	0.49
			Min, Max	-1.05, 1.31	-1.10, 1.91	
			One-sample t-test	0.59	0.89	
			Wilcoxon signed-rank test	0.63	0.71	
		24 週	n	61	63	
			Mean±SD	0.06±0.46	0.00±0.49	0.50
			Median [Q1, Q3]	0.02 [-0.17, 0.24]	0.03 [-0.24, 0.31]	0.78
			Min, Max	-0.87, 1.53	-1.77, 1.06	
			One-sample t-test	0.32	0.99	
			Wilcoxon signed-rank test	0.44	0.66	
	変化率	12 週	n	61	65	
			Mean±SD	0.9±7.0	-0.3±7.3	0.36
			Median [Q1, Q3]	-0.4 [-3.5, 5.2]	-0.3 [-4.8, 4.8]	0.43
			Min, Max	-14.3, 24.2	-17.3, 25.2	
			One-sample t-test	0.34	0.74	
			Wilcoxon signed-rank test	0.55	0.72	
		24 週	n	61	63	
			Mean±SD	1.3±8.1	0.1±7.6	0.39
			Median [Q1, Q3]	0.3 [-2.8, 4.1]	0.5 [-4.0, 5.0]	0.71
			Min, Max	-15.5, 30.5	-27.6, 16.9	
			One-sample t-test	0.21	0.93	
			Wilcoxon signed-rank test	0.37	0.62	

表 5.2.2. [PPS] BNP

変数		観察ポイント	統計量	トピロキソスタット群	アロプリノール群	群間比較P値
対数変換 BNP (ln(pg/mL))	測定値	0 週	n	63	66	
			Mean±SD	4.92±0.74	4.81±0.65	0.37
			Median [Q1, Q3]	4.80 [4.38, 5.52]	4.82 [4.36, 5.25]	0.58
			Min, Max	3.33, 6.66	3.18, 6.14	
		12 週	n	62	66	
			Mean±SD	4.94±0.68	4.78±0.82	0.23
			Median [Q1, Q3]	4.85 [4.53, 5.33]	4.89 [4.19, 5.32]	0.38
			Min, Max	3.03, 6.44	2.79, 7.25	
		24 週	n	61	63	
			Mean±SD	4.93±0.72	4.79±0.75	0.32
			Median [Q1, Q3]	4.99 [4.55, 5.35]	4.87 [4.24, 5.37]	0.35
			Min, Max	2.65, 6.70	3.12, 6.09	
	変化量	12 週	n	61	65	
			Mean±SD	0.02±0.45	−0.02±0.48	0.61
			Median [Q1, Q3]	0.07 [−0.22, 0.26]	−0.05 [−0.34, 0.30]	0.39
			Min, Max	−1.29, 1.13	−1.04, 1.59	
			One-sample t-test	0.75	0.69	
			Wilcoxon signed-rank test	0.50	0.57	
		24 週	n	60	62	
			Mean±SD	0.04±0.53	−0.02±0.49	0.48
			Median [Q1, Q3]	0.06 [−0.23, 0.29]	0.04 [−0.19, 0.26]	0.85
			Min, Max	−1.68, 1.57	−1.87, 0.77	
			One-sample t-test	0.52	0.73	
			Wilcoxon signed-rank test	0.44	0.58	
	変化率	12 週	n	61	65	
			Mean±SD	1.0±9.8	−0.6±10.1	0.36
			Median [Q1, Q3]	1.3 [−4.1, 5.8]	−1.0 [−6.4, 5.8]	0.33
			Min, Max	−29.9, 32.2	−22.5, 28.1	
			One-sample t-test	0.42	0.63	
			Wilcoxon signed-rank test	0.39	0.58	
		24 週	n	60	62	
			Mean±SD	1.5±12.3	−0.2±10.2	0.38
			Median [Q1, Q3]	1.3 [−4.8, 6.4]	0.7 [−4.8, 5.1]	0.76
			Min, Max	−38.8, 40.3	−36.1, 16.6	
			One-sample t-test	0.34	0.85	
			Wilcoxon signed-rank test	0.39	0.64	

表 5.2.3. [PPS] NT-proBNP の変化率と BNP 変化率の相関

変数 1	変数 2	n	Pearson		Spearman	
			相関係数 (95%CI)	P 値	相関係数 (95%CI)	P 値
対数変換 NT-proBNP (ln(pg/mL)) 12 週変化率	対数変換 BNP (ln(pg/mL)) 12 週変化率	123	0.74 (0.65, 0.81)	<0.001	0.68 (0.57, 0.77)	<0.001
対数変換 NT-proBNP (ln(pg/mL)) 24 週変化率	対数変換 BNP (ln(pg/mL)) 24 週変化率	122	0.83 (0.76, 0.88)	<0.001	0.69 (0.59, 0.77)	<0.001

表 5.2.4. [PPS] FMD

変数		観察ポイント	統計量	トピロキソスタット群	アロプリノール群	群間比較P値
FMD(%)	測定値	0週	n	45	47	
			Mean±SD	4.78±2.39	4.63±2.10	0.76
			Median [Q1, Q3]	4.60 [3.00, 5.80]	4.70 [3.40, 5.70]	0.98
			Min, Max	1.10, 11.50	1.10, 10.80	
		24週	n	45	45	
			Mean±SD	4.90±2.48	4.24±1.59	0.14
			Median [Q1, Q3]	4.00 [3.40, 6.10]	4.10 [3.40, 5.20]	0.38
			Min, Max	1.30, 12.60	1.20, 9.20	
	変化量	24週	n	44	44	
			Mean±SD	0.11±1.74	-0.40±1.34	0.13
			Median [Q1, Q3]	0.25 [-0.75, 1.10]	-0.25 [-0.90, 0.20]	0.06
			Min, Max	-6.60, 4.40	-5.60, 2.40	
			One-sample t-test	0.69	0.05	
			Wilcoxon signed-rank test	0.43	0.035	

表 5.2.5. [PPS] EndoPAT

変数		観察ポイント	統計量	トピロキソスタット群	アロプリノール群	群間比較P値
EndoPAT（RHI指数）	測定値	0 週	n	62	65	
			Mean±SD	1.74±0.55	1.81±0.66	0.51
			Median [Q1, Q3]	1.64 [1.37, 2.00]	1.67 [1.45, 1.96]	0.55
			Min, Max	0.92, 3.66	0.75, 4.56	
		24 週	n	61	60	
			Mean±SD	1.73±0.49	1.79±0.58	0.53
			Median [Q1, Q3]	1.62 [1.39, 1.90]	1.68 [1.40, 2.04]	0.64
			Min, Max	0.78, 2.87	0.86, 3.52	
	変化量	24 週	n	60	59	
			Mean±SD	−0.03±0.47	−0.02±0.67	0.94
			Median [Q1, Q3]	0.01 [−0.25, 0.21]	−0.07 [−0.32, 0.31]	0.90
			Min, Max	−1.52, 0.98	−2.48, 1.66	
			One-sample t-test	0.63	0.81	
			Wilcoxon signed-rank test	0.71	0.78	

表 5.2.6. [PPS] FMDの変化量とEndoPATの変化量の相関

変数1	変数2	n	Pearson		Spearman	
			相関係数 (95%CI)	P値	相関係数 (95%CI)	P値
FMD（%） 24週変化量	EndoPAT（RHI指数） 24週変化量	88	0.03 (−0.18, 0.24)	0.80	0.06 (−0.15, 0.26)	0.59

表 5.2.7. [PPS] 血中尿酸値

変数		観察ポイント	統計量	トピロキソスタット群	アロプリノール群	群間比較P値
血中尿酸値(mg/dL)	測定値	0 週	n	63	66	
			Mean±SD	8.3±1.4	8.1±1.4	0.62
			Median [Q1, Q3]	8.1 [7.2, 9.1]	8.0 [7.3, 8.7]	0.66
			Min, Max	5.7, 11.6	5.4, 12.4	
		12 週	n	63	66	
			Mean±SD	5.4±1.1	5.8±1.1	0.032
			Median [Q1, Q3]	5.5 [4.9, 6.1]	5.9 [5.1, 6.4]	0.035
			Min, Max	3.2, 8.7	3.6, 9.0	
		24 週	n	61	63	
			Mean±SD	5.5±1.2	5.9±1.1	0.05
			Median [Q1, Q3]	5.4 [4.6, 6.5]	6.0 [5.2, 6.4]	0.11
			Min, Max	3.1, 7.8	3.6, 9.7	
	変化量	12 週	n	62	65	
			Mean±SD	−2.8±1.2	−2.2±1.2	0.007
			Median [Q1, Q3]	−2.7 [−3.8, −2.0]	−2.0 [−2.7, −1.5]	0.008
			Min, Max	−5.5, −0.9	−6.2, 0.7	
			One-sample t-test	<0.001	<0.001	
			Wilcoxon signed-rank test	<0.001	<0.001	
		24 週	n	60	62	
			Mean±SD	−2.7±1.5	−2.2±1.2	0.042
			Median [Q1, Q3]	−2.6 [−3.7, −1.8]	−2.0 [−3.1, −1.4]	0.020
			Min, Max	−6.6, 0.6	−5.7, 0.5	
			One-sample t-test	<0.001	<0.001	
			Wilcoxon signed-rank test	<0.001	<0.001	

表 5.2.8. [PPS] バイタルサイン、一般血液・尿検査

変数		観察ポイント	統計量	トピロキソスタット群	アロプリノール群	群間比較 P 値
体重(kg)	測定値	0 週	n	64	67	
			Mean±SD	67.1±13.7	66.4±12.9	0.78
			Median [Q1, Q3]	64.0 [56.3, 75.5]	66.0 [59.4, 75.0]	0.94
			Min, Max	44.5, 107.6	41.0, 104.0	
		12 週	n	58	61	
			Mean±SD	66.6±13.6	66.5±13.2	0.98
			Median [Q1, Q3]	64.3 [57.8, 74.1]	66.0 [59.8, 74.0]	0.79
			Min, Max	42.0, 111.0	44.0, 108.0	
		24 週	n	60	62	
			Mean±SD	67.3±14.2	66.4±14.0	0.72
			Median [Q1, Q3]	65.4 [57.9, 76.0]	64.5 [59.4, 75.0]	0.86
			Min, Max	42.0, 113.9	40.0, 110.0	
	変化量	12 週	n	58	61	
			Mean±SD	0.0±2.3	-0.4±4.5	0.58
			Median [Q1, Q3]	0.0 [-1.0, 1.0]	0.0 [-0.1, 1.0]	0.47
			Min, Max	-8.6, 8.0	-30.0, 6.9	
			One-sample t-test	0.97	0.54	
			Wilcoxon signed-rank test	0.89	0.37	
		24 週	n	60	62	
			Mean±SD	0.4±2.4	-0.4±5.0	0.27
			Median [Q1, Q3]	0.0 [-1.3, 1.6]	0.0 [-1.0, 1.3]	0.62
			Min, Max	-4.0, 8.0	-31.0, 11.6	
			One-sample t-test	0.23	0.51	
			Wilcoxon signed-rank test	0.42	0.92	
BMI(kg/m^2)	測定値	0 週	n	64	67	
			Mean±SD	25.3±4.3	25.0±3.6	0.72
			Median [Q1, Q3]	25.0 [21.9, 28.0]	24.7 [22.1, 27.2]	0.86
			Min, Max	17.1, 39.0	19.4, 38.2	
		12 週	n	58	61	
			Mean±SD	25.1±4.5	24.9±3.9	0.80
			Median [Q1, Q3]	24.3 [21.6, 27.9]	24.7 [22.2, 26.7]	0.92
			Min, Max	16.0, 40.3	19.0, 39.7	
		24 週	n	60	62	
			Mean±SD	25.4±4.6	25.0±4.1	0.63
			Median [Q1, Q3]	25.2 [22.2, 28.2]	24.8 [22.4, 26.7]	0.67
			Min, Max	16.0, 41.3	18.3, 40.4	
	変化量	12 週	n	58	61	
			Mean±SD	0.0±0.8	-0.1±1.7	0.54
			Median [Q1, Q3]	0.0 [-0.4, 0.3]	0.0 [0.0, 0.4]	0.50
			Min, Max	-2.6, 2.9	-11.2, 2.4	
			One-sample t-test	0.85	0.55	
			Wilcoxon signed-rank test	0.95	0.44	
		24 週	n	60	62	
			Mean±SD	0.2±0.9	-0.2±1.9	0.24
			Median [Q1, Q3]	0.0 [-0.5, 0.7]	0.0 [-0.4, 0.6]	0.56
			Min, Max	-1.4, 2.9	-11.5, 4.1	

			One-sample t-test	0.18	0.50	
			Wilcoxon signed-rank test	0.33	0.92	
収縮期血圧 (mmHg)	測定値	0 週	n	64	67	
			Mean±SD	124.1±19.4	125.2±17.9	0.74
			Median [Q1, Q3]	120.5 [110.5, 136.5]	124.0 [113.0, 137.0]	0.70
			Min, Max	91.0, 171.0	90.0, 163.0	
		12 週	n	63	66	
			Mean±SD	122.8±17.8	124.6±17.2	0.55
			Median [Q1, Q3]	120.0 [109.0, 135.0]	121.5 [114.0, 140.0]	0.45
			Min, Max	96.0, 168.0	93.0, 158.0	
		24 週	n	61	63	
			Mean±SD	126.3±18.5	123.9±18.3	0.47
			Median [Q1, Q3]	124.0 [115.0, 136.0]	124.0 [114.0, 135.0]	0.62
			Min, Max	95.0, 179.0	84.0, 164.0	
	変化量	12 週	n	63	66	
			Mean±SD	-1.2±17.2	-0.1±16.0	0.70
			Median [Q1, Q3]	1.0 [-14.0, 10.0]	2.0 [-9.0, 8.0]	0.67
			Min, Max	-53.0, 37.0	-53.0, 32.0	
			One-sample t-test	0.57	0.96	
			Wilcoxon signed-rank test	0.76	0.82	
		24 週	n	61	63	
			Mean±SD	2.2±16.4	0.1±16.7	0.47
			Median [Q1, Q3]	2.0 [-8.0, 11.0]	1.0 [-10.0, 9.0]	0.47
			Min, Max	-47.0, 53.0	-42.0, 40.0	
			One-sample t-test	0.30	0.98	
			Wilcoxon signed-rank test	0.23	0.91	
拡張期血圧 (mmHg)	測定値	0 週	n	64	67	
			Mean±SD	72.5±14.7	73.2±12.2	0.76
			Median [Q1, Q3]	70.5 [60.0, 82.0]	73.0 [66.0, 79.0]	0.47
			Min, Max	47.0, 110.0	43.0, 118.0	
		12 週	n	63	66	
			Mean±SD	71.7±15.2	72.8±14.0	0.67
			Median [Q1, Q3]	70.0 [60.0, 83.0]	73.5 [63.0, 84.0]	0.53
			Min, Max	46.0, 106.0	40.0, 108.0	
		24 週	n	61	63	
			Mean±SD	71.9±14.4	73.6±14.1	0.51
			Median [Q1, Q3]	71.0 [60.0, 83.0]	70.0 [64.0, 82.0]	0.55
			Min, Max	40.0, 110.0	48.0, 118.0	
	変化量	12 週	n	63	66	
			Mean±SD	-1.1±15.2	-0.2±12.4	0.71
			Median [Q1, Q3]	-2.0 [-11.0, 5.0]	0.0 [-8.0, 8.0]	0.38
			Min, Max	-45.0, 45.0	-34.0, 35.0	
			One-sample t-test	0.57	0.91	
			Wilcoxon signed-rank test	0.28	0.94	
		24 週	n	61	63	
			Mean±SD	-0.6±14.0	0.7±12.3	0.58
			Median [Q1, Q3]	0.0 [-8.0, 6.0]	0.0 [-8.0, 8.0]	0.58
			Min, Max	-34.0, 52.0	-27.0, 32.0	
			One-sample t-test	0.74	0.65	

			Wilcoxon signed-rank test	0.51	0.82	
脈拍(bpm)	測定値	0 週	n	63	67	
			Mean±SD	71.8±13.6	71.9±13.4	0.99
			Median [Q1, Q3]	70.0 [63.0, 80.0]	72.0 [62.0, 83.0]	0.81
			Min, Max	44.0, 111.0	41.0, 98.0	
		12 週	n	59	65	
			Mean±SD	75.6±15.6	73.3±12.8	0.37
			Median [Q1, Q3]	75.0 [64.0, 86.0]	74.0 [63.0, 84.0]	0.52
			Min, Max	47.0, 134.0	47.0, 106.0	
		24 週	n	59	63	
			Mean±SD	72.6±14.2	75.5±16.4	0.30
			Median [Q1, Q3]	73.0 [63.0, 82.0]	73.0 [62.0, 86.0]	0.52
			Min, Max	43.0, 120.0	51.0, 130.0	
	変化量	12 週	n	59	65	
			Mean±SD	2.7±10.2	1.1±13.1	0.46
			Median [Q1, Q3]	0.0 [-4.0, 7.0]	0.0 [-6.0, 9.0]	0.79
			Min, Max	-14.0, 33.0	-40.0, 29.0	
			One-sample t-test	0.047	0.50	
			Wilcoxon signed-rank test	0.16	0.37	
		24 週	n	59	63	
			Mean±SD	1.1±14.6	3.3±16.4	0.44
			Median [Q1, Q3]	0.0 [-7.0, 10.0]	1.0 [-7.0, 9.0]	0.86
			Min, Max	-47.0, 46.0	-25.0, 56.0	
			One-sample t-test	0.55	0.11	
			Wilcoxon signed-rank test	0.47	0.34	
NYHA 分類	測定値	0 週	n	64	67	
			Median [Q1, Q3]	1.0 [1.0, 2.0]	1.0 [1.0, 2.0]	0.72
			Min, Max	1.0, 2.0	1.0, 2.0	
		12 週	n	62	66	
			Median [Q1, Q3]	1.0 [1.0, 2.0]	1.0 [1.0, 2.0]	0.81
			Min, Max	1.0, 2.0	1.0, 2.0	
		24 週	n	61	62	
			Median [Q1, Q3]	1.0 [1.0, 2.0]	1.0 [1.0, 2.0]	0.61
			Min, Max	1.0, 3.0	1.0, 4.0	
	変化量	12 週	n	62	66	
			Median [Q1, Q3]	0.0 [0.0, 0.0]	0.0 [0.0, 0.0]	0.78
			Min, Max	-1.0, 1.0	-1.0, 1.0	
			Wilcoxon signed-rank test	1.00	0.73	
		24 週	n	61	62	
			Median [Q1, Q3]	0.0 [0.0, 0.0]	0.0 [0.0, 0.0]	0.16
			Min, Max	-1.0, 1.0	-1.0, 2.0	
			Wilcoxon signed-rank test	1.00	0.23	
赤血球数(× 10^4/μL)	測定値	0 週	n	63	66	
			Mean±SD	443.8±47.0	433.4±64.4	0.30
			Median [Q1, Q3]	446.0 [411.0, 472.0]	431.5 [384.0, 478.0]	0.22
			Min, Max	355.0, 568.0	274.0, 577.0	
		12 週	n	63	66	
			Mean±SD	443.7±52.1	429.0±69.1	0.18
			Median [Q1, Q3]	433.0 [406.0, 488.0]	422.5 [378.0, 485.0]	0.22

			Min, Max	303.0, 565.0	285.0, 570.0	
		24 週	n	62	63	
			Mean±SD	440.8±57.2	421.3±66.5	0.08
			Median [Q1, Q3]	447.5 [408.0, 480.0]	410.0 [381.0, 458.0]	0.05
			Min, Max	313.0, 598.0	284.0, 580.0	
	変化量	12 週	n	62	65	
			Mean±SD	-0.1±25.3	-4.3±33.9	0.42
			Median [Q1, Q3]	1.0 [-20.0, 15.0]	0.0 [-23.0, 18.0]	0.65
			Min, Max	-55.0, 73.0	-135.0, 70.0	
			One-sample t-test	0.98	0.31	
			Wilcoxon signed-rank test	0.97	0.54	
		24 週	n	61	62	
			Mean±SD	-3.7±29.6	-8.7±25.7	0.32
			Median [Q1, Q3]	-2.0 [-16.0, 16.0]	-11.0 [-26.0, 10.0]	0.16
			Min, Max	-125.0, 62.0	-64.0, 53.0	
			One-sample t-test	0.33	0.010	
			Wilcoxon signed-rank test	0.57	0.012	
白血球数 (/μL)	測定値	0 週	n	63	66	
			Mean±SD	5730.2±1327.5	6116.1±1846.8	0.18
			Median [Q1, Q3]	5500.0 [4900.0, 6700.0]	5680.0 [5000.0, 7000.0]	0.32
			Min, Max	2900.0, 9400.0	2500.0, 11700.0	
		12 週	n	63	66	
			Mean±SD	5803.2±1495.2	5974.2±1723.0	0.55
			Median [Q1, Q3]	5500.0 [4700.0, 6900.0]	5850.0 [4700.0, 7000.0]	0.64
			Min, Max	3200.0, 9800.0	3100.0, 11400.0	
		24 週	n	62	63	
			Mean±SD	5662.9±1439.1	5950.8±1648.4	0.30
			Median [Q1, Q3]	5550.0 [4600.0, 6400.0]	5800.0 [4900.0, 6400.0]	0.38
			Min, Max	3000.0, 10100.0	3100.0, 11100.0	
	変化量	12 週	n	62	65	
			Mean±SD	108.1±1125.3	-82.5±1274.3	0.37
			Median [Q1, Q3]	200.0 [-700.0, 1000.0]	0.0 [-700.0, 400.0]	0.43
			Min, Max	-2100.0, 2600.0	-5900.0, 2600.0	
			One-sample t-test	0.45	0.60	
			Wilcoxon signed-rank test	0.50	0.61	
		24 週	n	61	62	
			Mean±SD	24.6±978.9	-15.5±1107.0	0.83
			Median [Q1, Q3]	100.0 [-400.0, 400.0]	-100.0 [-600.0, 500.0]	0.38
			Min, Max	-3400.0, 2900.0	-2700.0, 3400.0	
			One-sample t-test	0.85	0.91	
			Wilcoxon signed-rank test	0.54	0.53	
ヘモグロビン (g/dL)	測定値	0 週	n	63	66	
			Mean±SD	13.6±1.5	13.3±1.9	0.40
			Median [Q1, Q3]	13.8 [12.4, 14.7]	13.1 [12.1, 14.7]	0.32

			Min, Max	9.0, 16.6	8.8, 17.1	
		12 週	n	63	66	
			Mean±SD	13.5±1.6	13.2±2.1	0.38
			Median [Q1, Q3]	13.4 [12.4, 14.9]	13.0 [11.4, 15.0]	0.40
			Min, Max	9.2, 16.5	8.6, 16.9	
		24 週	n	62	63	
			Mean±SD	13.4±1.9	13.0±2.0	0.34
			Median [Q1, Q3]	13.7 [12.1, 14.9]	12.8 [11.7, 14.5]	0.26
			Min, Max	8.5, 17.0	8.5, 17.5	
	変化量	12 週	n	62	65	
			Mean±SD	-0.1±0.8	-0.1±0.9	0.91
			Median [Q1, Q3]	-0.1 [-0.5, 0.3]	0.0 [-0.5, 0.5]	0.62
			Min, Max	-1.9, 2.2	-4.0, 1.7	
			One-sample t-test	0.32	0.46	
			Wilcoxon signed-rank test	0.29	0.75	
		24 週	n	61	62	
			Mean±SD	-0.3±0.9	-0.2±0.8	0.62
			Median [Q1, Q3]	-0.1 [-0.7, 0.4]	-0.3 [-0.8, 0.3]	0.96
			Min, Max	-3.6, 1.5	-1.7, 1.9	
			One-sample t-test	0.036	0.07	
			Wilcoxon signed-rank test	0.07	0.06	
ヘマトクリット (%)	測定値	0 週	n	63	66	
			Mean±SD	41.1±4.3	40.2±5.2	0.28
			Median [Q1, Q3]	41.4 [38.3, 43.8]	39.9 [36.5, 43.2]	0.18
			Min, Max	28.3, 51.6	27.4, 51.1	
		12 週	n	63	66	
			Mean±SD	41.1±4.7	40.2±5.8	0.32
			Median [Q1, Q3]	40.9 [38.0, 44.4]	39.6 [35.6, 45.2]	0.34
			Min, Max	28.5, 51.9	27.5, 50.4	
		24 週	n	62	63	
			Mean±SD	40.7±5.3	39.5±5.6	0.24
			Median [Q1, Q3]	41.0 [37.0, 44.4]	38.9 [35.5, 43.1]	0.18
			Min, Max	28.0, 53.5	27.2, 52.2	
	変化量	12 週	n	62	65	
			Mean±SD	-0.1±2.3	0.0±2.9	0.87
			Median [Q1, Q3]	0.1 [-1.2, 1.3]	-0.1 [-1.5, 1.6]	0.62
			Min, Max	-6.3, 5.9	-13.1, 5.1	
			One-sample t-test	0.68	0.91	
			Wilcoxon signed-rank test	0.84	0.69	
		24 週	n	61	62	
			Mean±SD	-0.6±2.9	-0.4±2.2	0.74
			Median [Q1, Q3]	-0.5 [-1.5, 1.3]	-0.5 [-2.4, 1.2]	0.68
			Min, Max	-12.1, 4.9	-5.0, 5.0	
			One-sample t-test	0.13	0.15	
			Wilcoxon signed-rank test	0.36	0.12	
血小板数(× 10^4/μL)	測定値	0 週	n	63	66	
			Mean±SD	20.3±8.1	20.0±6.6	0.80
			Median [Q1, Q3]	19.5 [15.5, 22.2]	19.3 [15.7, 22.6]	0.90
			Min, Max	8.6, 68.6	9.9, 46.8	

		12 週	n	63	66	
			Mean±SD	20.6±6.4	20.2±5.6	0.72
			Median [Q1, Q3]	19.7 [16.6, 23.1]	20.1 [15.9, 23.5]	0.90
			Min, Max	8.0, 37.1	10.1, 37.1	
		24 週	n	62	63	
			Mean±SD	20.3±5.6	19.4±5.4	0.36
			Median [Q1, Q3]	19.9 [17.0, 23.6]	18.9 [16.2, 22.5]	0.39
			Min, Max	8.6, 39.4	8.0, 37.0	
	変化量	12 週	n	62	65	
			Mean±SD	0.3±5.9	0.7±3.8	0.67
			Median [Q1, Q3]	0.5 [-0.5, 2.1]	0.3 [-0.9, 1.8]	0.58
			Min, Max	-35.2, 14.3	-7.3, 21.7	
			One-sample t-test	0.65	0.14	
			Wilcoxon signed-rank test	0.038	0.13	
		24 週	n	61	62	
			Mean±SD	0.9±2.9	0.0±2.7	0.09
			Median [Q1, Q3]	0.9 [-0.8, 2.5]	-0.3 [-1.5, 1.1]	0.022
			Min, Max	-6.4, 8.6	-6.0, 11.5	
			One-sample t-test	0.021	0.95	
			Wilcoxon signed-rank test	0.011	0.52	
AST(IU/L)	測定値	0 週	n	63	66	
			Mean±SD	26.2±12.7	23.5±10.3	0.19
			Median [Q1, Q3]	24.0 [19.0, 29.0]	21.0 [17.0, 27.0]	0.06
			Min, Max	13.0, 106.0	12.0, 85.0	
		12 週	n	63	66	
			Mean±SD	26.4±9.2	24.5±8.3	0.22
			Median [Q1, Q3]	25.0 [20.0, 30.0]	23.0 [19.0, 28.0]	0.19
			Min, Max	14.0, 63.0	11.0, 57.0	
		24 週	n	62	63	
			Mean±SD	25.4±6.7	24.0±7.6	0.31
			Median [Q1, Q3]	24.0 [20.0, 31.0]	22.0 [19.0, 27.0]	0.20
			Min, Max	16.0, 45.0	13.0, 47.0	
	変化量	12 週	n	62	65	
			Mean±SD	0.3±11.8	0.9±6.9	0.71
			Median [Q1, Q3]	1.0 [-2.0, 2.0]	1.0 [-2.0, 5.0]	0.53
			Min, Max	-73.0, 35.0	-28.0, 13.0	
			One-sample t-test	0.85	0.28	
			Wilcoxon signed-rank test	0.21	0.040	
		24 週	n	61	62	
			Mean±SD	-0.4±10.8	1.3±6.0	0.29
			Median [Q1, Q3]	1.0 [-3.0, 4.0]	1.0 [-2.0, 4.0]	0.57
			Min, Max	-73.0, 11.0	-18.0, 19.0	
			One-sample t-test	0.77	0.10	
			Wilcoxon signed-rank test	0.25	0.06	
ALT(IU/L)	測定値	0 週	n	63	66	
			Mean±SD	20.5±11.8	18.1±10.5	0.24
			Median [Q1, Q3]	18.0 [14.0, 25.0]	16.0 [12.0, 21.0]	0.05
			Min, Max	8.0, 87.0	6.0, 63.0	
		12 週	n	63	66	

			Mean±SD	21.0±10.5	18.2±8.4	0.09
			Median [Q1, Q3]	18.0 [14.0, 28.0]	16.0 [13.0, 22.0]	0.13
			Min, Max	8.0, 60.0	6.0, 48.0	
		24 週	n	62	63	
			Mean±SD	20.5±10.0	18.2±9.4	0.18
			Median [Q1, Q3]	19.5 [13.0, 24.0]	16.0 [11.0, 23.0]	0.12
			Min, Max	8.0, 60.0	7.0, 55.0	
	変化量	12 週	n	62	65	
			Mean±SD	0.4±10.5	0.1±6.5	0.85
			Median [Q1, Q3]	1.0 [-2.0, 4.0]	1.0 [-1.0, 3.0]	0.94
			Min, Max	-66.0, 21.0	-27.0, 15.0	
			One-sample t-test	0.78	0.92	
			Wilcoxon signed-rank test	0.15	0.13	
		24 週	n	61	62	
			Mean±SD	0.1±11.3	0.7±8.0	0.76
			Median [Q1, Q3]	0.0 [-2.0, 3.0]	0.0 [-2.0, 4.0]	0.74
			Min, Max	-72.0, 32.0	-20.0, 47.0	
			One-sample t-test	0.94	0.52	
			Wilcoxon signed-rank test	0.38	0.52	
ALP(IU/L)	測定値	0 週	n	60	65	
			Mean±SD	228.8±95.0	221.8±74.8	0.65
			Median [Q1, Q3]	200.5 [175.5, 239.0]	207.0 [175.0, 261.0]	0.78
			Min, Max	126.0, 636.0	86.0, 473.0	
		12 週	n	60	66	
			Mean±SD	237.9±94.1	231.9±76.9	0.70
			Median [Q1, Q3]	217.0 [181.5, 263.5]	216.5 [182.0, 289.0]	0.98
			Min, Max	110.0, 658.0	94.0, 492.0	
		24 週	n	61	63	
			Mean±SD	236.3±91.7	234.3±90.5	0.90
			Median [Q1, Q3]	220.0 [168.0, 259.0]	220.0 [177.0, 266.0]	0.89
			Min, Max	122.0, 525.0	96.0, 583.0	
	変化量	12 週	n	57	64	
			Mean±SD	6.5±37.8	11.2±43.2	0.53
			Median [Q1, Q3]	4.0 [-11.0, 22.0]	17.5 [1.5, 30.0]	0.07
			Min, Max	-91.0, 101.0	-182.0, 141.0	
			One-sample t-test	0.20	0.042	
			Wilcoxon signed-rank test	0.25	<0.001	
		24 週	n	57	61	
			Mean±SD	7.2±52.9	17.1±52.3	0.31
			Median [Q1, Q3]	3.0 [-17.0, 32.0]	15.0 [-7.0, 33.0]	0.13
			Min, Max	-152.0, 222.0	-173.0, 264.0	
			One-sample t-test	0.31	0.013	
			Wilcoxon signed-rank test	0.35	<0.001	
T-Bil(mg/dL)	測定値	0 週	n	63	66	
			Mean±SD	0.89±0.39	0.83±0.42	0.36
			Median [Q1, Q3]	0.80 [0.60, 1.00]	0.72 [0.60, 0.98]	0.14
			Min, Max	0.27, 2.50	0.30, 2.79	
		12 週	n	62	66	
			Mean±SD	0.82±0.35	0.78±0.36	0.59

			Median [Q1, Q3]	0.73 [0.51, 1.00]	0.70 [0.50, 0.90]	0.45
			Min, Max	0.26, 1.70	0.30, 2.01	
		24 週	n	62	63	
			Mean±SD	0.81±0.32	0.77±0.36	0.53
			Median [Q1, Q3]	0.80 [0.60, 1.00]	0.70 [0.50, 0.90]	0.32
			Min, Max	0.29, 1.80	0.30, 2.30	
	変化量	12 週	n	61	65	
			Mean±SD	-0.09±0.31	-0.04±0.33	0.40
			Median [Q1, Q3]	-0.10 [-0.20, 0.06]	-0.10 [-0.20, 0.10]	0.93
			Min, Max	-1.00, 0.48	-0.78, 1.21	
			One-sample t-test	0.029	0.32	
			Wilcoxon signed-rank test	0.040	0.07	
		24 週	n	61	62	
			Mean±SD	-0.09±0.27	-0.06±0.24	0.39
			Median [Q1, Q3]	-0.10 [-0.20, 0.10]	-0.10 [-0.20, 0.10]	0.52
			Min, Max	-0.80, 0.50	-0.60, 0.40	
			One-sample t-test	0.008	0.07	
			Wilcoxon signed-rank test	0.013	0.12	
γGTP(U/L)	測定値	0 週	n	62	65	
			Mean±SD	50.1±47.9	39.0±38.6	0.15
			Median [Q1, Q3]	32.5 [23.0, 56.0]	26.0 [20.0, 40.0]	0.044
			Min, Max	10.0, 283.0	9.0, 235.0	
		12 週	n	62	66	
			Mean±SD	53.1±50.3	46.7±70.5	0.56
			Median [Q1, Q3]	35.5 [24.0, 60.0]	25.5 [18.0, 41.0]	0.023
			Min, Max	9.0, 265.0	10.0, 522.0	
		24 週	n	61	63	
			Mean±SD	54.1±53.3	37.1±32.1	0.033
			Median [Q1, Q3]	36.0 [22.0, 60.0]	26.0 [17.0, 41.0]	0.020
			Min, Max	8.0, 247.0	11.0, 174.0	
	変化量	12 週	n	60	64	
			Mean±SD	2.3±22.9	8.5±62.9	0.47
			Median [Q1, Q3]	1.0 [-3.5, 7.5]	0.0 [-3.0, 3.5]	0.46
			Min, Max	-71.0, 88.0	-33.0, 493.0	
			One-sample t-test	0.44	0.28	
			Wilcoxon signed-rank test	0.20	0.61	
		24 週	n	59	61	
			Mean±SD	6.0±28.1	0.0±15.4	0.15
			Median [Q1, Q3]	0.0 [-5.0, 10.0]	1.0 [-3.0, 3.0]	0.62
			Min, Max	-50.0, 145.0	-61.0, 41.0	
			One-sample t-test	0.11	0.98	
			Wilcoxon signed-rank test	0.31	0.80	
対数変換 γGTP(ln (U/L))	測定値	0 週	n	62	65	
			Mean±SD	3.61±0.74	3.38±0.69	0.07
			Median [Q1, Q3]	3.48 [3.14, 4.03]	3.26 [3.00, 3.69]	0.044
			Min, Max	2.30, 5.65	2.20, 5.46	
		12 週	n	62	66	
			Mean±SD	3.68±0.73	3.43±0.78	0.07
			Median [Q1, Q3]	3.57 [3.18, 4.09]	3.24 [2.89, 3.71]	0.023

			Min, Max	2.20, 5.58	2.30, 6.26	
		24 週	n	61	63	
			Mean±SD	3.67±0.77	3.37±0.66	0.021
			Median [Q1, Q3]	3.58 [3.09, 4.09]	3.26 [2.83, 3.71]	0.020
			Min, Max	2.08, 5.51	2.40, 5.16	
	変化量	12 週	n	60	64	
			Mean±SD	0.04±0.26	0.06±0.43	0.75
			Median [Q1, Q3]	0.04 [-0.09, 0.20]	0.00 [-0.12, 0.13]	0.48
			Min, Max	-0.67, 0.62	-0.57, 2.89	
			One-sample t-test	0.25	0.28	
			Wilcoxon signed-rank test	0.15	0.65	
		24 週	n	59	61	
			Mean±SD	0.09±0.38	0.02±0.26	0.25
			Median [Q1, Q3]	0.00 [-0.14, 0.23]	0.01 [-0.14, 0.13]	0.53
			Min, Max	-0.77, 1.46	-0.59, 0.91	
			One-sample t-test	0.09	0.61	
			Wilcoxon signed-rank test	0.26	0.81	
TC(mg/dL)	測定値	0 週	n	62	66	
			Mean±SD	175.2±31.0	167.6±31.9	0.18
			Median [Q1, Q3]	175.0 [154.0, 194.0]	164.0 [146.0, 187.0]	0.15
			Min, Max	110.0, 256.0	96.0, 254.0	
		12 週	n	63	66	
			Mean±SD	170.9±37.5	170.1±38.6	0.91
			Median [Q1, Q3]	169.0 [147.0, 193.0]	164.0 [142.0, 192.0]	0.86
			Min, Max	69.0, 276.0	97.0, 264.0	
		24 週	n	62	63	
			Mean±SD	173.4±32.6	162.0±30.3	0.046
			Median [Q1, Q3]	172.5 [151.0, 191.0]	161.0 [143.0, 180.0]	0.08
			Min, Max	112.0, 249.0	97.0, 243.0	
	変化量	12 週	n	61	65	
			Mean±SD	-4.6±24.6	1.5±21.9	0.15
			Median [Q1, Q3]	-6.0 [-18.0, 9.0]	-2.0 [-11.0, 18.0]	0.27
			Min, Max	-91.0, 44.0	-49.0, 51.0	
			One-sample t-test	0.15	0.59	
			Wilcoxon signed-rank test	0.19	0.88	
		24 週	n	60	62	
			Mean±SD	-1.2±23.1	-4.7±18.9	0.36
			Median [Q1, Q3]	3.0 [-9.5, 12.0]	-6.0 [-16.0, 5.0]	0.08
			Min, Max	-69.0, 69.0	-70.0, 33.0	
			One-sample t-test	0.68	0.05	
			Wilcoxon signed-rank test	0.67	0.036	
HDL-C (mg/dL)	測定値	0 週	n	63	66	
			Mean±SD	56.3±16.3	56.0±18.4	0.91
			Median [Q1, Q3]	53.0 [47.0, 65.0]	51.0 [44.0, 63.0]	0.49
			Min, Max	30.0, 105.0	30.0, 135.0	
		12 週	n	62	66	
			Mean±SD	56.5±14.6	57.6±20.5	0.73
			Median [Q1, Q3]	56.0 [45.0, 65.0]	53.0 [44.0, 62.0]	0.56
			Min, Max	27.0, 103.0	30.0, 149.0	

		24 週	n	61	63	
			Mean±SD	55.7±14.2	54.3±17.4	0.63
			Median [Q1, Q3]	53.0 [47.0, 62.0]	52.0 [44.0, 58.0]	0.29
			Min, Max	33.0, 98.0	31.0, 139.0	
	変化量	12 週	n	61	65	
			Mean±SD	-0.1±9.4	1.9±7.7	0.17
			Median [Q1, Q3]	1.0 [-5.0, 6.0]	0.0 [-3.0, 6.0]	0.49
			Min, Max	-30.0, 29.0	-13.0, 26.0	
			One-sample t-test	0.90	0.047	
			Wilcoxon signed-rank test	0.91	0.18	
		24 週	n	60	62	
			Mean±SD	-0.5±8.6	-1.0±8.8	0.72
			Median [Q1, Q3]	-1.0 [-4.5, 4.5]	0.0 [-4.0, 4.0]	0.90
			Min, Max	-23.0, 23.0	-39.0, 15.0	
			One-sample t-test	0.68	0.36	
			Wilcoxon signed-rank test	0.72	0.87	
TG (mg/dL)	測定値	0 週	n	62	66	
			Mean±SD	110.9±50.5	117.7±65.3	0.51
			Median [Q1, Q3]	96.5 [74.0, 145.0]	101.5 [73.0, 142.0]	0.80
			Min, Max	32.0, 280.0	44.0, 408.0	
		12 週	n	59	61	
			Mean±SD	126.8±66.5	124.2±69.7	0.83
			Median [Q1, Q3]	106.0 [70.0, 169.0]	104.0 [79.0, 138.0]	0.94
			Min, Max	42.0, 338.0	22.0, 420.0	
		24 週	n	61	63	
			Mean±SD	111.2±56.7	108.1±55.1	0.76
			Median [Q1, Q3]	99.0 [70.0, 134.0]	97.0 [70.0, 126.0]	0.73
			Min, Max	46.0, 362.0	47.0, 348.0	
	変化量	12 週	n	57	61	
			Mean±SD	17.5±43.2	8.6±64.0	0.38
			Median [Q1, Q3]	14.0 [-6.0, 38.0]	2.0 [-14.0, 33.0]	0.16
			Min, Max	-101.0, 121.0	-191.0, 284.0	
			One-sample t-test	0.003	0.30	
			Wilcoxon signed-rank test	0.002	0.27	
		24 週	n	59	62	
			Mean±SD	2.3±36.7	-7.9±43.6	0.17
			Median [Q1, Q3]	4.0 [-21.0, 23.0]	-5.0 [-22.0, 18.0]	0.30
			Min, Max	-65.0, 136.0	-203.0, 113.0	
			One-sample t-test	0.63	0.16	
			Wilcoxon signed-rank test	0.85	0.23	
LDL-C (mg/dL)	測定値	0 週	n	63	66	
			Mean±SD	101.7±30.4	94.3±24.5	0.13
			Median [Q1, Q3]	99.0 [80.0, 125.0]	92.0 [79.0, 107.0]	0.15
			Min, Max	45.0, 176.0	38.0, 160.0	
		12 週	n	62	66	
			Mean±SD	97.6±31.7	94.5±29.8	0.56
			Median [Q1, Q3]	92.5 [73.0, 116.0]	91.5 [76.0, 109.0]	0.70
			Min, Max	45.0, 180.0	39.0, 174.0	
		24 週	n	61	63	

			Mean±SD	99.7±27.4	91.1±25.5	0.08
			Median [Q1, Q3]	101.0 [76.0, 119.0]	90.0 [72.0, 110.0]	0.09
			Min, Max	44.0, 156.0	35.0, 152.0	
	変化量	12 週	n	61	65	
			Mean±SD	-3.6±20.5	-0.9±17.3	0.42
			Median [Q1, Q3]	-4.0 [-12.0, 6.0]	-2.0 [-11.0, 11.0]	0.60
			Min, Max	-75.0, 37.0	-43.0, 47.0	
			One-sample t-test	0.17	0.67	
			Wilcoxon signed-rank test	0.18	0.41	
		24 週	n	60	62	
			Mean±SD	-0.8±19.3	-3.1±13.4	0.44
			Median [Q1, Q3]	0.0 [-6.5, 10.0]	-5.5 [-11.0, 6.0]	0.08
			Min, Max	-68.0, 52.0	-34.0, 26.0	
			One-sample t-test	0.76	0.07	
			Wilcoxon signed-rank test	0.82	0.06	
Na(mEq/L)	測定値	0 週	n	63	66	
			Mean±SD	140.5±3.3	140.5±2.2	0.91
			Median [Q1, Q3]	141.0 [139.0, 143.0]	140.0 [139.0, 142.0]	0.51
			Min, Max	121.0, 145.0	135.0, 146.0	
		12 週	n	63	66	
			Mean±SD	140.4±2.6	140.5±3.9	0.88
			Median [Q1, Q3]	140.0 [139.0, 142.0]	141.0 [139.0, 142.0]	0.39
			Min, Max	129.0, 146.0	114.0, 146.0	
		24 週	n	62	63	
			Mean±SD	140.6±2.5	140.4±1.9	0.56
			Median [Q1, Q3]	141.0 [139.0, 142.0]	140.0 [139.0, 142.0]	0.32
			Min, Max	134.0, 146.0	136.0, 145.0	
	変化量	12 週	n	62	65	
			Mean±SD	-0.1±2.2	0.0±4.0	0.74
			Median [Q1, Q3]	0.0 [-2.0, 1.0]	0.0 [-1.0, 2.0]	0.09
			Min, Max	-4.0, 8.0	-27.0, 6.0	
			One-sample t-test	0.60	0.93	
			Wilcoxon signed-rank test	0.33	0.21	
		24 週	n	61	62	
			Mean±SD	0.0±2.5	-0.2±2.1	0.67
			Median [Q1, Q3]	0.0 [-1.0, 1.0]	0.0 [-2.0, 1.0]	0.90
			Min, Max	-5.0, 14.0	-6.0, 7.0	
			One-sample t-test	0.96	0.54	
			Wilcoxon signed-rank test	0.52	0.36	
血糖値(mg/dL)	測定値	0 週	n	62	66	
			Mean±SD	105.8±18.8	103.8±14.6	0.52
			Median [Q1, Q3]	101.5 [93.0, 113.0]	101.5 [94.0, 109.0]	0.85
			Min, Max	80.0, 179.0	83.0, 151.0	
		12 週	n	61	60	
			Mean±SD	111.4±22.8	114.3±27.8	0.53
			Median [Q1, Q3]	105.0 [98.0, 121.0]	106.0 [95.0, 125.0]	0.97
			Min, Max	49.0, 192.0	57.0, 194.0	
		24 週	n	61	63	
			Mean±SD	107.4±19.7	106.2±17.0	0.72

			Median [Q1, Q3]	103.0 [96.0, 115.0]	102.0 [96.0, 114.0]	0.66
			Min, Max	77.0, 186.0	83.0, 162.0	
	変化量	12 週	n	59	60	
			Mean±SD	6.3±18.6	10.4±20.0	0.25
			Median [Q1, Q3]	5.0 [-1.0, 17.0]	7.0 [-1.0, 17.0]	0.62
			Min, Max	-74.0, 47.0	-42.0, 79.0	
			One-sample t-test	0.012	<0.001	
			Wilcoxon signed-rank test	<0.001	<0.001	
		24 週	n	59	62	
			Mean±SD	1.3±12.2	2.1±11.6	0.73
			Median [Q1, Q3]	2.0 [-5.0, 7.0]	2.5 [-2.0, 6.0]	0.78
			Min, Max	-33.0, 41.0	-45.0, 53.0	
			One-sample t-test	0.42	0.17	
			Wilcoxon signed-rank test	0.37	0.049	
BUN(血中浸透圧)(mg/dL)	測定値	0 週	n	63	66	
			Mean±SD	19.8±6.4	19.6±5.2	0.82
			Median [Q1, Q3]	18.2 [15.0, 23.0]	18.8 [16.0, 22.0]	0.78
			Min, Max	10.0, 42.0	10.0, 35.0	
		12 週	n	63	66	
			Mean±SD	20.6±8.5	20.1±8.7	0.74
			Median [Q1, Q3]	18.0 [15.0, 23.5]	19.0 [15.0, 22.0]	0.72
			Min, Max	11.0, 67.0	9.0, 70.0	
		24 週	n	62	63	
			Mean±SD	21.0±9.0	19.4±6.2	0.25
			Median [Q1, Q3]	19.0 [15.2, 25.0]	19.0 [15.8, 21.6]	0.58
			Min, Max	9.0, 57.0	8.0, 47.0	
	変化量	12 週	n	62	65	
			Mean±SD	1.0±5.6	0.5±7.8	0.68
			Median [Q1, Q3]	0.9 [-2.0, 4.0]	0.0 [-3.0, 3.0]	0.53
			Min, Max	-9.7, 25.0	-18.7, 48.0	
			One-sample t-test	0.17	0.62	
			Wilcoxon signed-rank test	0.22	0.80	
		24 週	n	61	62	
			Mean±SD	1.3±6.2	-0.1±5.2	0.17
			Median [Q1, Q3]	1.0 [-2.0, 3.0]	0.0 [-2.0, 3.0]	0.32
			Min, Max	-13.0, 27.0	-17.3, 21.0	
			One-sample t-test	0.10	0.89	
			Wilcoxon signed-rank test	0.16	1.00	
血清クレアチニン(mg/dL)	測定値	0 週	n	63	65	
			Mean±SD	1.06±0.21	1.03±0.24	0.39
			Median [Q1, Q3]	1.07 [0.89, 1.19]	0.97 [0.87, 1.17]	0.21
			Min, Max	0.65, 1.63	0.58, 1.71	
		12 週	n	63	66	
			Mean±SD	1.06±0.23	1.05±0.30	0.73
			Median [Q1, Q3]	1.08 [0.84, 1.18]	0.97 [0.84, 1.22]	0.41
			Min, Max	0.72, 1.69	0.53, 2.00	
		24 週	n	62	63	
			Mean±SD	1.07±0.28	1.04±0.29	0.59
			Median [Q1, Q3]	1.05 [0.83, 1.20]	0.96 [0.83, 1.25]	0.48

			Min, Max	0.57, 2.20	0.56, 2.01	
	変化量	12 週	n	62	64	
			Mean±SD	0.01±0.11	0.02±0.18	0.73
			Median [Q1, Q3]	0.01 [-0.04, 0.07]	0.02 [-0.05, 0.08]	0.86
			Min, Max	-0.33, 0.25	-0.86, 0.63	
			One-sample t-test	0.57	0.43	
			Wilcoxon signed-rank test	0.38	0.29	
		24 週	n	61	61	
			Mean±SD	0.02±0.17	0.02±0.14	0.90
			Median [Q1, Q3]	-0.01 [-0.05, 0.06]	0.03 [-0.06, 0.10]	0.29
			Min, Max	-0.30, 0.74	-0.35, 0.42	
			One-sample t-test	0.39	0.22	
			Wilcoxon signed-rank test	0.92	0.18	
クレアチニン・クリアランス (mL/min)	測定値	0 週	n	63	65	
			Mean±SD	63.0±31.4	67.5±30.0	0.41
			Median [Q1, Q3]	55.9 [42.8, 79.5]	61.4 [45.7, 78.1]	0.22
			Min, Max	25.8, 204.0	31.4, 164.6	
		12 週	n	58	61	
			Mean±SD	60.5±30.2	66.3±31.9	0.31
			Median [Q1, Q3]	52.2 [41.0, 71.4]	57.1 [43.4, 82.1]	0.32
			Min, Max	21.6, 213.3	19.3, 182.2	
		24 週	n	60	62	
			Mean±SD	62.8±32.7	65.3±31.2	0.67
			Median [Q1, Q3]	55.9 [42.1, 75.5]	56.6 [40.6, 81.7]	0.67
			Min, Max	21.3, 216.7	27.3, 172.4	
	変化量	12 週	n	58	59	
			Mean±SD	-0.7±5.3	-0.1±10.6	0.69
			Median [Q1, Q3]	-1.0 [-4.7, 1.7]	-1.7 [-6.2, 5.8]	0.95
			Min, Max	-12.4, 12.2	-24.4, 45.6	
			One-sample t-test	0.30	0.94	
			Wilcoxon signed-rank test	0.16	0.60	
		24 週	n	59	60	
			Mean±SD	0.5±7.5	-1.0±8.9	0.32
			Median [Q1, Q3]	0.2 [-2.8, 3.4]	-2.7 [-7.1, 4.2]	0.16
			Min, Max	-18.6, 30.6	-26.4, 24.4	
			One-sample t-test	0.63	0.37	
			Wilcoxon signed-rank test	0.70	0.20	
eGFR (mL/min/1.73m^2)	測定値	0 週	n	63	65	
			Mean±SD	53.5±15.0	56.7±16.9	0.25
			Median [Q1, Q3]	50.9 [43.1, 62.4]	54.0 [43.8, 66.8]	0.36
			Min, Max	29.4, 94.2	30.1, 98.3	
		12 週	n	63	66	
			Mean±SD	53.5±14.8	55.9±17.6	0.41
			Median [Q1, Q3]	51.1 [43.3, 64.6]	52.9 [40.1, 69.2]	0.62
			Min, Max	26.7, 95.7	27.3, 99.6	
		24 週	n	62	63	
			Mean±SD	53.8±16.0	55.5±16.8	0.56
			Median [Q1, Q3]	52.1 [43.4, 64.3]	52.6 [41.5, 68.5]	0.69
			Min, Max	23.1, 95.0	27.2, 93.0	

	変化量	12 週	n	62	64	
			Mean±SD	-0.2±5.4	-0.1±8.5	0.94
			Median [Q1, Q3]	-0.5 [-3.8, 2.8]	-1.4 [-5.2, 4.3]	0.88
			Min, Max	-14.6, 16.2	-17.0, 40.5	
			One-sample t-test	0.77	0.93	
			Wilcoxon signed-rank test	0.57	0.61	
		24 週	n	61	61	
			Mean±SD	0.1±7.5	-0.5±6.9	0.69
			Median [Q1, Q3]	0.8 [-3.7, 3.7]	-1.8 [-5.3, 4.0]	0.29
			Min, Max	-22.1, 28.3	-14.4, 17.6	
			One-sample t-test	0.96	0.60	
			Wilcoxon signed-rank test	0.80	0.39	
尿中クレアチン (mg/dl)	測定値	0 週	n	54	55	
			Mean±SD	99.5±56.7	105.9±59.1	0.57
			Median [Q1, Q3]	91.0 [62.7, 138.0]	96.0 [60.0, 155.0]	0.52
			Min, Max	14.0, 252.0	16.0, 277.0	
		12 週	n	52	55	
			Mean±SD	99.3±65.9	92.7±69.3	0.61
			Median [Q1, Q3]	90.0 [51.9, 123.5]	73.0 [37.0, 123.0]	0.45
			Min, Max	11.0, 334.0	7.0, 344.0	
		24 週	n	52	53	
			Mean±SD	101.6±83.7	87.4±50.7	0.29
			Median [Q1, Q3]	88.0 [50.5, 132.0]	83.0 [44.0, 130.0]	0.68
			Min, Max	14.8, 478.0	15.0, 238.0	
	変化量	12 週	n	52	54	
			Mean±SD	-1.4±76.8	-14.5±76.0	0.38
			Median [Q1, Q3]	6.1 [-36.8, 35.5]	-9.0 [-56.0, 32.0]	0.39
			Min, Max	-197.0, 280.0	-214.0, 158.0	
			One-sample t-test	0.90	0.17	
			Wilcoxon signed-rank test	0.99	0.22	
		24 週	n	52	52	
			Mean±SD	0.5±82.1	-15.4±60.0	0.26
			Median [Q1, Q3]	-14.0 [-47.4, 34.0]	-12.0 [-43.0, 20.0]	0.51
			Min, Max	-180.0, 328.0	-206.0, 120.0	
			One-sample t-test	0.96	0.07	
			Wilcoxon signed-rank test	0.52	0.08	
尿中浸透圧 (mOsm/Kg・H2O)	測定値	0 週	n	53	56	
			Mean±SD	515.7±193.4	554.7±197.7	0.30
			Median [Q1, Q3]	521.0 [405.0, 657.0]	586.0 [408.5, 699.5]	0.31
			Min, Max	163.0, 946.0	188.0, 1018.0	
		12 週	n	51	55	
			Mean±SD	494.1±154.9	518.5±189.2	0.47
			Median [Q1, Q3]	506.0 [394.0, 598.0]	514.0 [358.0, 652.0]	0.52
			Min, Max	144.5, 987.0	192.0, 1130.0	
		24 週	n	52	52	
			Mean±SD	531.7±195.8	493.9±194.8	0.33
			Median [Q1, Q3]	550.0 [402.0, 681.5]	476.5 [352.5, 626.0]	0.37
			Min, Max	174.0, 1039.0	130.0, 863.0	
	変化量	12 週	n	50	55	

			Mean±SD	−25.4±188.7	−37.9±205.9	0.75
			Median [Q1, Q3]	−22.0 [−148.0, 92.0]	−62.0 [−178.0, 80.0]	0.57
			Min, Max	−503.5, 432.0	−475.0, 594.0	
			One-sample t-test	0.35	0.18	
			Wilcoxon signed-rank test	0.27	0.10	
		24 週	n	51	52	
			Mean±SD	8.8±189.7	−55.3±161.0	0.07
			Median [Q1, Q3]	−8.0 [−125.0, 147.0]	−37.0 [−116.5, 11.5]	0.10
			Min, Max	−381.0, 556.0	−614.0, 447.0	
			One-sample t-test	0.74	0.017	
			Wilcoxon signed-rank test	0.97	0.005	

表 5.2.9. [PPS] 心臓超音波検査パラメーター

変数		観察ポイント	統計量	トピロキソスタット群	アロプリノール群	群間比較 P 値
左室駆出率 (EF) (M.simpson 法) (%)	測定値	0 週	n	64	65	
			Mean±SD	53.1±12.6	51.2±13.0	0.40
			Median [Q1, Q3]	55.0 [44.0, 61.9]	55.0 [44.0, 61.0]	0.50
			Min, Max	25.2, 74.0	19.3, 71.4	
		24 週	n	60	62	
			Mean±SD	53.3±12.5	52.7±13.8	0.79
			Median [Q1, Q3]	55.8 [43.2, 62.8]	54.9 [44.4, 61.5]	0.80
			Min, Max	20.7, 75.0	21.8, 77.7	
	変化量	24 週	n	60	61	
			Mean±SD	-0.1±5.5	1.4±5.2	0.13
			Median [Q1, Q3]	-0.3 [-3.9, 3.0]	1.0 [-2.5, 4.0]	0.12
			Min, Max	-12.0, 12.1	-10.0, 17.0	
			One-sample t-test	0.94	0.039	
			Wilcoxon signed-rank test	0.81	0.09	
左室拡張末期径(LVDd) (mm)	測定値	0 週	n	62	67	
			Mean±SD	52.8±8.2	53.0±8.8	0.89
			Median [Q1, Q3]	51.4 [47.3, 56.1]	51.7 [46.8, 57.4]	0.85
			Min, Max	38.1, 78.4	36.0, 76.2	
		24 週	n	59	63	
			Mean±SD	51.3±8.0	52.3±9.8	0.54
			Median [Q1, Q3]	50.2 [47.3, 55.3]	51.4 [47.1, 56.4]	0.49
			Min, Max	31.5, 72.4	10.5, 77.8	
	変化量	24 週	n	57	63	
			Mean±SD	-0.8±5.9	-1.0±6.6	0.91
			Median [Q1, Q3]	-0.8 [-3.8, 2.4]	-0.5 [-3.5, 1.9]	0.94
			Min, Max	-20.7, 16.2	-36.3, 16.0	
			One-sample t-test	0.30	0.26	
			Wilcoxon signed-rank test	0.31	0.33	
左室収縮末期径(LVDs) (mm)	測定値	0 週	n	58	65	
			Mean±SD	38.2±10.5	38.0±11.1	0.94
			Median [Q1, Q3]	35.8 [30.9, 46.1]	36.1 [31.0, 43.2]	1.00
			Min, Max	20.6, 68.2	12.7, 70.7	
		24 週	n	58	60	
			Mean±SD	36.6±9.2	38.2±11.1	0.39
			Median [Q1, Q3]	35.7 [29.7, 41.6]	36.4 [31.7, 45.2]	0.53
			Min, Max	20.0, 56.4	7.9, 69.9	
	変化量	24 週	n	52	58	
			Mean±SD	-0.5±5.3	-0.1±8.0	0.77
			Median [Q1, Q3]	0.0 [-3.3, 2.8]	-0.8 [-2.8, 3.0]	0.99
			Min, Max	-17.2, 15.6	-26.6, 39.3	
			One-sample t-test	0.49	0.91	
			Wilcoxon signed-rank test	0.54	0.48	
僧帽弁口血流速波形(E) (cm/sec)	測定値	0 週	n	64	67	
			Mean±SD	80.4±41.3	75.0±37.7	0.44
			Median [Q1, Q3]	72.6 [55.1, 92.0]	64.3 [51.0, 87.9]	0.33
			Min, Max	37.3, 268.0	26.4, 214.8	

		24 週	n	61	63	
			Mean±SD	76.9±38.2	82.3±41.1	0.45
			Median [Q1, Q3]	68.5 [55.1, 85.9]	77.5 [53.3, 98.2]	0.48
			Min, Max	25.3, 241.0	31.0, 245.5	
	変化量	24 週	n	61	63	
			Mean±SD	-3.4±17.0	6.1±22.0	0.008
			Median [Q1, Q3]	-1.0 [-10.9, 6.1]	4.0 [-7.5, 14.8]	0.018
			Min, Max	-48.9, 44.2	-42.8, 96.1	
			One-sample t-test	0.12	0.031	
			Wilcoxon signed-rank test	0.14	0.07	
僧帽弁口血流速波形(A)(cm/sec)	測定値	0 週	n	32	40	
			Mean±SD	67.6±24.8	74.4±26.1	0.27
			Median [Q1, Q3]	71.4 [52.5, 87.7]	69.2 [58.5, 84.5]	0.65
			Min, Max	21.6, 113.0	30.9, 181.0	
		24 週	n	33	35	
			Mean±SD	62.1±25.5	72.9±24.3	0.08
			Median [Q1, Q3]	64.7 [50.7, 80.7]	68.3 [57.3, 86.9]	0.13
			Min, Max	14.6, 119.0	26.9, 122.0	
	変化量	24 週	n	31	34	
			Mean±SD	-4.2±17.9	-3.8±21.1	0.93
			Median [Q1, Q3]	-3.2 [-13.8, 5.4]	-3.5 [-9.7, 4.1]	0.72
			Min, Max	-64.3, 48.4	-100.0, 42.0	
			One-sample t-test	0.20	0.31	
			Wilcoxon signed-rank test	0.11	0.28	
僧帽弁口血流速波形(DT)(msec)	測定値	0 週	n	62	66	
			Mean±SD	202.6±49.9	222.2±67.2	0.06
			Median [Q1, Q3]	189.4 [174.1, 224.0]	209.5 [175.0, 263.5]	0.15
			Min, Max	105.6, 375.0	100.0, 400.0	
		24 週	n	60	63	
			Mean±SD	209.7±74.9	227.8±86.5	0.22
			Median [Q1, Q3]	184.0 [160.0, 240.0]	200.0 [160.0, 258.0]	0.34
			Min, Max	24.0, 531.7	104.0, 544.0	
	変化量	24 週	n	58	62	
			Mean±SD	-0.1±51.6	-0.1±58.2	1.00
			Median [Q1, Q3]	0.0 [-27.9, 33.0]	-3.8 [-32.0, 33.0]	0.73
			Min, Max	-160.0, 165.0	-136.0, 156.2	
			One-sample t-test	0.98	0.99	
			Wilcoxon signed-rank test	0.80	0.85	
左室弁輪運動速波形(E/e')	測定値	0 週	n	53	65	
			Mean±SD	13.6±6.5	12.9±7.2	0.60
			Median [Q1, Q3]	12.7 [9.3, 15.8]	11.7 [8.5, 14.5]	0.41
			Min, Max	4.4, 38.2	3.6, 46.2	
		24 週	n	58	61	
			Mean±SD	12.0±6.0	13.2±6.3	0.30
			Median [Q1, Q3]	10.5 [8.6, 13.6]	11.9 [9.2, 15.3]	0.18
			Min, Max	4.9, 36.5	4.4, 34.5	
	変化量	24 週	n	49	59	
			Mean±SD	-1.3±4.6	0.4±5.5	0.08
			Median [Q1, Q3]	-0.9 [-4.1, 1.3]	0.8 [-3.2, 4.6]	0.07

			Min, Max	−10.5, 9.3	−11.7, 13.9	
			One-sample t-test	0.046	0.56	
			Wilcoxon signed-rank test	0.06	0.55	
下大静脈径 (IVCd) (mm)	測定値	0 週	n	62	62	
			Mean±SD	14.6±5.6	14.0±4.1	0.49
			Median [Q1, Q3]	14.1 [11.4, 16.8]	13.6 [10.8, 16.7]	0.65
			Min, Max	4.2, 43.0	3.2, 27.0	
		24 週	n	60	61	
			Mean±SD	15.9±6.5	15.4±6.0	0.69
			Median [Q1, Q3]	14.9 [12.2, 17.8]	14.9 [12.1, 17.2]	0.81
			Min, Max	8.0, 49.2	7.8, 51.2	
	変化量	24 週	n	58	57	
			Mean±SD	1.1±5.9	1.2±6.3	0.97
			Median [Q1, Q3]	0.8 [−3.2, 3.6]	0.9 [−1.5, 3.4]	0.89
			Min, Max	−6.8, 32.4	−10.0, 36.5	
			One-sample t-test	0.15	0.17	
			Wilcoxon signed-rank test	0.30	0.16	
左房短径 (LAD) (mm)	測定値	0 週	n	64	65	
			Mean±SD	45.2±7.2	43.3±8.0	0.16
			Median [Q1, Q3]	44.4 [39.7, 48.6]	42.3 [38.6, 47.6]	0.13
			Min, Max	30.9, 61.1	26.0, 64.8	
		24 週	n	61	63	
			Mean±SD	45.8±7.1	44.4±7.6	0.27
			Median [Q1, Q3]	44.9 [42.0, 49.3]	44.4 [38.1, 48.2]	0.22
			Min, Max	29.0, 66.8	31.8, 65.5	
	変化量	24 週	n	61	61	
			Mean±SD	0.9±4.7	1.0±5.5	0.87
			Median [Q1, Q3]	0.6 [−1.7, 3.1]	0.8 [−2.4, 4.6]	0.81
			Min, Max	−13.6, 14.6	−12.0, 20.6	
			One-sample t-test	0.16	0.16	
			Wilcoxon signed-rank test	0.10	0.13	
三尖弁圧較差 (TRPG) (mmHg)	測定値	0 週	n	56	52	
			Mean±SD	24.0±10.0	19.7±6.8	0.011
			Median [Q1, Q3]	22.4 [17.5, 28.3]	18.2 [16.1, 24.2]	0.008
			Min, Max	7.4, 70.3	4.0, 41.0	
		24 週	n	52	50	
			Mean±SD	23.9±7.4	22.5±9.1	0.39
			Median [Q1, Q3]	23.0 [19.1, 28.1]	20.6 [16.2, 27.7]	0.19
			Min, Max	10.0, 46.0	10.0, 51.0	
	変化量	24 週	n	49	43	
			Mean±SD	0.9±5.3	3.1±7.3	0.10
			Median [Q1, Q3]	0.0 [−1.8, 5.1]	2.0 [−1.0, 7.0]	0.14
			Min, Max	−12.1, 13.0	−13.4, 23.7	
			One-sample t-test	0.24	0.008	
			Wilcoxon signed-rank test	0.27	0.005	

表 5.2.10. [PPS] 特殊血液検査

変数		観察ポイント	統計量	トピロキソスタット群	アロプリノール群	群間比較P 値
hsCRP(mg/dL)	測定値	0 週	n	63	66	
			Mean±SD	0.14±0.16	0.11±0.13	0.28
			Median [Q1, Q3]	0.07 [0.03, 0.21]	0.06 [0.03, 0.14]	0.50
			Min, Max	0.00, 0.50	0.01, 0.50	
		12 週	n	60	62	
			Mean±SD	0.14±0.15	0.13±0.14	0.57
			Median [Q1, Q3]	0.08 [0.04, 0.19]	0.08 [0.03, 0.15]	0.88
			Min, Max	0.00, 0.50	0.01, 0.50	
		24 週	n	60	59	
			Mean±SD	0.15±0.16	0.13±0.14	0.30
			Median [Q1, Q3]	0.09 [0.05, 0.20]	0.07 [0.04, 0.16]	0.23
			Min, Max	0.01, 0.50	0.01, 0.50	
	変化量	12 週	n	59	61	
			Mean±SD	0.00±0.17	0.02±0.15	0.50
			Median [Q1, Q3]	0.00 [-0.02, 0.05]	0.01 [-0.03, 0.05]	0.81
			Min, Max	-0.44, 0.39	-0.36, 0.47	
			One-sample t-test	0.98	0.28	
			Wilcoxon signed-rank test	0.47	0.28	
		24 週	n	59	58	
			Mean±SD	0.03±0.16	0.01±0.13	0.51
			Median [Q1, Q3]	0.01 [-0.01, 0.07]	0.00 [-0.01, 0.03]	0.37
			Min, Max	-0.45, 0.49	-0.37, 0.41	
			One-sample t-test	0.17	0.53	
			Wilcoxon signed-rank test	0.028	0.17	
	変化率	12 週	n	59	61	
			Mean±SD	115.3±477.3	105.4±290.0	0.89
			Median [Q1, Q3]	1.1 [-41.9, 110.2]	21.1 [-35.3, 111.8]	0.61
			Min, Max	-90.2, 3266.7	-91.5, 1751.9	
			One-sample t-test	0.07	0.006	
			Wilcoxon signed-rank test	0.039	0.008	
		24 週	n	59	58	
			Mean±SD	167.5±560.2	54.9±127.8	0.14
			Median [Q1, Q3]	27.1 [-14.3, 140.7]	16.5 [-8.2, 87.2]	0.36
			Min, Max	-93.9, 4066.7	-90.1, 497.8	
			One-sample t-test	0.025	0.002	
			Wilcoxon signed-rank test	<0.001	0.003	
対数変換 hsCRP(ln (mg/dL))	測定値	0 週	n	63	66	
			Mean±SD	-2.61±1.21	-2.75±1.11	0.49
			Median [Q1, Q3]	-2.62 [-3.61, -1.58]	-2.87 [-3.58, -1.97]	0.50
			Min, Max	-5.52, -0.69	-4.96, -0.69	
		12 週	n	60	62	
			Mean±SD	-2.53±1.16	-2.57±1.05	0.86
			Median [Q1, Q3]	-2.59 [-3.32, -1.68]	-2.52 [-3.41, -1.90]	0.88
			Min, Max	-5.52, -0.69	-4.83, -0.69	
		24 週	n	60	59	
			Mean±SD	-2.38±1.08	-2.57±1.00	0.32

			Median [Q1, Q3]	-2.43 [-3.03, -1.63]	-2.70 [-3.19, -1.83]	0.23
			Min, Max	-5.12, -0.69	-4.42, -0.69	
	変化量	12 週	n	59	61	
			Mean±SD	0.10±1.01	0.20±0.99	0.59
			Median [Q1, Q3]	0.01 [-0.54, 0.74]	0.19 [-0.44, 0.75]	0.61
			Min, Max	-2.32, 3.52	-2.47, 2.92	
			One-sample t-test	0.45	0.12	
			Wilcoxon signed-rank test	0.44	0.11	
		24 週	n	59	58	
			Mean±SD	0.33±1.06	0.15±0.80	0.31
			Median [Q1, Q3]	0.24 [-0.15, 0.88]	0.15 [-0.09, 0.63]	0.36
			Min, Max	-2.80, 3.73	-2.31, 1.79	
			One-sample t-test	0.020	0.15	
			Wilcoxon signed-rank test	0.009	0.049	
	変化率	12 週	n	59	61	
			Mean±SD	14.9±75.4	3.5±55.8	0.35
			Median [Q1, Q3]	-0.4 [-26.2, 17.3]	-6.7 [-24.0, 17.9]	0.71
			Min, Max	-79.5, 303.5	-80.8, 236.2	
			One-sample t-test	0.13	0.62	
			Wilcoxon signed-rank test	0.90	0.48	
		24 週	n	59	58	
			Mean±SD	2.9±74.1	4.4±55.1	0.90
			Median [Q1, Q3]	-8.2 [-28.5, 7.2]	-4.9 [-17.9, 5.2]	0.37
			Min, Max	-84.3, 378.3	-71.2, 263.2	
			One-sample t-test	0.77	0.55	
			Wilcoxon signed-rank test	0.042	0.16	
シスタチンC (mg/L)	測定値	0 週	n	64	67	
			Mean±SD	1.19±0.27	1.16±0.33	0.64
			Median [Q1, Q3]	1.18 [0.99, 1.35]	1.08 [0.92, 1.33]	0.39
			Min, Max	0.70, 2.02	0.69, 2.08	
		12 週	n	61	65	
			Mean±SD	1.21±0.31	1.16±0.35	0.40
			Median [Q1, Q3]	1.19 [1.00, 1.33]	1.12 [0.92, 1.30]	0.21
			Min, Max	0.70, 2.03	0.63, 2.41	
		24 週	n	61	63	
			Mean±SD	1.20±0.32	1.20±0.36	0.98
			Median [Q1, Q3]	1.16 [0.98, 1.35]	1.14 [0.97, 1.36]	0.76
			Min, Max	0.73, 2.26	0.70, 2.32	
	変化量	12 週	n	61	65	
			Mean±SD	0.04±0.15	0.01±0.16	0.25
			Median [Q1, Q3]	0.03 [-0.02, 0.08]	-0.01 [-0.06, 0.07]	0.19
			Min, Max	-0.28, 0.55	-0.60, 0.49	
			One-sample t-test	0.043	0.68	
			Wilcoxon signed-rank test	0.034	0.72	
		24 週	n	61	63	
			Mean±SD	0.04±0.18	0.04±0.12	0.93
			Median [Q1, Q3]	0.03 [-0.03, 0.09]	0.04 [-0.03, 0.12]	0.50
			Min, Max	-0.32, 0.65	-0.38, 0.33	
			One-sample t-test	0.11	0.013	

			Wilcoxon signed-rank test	0.08	0.005	
	変化率	12 週	n	61	65	
			Mean±SD	3.4±11.0	1.0±12.3	0.26
			Median [Q1, Q3]	2.8 [-1.7, 7.4]	-0.9 [-5.2, 7.6]	0.20
			Min, Max	-16.0, 37.2	-42.3, 41.4	
			One-sample t-test	0.020	0.50	
			Wilcoxon signed-rank test	0.025	0.65	
		24 週	n	61	63	
			Mean±SD	3.0±12.9	3.4±10.0	0.86
			Median [Q1, Q3]	3.3 [-2.8, 9.1]	4.6 [-3.0, 12.1]	0.53
			Min, Max	-27.0, 40.4	-24.5, 20.9	
			One-sample t-test	0.07	0.009	
			Wilcoxon signed-rank test	0.06	0.006	
MDA-LDL (U/L)	測定値	0 週	n	64	67	
			Mean±SD	93.9±31.3	95.6±28.5	0.74
			Median [Q1, Q3]	96.0 [70.5, 112.5]	95.0 [71.0, 114.0]	0.84
			Min, Max	30.0, 167.0	51.0, 170.0	
		12 週	n	61	65	
			Mean±SD	89.9±34.9	86.0±27.0	0.48
			Median [Q1, Q3]	82.0 [67.0, 108.0]	80.0 [68.0, 101.0]	0.77
			Min, Max	30.0, 188.0	31.0, 182.0	
		24 週	n	61	63	
			Mean±SD	90.8±33.2	85.6±30.6	0.37
			Median [Q1, Q3]	87.0 [69.0, 104.0]	79.0 [63.0, 101.0]	0.29
			Min, Max	36.0, 196.0	35.0, 190.0	
	変化量	12 週	n	61	65	
			Mean±SD	-3.9±26.2	-10.1±25.2	0.18
			Median [Q1, Q3]	-7.0 [-20.0, 8.0]	-5.0 [-28.0, 6.0]	0.32
			Min, Max	-61.0, 74.0	-62.0, 46.0	
			One-sample t-test	0.25	0.002	
			Wilcoxon signed-rank test	0.09	0.004	
		24 週	n	61	63	
			Mean±SD	-3.0±26.4	-9.2±21.6	0.15
			Median [Q1, Q3]	-4.0 [-19.0, 16.0]	-8.0 [-24.0, 2.0]	0.13
			Min, Max	-71.0, 54.0	-70.0, 37.0	
			One-sample t-test	0.38	0.001	
			Wilcoxon signed-rank test	0.62	<0.001	
	変化率	12 週	n	61	65	
			Mean±SD	-0.8±30.5	-7.3±26.0	0.20
			Median [Q1, Q3]	-6.4 [-20.9, 11.1]	-6.0 [-27.5, 7.5]	0.32
			Min, Max	-46.2, 119.5	-47.5, 54.9	
			One-sample t-test	0.84	0.027	
			Wilcoxon signed-rank test	0.21	0.019	
		24 週	n	61	63	
			Mean±SD	1.5±32.1	-8.2±21.2	0.047
			Median [Q1, Q3]	-3.6 [-21.6, 20.0]	-7.5 [-20.0, 3.1]	0.11
			Min, Max	-48.3, 131.7	-56.5, 50.0	
			One-sample t-test	0.71	0.003	
			Wilcoxon signed-rank test	0.92	0.001	

5. 副次評価項目
5.2. PPS
特殊血液検査

XOR 活性 (pmol/h/mL plasma)	測定値	0 週	n	64	67	
			Mean±SD	57.9±55.0	67.6±128.1	0.57
			Median [Q1, Q3]	40.5 [26.1, 68.1]	29.4 [17.8, 57.5]	0.08
			Min, Max	8.0, 342.0	6.7, 946.0	
		24 週	n	61	63	
			Mean±SD	23.4±34.5	24.9±34.7	0.80
			Median [Q1, Q3]	11.8 [6.7, 27.4]	12.6 [6.8, 23.9]	0.70
			Min, Max	6.7, 250.0	6.7, 210.0	
	変化量	24 週	n	61	63	
			Mean±SD	−34.9±58.9	−43.0±114.7	0.62
			Median [Q1, Q3]	−21.9 [−44.3, −11.7]	−18.3 [−32.2, −8.2]	0.27
			Min, Max	−334.9, 182.2	−868.1, 65.0	
			One-sample t-test	<0.001	0.004	
			Wilcoxon signed-rank test	<0.001	<0.001	
	変化率	24 週	n	61	63	
			Mean±SD	−50.8±54.2	−46.4±40.9	0.61
			Median [Q1, Q3]	−66.5 [−78.8, −50.6]	−55.7 [−73.4, −36.4]	0.06
			Min, Max	−97.9, 268.7	−91.8, 130.0	
			One-sample t-test	<0.001	<0.001	
			Wilcoxon signed-rank test	<0.001	<0.001	
対数変換 XOR 活性(ln (pmol/h/mL plasma))	測定値	0 週	n	64	67	
			Mean±SD	3.8±0.7	3.6±1.0	0.19
			Median [Q1, Q3]	3.7 [3.3, 4.2]	3.4 [2.9, 4.1]	0.08
			Min, Max	2.1, 5.8	1.9, 6.9	
		24 週	n	61	63	
			Mean±SD	2.7±0.8	2.7±0.9	0.75
			Median [Q1, Q3]	2.5 [1.9, 3.3]	2.5 [1.9, 3.2]	0.70
			Min, Max	1.9, 5.5	1.9, 5.3	
	変化量	24 週	n	61	63	
			Mean±SD	−1.1±0.8	−0.8±0.7	0.10
			Median [Q1, Q3]	−1.1 [−1.5, −0.7]	−0.8 [−1.3, −0.5]	0.06
			Min, Max	−3.9, 1.3	−2.5, 0.8	
			One-sample t-test	<0.001	<0.001	
			Wilcoxon signed-rank test	<0.001	<0.001	
	変化率	24 週	n	61	63	
			Mean±SD	−27.5±20.2	−22.4±17.1	0.13
			Median [Q1, Q3]	−31.3 [−43.6, −17.2]	−23.7 [−34.5, −13.1]	0.07
			Min, Max	−66.3, 30.9	−55.8, 26.6	
			One-sample t-test	<0.001	<0.001	
			Wilcoxon signed-rank test	<0.001	<0.001	

表 5.2.11. [PPS] 特殊尿検査

変数		観察ポイント	統計量	トピロキソスタット群	アロプリノール群	群間比較 P 値
8-OHdG (ng/mg・Cr)	測定値	0 週	n	64	67	
			Mean±SD	7.8±3.6	7.3±4.0	0.46
			Median [Q1, Q3]	7.3 [5.4, 9.4]	6.7 [4.8, 9.0]	0.25
			Min, Max	2.9, 22.3	2.1, 27.8	
		12 週	n	61	65	
			Mean±SD	8.9±3.8	10.2±5.5	0.13
			Median [Q1, Q3]	8.1 [6.5, 10.2]	9.1 [7.0, 11.5]	0.13
			Min, Max	3.6, 20.7	4.3, 37.9	
		24 週	n	61	63	
			Mean±SD	8.5±4.2	10.3±4.6	0.028
			Median [Q1, Q3]	8.0 [5.7, 9.6]	9.4 [7.6, 12.0]	0.006
			Min, Max	1.5, 24.9	3.6, 29.0	
	変化量	12 週	n	61	65	
			Mean±SD	1.0±3.4	2.8±4.6	0.012
			Median [Q1, Q3]	1.0 [-0.5, 2.4]	2.6 [0.3, 4.6]	0.002
			Min, Max	-7.4, 12.7	-10.1, 27.3	
			One-sample t-test	0.034	<0.001	
			Wilcoxon signed-rank test	0.008	<0.001	
		24 週	n	61	63	
			Mean±SD	0.8±3.7	3.1±3.1	<0.001
			Median [Q1, Q3]	0.9 [-1.2, 2.3]	3.0 [1.1, 5.0]	<0.001
			Min, Max	-9.9, 11.9	-2.3, 10.6	
			One-sample t-test	0.09	<0.001	
			Wilcoxon signed-rank test	0.049	<0.001	
	変化率	12 週	n	61	65	
			Mean±SD	21.3±44.5	53.3±66.2	0.002
			Median [Q1, Q3]	15.1 [-5.7, 39.6]	45.8 [5.6, 79.3]	0.003
			Min, Max	-61.7, 158.8	-43.0, 275.0	
			One-sample t-test	<0.001	<0.001	
			Wilcoxon signed-rank test	<0.001	<0.001	
		24 週	n	61	63	
			Mean±SD	19.5±52.5	60.6±67.3	<0.001
			Median [Q1, Q3]	13.2 [-16.4, 39.0]	41.9 [12.7, 97.3]	<0.001
			Min, Max	-83.0, 197.6	-25.0, 296.4	
			One-sample t-test	0.005	<0.001	
			Wilcoxon signed-rank test	0.009	<0.001	
尿中 L-FABP (濃度) (ng/mL)	測定値	0 週	n	64	67	
			Mean±SD	4.0±4.1	5.2±7.1	0.26
			Median [Q1, Q3]	2.3 [1.6, 3.9]	2.3 [1.6, 5.5]	0.55
			Min, Max	1.5, 21.5	1.5, 36.3	
		12 週	n	61	65	
			Mean±SD	3.5±3.2	4.9±7.6	0.18
			Median [Q1, Q3]	2.0 [1.5, 3.7]	2.2 [1.5, 5.0]	0.28
			Min, Max	1.5, 12.8	1.5, 48.8	
		24 週	n	61	63	
			Mean±SD	4.0±5.0	5.4±8.3	0.27

			Median [Q1, Q3]	2.4 [1.5, 4.1]	2.0 [1.5, 6.7]	0.97
			Min, Max	1.5, 33.3	1.5, 51.8	
	変化量	12 週	n	61	65	
			Mean±SD	-0.5±3.7	0.3±7.1	0.46
			Median [Q1, Q3]	0.0 [-0.8, 0.5]	0.0 [-0.6, 0.9]	0.62
			Min, Max	-13.4, 10.5	-26.5, 32.6	
			One-sample t-test	0.32	0.76	
			Wilcoxon signed-rank test	0.55	0.90	
		24 週	n	61	63	
			Mean±SD	0.4±4.3	0.3±7.2	0.94
			Median [Q1, Q3]	0.0 [-0.6, 1.1]	0.0 [-0.9, 0.9]	0.73
			Min, Max	-9.8, 21.9	-28.3, 33.4	
			One-sample t-test	0.44	0.70	
			Wilcoxon signed-rank test	0.51	0.83	
	変化率	12 週	n	61	65	
			Mean±SD	17.5±99.7	32.1±119.2	0.46
			Median [Q1, Q3]	0.0 [-25.2, 33.3]	0.0 [-23.1, 40.7]	0.56
			Min, Max	-89.9, 552.6	-85.1, 581.3	
			One-sample t-test	0.18	0.033	
			Wilcoxon signed-rank test	0.80	0.31	
		24 週	n	61	63	
			Mean±SD	31.7±103.6	54.0±255.4	0.53
			Median [Q1, Q3]	0.0 [-20.8, 51.5]	0.0 [-30.9, 40.0]	0.57
			Min, Max	-81.9, 416.7	-85.2, 1855.6	
			One-sample t-test	0.020	0.10	
			Wilcoxon signed-rank test	0.16	0.51	
対数変換 尿中L-FABP(濃度)(ln(ng/mL))	測定値	0 週	n	64	67	
			Mean±SD	1.06±0.74	1.17±0.86	0.45
			Median [Q1, Q3]	0.83 [0.44, 1.36]	0.83 [0.47, 1.70]	0.55
			Min, Max	0.41, 3.07	0.41, 3.59	
		12 週	n	61	65	
			Mean±SD	0.96±0.70	1.11±0.83	0.26
			Median [Q1, Q3]	0.69 [0.41, 1.31]	0.79 [0.41, 1.61]	0.28
			Min, Max	0.41, 2.55	0.41, 3.89	
		24 週	n	61	63	
			Mean±SD	1.05±0.71	1.15±0.90	0.52
			Median [Q1, Q3]	0.88 [0.41, 1.41]	0.69 [0.41, 1.90]	0.97
			Min, Max	0.41, 3.51	0.41, 3.95	
	変化量	12 週	n	61	65	
			Mean±SD	-0.08±0.71	0.01±0.74	0.53
			Median [Q1, Q3]	0.00 [-0.29, 0.29]	0.00 [-0.26, 0.34]	0.56
			Min, Max	-2.30, 1.88	-1.91, 1.92	
			One-sample t-test	0.41	0.95	
			Wilcoxon signed-rank test	0.59	0.83	
		24 週	n	61	63	
			Mean±SD	0.05±0.67	0.02±0.80	0.79
			Median [Q1, Q3]	0.00 [-0.23, 0.42]	0.00 [-0.37, 0.34]	0.57
			Min, Max	-1.71, 1.64	-1.91, 2.97	
			One-sample t-test	0.54	0.87	

			Wilcoxon signed-rank test	0.47	0.88	
	変化率	12 週	n	61	65	
			Mean±SD	12.2±75.9	19.4±74.7	0.59
			Median [Q1, Q3]	0.0 [-31.0, 39.8]	0.0 [-24.9, 47.5]	0.54
			Min, Max	-85.0, 339.8	-82.5, 260.0	
			One-sample t-test	0.21	0.041	
			Wilcoxon signed-rank test	0.61	0.22	
		24 週	n	61	63	
			Mean±SD	28.6±91.2	24.1±105.9	0.80
			Median [Q1, Q3]	0.0 [-23.6, 45.0]	0.0 [-29.6, 36.4]	0.48
			Min, Max	-80.8, 396.9	-82.5, 505.8	
			One-sample t-test	0.017	0.08	
			Wilcoxon signed-rank test	0.15	0.62	
尿中 L-FABP (Cre 補正値) (μg/g･Cr)	測定値	0 週	n	64	67	
			Mean±SD	5.1±5.0	5.2±6.1	0.95
			Median [Q1, Q3]	3.3 [2.0, 6.1]	3.3 [1.9, 5.7]	0.87
			Min, Max	0.8, 25.1	0.8, 33.4	
		12 週	n	61	65	
			Mean±SD	4.7±5.8	6.1±7.3	0.24
			Median [Q1, Q3]	2.7 [2.0, 4.8]	3.6 [2.3, 7.1]	0.043
			Min, Max	0.6, 39.1	1.2, 47.2	
		24 週	n	61	63	
			Mean±SD	5.9±9.6	6.6±7.1	0.64
			Median [Q1, Q3]	3.0 [2.1, 5.5]	3.5 [2.2, 9.0]	0.18
			Min, Max	0.8, 55.7	0.9, 37.5	
	変化量	12 週	n	61	65	
			Mean±SD	-0.2±5.1	1.2±4.2	0.09
			Median [Q1, Q3]	0.0 [-0.7, 0.8]	0.4 [-0.7, 2.1]	0.08
			Min, Max	-17.7, 20.5	-8.7, 18.8	
			One-sample t-test	0.78	0.022	
			Wilcoxon signed-rank test	0.78	0.034	
		24 週	n	61	63	
			Mean±SD	1.2±8.0	1.3±5.6	0.91
			Median [Q1, Q3]	-0.1 [-1.3, 1.0]	0.4 [-1.0, 2.3]	0.32
			Min, Max	-16.7, 38.0	-15.8, 29.6	
			One-sample t-test	0.25	0.07	
			Wilcoxon signed-rank test	0.92	0.10	
	変化率	12 週	n	61	65	
			Mean±SD	12.2±82.4	47.1±108.5	0.045
			Median [Q1, Q3]	0.0 [-24.6, 20.0]	10.0 [-19.3, 69.2]	0.05
			Min, Max	-84.7, 490.0	-65.8, 494.7	
			One-sample t-test	0.25	<0.001	
			Wilcoxon signed-rank test	0.88	0.002	
		24 週	n	61	63	
			Mean±SD	35.8±138.1	75.3±236.4	0.26
			Median [Q1, Q3]	-5.3 [-32.7, 50.0]	12.9 [-18.2, 77.8]	0.19
			Min, Max	-79.9, 866.7	-77.4, 1418.2	
			One-sample t-test	0.048	0.014	
			Wilcoxon signed-rank test	0.36	0.004	

対数変換 尿中 L-FABP(Cre 補正値)(ln (μg/g･Cr))	測定値	0 週	n	64	67	
			Mean±SD	1.29±0.81	1.27±0.81	0.92
			Median [Q1, Q3]	1.19 [0.67, 1.80]	1.19 [0.64, 1.74]	0.87
			Min, Max	-0.22, 3.22	-0.22, 3.51	
		12 週	n	61	65	
			Mean±SD	1.16±0.79	1.42±0.80	0.07
			Median [Q1, Q3]	0.99 [0.69, 1.57]	1.28 [0.83, 1.96]	0.043
			Min, Max	-0.51, 3.67	0.18, 3.85	
		24 週	n	61	63	
			Mean±SD	1.26±0.88	1.47±0.88	0.18
			Median [Q1, Q3]	1.10 [0.74, 1.70]	1.25 [0.79, 2.20]	0.18
			Min, Max	-0.22, 4.02	-0.11, 3.62	
	変化量	12 週	n	61	65	
			Mean±SD	-0.09±0.66	0.19±0.60	0.016
			Median [Q1, Q3]	0.00 [-0.28, 0.18]	0.10 [-0.21, 0.53]	0.05
			Min, Max	-1.88, 1.77	-1.07, 1.78	
			One-sample t-test	0.32	0.013	
			Wilcoxon signed-rank test	0.67	0.019	
		24 週	n	61	63	
			Mean±SD	0.03±0.71	0.20±0.76	0.21
			Median [Q1, Q3]	-0.05 [-0.40, 0.41]	0.12 [-0.20, 0.58]	0.19
			Min, Max	-1.60, 2.27	-1.49, 2.72	
			One-sample t-test	0.75	0.044	
			Wilcoxon signed-rank test	0.98	0.07	
	変化率	12 週	n	61	65	
			Mean±SD	32.2±153.1	64.1±256.1	0.40
			Median [Q1, Q3]	0.0 [-24.0, 25.4]	7.5 [-17.9, 49.4]	0.21
			Min, Max	-84.7, 902.5	-349.0, 1454.5	
			One-sample t-test	0.11	0.048	
			Wilcoxon signed-rank test	0.85	0.06	
		24 週	n	61	63	
			Mean±SD	35.0±185.9	49.1±455.6	0.82
			Median [Q1, Q3]	-5.0 [-30.4, 43.3]	4.6 [-23.2, 49.4]	0.40
			Min, Max	-181.7, 1244.3	-1173.8, 2853.9	
			One-sample t-test	0.15	0.40	
			Wilcoxon signed-rank test	0.76	0.14	
尿中アルブミン (アルブミン)(μg/mL)	測定値	0 週	n	64	67	
			Mean±SD	101.4±256.1	172.2±675.3	0.43
			Median [Q1, Q3]	12.9 [5.3, 48.7]	18.9 [6.2, 67.2]	0.28
			Min, Max	0.5, 1402.6	0.6, 4566.9	
		12 週	n	61	66	
			Mean±SD	92.2±329.3	117.7±278.1	0.64
			Median [Q1, Q3]	8.9 [4.4, 35.9]	17.9 [6.4, 55.8]	0.10
			Min, Max	0.5, 2209.6	0.6, 1628.5	
		24 週	n	61	63	
			Mean±SD	54.1±143.8	151.5±712.2	0.30
			Median [Q1, Q3]	12.2 [5.6, 31.6]	16.5 [4.3, 40.8]	0.50
			Min, Max	0.7, 1024.8	0.8, 5627.4	
	変化量	12 週	n	61	66	

			Mean±SD	-11.9±284.3	-55.9±615.3	0.61
			Median [Q1, Q3]	-1.3 [-6.2, 1.7]	-2.7 [-14.1, 7.8]	0.91
			Min, Max	-1102.5, 1630.7	-4135.9, 1401.0	
			One-sample t-test	0.74	0.46	
			Wilcoxon signed-rank test	0.19	0.35	
		24 週	n	61	63	
			Mean±SD	-40.4±217.0	-26.3±635.8	0.87
			Median [Q1, Q3]	0.0 [-8.6, 5.6]	-1.9 [-11.1, 5.3]	0.82
			Min, Max	-1079.0, 445.9	-4202.7, 2500.1	
			One-sample t-test	0.15	0.74	
			Wilcoxon signed-rank test	0.46	0.29	
	変化率	12 週	n	61	66	
			Mean±SD	40.2±224.5	112.0±370.8	0.19
			Median [Q1, Q3]	-14.0 [-43.4, 45.5]	-14.5 [-66.0, 105.3]	0.64
			Min, Max	-99.4, 1565.5	-95.7, 2425.0	
			One-sample t-test	0.17	0.017	
			Wilcoxon signed-rank test	0.98	0.44	
		24 週	n	61	63	
			Mean±SD	69.2±238.2	76.0±274.3	0.88
			Median [Q1, Q3]	0.0 [-50.0, 56.6]	-14.1 [-61.9, 55.8]	0.63
			Min, Max	-93.4, 1118.6	-92.0, 1366.7	
			One-sample t-test	0.027	0.032	
			Wilcoxon signed-rank test	0.56	0.92	
対数変換 尿中アルブミン(アルブミン)(ln(μg/mL))	測定値	0 週	n	64	67	
			Mean±SD	2.82±1.80	3.08±1.79	0.40
			Median [Q1, Q3]	2.56 [1.67, 3.88]	2.94 [1.82, 4.21]	0.28
			Min, Max	-0.69, 7.25	-0.51, 8.43	
		12 週	n	61	66	
			Mean±SD	2.51±1.70	2.99±1.91	0.14
			Median [Q1, Q3]	2.19 [1.48, 3.58]	2.88 [1.86, 4.02]	0.10
			Min, Max	-0.69, 7.70	-0.51, 7.40	
		24 週	n	61	63	
			Mean±SD	2.63±1.56	2.90±1.76	0.37
			Median [Q1, Q3]	2.50 [1.72, 3.45]	2.80 [1.46, 3.71]	0.50
			Min, Max	-0.36, 6.93	-0.22, 8.64	
	変化量	12 週	n	61	66	
			Mean±SD	-0.26±1.22	-0.07±1.28	0.39
			Median [Q1, Q3]	-0.15 [-0.57, 0.37]	-0.16 [-1.08, 0.72]	0.64
			Min, Max	-5.11, 2.81	-3.15, 3.23	
			One-sample t-test	0.10	0.67	
			Wilcoxon signed-rank test	0.16	0.57	
		24 週	n	61	63	
			Mean±SD	-0.09±1.11	-0.11±1.10	0.89
			Median [Q1, Q3]	0.00 [-0.69, 0.45]	-0.15 [-0.96, 0.44]	0.63
			Min, Max	-2.72, 2.50	-2.53, 2.69	
			One-sample t-test	0.54	0.41	
			Wilcoxon signed-rank test	0.55	0.20	
	変化率	12 週	n	60	66	
			Mean±SD	7.9±73.6	9.6±253.3	0.96

			Median [Q1, Q3]	-5.8 [-24.1, 21.1]	-5.6 [-37.7, 21.5]	0.76
			Min, Max	-83.9, 306.9	-848.3, 1771.0	
			One-sample t-test	0.41	0.76	
			Wilcoxon signed-rank test	0.50	0.33	
		24 週	n	60	63	
			Mean±SD	22.5±145.5	-7.3±336.1	0.53
			Median [Q1, Q3]	-3.6 [-29.0, 22.1]	-6.6 [-29.8, 11.1]	0.47
			Min, Max	-95.0, 1054.1	-2226.7, 1294.0	
			One-sample t-test	0.24	0.86	
			Wilcoxon signed-rank test	0.91	0.15	
尿中アルブミン(クレアチニン)(mg/dL)	測定値	0 週	n	64	67	
			Mean±SD	98.6±57.4	107.4±57.2	0.38
			Median [Q1, Q3]	86.5 [56.1, 141.3]	98.4 [64.8, 152.1]	0.36
			Min, Max	7.2, 251.0	14.8, 273.2	
		12 週	n	61	66	
			Mean±SD	100.3±62.6	95.5±69.0	0.68
			Median [Q1, Q3]	85.5 [55.2, 132.0]	75.2 [41.9, 125.0]	0.45
			Min, Max	11.0, 329.8	6.7, 358.9	
		24 週	n	61	63	
			Mean±SD	104.0±81.6	86.6±49.0	0.15
			Median [Q1, Q3]	86.2 [55.4, 135.9]	83.0 [54.6, 116.2]	0.36
			Min, Max	13.8, 482.6	16.6, 283.0	
	変化量	12 週	n	61	66	
			Mean±SD	1.2±72.2	-10.9±71.3	0.35
			Median [Q1, Q3]	6.7 [-30.1, 39.7]	-3.0 [-48.5, 35.1]	0.34
			Min, Max	-193.9, 276.8	-204.5, 174.8	
			One-sample t-test	0.90	0.22	
			Wilcoxon signed-rank test	0.66	0.30	
		24 週	n	61	63	
			Mean±SD	5.2±77.0	-17.4±58.3	0.07
			Median [Q1, Q3]	0.5 [-29.8, 36.0]	-10.6 [-51.5, 11.9]	0.12
			Min, Max	-174.6, 333.3	-204.8, 131.0	
			One-sample t-test	0.60	0.021	
			Wilcoxon signed-rank test	0.95	0.030	
	変化率	12 週	n	61	66	
			Mean±SD	41.8±140.3	8.2±77.7	0.09
			Median [Q1, Q3]	7.6 [-36.0, 46.5]	-6.0 [-47.1, 42.0]	0.24
			Min, Max	-93.8, 553.3	-91.3, 320.3	
			One-sample t-test	0.023	0.39	
			Wilcoxon signed-rank test	0.21	0.96	
		24 週	n	61	63	
			Mean±SD	34.5±109.5	5.6±90.8	0.11
			Median [Q1, Q3]	1.1 [-25.3, 59.8]	-18.1 [-39.0, 20.7]	0.08
			Min, Max	-86.1, 492.5	-92.3, 519.8	
			One-sample t-test	0.017	0.62	
			Wilcoxon signed-rank test	0.19	0.14	
対数変換 尿中アルブミン(クレアチニン)(ln	測定値	0 週	n	64	67	
			Mean±SD	4.37±0.75	4.50±0.66	0.31
			Median [Q1, Q3]	4.46 [4.03, 4.95]	4.59 [4.17, 5.02]	0.36

(mg/dL))			Min, Max	1.97, 5.53	2.69, 5.61	
		12 週	n	61	66	
			Mean±SD	4.40±0.69	4.28±0.82	0.35
			Median [Q1, Q3]	4.45 [4.01, 4.88]	4.32 [3.73, 4.83]	0.45
			Min, Max	2.39, 5.80	1.89, 5.88	
		24 週	n	61	63	
			Mean±SD	4.40±0.71	4.29±0.64	0.33
			Median [Q1, Q3]	4.46 [4.01, 4.91]	4.42 [4.00, 4.76]	0.36
			Min, Max	2.62, 6.18	2.81, 5.65	
	変化量	12 週	n	61	66	
			Mean±SD	0.01±0.83	−0.21±0.85	0.14
			Median [Q1, Q3]	0.07 [−0.45, 0.38]	−0.06 [−0.64, 0.35]	0.24
			Min, Max	−2.78, 1.88	−2.44, 1.44	
			One-sample t-test	0.90	0.048	
			Wilcoxon signed-rank test	0.70	0.19	
		24 週	n	61	63	
			Mean±SD	0.03±0.75	−0.18±0.69	0.11
			Median [Q1, Q3]	0.01 [−0.29, 0.47]	−0.20 [−0.49, 0.19]	0.08
			Min, Max	−1.97, 1.78	−2.57, 1.82	
			One-sample t-test	0.76	0.045	
			Wilcoxon signed-rank test	0.68	0.025	
	変化率	12 週	n	61	66	
			Mean±SD	2.9±22.9	−3.4±19.1	0.09
			Median [Q1, Q3]	1.6 [−9.7, 8.9]	−1.5 [−16.1, 7.1]	0.24
			Min, Max	−53.7, 95.2	−56.3, 45.7	
			One-sample t-test	0.32	0.15	
			Wilcoxon signed-rank test	0.60	0.27	
		24 週	n	61	63	
			Mean±SD	3.0±20.7	−2.6±16.4	0.10
			Median [Q1, Q3]	0.3 [−6.5, 13.1]	−4.3 [−11.9, 4.4]	0.06
			Min, Max	−39.0, 81.3	−47.6, 58.1	
			One-sample t-test	0.26	0.21	
			Wilcoxon signed-rank test	0.47	0.044	
尿中アルブミン(クレアチニン補正値)(mg/g Cr)	測定値	0 週	n	63	67	
			Mean±SD	126.0±350.2	160.6±562.6	0.68
			Median [Q1, Q3]	12.3 [5.3, 83.4]	19.5 [6.5, 53.5]	0.54
			Min, Max	1.6, 2366.9	1.9, 3560.1	
		12 週	n	61	66	
			Mean±SD	100.7±394.1	172.1±571.4	0.42
			Median [Q1, Q3]	10.3 [4.9, 34.7]	18.7 [8.0, 61.4]	0.027
			Min, Max	1.3, 2920.0	1.6, 4259.3	
		24 週	n	61	63	
			Mean±SD	95.8±335.4	161.6±503.1	0.39
			Median [Q1, Q3]	14.6 [5.1, 35.0]	19.3 [5.9, 60.6]	0.19
			Min, Max	2.0, 2351.7	2.0, 3452.6	
	変化量	12 週	n	60	66	
			Mean±SD	−22.7±126.0	9.7±323.6	0.47
			Median [Q1, Q3]	−1.3 [−23.8, 2.7]	−0.3 [−7.0, 18.7]	0.09
			Min, Max	−567.9, 553.1	−2030.1, 1410.1	

			One-sample t-test	0.17	0.81	
			Wilcoxon signed-rank test	0.033	0.44	
		24 週	n	60	63	
			Mean±SD	-24.7±107.6	-6.0±277.2	0.63
			Median [Q1, Q3]	0.3 [-7.0, 4.7]	-0.6 [-8.0, 18.2]	0.73
			Min, Max	-552.6, 112.8	-1843.0, 652.1	
			One-sample t-test	0.08	0.86	
			Wilcoxon signed-rank test	0.67	0.94	
	変化率	12 週	n	60	66	
			Mean±SD	5.9±89.5	75.9±186.0	0.009
			Median [Q1, Q3]	-17.1 [-42.2, 24.2]	-3.8 [-38.6, 118.2]	0.048
			Min, Max	-92.6, 326.7	-79.8, 784.2	
			One-sample t-test	0.61	0.002	
			Wilcoxon signed-rank test	0.19	0.11	
		24 週	n	60	63	
			Mean±SD	25.5±119.6	113.9±370.8	0.08
			Median [Q1, Q3]	4.6 [-48.1, 26.7]	-6.4 [-45.3, 53.2]	0.70
			Min, Max	-93.4, 509.6	-88.4, 1790.8	
			One-sample t-test	0.10	0.018	
			Wilcoxon signed-rank test	0.99	0.55	
対数変換 尿中アルブミン(クレアチニン補正値)(ln(mg/g Cr))	測定値	0 週	n	63	67	
			Mean±SD	3.08±1.74	3.19±1.65	0.73
			Median [Q1, Q3]	2.51 [1.67, 4.42]	2.97 [1.87, 3.98]	0.54
			Min, Max	0.47, 7.77	0.64, 8.18	
		12 週	n	61	66	
			Mean±SD	2.71±1.60	3.32±1.71	0.041
			Median [Q1, Q3]	2.33 [1.59, 3.55]	2.93 [2.08, 4.12]	0.027
			Min, Max	0.26, 7.98	0.47, 8.36	
		24 週	n	61	63	
			Mean±SD	2.83±1.56	3.21±1.73	0.20
			Median [Q1, Q3]	2.68 [1.63, 3.56]	2.96 [1.77, 4.10]	0.19
			Min, Max	0.69, 7.76	0.69, 8.15	
	変化量	12 週	n	60	66	
			Mean±SD	-0.28±0.90	0.14±0.90	0.010
			Median [Q1, Q3]	-0.19 [-0.55, 0.22]	-0.04 [-0.49, 0.78]	0.048
			Min, Max	-2.61, 1.45	-1.60, 2.18	
			One-sample t-test	0.019	0.21	
			Wilcoxon signed-rank test	0.028	0.46	
		24 週	n	60	63	
			Mean±SD	-0.12±0.87	0.06±1.05	0.30
			Median [Q1, Q3]	0.04 [-0.66, 0.24]	-0.07 [-0.60, 0.43]	0.70
			Min, Max	-2.71, 1.81	-2.16, 2.94	
			One-sample t-test	0.29	0.64	
			Wilcoxon signed-rank test	0.44	0.81	
	変化率	12 週	n	60	66	
			Mean±SD	-5.0±34.1	11.8±48.1	0.026
			Median [Q1, Q3]	-7.2 [-27.5, 8.6]	-1.6 [-15.3, 26.6]	0.047
			Min, Max	-68.5, 132.1	-71.4, 196.2	
			One-sample t-test	0.26	0.0499	

			Wilcoxon signed-rank test	0.05	0.38	
		24 週	n	60	63	
			Mean±SD	2.3±30.9	12.2±60.4	0.26
			Median [Q1, Q3]	2.3 [-22.0, 16.8]	-2.7 [-16.3, 19.8]	0.97
			Min, Max	-50.3, 98.6	-63.0, 254.4	
			One-sample t-test	0.56	0.12	
			Wilcoxon signed-rank test	0.92	0.96	

6. 安全性評価項目

6.1. 有害事象

表 6.1.1. [安全性解析対象集団] 有害事象の発現件数

変数	発現件数	
	トピロキソスタット群<br>n = 70	アロプリノール群<br>n = 70
任意の有害事象	28	22
うっ血性心不全	2	1
てんかん	1	0
意識消失	0	1
壊死性筋膜炎	0	1
感情不安定	1	0
感冒	1	0
肝機能検査値上昇	4	5
胸水	0	1
血腫	1	0
高カリウム血症	1	0
高血糖	1	2
死亡	2	0
紫斑	1	0
徐脈	0	1
消化管出血	1	1
心不全	1	3
腎機能障害	3	2
脱水	1	0
痛風	1	1
転倒	1	0
動悸	1	0
尿管癌	1	0
肺炎	0	2
発疹	1	0
皮膚炎	0	1
便秘症	1	0
裂孔ヘルニア	1	0
重篤 / 任意の有害事象	9	8
重篤 / うっ血性心不全	1	1
重篤 / てんかん	1	0
重篤 / 意識消失	0	1
重篤 / 壊死性筋膜炎	0	1
重篤 / 感情不安定	0	0
重篤 / 感冒	0	0
重篤 / 肝機能検査値上昇	0	0
重篤 / 胸水	0	0
重篤 / 血腫	1	0
重篤 / 高カリウム血症	0	0
重篤 / 高血糖	0	0
重篤 / 死亡	2	0

重篤 / 紫斑	0	0
重篤 / 徐脈	0	0
重篤 / 消化管出血	1	1
重篤 / 心不全	1	2
重篤 / 腎機能障害	0	0
重篤 / 脱水	1	0
重篤 / 痛風	0	0
重篤 / 転倒	0	0
重篤 / 動悸	0	0
重篤 / 尿管癌	1	0
重篤 / 肺炎	0	2
重篤 / 発疹	0	0
重篤 / 皮膚炎	0	0
重篤 / 便秘症	0	0
重篤 / 裂孔ヘルニア	0	0

表 6.1.2. [安全性解析対象集団] 有害事象の発現例数と発現率(%)

変数	発現例数(%)		群間比較 P 値	
	トピロキソスタット群<br>n = 70	アロプリノール群<br>n = 70	χ 二乗<br>検定	Fisher<br>検定
死亡	2 (2.9)	0 (0.0)	0.15*	0.50
任意の有害事象	21 (30.0)	14 (20.0)	0.17	0.24
うっ血性心不全	2 (2.9)	1 (1.4)	0.56*	1.00
てんかん	1 (1.4)	0 (0.0)	0.32*	1.00
意識消失	0 (0.0)	1 (1.4)	0.32*	1.00
壊死性筋膜炎	0 (0.0)	1 (1.4)	0.32*	1.00
感情不安定	1 (1.4)	0 (0.0)	0.32*	1.00
感冒	1 (1.4)	0 (0.0)	0.32*	1.00
肝機能検査値上昇	4 (5.7)	5 (7.1)	0.73*	1.00
胸水	0 (0.0)	1 (1.4)	0.32*	1.00
血腫	1 (1.4)	0 (0.0)	0.32*	1.00
高カリウム血症	1 (1.4)	0 (0.0)	0.32*	1.00
高血糖	1 (1.4)	2 (2.9)	0.56*	1.00
死亡	2 (2.9)	0 (0.0)	0.15*	0.50
紫斑	1 (1.4)	0 (0.0)	0.32*	1.00
徐脈	0 (0.0)	1 (1.4)	0.32*	1.00
消化管出血	1 (1.4)	1 (1.4)	1.00*	1.00
心不全	1 (1.4)	3 (4.3)	0.31*	0.62
腎機能障害	3 (4.3)	2 (2.9)	0.65*	1.00
脱水	1 (1.4)	0 (0.0)	0.32*	1.00
痛風	1 (1.4)	1 (1.4)	1.00*	1.00
転倒	1 (1.4)	0 (0.0)	0.32*	1.00
動悸	1 (1.4)	0 (0.0)	0.32*	1.00
尿管癌	1 (1.4)	0 (0.0)	0.32*	1.00
肺炎	0 (0.0)	2 (2.9)	0.15*	0.50
発疹	1 (1.4)	0 (0.0)	0.32*	1.00
皮膚炎	0 (0.0)	1 (1.4)	0.32*	1.00
便秘症	1 (1.4)	0 (0.0)	0.32*	1.00
裂孔ヘルニア	1 (1.4)	0 (0.0)	0.32*	1.00
重篤 / 任意の有害事象	6 (8.6)	5 (7.1)	0.75	1.00
重篤 / うっ血性心不全	1 (1.4)	1 (1.4)	1.00*	1.00
重篤 / てんかん	1 (1.4)	0 (0.0)	0.32*	1.00
重篤 / 意識消失	0 (0.0)	1 (1.4)	0.32*	1.00
重篤 / 壊死性筋膜炎	0 (0.0)	1 (1.4)	0.32*	1.00
重篤 / 感情不安定	0 (0.0)	0 (0.0)	−	−
重篤 / 感冒	0 (0.0)	0 (0.0)	−	−
重篤 / 肝機能検査値上昇	0 (0.0)	0 (0.0)	−	−
重篤 / 胸水	0 (0.0)	0 (0.0)	−	−
重篤 / 血腫	1 (1.4)	0 (0.0)	0.32*	1.00
重篤 / 高カリウム血症	0 (0.0)	0 (0.0)	−	−
重篤 / 高血糖	0 (0.0)	0 (0.0)	−	−
重篤 / 死亡	2 (2.9)	0 (0.0)	0.15*	0.50
重篤 / 紫斑	0 (0.0)	0 (0.0)	−	−
重篤 / 徐脈	0 (0.0)	0 (0.0)	−	−
重篤 / 消化管出血	1 (1.4)	1 (1.4)	1.00*	1.00

重篤 / 心不全	1 (1.4)	2 (2.9)	0.56*	1.00
重篤 / 腎機能障害	0 (0.0)	0 (0.0)	-	-
重篤 / 脱水	1 (1.4)	0 (0.0)	0.32*	1.00
重篤 / 痛風	0 (0.0)	0 (0.0)	-	-
重篤 / 転倒	0 (0.0)	0 (0.0)	-	-
重篤 / 動悸	0 (0.0)	0 (0.0)	-	-
重篤 / 尿管癌	1 (1.4)	0 (0.0)	0.32*	1.00
重篤 / 肺炎	0 (0.0)	2 (2.9)	0.15*	0.50
重篤 / 発疹	0 (0.0)	0 (0.0)	-	-
重篤 / 皮膚炎	0 (0.0)	0 (0.0)	-	-
重篤 / 便秘症	0 (0.0)	0 (0.0)	-	-
重篤 / 裂孔ヘルニア	0 (0.0)	0 (0.0)	-	-

χ二乗検定のＰ値において、アスタリスクがついているものは、χ二乗検定の実施要件を満たしていないため、結果に信頼性がないことを示します。

6.2. 痛風関節炎(副作用)

6.2.1. FAS/安全性解析対象集団

FAS と安全性解析対象集団は同一集団となったため、以下まとめて結果を表記する。

表 6.2.1.1. [FAS/安全性解析対象集団] 痛風関節炎(副作用)の発現件数

変数	発現件数	
	トピロキソスタット群<br>n = 70	アロプリノール群<br>n = 70
因果関係の否定できない痛風関節炎	1	0
因果関係の否定できない痛風関節炎　軽度	1	0
因果関係の否定できない痛風関節炎　中等度	0	0
因果関係の否定できない痛風関節炎　高度	0	0

表 6.2.1.2. [FAS/安全性解析対象集団] 痛風関節炎(副作用)の発現例数と発現率(%)

変数	トピロキソスタット群<br>n = 70		アロプリノール群<br>n = 70		群間比較 P 値	
	発現例数<br>(%)	発現率の<br>95%CI	発現例数<br>(%)	発現率の<br>95%CI	χ 二乗<br>検定	Fisher<br>検定
因果関係の否定できない痛風関節炎	1 (1.4)	(0.0, 7.7)	0 (0.0)	(0.0, 5.1)	0.32*	1.00
因果関係の否定できない痛風関節炎　軽度	1 (1.4)	(0.0, 7.7)	0 (0.0)	(0.0, 5.1)	0.32*	1.00
因果関係の否定できない痛風関節炎　中等度	0 (0.0)	(0.0, 5.1)	0 (0.0)	(0.0, 5.1)	−	−
因果関係の否定できない痛風関節炎　高度	0 (0.0)	(0.0, 5.1)	0 (0.0)	(0.0, 5.1)	−	−

χ 二乗検定の P 値において、アスタリスクがついているものは、χ 二乗検定の実施要件を満たしていないため、結果に信頼性がないことを示します。

6.2.2. PPS

表 6.2.2.1. [PPS] 痛風関節炎(副作用)の発現件数

変数	発現件数	
	トピロキソスタット群 n = 64	アロプリノール群 n = 67
因果関係の否定できない痛風関節炎	1	0
因果関係の否定できない痛風関節炎 軽度	1	0
因果関係の否定できない痛風関節炎 中等度	0	0
因果関係の否定できない痛風関節炎 高度	0	0

表 6.2.2.2. [PPS] 痛風関節炎(副作用)の発現例数と発現率(%)

変数	トピロキソスタット群 n = 64		アロプリノール群 n = 67		群間比較 P 値	
	発現例数 (%)	発現率の 95%CI	発現例数 (%)	発現率の 95%CI	χ 二乗検定	Fisher 検定
因果関係の否定できない痛風関節炎	1 (1.6)	(0.0, 8.4)	0 (0.0)	(0.0, 5.4)	0.30*	0.49
因果関係の否定できない痛風関節炎 軽度	1 (1.6)	(0.0, 8.4)	0 (0.0)	(0.0, 5.4)	0.30*	0.49
因果関係の否定できない痛風関節炎 中等度	0 (0.0)	(0.0, 5.6)	0 (0.0)	(0.0, 5.4)	−	−
因果関係の否定できない痛風関節炎 高度	0 (0.0)	(0.0, 5.6)	0 (0.0)	(0.0, 5.4)	−	−

χ 二乗検定の P 値において、アスタリスクがついているものは、χ 二乗検定の実施要件を満たしていないため、結果に信頼性がないことを示します。

6.3. 肝機能検査値異常（副作用）

6.3.1. FAS/安全性解析対象集団

FAS と安全性解析対象集団は同一集団となったため、以下まとめて結果を表記する。

表 6.3.1.1. [FAS/安全性解析対象集団] 肝機能検査値異常(副作用)の発現件数

変数	発現件数	
	トピロキソスタット群 n = 70	アロプリノール群 n = 70
因果関係の否定できない肝機能検査値異常	1	1
因果関係の否定できない肝機能検査値異常 ALT	1	0
因果関係の否定できない肝機能検査値異常 ALT Category1	1	0
因果関係の否定できない肝機能検査値異常 ALT Category2	0	0
因果関係の否定できない肝機能検査値異常 ALT Category3	0	0
因果関係の否定できない肝機能検査値異常 AST	0	0
因果関係の否定できない肝機能検査値異常 AST Category1	0	0
因果関係の否定できない肝機能検査値異常 AST Category2	0	0
因果関係の否定できない肝機能検査値異常 AST Category3	0	0
因果関係の否定できない肝機能検査値異常 T-Bil	0	1
因果関係の否定できない肝機能検査値異常 T-Bil Category1	0	1
因果関係の否定できない肝機能検査値異常 T-Bil Category2	0	0
因果関係の否定できない肝機能検査値異常 T-Bil Category3	0	0

表 6.3.1.2. [FAS/安全性解析対象集団] 肝機能検査値異常(副作用)の発現例数と発現率(%)

変数	トピロキソスタット群 n = 70		アロプリノール群 n = 70		群間比較 P 値	
	発現例数(%)	発現率の95%CI	発現例数(%)	発現率の95%CI	χ 二乗検定	Fisher 検定
因果関係の否定できない肝機能検査値異常	1 (1.4)	(0.0, 7.7)	1 (1.4)	(0.0, 7.7)	1.00*	1.00
因果関係の否定できない肝機能検査値異常 ALT	1 (1.4)	(0.0, 7.7)	0 (0.0)	(0.0, 5.1)	0.32*	1.00
因果関係の否定できない肝機能検査値異常 ALT Category1	1 (1.4)	(0.0, 7.7)	0 (0.0)	(0.0, 5.1)	0.32*	1.00
因果関係の否定できない肝機能検査値異常 ALT Category2	0 (0.0)	(0.0, 5.1)	0 (0.0)	(0.0, 5.1)	−	−
因果関係の否定できない肝機能検査値異常 ALT Category3	0 (0.0)	(0.0, 5.1)	0 (0.0)	(0.0, 5.1)	−	−
因果関係の否定できない肝機能検査値異常 AST	0 (0.0)	(0.0, 5.1)	0 (0.0)	(0.0, 5.1)	−	−
因果関係の否定できない肝機能検査値異常 AST Category1	0 (0.0)	(0.0, 5.1)	0 (0.0)	(0.0, 5.1)	−	−
因果関係の否定できない肝機能検査値異常 AST Category2	0 (0.0)	(0.0, 5.1)	0 (0.0)	(0.0, 5.1)	−	−
因果関係の否定できない肝機能検査値異常 AST Category3	0 (0.0)	(0.0, 5.1)	0 (0.0)	(0.0, 5.1)	−	−
因果関係の否定できない肝機能検査値異常 T-Bil	0 (0.0)	(0.0, 5.1)	1 (1.4)	(0.0, 7.7)	0.32*	1.00
因果関係の否定できない肝機能検査値異常 T-Bil Category1	0 (0.0)	(0.0, 5.1)	1 (1.4)	(0.0, 7.7)	0.32*	1.00
因果関係の否定できない肝機能検査値異常 T-Bil Category2	0 (0.0)	(0.0, 5.1)	0 (0.0)	(0.0, 5.1)	−	−
因果関係の否定できない肝機能検査値異常 T-Bil Category3	0 (0.0)	(0.0, 5.1)	0 (0.0)	(0.0, 5.1)	−	−

χ 二乗検定の P 値において、アスタリスクがついているものは、χ 二乗検定の実施要件を満たしていないため、結果に信頼性がないことを示します。

6.3.2. PPS

表 6.3.2.1. [PPS] 肝機能検査値異常(副作用)の発現件数

変数	発現件数	
	トピロキソスタット群 n = 64	アロプリノール群 n = 67
因果関係の否定できない肝機能検査値異常	1	1
因果関係の否定できない肝機能検査値異常 ALT	1	0
因果関係の否定できない肝機能検査値異常 ALT Category1	1	0
因果関係の否定できない肝機能検査値異常 ALT Category2	0	0
因果関係の否定できない肝機能検査値異常 ALT Category3	0	0
因果関係の否定できない肝機能検査値異常 AST	0	0
因果関係の否定できない肝機能検査値異常 AST Category1	0	0
因果関係の否定できない肝機能検査値異常 AST Category2	0	0
因果関係の否定できない肝機能検査値異常 AST Category3	0	0
因果関係の否定できない肝機能検査値異常 T-Bil	0	1
因果関係の否定できない肝機能検査値異常 T-Bil Category1	0	1
因果関係の否定できない肝機能検査値異常 T-Bil Category2	0	0
因果関係の否定できない肝機能検査値異常 T-Bil Category3	0	0

表 6.3.2.2. [PPS] 肝機能検査値異常(副作用)の発現例数と発現率(%)

変数	トピロキソスタット群 n = 64		アロプリノール群 n = 67		群間比較 P 値	
	発現例数(%)	発現率の95%CI	発現例数(%)	発現率の95%CI	χ 二乗検定	Fisher 検定
因果関係の否定できない肝機能検査値異常	1 (1.6)	(0.0, 8.4)	1 (1.5)	(0.0, 8.0)	0.97*	1.00
因果関係の否定できない肝機能検査値異常 ALT	1 (1.6)	(0.0, 8.4)	0 (0.0)	(0.0, 5.4)	0.30*	0.49
因果関係の否定できない肝機能検査値異常 ALT Category1	1 (1.6)	(0.0, 8.4)	0 (0.0)	(0.0, 5.4)	0.30*	0.49
因果関係の否定できない肝機能検査値異常 ALT Category2	0 (0.0)	(0.0, 5.6)	0 (0.0)	(0.0, 5.4)	−	−
因果関係の否定できない肝機能検査値異常 ALT Category3	0 (0.0)	(0.0, 5.6)	0 (0.0)	(0.0, 5.4)	−	−
因果関係の否定できない肝機能検査値異常 AST	0 (0.0)	(0.0, 5.6)	0 (0.0)	(0.0, 5.4)	−	−
因果関係の否定できない肝機能検査値異常 AST Category1	0 (0.0)	(0.0, 5.6)	0 (0.0)	(0.0, 5.4)	−	−
因果関係の否定できない肝機能検査値異常 AST Category2	0 (0.0)	(0.0, 5.6)	0 (0.0)	(0.0, 5.4)	−	−
因果関係の否定できない肝機能検査値異常 AST Category3	0 (0.0)	(0.0, 5.6)	0 (0.0)	(0.0, 5.4)	−	−
因果関係の否定できない肝機能検査値異常 T-Bil	0 (0.0)	(0.0, 5.6)	1 (1.5)	(0.0, 8.0)	0.33*	1.00
因果関係の否定できない肝機能検査値異常 T-Bil Category1	0 (0.0)	(0.0, 5.6)	1 (1.5)	(0.0, 8.0)	0.33*	1.00
因果関係の否定できない肝機能検査値異常 T-Bil Category2	0 (0.0)	(0.0, 5.6)	0 (0.0)	(0.0, 5.4)	−	−
因果関係の否定できない肝機能検査値異常 T-Bil Category3	0 (0.0)	(0.0, 5.6)	0 (0.0)	(0.0, 5.4)	−	−

χ 二乗検定の P 値において、アスタリスクがついているものは、χ 二乗検定の実施要件を満たしていないため、結果に信頼性がないことを示します。

7. 部分集団解析

7.1. FAS

7.1.1. 部分集団 1

eGFR について、ベースライン値が 50 mL/min/1.73m^2 未満、かつ 24 週時変化率の絶対値が 15%以上の症例を除いた集団を部分集団 1 とする。

表 7.1.1.1. [FAS] 要約統計量と t 検定、Wilcoxon 検定

変数		観察ポイント	統計量	トピロキソスタット群	アロプリノール群	群間比較 P 値
対数変換 NT-proBNP (ln(pg/mL))	測定値	24 週	n	57	56	
			Mean±SD	6.5±0.9	6.2±1.0	0.11
			Median [Q1, Q3]	6.3 [5.9, 7.1]	6.1 [5.5, 7.0]	0.10
			Min, Max	4.7, 8.9	4.2, 8.8	
	変化率	24 週	n	57	56	
			Mean±SD	0.9±7.9	-0.5±8.3	0.36
			Median [Q1, Q3]	0.2 [-3.3, 4.2]	-0.4 [-4.4, 4.2]	0.68
			Min, Max	-15.5, 30.5	-27.6, 16.9	
			One-sample t-test	0.41	0.65	
			Wilcoxon signed-rank test	0.66	0.92	

表 7.1.1.2. [FAS] 共分散分析

変数		観察ポイント	n	調整済み平均値(SE)		調整済み平均値の差(95%CI)	群間比較 P 値
				トピロキソスタット群	アロプリノール群		
対数変換 NT-proBNP (ln(pg/mL))	変化率	24 週	113	0.0 (1.2)	-1.4 (1.2)	1.4 (-1.6, 4.4)	0.37

群を固定効果、割付調整因子(BNP 200 pg/ml 未満／200 pg/ml 以上、心臓超音波検査 EF 45%未満／45%以上)を共変量とした共分散分析を実施。

表 7.1.1.3. [FAS] MMRM

変数		観察ポイント	n	調整済み平均値(SE)		調整済み平均値の差(95%CI)	群間比較 P 値
				トピロキソスタット群	アロプリノール群		
対数変換 NT-proBNP (ln(pg/mL))	変化率	24 週	118	0.4 (1.2)	-0.7 (1.2)	1.1 (-1.9, 4.2)	0.47

群および時期効果、群と時期の交互作用、割付調整因子(BNP 200 pg/ml 未満／200 pg/ml 以上、心臓超音波検査 EF 45%未満／45%以上)を固定効果、研究対象者を変量効果としたモデルにて MMRM を実施。

7.1.2. 部分集団 2

eGFR について、ベースライン値が 50 mL/min/1.73m^2 以上 60 mL/min/1.73m^2 未満、かつ 24 週時変化率が－15%以下、かつ 24 週時測定値が 50 mL/min/1.73m^2 未満の症例を除いた集団を部分集団 2 とする。

表 7.1.2.1. [FAS] 要約統計量と t 検定、Wilcoxon 検定

変数		観察ポイント	統計量	トピロキソスタット群	アロプリノール群	群間比較 P 値
対数変換 NT-proBNP (ln(pg/mL))	測定値	24 週	n	64	64	
			Mean±SD	6.5±0.9	6.3±1.0	0.14
			Median [Q1, Q3]	6.4 [5.9, 7.2]	6.2 [5.5, 7.0]	0.12
			Min, Max	4.7, 8.9	4.2, 9.4	
	変化率	24 週	n	64	64	
			Mean±SD	1.2±7.4	-0.3±8.0	0.27
			Median [Q1, Q3]	0.4 [-2.9, 4.4]	-0.4 [-4.2, 5.0]	0.48
			Min, Max	-15.5, 28.4	-27.6, 16.9	
			One-sample t-test	0.19	0.77	
			Wilcoxon signed-rank test	0.29	0.82	

表 7.1.2.2. [FAS] 共分散分析

変数		観察ポイント	n	調整済み平均値(SE)		調整済み平均値の差(95%CI)	群間比較 P 値
				トピロキソスタット群	アロプリノール群		
対数変換 NT-proBNP (ln(pg/mL))	変化率	24 週	128	0.4 (1.1)	-1.0 (1.1)	1.4 (-1.4, 4.1)	0.32

群を固定効果、割付調整因子(BNP 200 pg/ml 未満／200 pg/ml 以上、心臓超音波検査 EF 45%未満／45%以上)を共変量とした共分散分析を実施。

表 7.1.2.3. [FAS] MMRM

変数		観察ポイント	n	調整済み平均値(SE)		調整済み平均値の差(95%CI)	群間比較 P 値
				トピロキソスタット群	アロプリノール群		
対数変換 NT-proBNP (ln(pg/mL))	変化率	24 週	132	0.6 (1.1)	-0.5 (1.0)	1.1 (-1.7, 3.8)	0.45

群および時期効果、群と時期の交互作用、割付調整因子(BNP 200 pg/ml 未満／200 pg/ml 以上、心臓超音波検査 EF 45%未満／45%以上)を固定効果、研究対象者を変量効果としたモデルにて MMRM を実施。

7.1.3. 部分集団 3

eGFR について、ベースライン値が 60 mL/min/1.73m^2 以上、かつ 24 週時測定値が 50 mL/min/1.73m^2 未満の症例を除いた集団を部分集団 3 とする。

表 7.1.3.1. [FAS] 要約統計量と t 検定、Wilcoxon 検定

変数		観察ポイント	統計量	トピロキソスタット群	アロプリノール群	群間比較 P 値
対数変換 NT-proBNP (ln(pg/mL))	測定値	24 週	n	65	66	
			Mean±SD	6.5±0.9	6.3±1.0	0.23
			Median [Q1, Q3]	6.4 [5.9, 7.1]	6.2 [5.5, 7.0]	0.18
			Min, Max	4.7, 8.4	4.2, 9.4	
	変化率	24 週	n	65	66	
			Mean±SD	1.4±8.2	-0.4±8.0	0.21
			Median [Q1, Q3]	0.3 [-3.1, 4.2]	-0.4 [-4.3, 5.0]	0.51
			Min, Max	-15.5, 30.5	-27.6, 16.9	
			One-sample t-test	0.18	0.69	
			Wilcoxon signed-rank test	0.37	0.92	

表 7.1.3.2. [FAS] 共分散分析

変数		観察ポイント	n	調整済み平均値(SE)		調整済み平均値の差(95%CI)	群間比較 P 値
				トピロキソスタット群	アロプリノール群		
対数変換 NT-proBNP (ln(pg/mL))	変化率	24 週	131	0.4 (1.1)	-1.2 (1.1)	1.6 (-1.2, 4.4)	0.25

群を固定効果、割付調整因子(BNP 200 pg/ml 未満/200 pg/ml 以上、心臓超音波検査 EF 45%未満/45%以上)を共変量とした共分散分析を実施。

表 7.1.3.3. [FAS] MMRM

変数		観察ポイント	n	調整済み平均値(SE)		調整済み平均値の差(95%CI)	群間比較 P 値
				トピロキソスタット群	アロプリノール群		
対数変換 NT-proBNP (ln(pg/mL))	変化率	24 週	136	1.0 (1.1)	-0.5 (1.1)	1.5 (-1.3, 4.3)	0.30

群および時期効果、群と時期の交互作用、割付調整因子(BNP 200 pg/ml 未満/200 pg/ml 以上、心臓超音波検査 EF 45%未満/45%以上)を固定効果、研究対象者を変量効果としたモデルにて MMRM を実施。

7.2. PPS

7.2.1. 部分集団 1

eGFR について、ベースライン値が 50 mL/min/1.73m^2 未満、かつ 24 週時変化率の絶対値が 15%以上の症例を除いた集団を部分集団 1 とする。

表 7.2.1.1. [PPS] 要約統計量と t 検定、Wilcoxon 検定

変数		観察ポイント	統計量	トピロキソスタット群	アロプリノール群	群間比較 P 値
対数変換 NT-proBNP (ln(pg/mL))	測定値	24 週	n	52	54	
			Mean±SD	6.4±0.8	6.2±1.0	0.32
			Median [Q1, Q3]	6.2 [5.9, 7.0]	6.1 [5.5, 7.0]	0.23
			Min, Max	4.7, 8.4	4.6, 8.8	
	変化率	24 週	n	52	54	
			Mean±SD	0.5±7.7	−0.1±7.8	0.69
			Median [Q1, Q3]	0.0 [−3.2, 3.9]	0.4 [−4.3, 5.0]	0.97
			Min, Max	−15.5, 30.5	−27.6, 16.9	
			One-sample t-test	0.62	0.95	
			Wilcoxon signed-rank test	0.84	0.88	

表 7.2.1.2. [PPS] 共分散分析

変数		観察ポイント	n	調整済み平均値(SE)		調整済み平均値の差(95%CI)	群間比較 P 値
				トピロキソスタット群	アロプリノール群		
対数変換 NT-proBNP (ln(pg/mL))	変化率	24 週	106	−0.5 (1.2)	-1.1 (1.2)	0.6 (−2.4, 3.6)	0.69

群を固定効果、割付調整因子(BNP 200 pg/ml 未満／200 pg/ml 以上、心臓超音波検査 EF 45%未満／45%以上)を共変量とした共分散分析を実施。

表 7.2.1.3. [PPS] MMRM

変数		観察ポイント	n	調整済み平均値(SE)		調整済み平均値の差(95%CI)	群間比較 P 値
				トピロキソスタット群	アロプリノール群		
対数変換 NT-proBNP (ln(pg/mL))	変化率	24 週	111	0.1 (1.2)	−0.5 (1.2)	0.6 (−2.5, 3.6)	0.70

群および時期効果、群と時期の交互作用、割付調整因子(BNP 200 pg/ml 未満／200 pg/ml 以上、心臓超音波検査 EF 45%未満／45%以上)を固定効果、研究対象者を変量効果としたモデルにて MMRM を実施。

7.2.2. 部分集団 2

eGFR について、ベースライン値が 50 mL/min/1.73m^2 以上 60 mL/min/1.73m^2 未満、かつ 24 週時変化率が－15%以下、かつ 24 週時測定値が 50 mL/min/1.73m^2 未満の症例を除いた集団を部分集団 2 とする。

表 7.2.2.1. [PPS] 要約統計量と t 検定、Wilcoxon 検定

変数		観察ポイント	統計量	トピロキソスタット群	アロプリノール群	群間比較 P 値
対数変換 NT-proBNP (ln(pg/mL))	測定値	24 週	n	59	61	
			Mean±SD	6.5±0.9	6.3±1.0	0.30
			Median [Q1, Q3]	6.4 [5.9, 7.2]	6.2 [5.5, 7.0]	0.21
			Min, Max	4.7, 8.4	4.6, 9.4	
	変化率	24 週	n	59	61	
			Mean±SD	1.0±7.2	0.2±7.6	0.58
			Median [Q1, Q3]	0.3 [-2.8, 4.1]	0.5 [-3.9, 5.0]	0.78
			Min, Max	-15.5, 28.4	-27.6, 16.9	
			One-sample t-test	0.31	0.83	
			Wilcoxon signed-rank test	0.38	0.52	

表 7.2.2.2. [PPS] 共分散分析

変数		観察ポイント	n	調整済み平均値(SE)		調整済み平均値の差(95%CI)	群間比較 P 値
				トピロキソスタット群	アロプリノール群		
対数変換 NT-proBNP (ln(pg/mL))	変化率	24 週	120	0.1 (1.1)	-0.5 (1.1)	0.6 (-2.0, 3.3)	0.63

群を固定効果、割付調整因子(BNP 200 pg/ml 未満／200 pg/ml 以上、心臓超音波検査 EF 45%未満／45%以上)を共変量とした共分散分析を実施。

表 7.2.2.3. [PPS] MMRM

変数		観察ポイント	n	調整済み平均値(SE)		調整済み平均値の差(95%CI)	群間比較 P 値
				トピロキソスタット群	アロプリノール群		
対数変換 NT-proBNP (ln(pg/mL))	変化率	24 週	124	0.3 (1.1)	-0.2 (1.1)	0.5 (-2.2, 3.3)	0.70

群および時期効果、群と時期の交互作用、割付調整因子(BNP 200 pg/ml 未満／200 pg/ml 以上、心臓超音波検査 EF 45%未満／45%以上)を固定効果、研究対象者を変量効果としたモデルにて MMRM を実施。

7.2.3. 部分集団 3

eGFR について、ベースライン値が 60 mL/min/1.73m^{2}以上、かつ 24 週時測定値が 50 mL/min/1.73m^{2}未満の症例を除いた集団を部分集団 3 とする。

表 7.2.3.1. [PPS] 要約統計量と t 検定、Wilcoxon 検定

変数		観察ポイント	統計量	トピロキソスタット群	アロプリノール群	群間比較 P 値
対数変換 NT-proBNP (ln(pg/mL))	測定値	24 週	n	61	63	
			Mean±SD	6.5±0.9	6.3±1.0	0.33
			Median [Q1, Q3]	6.4 [5.9, 7.1]	6.2 [5.5, 7.0]	0.24
			Min, Max	4.7, 8.4	4.6, 9.4	
	変化率	24 週	n	61	63	
			Mean±SD	1.3±8.1	0.1±7.6	0.39
			Median [Q1, Q3]	0.3 [-2.8, 4.1]	0.5 [-4.0, 5.0]	0.71
			Min, Max	-15.5, 30.5	-27.6, 16.9	
			One-sample t-test	0.21	0.93	
			Wilcoxon signed-rank test	0.37	0.62	

表 7.2.3.2. [PPS] 共分散分析

変数		観察ポイント	n	調整済み平均値(SE)		調整済み平均値の差(95%CI)	群間比較 P 値
				トピロキソスタット群	アロプリノール群		
対数変換 NT-proBNP (ln(pg/mL))	変化率	24 週	124	0.4 (1.2)	-0.8 (1.1)	1.1 (-1.6, 3.9)	0.42

群を固定効果、割付調整因子(BNP 200 pg/ml 未満／200 pg/ml 以上、心臓超音波検査 EF 45%未満／45%以上)を共変量とした共分散分析を実施。

表 7.2.3.3. [PPS] MMRM

変数		観察ポイント	n	調整済み平均値(SE)		調整済み平均値の差(95%CI)	群間比較 P 値
				トピロキソスタット群	アロプリノール群		
対数変換 NT-proBNP (ln(pg/mL))	変化率	24 週	129	0.9 (1.1)	-0.2 (1.1)	1.2 (-1.7, 4.0)	0.42

群および時期効果、群と時期の交互作用、割付調整因子(BNP 200 pg/ml 未満／200 pg/ml 以上、心臓超音波検査 EF 45%未満／45%以上)を固定効果、研究対象者を変量効果としたモデルにて MMRM を実施。

8. 薬剤情報

表 8.1. [FAS] 研究対象薬の服薬の有無

変数	水準	トピロキソスタット群		アロプリノール群	
		n	症例数(%)	n	症例数(%)
トピロキソスタット	0 週	70	70 (100.0)	70	0 (0.0)
	12 週	70	69 (98.6)	70	0 (0.0)
	24 週	68	68 (100.0)	67	0 (0.0)
アロプリノール	0 週	70	0 (0.0)	70	70 (100.0)
	12 週	70	0 (0.0)	70	67 (95.7)
	24 週	68	0 (0.0)	67	66 (98.5)

表 8.2. [FAS] 研究対象薬トピロキソスタットの服薬用量

変数	観察ポイント	水準	トピロキソスタット群		アロプリノール群	
			n	症例数(%)	n	症例数(%)
トピロキソスタット 服薬量(mg/日)	0 週	0 mg	70	0 (0.0)	70	70 (100.0)
		20 mg		1 (1.4)		0 (0.0)
		30 mg		0 (0.0)		0 (0.0)
		40 mg		55 (78.6)		0 (0.0)
		60 mg		0 (0.0)		0 (0.0)
		80 mg		14 (20.0)		0 (0.0)
		120 mg		0 (0.0)		0 (0.0)
		160 mg		0 (0.0)		0 (0.0)
	12 週	0 mg	70	1 (1.4)	70	70 (100.0)
		20 mg		1 (1.4)		0 (0.0)
		30 mg		1 (1.4)		0 (0.0)
		40 mg		34 (48.6)		0 (0.0)
		60 mg		1 (1.4)		0 (0.0)
		80 mg		26 (37.1)		0 (0.0)
		120 mg		6 (8.6)		0 (0.0)
		160 mg		0 (0.0)		0 (0.0)
	24 週	0 mg	68	0 (0.0)	67	67 (100.0)
		20 mg		1 (1.5)		0 (0.0)
		30 mg		1 (1.5)		0 (0.0)
		40 mg		31 (45.6)		0 (0.0)
		60 mg		0 (0.0)		0 (0.0)
		80 mg		27 (39.7)		0 (0.0)
		120 mg		6 (8.8)		0 (0.0)
		160 mg		2 (2.9)		0 (0.0)

表 8.3. [FAS] 研究対象薬アロプリノールの服薬用量

変数	観察ポイント	水準	トピロキソスタット群		アロプリノール群	
			n	症例数(%)	n	症例数(%)
アロプリノール 服薬量(mg/日)	0 週	0 mg	70	70 (100.0)	70	0 (0.0)
		100 mg		0 (0.0)		69 (98.6)
		200 mg		0 (0.0)		1 (1.4)
	12 週	0 mg	70	70 (100.0)	70	3 (4.3)
		100 mg		0 (0.0)		48 (68.6)
		200 mg		0 (0.0)		19 (27.1)
	24 週	0 mg	68	68 (100.0)	67	1 (1.5)
		100 mg		0 (0.0)		46 (68.7)
		200 mg		0 (0.0)		20 (29.9)

表 8.4. [FAS] 併用薬 ベースライン時

変数	症例数(%)		群間比較 P 値	
	トピロキソスタット群<br>n = 70	アロプリノール群<br>n = 70	χ 二乗<br>検定	Fisher<br>検定
併用薬(研究対象薬以外の薬剤)	70 (100.0)	69 (98.6)	0.32*	1.00
SU 薬	3 (4.3)	2 (2.9)	0.65*	1.00
ビグアナイド薬	6 (8.6)	5 (7.1)	0.75	1.00
αグルコシダーゼ阻害薬	6 (8.6)	10 (14.3)	0.29	0.43
速効型インスリン分泌促進薬	1 (1.4)	1 (1.4)	1.00*	1.00
チアゾリジン薬	1 (1.4)	1 (1.4)	1.00*	1.00
選択的 DPP-4 阻害剤	13 (18.6)	11 (15.7)	0.65	0.82
選択的 SGLT2 阻害剤	0 (0.0)	0 (0.0)	-	-
GLP-1 受容体作動薬	1 (1.4)	0 (0.0)	0.32*	1.00
インスリン	2 (2.9)	4 (5.7)	0.40*	0.68
利尿薬	48 (68.6)	48 (68.6)	1.00	1.00
カルシウム拮抗薬	31 (44.3)	31 (44.3)	1.00	1.00
α遮断薬	0 (0.0)	1 (1.4)	0.32*	1.00
β遮断薬	33 (47.1)	35 (50.0)	0.74	0.87
α1β 遮断薬	16 (22.9)	17 (24.3)	0.84	1.00
ACEI	14 (20.0)	20 (28.6)	0.24	0.32
ARB	32 (45.7)	38 (54.3)	0.31	0.40
直接的レニン阻害薬	1 (1.4)	2 (2.9)	0.56*	1.00
V2-受容体拮抗剤	2 (2.9)	3 (4.3)	0.65*	1.00
血管・腎作動性高血圧治療剤	1 (1.4)	0 (0.0)	0.32*	1.00
スタチン	39 (55.7)	40 (57.1)	0.86	1.00
エゼチミブ	2 (2.9)	4 (5.7)	0.40*	0.68
EPA	2 (2.9)	3 (4.3)	0.65*	1.00
抗凝固剤	17 (24.3)	20 (28.6)	0.57	0.70
抗血小板剤	28 (40.0)	35 (50.0)	0.23	0.31
経口 FXa 阻害剤	10 (14.3)	11 (15.7)	0.81	1.00
選択的直接作用型第 Xa 因子阻害剤	18 (25.7)	11 (15.7)	0.14	0.21
5-HT2 ブロッカー	0 (0.0)	1 (1.4)	0.32*	1.00
Ca++拮抗性不整脈・虚血性心疾患治療剤	2 (2.9)	1 (1.4)	0.56*	1.00
H1 ブロッカー点眼剤	0 (0.0)	1 (1.4)	0.32*	1.00
H2 受容体拮抗剤	3 (4.3)	4 (5.7)	0.70*	1.00
アルツハイマー型、レビー小体型認知症治療剤	0 (0.0)	1 (1.4)	0.32*	1.00
アルツハイマー型認知症治療剤	0 (0.0)	1 (1.4)	0.32*	1.00
アルドース還元酵素阻害剤	0 (0.0)	1 (1.4)	0.32*	1.00
アレルギー性疾患治療剤	2 (2.9)	0 (0.0)	0.15*	0.50
ジギタリス配糖体製剤	2 (2.9)	2 (2.9)	1.00*	1.00
セロトニン・ノルアドレナリン再取り込み阻害剤	1 (1.4)	0 (0.0)	0.32*	1.00
チアプリド製剤	0 (0.0)	1 (1.4)	0.32*	1.00
ドパミン作動性パーキンソン病治療剤	1 (1.4)	0 (0.0)	0.32*	1.00
ドライパウダー吸入式喘息・COPD 治療配合剤	1 (1.4)	0 (0.0)	0.32*	1.00
プロトンポンプ・インヒビター	33 (47.1)	41 (58.6)	0.18	0.24
ホスホジエステラーゼ阻害剤	1 (1.4)	0 (0.0)	0.32*	1.00
マイナートランキライザー	1 (1.4)	0 (0.0)	0.32*	1.00

ロイコトリエン受容体拮抗薬	0 (0.0)	1 (1.4)	0.32*	1.00
亜鉛含有胃潰瘍治療剤	1 (1.4)	0 (0.0)	0.32*	1.00
胃炎・胃潰瘍治療剤	4 (5.7)	6 (8.6)	0.51	0.74
胃炎・潰瘍治療剤	3 (4.3)	0 (0.0)	0.08*	0.24
下行性疼痛抑制系賦活型疼痛治療剤	0 (0.0)	1 (1.4)	0.32*	1.00
可溶性の非イオン型鉄剤	0 (0.0)	2 (2.9)	0.15*	0.50
解熱鎮痛消炎剤	3 (4.3)	2 (2.9)	0.65*	1.00
活性生菌製剤	1 (1.4)	0 (0.0)	0.32*	1.00
緩下剤	1 (1.4)	1 (1.4)	1.00*	1.00
肝・胆・消化機能改善剤	4 (5.7)	2 (2.9)	0.40*	0.68
肝臓疾患用剤・アレルギー用薬	0 (0.0)	1 (1.4)	0.32*	1.00
含嗽剤	1 (1.4)	0 (0.0)	0.32*	1.00
気道潤滑去痰剤	1 (1.4)	0 (0.0)	0.32*	1.00
気道粘液調整・粘膜正常化剤	2 (2.9)	0 (0.0)	0.15*	0.50
逆流性食道炎・消化性潰瘍用剤	1 (1.4)	0 (0.0)	0.32*	1.00
強心配糖体製剤	1 (1.4)	0 (0.0)	0.32*	1.00
狭心症治療剤	14 (20.0)	11 (15.7)	0.51	0.66
狭心症治療用 ISMN 製剤	1 (1.4)	0 (0.0)	0.32*	1.00
筋緊張改善剤	0 (0.0)	1 (1.4)	0.32*	1.00
経口プロスタグランジン E1 誘導体製剤	0 (0.0)	1 (1.4)	0.32*	1.00
経皮吸収型鎮痛・抗炎症剤	1 (1.4)	1 (1.4)	1.00*	1.00
経皮吸収型鎮痛消炎剤	0 (0.0)	2 (2.9)	0.15*	0.50
経皮鎮痛消炎剤	3 (4.3)	0 (0.0)	0.08*	0.24
血行促進・皮膚保湿剤	0 (0.0)	1 (1.4)	0.32*	1.00
血清カリウム抑制剤	0 (0.0)	1 (1.4)	0.32*	1.00
抗てんかん剤	5 (7.1)	0 (0.0)	0.023*	0.06
抗甲状腺剤	1 (1.4)	0 (0.0)	0.32*	1.00
抗精神病薬	1 (1.4)	0 (0.0)	0.32*	1.00
抗不安剤	1 (1.4)	1 (1.4)	1.00*	1.00
甲状腺疾患治療薬	3 (4.3)	2 (2.9)	0.65*	1.00
高カリウム血症改善剤	2 (2.9)	2 (2.9)	1.00*	1.00
高コレステロール血症改善剤	2 (2.9)	0 (0.0)	0.15*	0.50
合成副腎皮質ホルモン剤	1 (1.4)	0 (0.0)	0.32*	1.00
持続性選択 H1 受容体拮抗・アレルギー性疾患治療剤	1 (1.4)	1 (1.4)	1.00*	1.00
徐放型鉄剤	0 (0.0)	2 (2.9)	0.15*	0.50
徐放性カリウム剤	0 (0.0)	1 (1.4)	0.32*	1.00
徐放性不整脈治療剤	1 (1.4)	0 (0.0)	0.32*	1.00
消化管運動機能改善剤	2 (2.9)	2 (2.9)	1.00*	1.00
消化管内ガス駆除剤	1 (1.4)	0 (0.0)	0.32*	1.00
消化酵素製剤	1 (1.4)	0 (0.0)	0.32*	1.00
消化性潰瘍治療剤	3 (4.3)	0 (0.0)	0.08*	0.24
植物性緩下剤	3 (4.3)	3 (4.3)	1.00*	1.00
心選択性 β ブロッカー	1 (1.4)	0 (0.0)	0.32*	1.00
心不全治療薬	4 (5.7)	0 (0.0)	0.042*	0.12
睡眠導入剤	3 (4.3)	1 (1.4)	0.31*	0.62
睡眠誘導剤	2 (2.9)	0 (0.0)	0.15*	0.50

制酸・緩下剤	15 (21.4)	7 (10.0)	0.06	0.10
整腸剤	0 (0.0)	1 (1.4)	0.32*	1.00
生菌製剤	0 (0.0)	1 (1.4)	0.32*	1.00
精神安定剤	3 (4.3)	0 (0.0)	0.08*	0.24
選択的α1A 遮断薬、前立腺肥大症に伴う排尿障害改善薬	1 (1.4)	0 (0.0)	0.32*	1.00
前立腺肥大症に伴う排尿障害改善剤	1 (1.4)	0 (0.0)	0.32*	1.00
前立腺肥大症の排尿障害改善剤	1 (1.4)	2 (2.9)	0.56*	1.00
総合感冒剤	1 (1.4)	0 (0.0)	0.32*	1.00
対血管薬剤	0 (0.0)	1 (1.4)	0.32*	1.00
胆汁・腎排泄型 ACE 阻害剤	1 (1.4)	0 (0.0)	0.32*	1.00
長時間作用性吸入気管支拡張剤	2 (2.9)	1 (1.4)	0.56*	1.00
直接トロンビン阻害剤	1 (1.4)	0 (0.0)	0.32*	1.00
鎮咳剤	1 (1.4)	0 (0.0)	0.32*	1.00
入眠剤	2 (2.9)	0 (0.0)	0.15*	0.50
粘膜防御性胃炎・胃潰瘍治療剤	0 (0.0)	1 (1.4)	0.32*	1.00
脳循環・代謝改善剤	0 (0.0)	1 (1.4)	0.32*	1.00
排尿障害改善剤・降圧剤	0 (0.0)	1 (1.4)	0.32*	1.00
皮膚外用合成副腎皮質ホルモン剤	1 (1.4)	0 (0.0)	0.32*	1.00
頻脈性不整脈治療剤	1 (1.4)	1 (1.4)	1.00*	1.00
不整脈治療剤	6 (8.6)	6 (8.6)	1.00	1.00
末梢性神経障害治療剤	1 (1.4)	1 (1.4)	1.00*	1.00
慢性腎不全用剤	4 (5.7)	1 (1.4)	0.17*	0.37
葉酸錠	0 (0.0)	1 (1.4)	0.32*	1.00
喘息・COPD 治療配合剤	0 (0.0)	1 (1.4)	0.32*	1.00
疼痛治療剤	1 (1.4)	1 (1.4)	1.00*	1.00
解熱鎮痛剤	0 (0.0)	0 (0.0)	–	–
不眠症治療薬	0 (0.0)	0 (0.0)	–	–
骨粗鬆症治療剤	0 (0.0)	0 (0.0)	–	–
皮膚外用合成副腎皮質ホルモン・抗生物質配合剤	0 (0.0)	0 (0.0)	–	–
漢方製剤	0 (0.0)	0 (0.0)	–	–
胃炎・消化性潰瘍治療剤	0 (0.0)	0 (0.0)	–	–
セフェム系抗生物質	0 (0.0)	0 (0.0)	–	–
抗精神病剤	0 (0.0)	0 (0.0)	–	–

χ二乗検定のＰ値において、アスタリスクがついているものは、χ二乗検定の実施要件を満たしていないため、結果に信頼性がないことを示します。

表 8.5. [FAS] 併用薬 12 週時

変数	症例数(%)		群間比較 P 値	
	トピロキソスタット群 n = 70	アロプリノール群 n = 70	χ 二乗検定	Fisher 検定
併用薬(研究対象薬以外の薬剤)	69 (98.6)	69 (98.6)	1.00*	1.00
SU 薬	3 (4.3)	1 (1.4)	0.31*	0.62
ビグアナイド薬	6 (8.6)	5 (7.1)	0.75	1.00
αグルコシダーゼ阻害薬	6 (8.6)	10 (14.3)	0.29	0.43
速効型インスリン分泌促進薬	1 (1.4)	1 (1.4)	1.00*	1.00
チアゾリジン薬	1 (1.4)	1 (1.4)	1.00*	1.00
選択的 DPP-4 阻害剤	13 (18.6)	11 (15.7)	0.65	0.82
選択的 SGLT2 阻害剤	1 (1.4)	0 (0.0)	0.32*	1.00
GLP-1 受容体作動薬	1 (1.4)	0 (0.0)	0.32*	1.00
インスリン	2 (2.9)	5 (7.1)	0.24*	0.44
利尿薬	47 (67.1)	48 (68.6)	0.86	1.00
カルシウム拮抗薬	29 (41.4)	31 (44.3)	0.73	0.86
α遮断薬	0 (0.0)	1 (1.4)	0.32*	1.00
β遮断薬	32 (45.7)	35 (50.0)	0.61	0.74
α1β 遮断薬	17 (24.3)	17 (24.3)	1.00	1.00
ACEI	12 (17.1)	20 (28.6)	0.11	0.16
ARB	33 (47.1)	37 (52.9)	0.50	0.61
直接的レニン阻害薬	1 (1.4)	2 (2.9)	0.56*	1.00
V2-受容体拮抗剤	2 (2.9)	2 (2.9)	1.00*	1.00
血管・腎作動性高血圧治療剤	1 (1.4)	0 (0.0)	0.32*	1.00
スタチン	38 (54.3)	39 (55.7)	0.87	1.00
エゼチミブ	2 (2.9)	3 (4.3)	0.65*	1.00
EPA	2 (2.9)	3 (4.3)	0.65*	1.00
抗凝固剤	16 (22.9)	20 (28.6)	0.44	0.56
抗血小板剤	27 (38.6)	34 (48.6)	0.23	0.31
経口 FXa 阻害剤	11 (15.7)	11 (15.7)	1.00	1.00
選択的直接作用型第 Xa 因子阻害剤	18 (25.7)	11 (15.7)	0.14	0.21
5-HT2 ブロッカー	0 (0.0)	1 (1.4)	0.32*	1.00
Ca++拮抗性不整脈・虚血性心疾患治療剤	2 (2.9)	1 (1.4)	0.56*	1.00
H1 ブロッカー点眼剤	0 (0.0)	1 (1.4)	0.32*	1.00
H2 受容体拮抗剤	4 (5.7)	4 (5.7)	1.00*	1.00
アルツハイマー型、レビー小体型認知症治療剤	0 (0.0)	1 (1.4)	0.32*	1.00
アルツハイマー型認知症治療剤	0 (0.0)	1 (1.4)	0.32*	1.00
アルドース還元酵素阻害剤	0 (0.0)	1 (1.4)	0.32*	1.00
アレルギー性疾患治療剤	2 (2.9)	0 (0.0)	0.15*	0.50
ジギタリス配糖体製剤	2 (2.9)	2 (2.9)	1.00*	1.00
セロトニン・ノルアドレナリン再取り込み阻害剤	1 (1.4)	0 (0.0)	0.32*	1.00
チアプリド製剤	0 (0.0)	1 (1.4)	0.32*	1.00
ドパミン作動性パーキンソン病治療剤	1 (1.4)	0 (0.0)	0.32*	1.00
ドライパウダー吸入式喘息・COPD 治療配合剤	1 (1.4)	0 (0.0)	0.32*	1.00
プロトンポンプ・インヒビター	33 (47.1)	40 (57.1)	0.24	0.31
ホスホジエステラーゼ阻害剤	1 (1.4)	0 (0.0)	0.32*	1.00
マイナートランキライザー	1 (1.4)	1 (1.4)	1.00*	1.00

ロイコトリエン受容体拮抗薬	0 (0.0)	1 (1.4)	0.32*	1.00
亜鉛含有胃潰瘍治療剤	1 (1.4)	0 (0.0)	0.32*	1.00
胃炎・胃潰瘍治療剤	4 (5.7)	6 (8.6)	0.51	0.74
胃炎・潰瘍治療剤	3 (4.3)	0 (0.0)	0.08*	0.24
下行性疼痛抑制系賦活型疼痛治療剤	0 (0.0)	1 (1.4)	0.32*	1.00
可溶性の非イオン型鉄剤	0 (0.0)	2 (2.9)	0.15*	0.50
解熱鎮痛消炎剤	3 (4.3)	2 (2.9)	0.65*	1.00
活性生菌製剤	1 (1.4)	0 (0.0)	0.32*	1.00
緩下剤	1 (1.4)	1 (1.4)	1.00*	1.00
肝・胆・消化機能改善剤	3 (4.3)	2 (2.9)	0.65*	1.00
肝臓疾患用剤・アレルギー用薬	0 (0.0)	1 (1.4)	0.32*	1.00
含嗽剤	1 (1.4)	0 (0.0)	0.32*	1.00
気道潤滑去痰剤	1 (1.4)	0 (0.0)	0.32*	1.00
気道粘液調整・粘膜正常化剤	2 (2.9)	0 (0.0)	0.15*	0.50
逆流性食道炎・消化性潰瘍用剤	1 (1.4)	0 (0.0)	0.32*	1.00
強心配糖体製剤	1 (1.4)	0 (0.0)	0.32*	1.00
狭心症治療剤	14 (20.0)	11 (15.7)	0.51	0.66
狭心症治療用 ISMN 製剤	1 (1.4)	0 (0.0)	0.32*	1.00
筋緊張改善剤	0 (0.0)	1 (1.4)	0.32*	1.00
経口プロスタグランジン E1 誘導体製剤	0 (0.0)	1 (1.4)	0.32*	1.00
経皮吸収型鎮痛・抗炎症剤	1 (1.4)	1 (1.4)	1.00*	1.00
経皮吸収型鎮痛消炎剤	0 (0.0)	2 (2.9)	0.15*	0.50
経皮鎮痛消炎剤	3 (4.3)	0 (0.0)	0.08*	0.24
血行促進・皮膚保湿剤	0 (0.0)	1 (1.4)	0.32*	1.00
血清カリウム抑制剤	0 (0.0)	1 (1.4)	0.32*	1.00
抗てんかん剤	6 (8.6)	0 (0.0)	0.012*	0.028
抗甲状腺剤	1 (1.4)	0 (0.0)	0.32*	1.00
抗精神病薬	1 (1.4)	0 (0.0)	0.32*	1.00
抗不安剤	1 (1.4)	1 (1.4)	1.00*	1.00
甲状腺疾患治療薬	2 (2.9)	2 (2.9)	1.00*	1.00
高カリウム血症改善剤	3 (4.3)	1 (1.4)	0.31*	0.62
高コレステロール血症改善剤	2 (2.9)	0 (0.0)	0.15*	0.50
合成副腎皮質ホルモン剤	1 (1.4)	0 (0.0)	0.32*	1.00
持続性選択 H1 受容体拮抗・アレルギー性疾患治療剤	1 (1.4)	1 (1.4)	1.00*	1.00
徐放型鉄剤	0 (0.0)	2 (2.9)	0.15*	0.50
徐放性カリウム剤	0 (0.0)	1 (1.4)	0.32*	1.00
徐放性不整脈治療剤	1 (1.4)	0 (0.0)	0.32*	1.00
消化管運動機能改善剤	3 (4.3)	2 (2.9)	0.65*	1.00
消化管内ガス駆除剤	1 (1.4)	0 (0.0)	0.32*	1.00
消化酵素製剤	1 (1.4)	0 (0.0)	0.32*	1.00
消化性潰瘍治療剤	3 (4.3)	0 (0.0)	0.08*	0.24
植物性緩下剤	3 (4.3)	3 (4.3)	1.00*	1.00
心選択性 β ブロッカー	1 (1.4)	0 (0.0)	0.32*	1.00
心不全治療薬	4 (5.7)	0 (0.0)	0.042*	0.12
睡眠導入剤	3 (4.3)	1 (1.4)	0.31*	0.62
睡眠誘導剤	2 (2.9)	0 (0.0)	0.15*	0.50

制酸・緩下剤	16 (22.9)	7 (10.0)	0.040	0.07
整腸剤	0 (0.0)	1 (1.4)	0.32*	1.00
生菌製剤	0 (0.0)	1 (1.4)	0.32*	1.00
精神安定剤	3 (4.3)	0 (0.0)	0.08*	0.24
選択的α1A 遮断薬、前立腺肥大症に伴う排尿障害改善薬	1 (1.4)	0 (0.0)	0.32*	1.00
前立腺肥大症に伴う排尿障害改善剤	1 (1.4)	0 (0.0)	0.32*	1.00
前立腺肥大症の排尿障害改善剤	1 (1.4)	2 (2.9)	0.56*	1.00
総合感冒剤	1 (1.4)	0 (0.0)	0.32*	1.00
対血管薬剤	0 (0.0)	1 (1.4)	0.32*	1.00
胆汁・腎排泄型 ACE 阻害剤	1 (1.4)	0 (0.0)	0.32*	1.00
長時間作用性吸入気管支拡張剤	2 (2.9)	1 (1.4)	0.56*	1.00
直接トロンビン阻害剤	1 (1.4)	0 (0.0)	0.32*	1.00
鎮咳剤	1 (1.4)	0 (0.0)	0.32*	1.00
入眠剤	2 (2.9)	0 (0.0)	0.15*	0.50
粘膜防御性胃炎・胃潰瘍治療剤	0 (0.0)	1 (1.4)	0.32*	1.00
脳循環・代謝改善剤	0 (0.0)	1 (1.4)	0.32*	1.00
排尿障害改善剤・降圧剤	0 (0.0)	1 (1.4)	0.32*	1.00
皮膚外用合成副腎皮質ホルモン剤	1 (1.4)	0 (0.0)	0.32*	1.00
頻脈性不整脈治療剤	1 (1.4)	0 (0.0)	0.32*	1.00
不整脈治療剤	6 (8.6)	6 (8.6)	1.00	1.00
末梢性神経障害治療剤	1 (1.4)	1 (1.4)	1.00*	1.00
慢性腎不全用剤	4 (5.7)	1 (1.4)	0.17*	0.37
葉酸錠	0 (0.0)	1 (1.4)	0.32*	1.00
喘息・COPD 治療配合剤	0 (0.0)	1 (1.4)	0.32*	1.00
疼痛治療剤	1 (1.4)	1 (1.4)	1.00*	1.00
解熱鎮痛剤	1 (1.4)	0 (0.0)	0.32*	1.00
不眠症治療薬	1 (1.4)	0 (0.0)	0.32*	1.00
骨粗鬆症治療剤	1 (1.4)	0 (0.0)	0.32*	1.00
皮膚外用合成副腎皮質ホルモン・抗生物質配合剤	1 (1.4)	0 (0.0)	0.32*	1.00
漢方製剤	1 (1.4)	0 (0.0)	0.32*	1.00
胃炎・消化性潰瘍治療剤	0 (0.0)	1 (1.4)	0.32*	1.00
セフェム系抗生物質	0 (0.0)	1 (1.4)	0.32*	1.00
抗精神病剤	1 (1.4)	0 (0.0)	0.32*	1.00

χ二乗検定のＰ値において、アスタリスクがついているものは、χ二乗検定の実施要件を満たしていないため、結果に信頼性がないことを示します。

表 8.6. [FAS] 併用薬 24 週時

変数	症例数(%)		群間比較 P 値	
	トピロキソスタット群 n = 68	アロプリノール群 n = 67	χ 二乗検定	Fisher 検定
併用薬(研究対象薬以外の薬剤)	68 (100.0)	66 (98.5)	0.31*	0.50
SU 薬	3 (4.4)	1 (1.5)	0.32*	0.62
ビグアナイド薬	6 (8.8)	4 (6.0)	0.53*	0.74
αグルコシダーゼ阻害薬	6 (8.8)	8 (11.9)	0.55	0.59
速効型インスリン分泌促進薬	1 (1.5)	1 (1.5)	0.99*	1.00
チアゾリジン薬	1 (1.5)	1 (1.5)	0.99*	1.00
選択的 DPP-4 阻害剤	13 (19.1)	11 (16.4)	0.68	0.82
選択的 SGLT2 阻害剤	1 (1.5)	0 (0.0)	0.32*	1.00
GLP-1 受容体作動薬	1 (1.5)	0 (0.0)	0.32*	1.00
インスリン	2 (2.9)	4 (6.0)	0.39*	0.44
利尿薬	46 (67.6)	45 (67.2)	0.95	1.00
カルシウム拮抗薬	29 (42.6)	30 (44.8)	0.80	0.86
α遮断薬	0 (0.0)	1 (1.5)	0.31*	0.50
β遮断薬	31 (45.6)	35 (52.2)	0.44	0.49
α1β 遮断薬	17 (25.0)	16 (23.9)	0.88	1.00
ACEI	12 (17.6)	19 (28.4)	0.14	0.16
ARB	33 (48.5)	37 (55.2)	0.44	0.49
直接的レニン阻害薬	1 (1.5)	2 (3.0)	0.55*	0.62
V2-受容体拮抗剤	3 (4.4)	2 (3.0)	0.66*	1.00
血管・腎作動性高血圧治療剤	1 (1.5)	0 (0.0)	0.32*	1.00
スタチン	38 (55.9)	39 (58.2)	0.78	0.86
エゼチミブ	2 (2.9)	3 (4.5)	0.64*	0.68
EPA	2 (2.9)	3 (4.5)	0.64*	0.68
抗凝固剤	15 (22.1)	18 (26.9)	0.52	0.55
抗血小板剤	26 (38.2)	33 (49.3)	0.20	0.23
経口 FXa 阻害剤	11 (16.2)	11 (16.4)	0.97	1.00
選択的直接作用型第 Xa 因子阻害剤	17 (25.0)	10 (14.9)	0.14	0.20
5-HT2 ブロッカー	0 (0.0)	1 (1.5)	0.31*	0.50
Ca++拮抗性不整脈・虚血性心疾患治療剤	2 (2.9)	1 (1.5)	0.57*	1.00
H1 ブロッカー点眼剤	0 (0.0)	1 (1.5)	0.31*	0.50
H2 受容体拮抗剤	3 (4.4)	4 (6.0)	0.68*	0.72
アルツハイマー型、レビー小体型認知症治療剤	0 (0.0)	1 (1.5)	0.31*	0.50
アルツハイマー型認知症治療剤	0 (0.0)	1 (1.5)	0.31*	0.50
アルドース還元酵素阻害剤	0 (0.0)	0 (0.0)	–	–
アレルギー性疾患治療剤	2 (2.9)	0 (0.0)	0.16*	0.50
ジギタリス配糖体製剤	3 (4.4)	2 (3.0)	0.66*	1.00
セロトニン・ノルアドレナリン再取り込み阻害剤	1 (1.5)	0 (0.0)	0.32*	1.00
チアプリド製剤	0 (0.0)	1 (1.5)	0.31*	0.50
ドパミン作動性パーキンソン病治療剤	1 (1.5)	0 (0.0)	0.32*	1.00
ドライパウダー吸入式喘息・COPD 治療配合剤	1 (1.5)	0 (0.0)	0.32*	1.00
プロトンポンプ・インヒビター	33 (48.5)	39 (58.2)	0.26	0.30
ホスホジエステラーゼ阻害剤	1 (1.5)	0 (0.0)	0.32*	1.00
マイナートランキライザー	1 (1.5)	1 (1.5)	0.99*	1.00
ロイコトリエン受容体拮抗薬	0 (0.0)	1 (1.5)	0.31*	0.50

亜鉛含有胃潰瘍治療剤	1 (1.5)	0 (0.0)	0.32*	1.00
胃炎・胃潰瘍治療剤	4 (5.9)	6 (9.0)	0.50*	0.53
胃炎・潰瘍治療剤	3 (4.4)	0 (0.0)	0.08*	0.24
下行性疼痛抑制系賦活型疼痛治療剤	0 (0.0)	1 (1.5)	0.31*	0.50
可溶性の非イオン型鉄剤	0 (0.0)	2 (3.0)	0.15*	0.24
解熱鎮痛消炎剤	3 (4.4)	2 (3.0)	0.66*	1.00
活性生菌製剤	1 (1.5)	0 (0.0)	0.32*	1.00
緩下剤	1 (1.5)	1 (1.5)	0.99*	1.00
肝・胆・消化機能改善剤	3 (4.4)	2 (3.0)	0.66*	1.00
肝臓疾患用剤・アレルギー用薬	0 (0.0)	1 (1.5)	0.31*	0.50
含嗽剤	1 (1.5)	0 (0.0)	0.32*	1.00
気道潤滑去痰剤	1 (1.5)	0 (0.0)	0.32*	1.00
気道粘液調整・粘膜正常化剤	2 (2.9)	0 (0.0)	0.16*	0.50
逆流性食道炎・消化性潰瘍用剤	1 (1.5)	0 (0.0)	0.32*	1.00
強心配糖体製剤	1 (1.5)	0 (0.0)	0.32*	1.00
狭心症治療剤	14 (20.6)	10 (14.9)	0.39	0.50
狭心症治療用 ISMN 製剤	1 (1.5)	0 (0.0)	0.32*	1.00
筋緊張改善剤	0 (0.0)	1 (1.5)	0.31*	0.50
経口プロスタグランジン E1 誘導体製剤	0 (0.0)	1 (1.5)	0.31*	0.50
経皮吸収型鎮痛・抗炎症剤	1 (1.5)	1 (1.5)	0.99*	1.00
経皮吸収型鎮痛消炎剤	0 (0.0)	1 (1.5)	0.31*	0.50
経皮鎮痛消炎剤	3 (4.4)	0 (0.0)	0.08*	0.24
血行促進・皮膚保湿剤	0 (0.0)	0 (0.0)	–	–
血清カリウム抑制剤	0 (0.0)	1 (1.5)	0.31*	0.50
抗てんかん剤	6 (8.8)	0 (0.0)	0.013*	0.028
抗甲状腺剤	1 (1.5)	0 (0.0)	0.32*	1.00
抗精神病薬	1 (1.5)	0 (0.0)	0.32*	1.00
抗不安剤	1 (1.5)	1 (1.5)	0.99*	1.00
甲状腺疾患治療薬	2 (2.9)	2 (3.0)	0.99*	1.00
高カリウム血症改善剤	3 (4.4)	1 (1.5)	0.32*	0.62
高コレステロール血症改善剤	2 (2.9)	0 (0.0)	0.16*	0.50
合成副腎皮質ホルモン剤	1 (1.5)	0 (0.0)	0.32*	1.00
持続性選択 H1 受容体拮抗・アレルギー性疾患治療剤	1 (1.5)	1 (1.5)	0.99*	1.00
徐放型鉄剤	0 (0.0)	2 (3.0)	0.15*	0.24
徐放性カリウム剤	0 (0.0)	1 (1.5)	0.31*	0.50
徐放性不整脈治療剤	1 (1.5)	0 (0.0)	0.32*	1.00
消化管運動機能改善剤	2 (2.9)	2 (3.0)	0.99*	1.00
消化管内ガス駆除剤	1 (1.5)	0 (0.0)	0.32*	1.00
消化酵素製剤	1 (1.5)	0 (0.0)	0.32*	1.00
消化性潰瘍治療剤	3 (4.4)	0 (0.0)	0.08*	0.24
植物性緩下剤	3 (4.4)	3 (4.5)	0.99*	1.00
心選択性 β ブロッカー	1 (1.5)	0 (0.0)	0.32*	1.00
心不全治療薬	4 (5.9)	0 (0.0)	0.044*	0.12
睡眠導入剤	3 (4.4)	1 (1.5)	0.32*	0.62
睡眠誘導剤	2 (2.9)	0 (0.0)	0.16*	0.50
制酸・緩下剤	15 (22.1)	7 (10.4)	0.07	0.10
整腸剤	0 (0.0)	1 (1.5)	0.31*	0.50
生菌製剤	0 (0.0)	0 (0.0)	–	–

精神安定剤	3 (4.4)	0 (0.0)	0.08*	0.24
選択的α1A 遮断薬、前立腺肥大症に伴う排尿障害改善薬	1 (1.5)	0 (0.0)	0.32*	1.00
前立腺肥大症に伴う排尿障害改善剤	1 (1.5)	0 (0.0)	0.32*	1.00
前立腺肥大症の排尿障害改善剤	1 (1.5)	2 (3.0)	0.55*	0.62
総合感冒剤	1 (1.5)	0 (0.0)	0.32*	1.00
対血管薬剤	0 (0.0)	1 (1.5)	0.31*	0.50
胆汁・腎排泄型 ACE 阻害剤	1 (1.5)	0 (0.0)	0.32*	1.00
長時間作用性吸入気管支拡張剤	2 (2.9)	1 (1.5)	0.57*	1.00
直接トロンビン阻害剤	1 (1.5)	0 (0.0)	0.32*	1.00
鎮咳剤	1 (1.5)	0 (0.0)	0.32*	1.00
入眠剤	2 (2.9)	0 (0.0)	0.16*	0.50
粘膜防御性胃炎・胃潰瘍治療剤	0 (0.0)	2 (3.0)	0.15*	0.24
脳循環・代謝改善剤	0 (0.0)	1 (1.5)	0.31*	0.50
排尿障害改善剤・降圧剤	0 (0.0)	1 (1.5)	0.31*	0.50
皮膚外用合成副腎皮質ホルモン剤	1 (1.5)	0 (0.0)	0.32*	1.00
頻脈性不整脈治療剤	1 (1.5)	0 (0.0)	0.32*	1.00
不整脈治療剤	6 (8.8)	6 (9.0)	0.98	1.00
末梢性神経障害治療剤	1 (1.5)	1 (1.5)	0.99*	1.00
慢性腎不全用剤	4 (5.9)	1 (1.5)	0.18*	0.37
葉酸錠	0 (0.0)	1 (1.5)	0.31*	0.50
喘息・COPD 治療配合剤	0 (0.0)	1 (1.5)	0.31*	0.50
疼痛治療剤	1 (1.5)	1 (1.5)	0.99*	1.00
解熱鎮痛剤	1 (1.5)	0 (0.0)	0.32*	1.00
不眠症治療薬	1 (1.5)	0 (0.0)	0.32*	1.00
骨粗鬆症治療剤	1 (1.5)	0 (0.0)	0.32*	1.00
皮膚外用合成副腎皮質ホルモン・抗生物質配合剤	1 (1.5)	0 (0.0)	0.32*	1.00
漢方製剤	1 (1.5)	0 (0.0)	0.32*	1.00
胃炎・消化性潰瘍治療剤	0 (0.0)	1 (1.5)	0.31*	0.50
セフェム系抗生物質	0 (0.0)	0 (0.0)	–	–
抗精神病剤	1 (1.5)	0 (0.0)	0.32*	1.00

χ二乗検定のＰ値において、アスタリスクがついているものは、χ二乗検定の実施要件を満たしていないため、結果に信頼性がないことを示します。
