# Supplementary material for: Topiroxostat versus allopurinol in patients with chronic heart failure complicated by hyperuricemia: A prospective, randomized, open-label, blinded-end-point clinical trial
Source: PLoS One. 2022 Jan 25;17(1):e0261445. doi: 10.1371/journal.pone.0261445 (PMC8789120; doi:10.1371/journal.pone.0261445)
Supplement: S2 Appendix — (PDF) [file pone.0261445.s009.pdf]

高尿酸血症を合併した心不全患者における
トピロキソスタットとアロプリノールのランダム化比較試験

The **E**ffect of **X**anthine Oxidase Inhibitor
in **C**hron**I**c heart failure patients
complica**TED** with hyper-**U**ricemi**A**

Excited-UA study

追加解析報告書
Ver 1.0

研究代表者 ：
獨協医科大学病院 心臓･血管内科 教授　井上 晃男

統計解析責任者 ：
株式会社 総合医科学研究所 医薬臨床研究支援事業部　山田 博万

追加解析報告書の作成･変更履歴

日付	版番号	変更点
2019/09/06	1.0	初版作成

目次

1. FAS 2

1.1. トロポニン I 2
表 1.1. [FAS] トロポニン I 2

1.2. 心房細動症例を除外した部分集団での FMD 4
表 1.2. [FAS] 心房細動症例を除外した部分集団での FMD 4

1.3. ベースライン時 EF45%未満の部分集団での FMD、8-OHdG、XOR 活性、尿酸 5
表 1.3. [FAS] ベースライン時 EF45%未満の部分集団での FMD、8-OHdG、XOR 活性、尿酸 ... 5

1.4. ベースライン時 EF50%以上の部分集団での FMD、8-OHdG、XOR 活性、尿酸 8
表 1.4. [FAS] ベースライン時 EF50%以上の部分集団での FMD、8-OHdG、XOR 活性、尿酸 ... 8

2. PPS 11

2.1. トロポニン I 11
表 2.1. [PPS] トロポニン I 11

2.2. 心房細動症例を除外した部分集団での FMD 13
表 2.2. [PPS] 心房細動症例を除外した部分集団での FMD 13

2.3. ベースライン時 EF45%未満の部分集団での FMD、8-OHdG、XOR 活性、尿酸 14
表 2.3. [PPS] ベースライン時 EF45%未満の部分集団での FMD、8-OHdG、XOR 活性、尿酸 . 14

2.4. ベースライン時 EF50%以上の部分集団での FMD、8-OHdG、XOR 活性、尿酸 17
表 2.4. [PPS] ベースライン時 EF50%以上の部分集団での FMD、8-OHdG、XOR 活性、尿酸 . 17

1. FAS
1.1. トロポニン I

表 1.1. [FAS] トロポニン I

変数		観察ポイント	統計量	トピロキソスタット群	アロプリノール群	群間比較 P 値
hs トロポニン I (pg/mL)	測定値	0 週	n	70	70	
			Mean±SD	23.1±39.1	13.4±19.9	0.07
			Median [Q1, Q3]	7.4 [3.9, 17.1]	6.8 [4.4, 14.6]	0.63
			Min, Max	1.2, 158.8	1.4, 132.3	
		12 週	n	65	68	
			Mean±SD	23.3±47.3	15.8±28.5	0.27
			Median [Q1, Q3]	7.3 [3.9, 16.2]	6.6 [4.2, 19.0]	0.89
			Min, Max	1.5, 294.8	1.1, 197.9	
		24 週	n	66	66	
			Mean±SD	18.8±30.5	16.7±46.5	0.76
			Median [Q1, Q3]	7.9 [4.5, 15.9]	6.5 [4.0, 13.1]	0.45
			Min, Max	1.2, 145.0	1.5, 375.3	
	変化量	12 週	n	65	68	
			Mean±SD	3.1±41.2	2.5±24.2	0.92
			Median [Q1, Q3]	0.2 [-1.4, 2.3]	0.1 [-1.1, 1.4]	0.75
			Min, Max	-154.4, 287.7	-40.7, 190.4	
			One-sample t-test	0.55	0.40	
			Wilcoxon signed-rank test	0.39	0.56	
		24 週	n	66	66	
			Mean±SD	1.1±12.0	4.7±42.3	0.50
			Median [Q1, Q3]	0.7 [-0.8, 2.6]	-0.3 [-2.1, 1.1]	0.032
			Min, Max	-68.9, 42.3	-18.4, 340.7	
			One-sample t-test	0.46	0.37	
			Wilcoxon signed-rank test	0.021	0.54	
	変化率	12 週	n	65	68	
			Mean±SD	70.8±502.9	42.1±308.9	0.69
			Median [Q1, Q3]	4.0 [-12.8, 33.3]	1.3 [-18.0, 24.4]	0.39
			Min, Max	-97.2, 4052.1	-44.9, 2538.7	
			One-sample t-test	0.26	0.26	
			Wilcoxon signed-rank test	0.09	0.37	
		24 週	n	66	66	
			Mean±SD	21.0±46.2	19.7±125.7	0.94
			Median [Q1, Q3]	17.2 [-9.2, 43.2]	-5.7 [-17.2, 29.4]	0.042
			Min, Max	-89.7, 188.0	-53.6, 984.7	
			One-sample t-test	<0.001	0.21	
			Wilcoxon signed-rank test	<0.001	0.35	
対数変換 hs トロポニン I (ln (pg/mL))	測定値	0 週	n	70	70	
			Mean±SD	2.24±1.25	2.07±0.94	0.38
			Median [Q1, Q3]	1.99 [1.36, 2.84]	1.91 [1.48, 2.68]	0.63
			Min, Max	0.18, 5.07	0.34, 4.89	
		12 週	n	65	68	
			Mean±SD	2.19±1.23	2.11±1.03	0.68

			Median [Q1, Q3]	1.99 [1.36, 2.79]	1.88 [1.42, 2.94]	0.89
			Min, Max	0.41, 5.69	0.10, 5.29	
		24 週	n	66	66	
			Mean±SD	2.20±1.12	2.07±0.98	0.46
			Median [Q1, Q3]	2.06 [1.50, 2.77]	1.87 [1.39, 2.57]	0.45
			Min, Max	0.18, 4.98	0.41, 5.93	
	変化量	12 週	n	65	68	
			Mean±SD	0.04±0.73	0.05±0.49	0.93
			Median [Q1, Q3]	0.04 [−0.14, 0.29]	0.01 [−0.20, 0.22]	0.39
			Min, Max	−3.59, 3.73	−0.60, 3.27	
			One-sample t-test	0.62	0.36	
			Wilcoxon signed-rank test	0.20	0.79	
		24 週	n	66	66	
			Mean±SD	0.11±0.46	0.03±0.46	0.30
			Median [Q1, Q3]	0.16 [−0.10, 0.36]	−0.06 [−0.19, 0.26]	0.042
			Min, Max	−2.27, 1.06	−0.77, 2.38	
			One-sample t-test	0.06	0.65	
			Wilcoxon signed-rank test	0.003	0.85	
	変化率	12 週	n	65	68	
			Mean±SD	11.09±43.38	2.93±31.60	0.22
			Median [Q1, Q3]	1.48 [−7.97, 15.57]	0.70 [−10.28, 11.34]	0.33
			Min, Max	−70.76, 191.04	−82.04, 162.43	
			One-sample t-test	0.043	0.45	
			Wilcoxon signed-rank test	0.18	0.83	
		24 週	n	66	66	
			Mean±SD	16.41±59.72	5.92±31.79	0.21
			Median [Q1, Q3]	7.63 [−5.17, 19.86]	−2.92 [−10.48, 16.91]	0.08
			Min, Max	−92.56, 424.08	−44.54, 147.54	
			One-sample t-test	0.029	0.14	
			Wilcoxon signed-rank test	0.002	0.54	

1.2. 心房細動症例を除外した部分集団での FMD

除外した心房細動症例の定義は、心不全の原疾患もしくは合併症に心房細動があり、かつ登録時の心電図検査でも心房細動の所見があった症例、とした。

表 1.2. [FAS] 心房細動症例を除外した部分集団での FMD

変数		観察ポイント	統計量	トピロキソスタット群	アロプリノール群	群間比較P値
FMD(%)	測定値	0 週	n	32	33	
			Mean±SD	4.88±2.38	4.73±2.29	0.80
			Median [Q1, Q3]	4.65 [3.00, 6.00]	4.70 [3.40, 5.70]	0.94
			Min, Max	1.70, 11.50	1.10, 10.80	
		24 週	n	31	31	
			Mean±SD	4.98±2.45	4.53±1.71	0.40
			Median [Q1, Q3]	4.00 [3.50, 6.10]	4.20 [3.40, 5.60]	0.69
			Min, Max	1.50, 12.60	1.60, 9.20	
	変化量	24 週	n	30	30	
			Mean±SD	−0.01±1.40	−0.22±1.53	0.59
			Median [Q1, Q3]	0.10 [−0.80, 1.00]	−0.10 [−0.90, 0.60]	0.52
			Min, Max	−4.30, 3.00	−5.60, 2.40	
			One-sample t-test	0.97	0.45	
			Wilcoxon signed-rank test	0.83	0.53	

1.3. ベースライン時 EF45%未満の部分集団での FMD、8-OHdG、XOR 活性、尿酸

表 1.3. [FAS] ベースライン時 EF45%未満の部分集団での FMD、8-OHdG、XOR 活性、尿酸

変数		観察ポイント	統計量	トピロキソスタット群	アロプリノール群	群間比較 P 値
FMD(%)	測定値	0 週	n	14	15	
			Mean±SD	4.90±3.00	5.03±2.42	0.90
			Median [Q1, Q3]	3.10 [2.90, 7.50]	4.50 [4.00, 6.70]	0.53
			Min, Max	1.90, 11.50	1.10, 9.80	
		24 週	n	13	15	
			Mean±SD	5.68±2.98	4.86±1.89	0.38
			Median [Q1, Q3]	4.70 [4.00, 6.90]	4.10 [3.60, 6.00]	0.45
			Min, Max	1.50, 12.60	2.80, 9.20	
	変化量	24 週	n	12	14	
			Mean±SD	0.55±1.13	0.23±1.35	0.52
			Median [Q1, Q3]	0.65 [-0.30, 1.10]	-0.05 [-0.90, 1.60]	0.50
			Min, Max	-1.30, 3.00	-1.60, 2.40	
			One-sample t-test	0.12	0.54	
			Wilcoxon signed-rank test	0.13	0.62	
8-OHdG (ng/mg・Cr)	測定値	0 週	n	19	20	
			Mean±SD	8.4±3.6	7.2±2.9	0.24
			Median [Q1, Q3]	7.3 [5.4, 11.2]	6.6 [5.5, 8.5]	0.29
			Min, Max	3.4, 15.7	2.8, 13.5	
		12 週	n	17	20	
			Mean±SD	9.0±3.3	8.5±2.8	0.60
			Median [Q1, Q3]	8.4 [7.3, 10.3]	8.6 [5.8, 11.0]	0.90
			Min, Max	3.6, 16.8	4.3, 13.6	
		24 週	n	15	19	
			Mean±SD	8.6±3.9	10.0±2.1	0.19
			Median [Q1, Q3]	8.5 [6.0, 9.6]	10.5 [8.6, 11.8]	0.039
			Min, Max	4.1, 20.1	5.9, 12.7	
	変化量	12 週	n	17	20	
			Mean±SD	0.4±2.9	1.3±3.3	0.37
			Median [Q1, Q3]	1.7 [-1.0, 2.3]	2.0 [-1.3, 4.2]	0.34
			Min, Max	-5.8, 3.6	-5.5, 6.4	
			One-sample t-test	0.60	0.09	
			Wilcoxon signed-rank test	0.40	0.10	
		24 週	n	15	19	
			Mean±SD	0.4±3.8	2.8±3.1	0.05
			Median [Q1, Q3]	1.3 [-1.2, 2.6]	2.9 [1.0, 4.6]	0.08
			Min, Max	-9.9, 6.2	-2.3, 8.3	
			One-sample t-test	0.68	0.001	
			Wilcoxon signed-rank test	0.34	0.001	
	変化率	12 週	n	17	20	
			Mean±SD	14.2±37.8	32.1±55.5	0.27
			Median [Q1, Q3]	20.0 [-12.0, 31.5]	31.1 [-18.6, 74.0]	0.40
			Min, Max	-61.7, 81.8	-43.0, 127.8	
			One-sample t-test	0.14	0.018	

			Wilcoxon signed-rank test	0.12	0.036	
		24 週	n	15	19	
			Mean±SD	17.9±55.5	59.7±77.5	0.09
			Median [Q1, Q3]	15.7 [-16.4, 29.5]	51.5 [16.2, 63.9]	0.022
			Min, Max	-70.7, 182.4	-18.0, 296.4	
			One-sample t-test	0.23	0.004	
			Wilcoxon signed-rank test	0.23	<0.001	
XOR 活性 (pmol/h/mL plasma)	測定値	0 週	n	19	20	
			Mean±SD	48.1±39.9	44.7±46.9	0.81
			Median [Q1, Q3]	33.5 [22.5, 67.8]	30.9 [16.7, 49.6]	0.42
			Min, Max	13.6, 170.0	6.7, 201.0	
		24 週	n	15	19	
			Mean±SD	34.1±61.3	28.0±54.4	0.76
			Median [Q1, Q3]	17.3 [9.2, 20.6]	9.2 [6.7, 27.8]	0.19
			Min, Max	6.7, 250.0	6.7, 246.0	
	変化量	24 週	n	15	19	
			Mean±SD	-16.4±64.7	-18.7±53.8	0.91
			Median [Q1, Q3]	-15.9 [-42.1, -3.3]	-19.2 [-28.8, -5.7]	0.96
			Min, Max	-110.2, 182.2	-174.1, 138.0	
			One-sample t-test	0.34	0.15	
			Wilcoxon signed-rank test	0.010	0.002	
	変化率	24 週	n	15	19	
			Mean±SD	-30.6±87.4	-45.8±47.8	0.52
			Median [Q1, Q3]	-59.8 [-75.5, -9.3]	-56.1 [-70.7, -40.4]	0.84
			Min, Max	-84.0, 268.7	-86.6, 127.8	
			One-sample t-test	0.20	<0.001	
			Wilcoxon signed-rank test	0.010	0.002	
対数変換 XOR 活性(ln (pmol/h/mL plasma))	測定値	0 週	n	19	20	
			Mean±SD	3.6±0.7	3.4±0.9	0.39
			Median [Q1, Q3]	3.5 [3.1, 4.2]	3.4 [2.8, 3.9]	0.42
			Min, Max	2.6, 5.1	1.9, 5.3	
		24 週	n	15	19	
			Mean±SD	2.9±0.9	2.6±1.0	0.42
			Median [Q1, Q3]	2.9 [2.2, 3.0]	2.2 [1.9, 3.3]	0.19
			Min, Max	1.9, 5.5	1.9, 5.5	
	変化量	24 週	n	15	19	
			Mean±SD	-0.7±0.8	-0.8±0.7	0.71
			Median [Q1, Q3]	-0.9 [-1.4, -0.1]	-0.8 [-1.2, -0.5]	0.84
			Min, Max	-1.8, 1.3	-2.0, 0.8	
			One-sample t-test	0.003	<0.001	
			Wilcoxon signed-rank test	0.004	<0.001	
	変化率	24 週	n	15	19	
			Mean±SD	-20.2±20.0	-24.6±16.6	0.49
			Median [Q1, Q3]	-24.2 [-32.7, -3.1]	-30.2 [-35.7, -17.4]	0.45
			Min, Max	-44.1, 30.9	-46.5, 17.6	
			One-sample t-test	0.002	<0.001	
			Wilcoxon signed-rank test	0.003	<0.001	
血中尿酸値 (mg/dL)	測定値	0 週	n	19	20	
			Mean±SD	8.6±1.4	8.5±1.4	0.89

			Median [Q1, Q3]	8.3 [7.2, 10.1]	8.5 [7.6, 8.9]	0.98
			Min, Max	6.9, 11.9	6.7, 11.6	
		12 週	n	18	20	
			Mean±SD	5.8±1.2	6.0±0.8	0.55
			Median [Q1, Q3]	6.0 [5.2, 6.7]	6.1 [5.8, 6.5]	0.56
			Min, Max	3.9, 8.7	4.5, 7.4	
		24 週	n	15	19	
			Mean±SD	6.2±1.3	6.1±1.1	0.81
			Median [Q1, Q3]	6.3 [4.7, 7.3]	6.0 [5.2, 6.5]	0.75
			Min, Max	4.5, 7.8	4.5, 8.9	
	変化量	12 週	n	18	20	
			Mean±SD	-2.6±1.2	-2.5±1.2	0.85
			Median [Q1, Q3]	-2.3 [-3.5, -1.6]	-2.3 [-2.8, -1.8]	0.91
			Min, Max	-5.3, -1.2	-5.0, -1.0	
			One-sample t-test	<0.001	<0.001	
			Wilcoxon signed-rank test	<0.001	<0.001	
		24 週	n	15	19	
			Mean±SD	-2.2±1.3	-2.3±1.5	0.81
			Median [Q1, Q3]	-2.5 [-2.8, -0.7]	-2.2 [-3.1, -1.6]	0.94
			Min, Max	-4.6, 0.0	-5.7, 1.2	
			One-sample t-test	<0.001	<0.001	
			Wilcoxon signed-rank test	<0.001	<0.001	

1.4. ベースライン時 EF50％以上の部分集団での FMD、8-OHdG、XOR 活性、尿酸

表 1.4. [FAS] ベースライン時 EF50％以上の部分集団での FMD、8-OHdG、XOR 活性、尿酸

変数		観察ポイント	統計量	トピロキソスタット群	アロプリノール群	群間比較 P 値
FMD（％）	測定値	0 週	n	32	28	
			Mean±SD	4.67±2.20	4.25±2.00	0.44
			Median [Q1, Q3]	4.65 [3.10, 5.65]	4.50 [2.65, 5.15]	0.53
			Min, Max	1.10, 10.50	1.30, 10.80	
		24 週	n	32	26	
			Mean±SD	4.61±2.21	3.79±1.34	0.10
			Median [Q1, Q3]	3.95 [3.15, 5.85]	3.80 [2.90, 4.70]	0.26
			Min, Max	1.30, 10.70	1.20, 6.20	
	変化量	24 週	n	32	26	
			Mean±SD	-0.06±2.05	-0.65±1.43	0.22
			Median [Q1, Q3]	0.25 [-1.00, 1.20]	-0.50 [-0.90, 0.10]	0.09
			Min, Max	-6.60, 4.40	-5.60, 1.30	
			One-sample t-test	0.87	0.030	
			Wilcoxon signed-rank test	0.69	0.020	
8-OHdG (ng/mg・Cr)	測定値	0 週	n	44	43	
			Mean±SD	7.3±3.4	6.8±3.4	0.45
			Median [Q1, Q3]	6.8 [4.9, 8.3]	6.0 [4.3, 8.3]	0.32
			Min, Max	2.9, 22.3	2.1, 19.9	
		12 週	n	41	41	
			Mean±SD	9.1±4.1	10.6±6.2	0.20
			Median [Q1, Q3]	8.2 [6.5, 11.0]	9.1 [7.0, 12.3]	0.20
			Min, Max	4.1, 20.7	4.6, 37.9	
		24 週	n	44	40	
			Mean±SD	8.7±4.3	10.1±4.7	0.15
			Median [Q1, Q3]	8.3 [5.9, 9.9]	8.7 [7.4, 12.1]	0.11
			Min, Max	1.5, 24.9	3.6, 25.6	
	変化量	12 週	n	41	41	
			Mean±SD	1.6±3.6	3.7±4.7	0.028
			Median [Q1, Q3]	1.1 [-0.2, 2.9]	2.6 [0.9, 4.7]	0.010
			Min, Max	-6.5, 12.7	-2.6, 27.3	
			One-sample t-test	0.006	<0.001	
			Wilcoxon signed-rank test	0.001	<0.001	
		24 週	n	44	40	
			Mean±SD	1.3±3.4	3.4±3.2	0.005
			Median [Q1, Q3]	1.2 [-0.4, 2.6]	3.2 [1.4, 5.4]	0.004
			Min, Max	-7.3, 11.9	-1.8, 10.6	
			One-sample t-test	0.011	<0.001	
			Wilcoxon signed-rank test	0.005	<0.001	
	変化率	12 週	n	41	41	
			Mean±SD	29.9±50.2	64.6±70.5	0.012
			Median [Q1, Q3]	18.0 [-1.8, 50.9]	49.0 [13.2, 85.7]	0.010
			Min, Max	-46.7, 158.8	-27.1, 275.0	
			One-sample t-test	<0.001	<0.001	

			Wilcoxon signed-rank test	<0.001	<0.001	
		24 週	n	44	40	
			Mean±SD	24.3±47.0	64.0±64.1	0.002
			Median [Q1, Q3]	15.6 [-5.4, 44.1]	42.9 [20.5, 109.0]	0.004
			Min, Max	-83.0, 151.2	-25.0, 195.6	
			One-sample t-test	0.001	<0.001	
			Wilcoxon signed-rank test	0.001	<0.001	
XOR 活性 (pmol/h/mL plasma)	測定値	0 週	n	44	43	
			Mean±SD	59.0±59.1	77.6±154.5	0.46
			Median [Q1, Q3]	40.5 [26.9, 61.4]	28.9 [17.8, 57.5]	0.11
			Min, Max	16.3, 342.0	7.5, 946.0	
		24 週	n	44	40	
			Mean±SD	19.0±18.7	29.1±41.5	0.15
			Median [Q1, Q3]	10.0 [6.7, 25.0]	13.5 [8.8, 22.8]	0.14
			Min, Max	6.7, 83.4	6.7, 210.0	
	変化量	24 週	n	44	40	
			Mean±SD	-40.0±56.3	-53.0±141.1	0.57
			Median [Q1, Q3]	-22.3 [-45.0, -13.7]	-18.0 [-46.0, -8.2]	0.32
			Min, Max	-334.9, 15.9	-868.1, 65.0	
			One-sample t-test	<0.001	0.022	
			Wilcoxon signed-rank test	<0.001	<0.001	
	変化率	24 週	n	44	40	
			Mean±SD	-59.0±33.7	-43.0±47.6	0.08
			Median [Q1, Q3]	-69.8 [-81.6, -51.6]	-52.6 [-72.0, -36.5]	0.025
			Min, Max	-97.9, 59.6	-91.8, 130.0	
			One-sample t-test	<0.001	<0.001	
			Wilcoxon signed-rank test	<0.001	<0.001	
対数変換 XOR 活性(ln (pmol/h/mL plasma))	測定値	0 週	n	44	43	
			Mean±SD	3.8±0.7	3.6±1.1	0.34
			Median [Q1, Q3]	3.7 [3.3, 4.1]	3.4 [2.9, 4.1]	0.11
			Min, Max	2.8, 5.8	2.0, 6.9	
		24 週	n	44	40	
			Mean±SD	2.6±0.8	2.8±0.9	0.19
			Median [Q1, Q3]	2.3 [1.9, 3.2]	2.6 [2.2, 3.1]	0.14
			Min, Max	1.9, 4.4	1.9, 5.3	
	変化量	24 週	n	44	40	
			Mean±SD	-1.2±0.8	-0.8±0.7	0.036
			Median [Q1, Q3]	-1.2 [-1.7, -0.7]	-0.7 [-1.3, -0.5]	0.025
			Min, Max	-3.9, 0.5	-2.5, 0.8	
			One-sample t-test	<0.001	<0.001	
			Wilcoxon signed-rank test	<0.001	<0.001	
	変化率	24 週	n	44	40	
			Mean±SD	-30.7±18.7	-20.9±18.4	0.018
			Median [Q1, Q3]	-34.8 [-44.4, -18.4]	-23.2 [-33.3, -13.7]	0.017
			Min, Max	-66.3, 14.2	-55.8, 26.6	
			One-sample t-test	<0.001	<0.001	

			Wilcoxon signed-rank test	<0.001	<0.001	
血中尿酸値 (mg/dL)	測定値	0 週	n	43	42	
			Mean±SD	8.2±1.4	8.2±1.5	0.87
			Median [Q1, Q3]	8.0 [7.2, 9.2]	8.0 [7.3, 8.7]	0.89
			Min, Max	5.7, 11.6	5.4, 12.4	
		12 週	n	44	43	
			Mean±SD	5.2±1.0	5.9±1.2	0.008
			Median [Q1, Q3]	5.4 [4.8, 5.8]	5.9 [5.1, 6.5]	0.013
			Min, Max	3.2, 7.6	3.6, 9.0	
		24 週	n	44	41	
			Mean±SD	5.3±1.1	5.9±1.2	0.017
			Median [Q1, Q3]	5.3 [4.5, 6.1]	6.0 [5.0, 6.4]	0.023
			Min, Max	3.1, 7.5	3.1, 9.7	
	変化量	12 週	n	43	42	
			Mean±SD	−3.0±1.2	−2.3±1.4	0.013
			Median [Q1, Q3]	−2.7 [−3.8, −2.2]	−2.0 [−3.0, −1.4]	0.010
			Min, Max	−5.5, −0.9	−6.2, 0.7	
			One-sample t-test	<0.001	<0.001	
			Wilcoxon signed-rank test	<0.001	<0.001	
		24 週	n	43	40	
			Mean±SD	−2.9±1.5	−2.3±1.3	0.030
			Median [Q1, Q3]	−3.1 [−4.0, −1.8]	−1.9 [−3.2, −1.3]	0.013
			Min, Max	−6.6, 0.6	−5.7, 0.5	
			One-sample t-test	<0.001	<0.001	
			Wilcoxon signed-rank test	<0.001	<0.001	

2. PPS
2.1. トロポニン I

表 2.1. [PPS] トロポニン I

変数		観察ポイント	統計量	トピロキソスタット群	アロプリノール群	群間比較 P 値
hs トロポニン I (pg/mL)	測定値	0 週	n	64	67	
			Mean±SD	20.7±36.2	13.5±20.3	0.16
			Median [Q1, Q3]	7.2 [4.1, 17.0]	6.6 [4.3, 14.6]	0.68
			Min, Max	1.2, 158.8	1.4, 132.3	
		12 週	n	61	65	
			Mean±SD	24.1±48.6	14.7±27.5	0.18
			Median [Q1, Q3]	7.3 [4.1, 16.2]	6.1 [4.1, 18.3]	0.84
			Min, Max	1.5, 294.8	1.1, 197.9	
		24 週	n	61	63	
			Mean±SD	19.6±31.6	17.0±47.6	0.71
			Median [Q1, Q3]	8.0 [4.5, 16.2]	6.5 [4.0, 13.1]	0.37
			Min, Max	1.2, 145.0	1.5, 375.3	
	変化量	12 週	n	61	65	
			Mean±SD	3.1±42.6	3.2±24.2	0.99
			Median [Q1, Q3]	0.2 [-1.4, 2.3]	0.1 [-1.0, 1.3]	0.76
			Min, Max	-154.4, 287.7	-18.9, 190.4	
			One-sample t-test	0.57	0.30	
			Wilcoxon signed-rank test	0.45	0.62	
		24 週	n	61	63	
			Mean±SD	1.2±12.5	4.9±43.3	0.52
			Median [Q1, Q3]	1.0 [-0.7, 2.6]	-0.2 [-2.1, 1.1]	0.024
			Min, Max	-68.9, 42.3	-18.4, 340.7	
			One-sample t-test	0.47	0.37	
			Wilcoxon signed-rank test	0.020	0.52	
	変化率	12 週	n	61	65	
			Mean±SD	74.1±519.2	43.7±315.9	0.69
			Median [Q1, Q3]	4.0 [-14.6, 27.8]	0.8 [-17.6, 23.4]	0.44
			Min, Max	-97.2, 4052.1	-44.9, 2538.7	
			One-sample t-test	0.27	0.27	
			Wilcoxon signed-rank test	0.13	0.42	
		24 週	n	61	63	
			Mean±SD	20.5±45.7	20.1±128.5	0.98
			Median [Q1, Q3]	18.1 [-9.2, 43.2]	-5.4 [-17.2, 29.4]	0.048
			Min, Max	-89.7, 188.0	-53.6, 984.7	
			One-sample t-test	<0.001	0.22	
			Wilcoxon signed-rank test	<0.001	0.38	
対数変換 hs トロポニン I (ln (pg/mL))	測定値	0 週	n	64	67	
			Mean±SD	2.19±1.19	2.06±0.96	0.51
			Median [Q1, Q3]	1.97 [1.41, 2.83]	1.89 [1.46, 2.68]	0.68
			Min, Max	0.18, 5.07	0.34, 4.89	
		12 週	n	61	65	
			Mean±SD	2.21±1.23	2.06±1.01	0.44

			Median [Q1, Q3]	1.99 [1.41, 2.79]	1.81 [1.41, 2.91]	0.84
			Min, Max	0.41, 5.69	0.10, 5.29	
		24 週	n	61	63	
			Mean±SD	2.23±1.15	2.06±1.00	0.38
			Median [Q1, Q3]	2.08 [1.50, 2.79]	1.87 [1.39, 2.57]	0.37
			Min, Max	0.18, 4.98	0.41, 5.93	
	変化量	12 週	n	61	65	
			Mean±SD	0.04±0.75	0.06±0.49	0.88
			Median [Q1, Q3]	0.04 [−0.16, 0.25]	0.01 [−0.19, 0.21]	0.44
			Min, Max	−3.59, 3.73	−0.60, 3.27	
			One-sample t-test	0.69	0.37	
			Wilcoxon signed-rank test	0.25	0.81	
		24 週	n	61	63	
			Mean±SD	0.10±0.46	0.02±0.46	0.34
			Median [Q1, Q3]	0.17 [−0.10, 0.36]	−0.06 [−0.19, 0.26]	0.048
			Min, Max	−2.27, 1.06	−0.77, 2.38	
			One-sample t-test	0.09	0.69	
			Wilcoxon signed-rank test	0.005	0.89	
	変化率	12 週	n	61	65	
			Mean±SD	10.99±44.37	2.87±32.27	0.24
			Median [Q1, Q3]	1.48 [−7.97, 13.63]	0.31 [−10.90, 10.92]	0.34
			Min, Max	−70.76, 191.04	−82.04, 162.43	
			One-sample t-test	0.06	0.48	
			Wilcoxon signed-rank test	0.23	0.91	
		24 週	n	61	63	
			Mean±SD	15.84±61.25	6.02±32.41	0.26
			Median [Q1, Q3]	7.85 [−5.17, 18.55]	−2.78 [−11.86, 16.91]	0.10
			Min, Max	−92.56, 424.08	−44.54, 147.54	
			One-sample t-test	0.048	0.15	
			Wilcoxon signed-rank test	0.004	0.56	

2.2. 心房細動症例を除外した部分集団での FMD

除外した心房細動症例の定義は、心不全の原疾患もしくは合併症に心房細動があり、かつ登録時の心電図検査でも心房細動の所見があった症例、とした。

表 2.2. [PPS] 心房細動症例を除外した部分集団での FMD

変数		観察ポイント	統計量	トピロキソスタット群	アロプリノール群	群間比較 P 値
FMD（%）	測定値	0 週	n	29	31	
			Mean±SD	4.84±2.38	4.86±2.27	0.97
			Median [Q1, Q3]	4.60 [3.10, 5.80]	4.80 [3.40, 5.80]	0.78
			Min, Max	1.70, 11.50	1.10, 10.80	
		24 週	n	29	29	
			Mean±SD	4.99±2.51	4.54±1.72	0.42
			Median [Q1, Q3]	4.00 [3.50, 6.10]	4.20 [3.40, 5.30]	0.77
			Min, Max	1.50, 12.60	1.60, 9.20	
	変化量	24 週	n	28	28	
			Mean±SD	0.13±1.18	−0.35±1.49	0.19
			Median [Q1, Q3]	0.10 [−0.70, 1.00]	−0.35 [−0.95, 0.40]	0.22
			Min, Max	−1.90, 3.00	−5.60, 2.40	
			One-sample t-test	0.57	0.22	
			Wilcoxon signed-rank test	0.63	0.20	

2.3. ベースライン時 EF45%未満の部分集団での FMD、8-OHdG、XOR 活性、尿酸

表 2.3. [PPS] ベースライン時 EF45%未満の部分集団での FMD、8-OHdG、XOR 活性、尿酸

変数		観察ポイント	統計量	トピロキソスタット群	アロプリノール群	群間比較 P 値
FMD(%)	測定値	0 週	n	12	13	
			Mean±SD	5.28±3.09	5.38±2.32	0.92
			Median [Q1, Q3]	4.30 [2.90, 7.55]	4.70 [4.00, 6.70]	0.61
			Min, Max	1.90, 11.50	1.10, 9.80	
		24 週	n	12	13	
			Mean±SD	5.79±3.09	4.93±1.92	0.41
			Median [Q1, Q3]	5.30 [3.75, 7.80]	4.10 [3.60, 5.20]	0.53
			Min, Max	1.50, 12.60	3.40, 9.20	
	変化量	24 週	n	11	12	
			Mean±SD	0.48±1.16	-0.02±1.31	0.35
			Median [Q1, Q3]	0.60 [-0.40, 1.10]	-0.15 [-0.95, 0.50]	0.24
			Min, Max	-1.30, 3.00	-1.60, 2.40	
			One-sample t-test	0.20	0.97	
			Wilcoxon signed-rank test	0.23	0.72	
8-OHdG (ng/mg・Cr)	測定値	0 週	n	16	18	
			Mean±SD	8.8±3.8	7.3±3.0	0.20
			Median [Q1, Q3]	8.3 [5.4, 11.7]	6.7 [5.7, 9.2]	0.27
			Min, Max	3.4, 15.7	2.8, 13.5	
		12 週	n	16	17	
			Mean±SD	9.0±3.5	8.7±2.7	0.76
			Median [Q1, Q3]	8.2 [7.2, 10.5]	8.6 [7.3, 10.9]	0.84
			Min, Max	3.6, 16.8	4.3, 13.6	
		24 週	n	14	17	
			Mean±SD	8.6±4.0	10.2±2.0	0.17
			Median [Q1, Q3]	8.5 [6.0, 9.6]	10.5 [8.7, 11.8]	0.045
			Min, Max	4.1, 20.1	5.9, 12.7	
	変化量	12 週	n	16	17	
			Mean±SD	0.2±2.9	1.3±3.3	0.30
			Median [Q1, Q3]	1.2 [-1.4, 2.3]	2.1 [-1.2, 4.2]	0.23
			Min, Max	-5.8, 3.6	-5.5, 5.4	
			One-sample t-test	0.80	0.12	
			Wilcoxon signed-rank test	0.55	0.12	
		24 週	n	14	17	
			Mean±SD	0.2±3.8	2.8±3.2	0.05
			Median [Q1, Q3]	1.0 [-1.2, 2.3]	2.9 [1.2, 4.6]	0.07
			Min, Max	-9.9, 6.2	-2.3, 8.3	
			One-sample t-test	0.83	0.003	
			Wilcoxon signed-rank test	0.49	0.002	
	変化率	12 週	n	16	17	
			Mean±SD	11.1±36.7	32.8±53.3	0.19
			Median [Q1, Q3]	16.5 [-13.5, 30.4]	33.8 [-16.0, 73.1]	0.26
			Min, Max	-61.7, 81.8	-43.0, 127.8	
			One-sample t-test	0.24	0.022	

			Wilcoxon signed-rank test	0.21	0.034	
		24 週	n	14	17	
			Mean±SD	15.3±56.6	62.1±81.5	0.08
			Median [Q1, Q3]	13.4 [-16.4, 28.0]	51.5 [16.2, 63.9]	0.018
			Min, Max	-70.7, 182.4	-18.0, 296.4	
			One-sample t-test	0.33	0.006	
			Wilcoxon signed-rank test	0.36	<0.001	
XOR 活性 (pmol/h/mL plasma)	測定値	0 週	n	16	18	
			Mean±SD	51.9±42.3	42.4±46.7	0.54
			Median [Q1, Q3]	38.3 [22.6, 70.9]	30.9 [15.3, 41.8]	0.22
			Min, Max	13.6, 170.0	6.7, 201.0	
		24 週	n	14	17	
			Mean±SD	35.5±63.4	16.4±13.7	0.23
			Median [Q1, Q3]	17.6 [9.2, 20.6]	9.2 [6.7, 26.9]	0.17
			Min, Max	6.7, 250.0	6.7, 47.8	
	変化量	24 週	n	14	17	
			Mean±SD	-16.8±67.1	-28.1±40.4	0.57
			Median [Q1, Q3]	-18.5 [-42.1, -3.3]	-21.1 [-28.8, -8.6]	0.87
			Min, Max	-110.2, 182.2	-174.1, 0.0	
			One-sample t-test	0.37	0.011	
			Wilcoxon signed-rank test	0.017	<0.001	
	変化率	24 週	n	14	17	
			Mean±SD	-29.7±90.6	-54.5±23.8	0.29
			Median [Q1, Q3]	-61.4 [-75.5, -9.3]	-56.1 [-67.7, -46.2]	0.86
			Min, Max	-84.0, 268.7	-86.6, 0.0	
			One-sample t-test	0.24	<0.001	
			Wilcoxon signed-rank test	0.017	<0.001	
対数変換 XOR 活性(ln (pmol/h/mL plasma))	測定値	0 週	n	16	18	
			Mean±SD	3.7±0.7	3.3±0.9	0.22
			Median [Q1, Q3]	3.6 [3.1, 4.3]	3.4 [2.7, 3.7]	0.22
			Min, Max	2.6, 5.1	1.9, 5.3	
		24 週	n	14	17	
			Mean±SD	2.9±1.0	2.5±0.7	0.19
			Median [Q1, Q3]	2.9 [2.2, 3.0]	2.2 [1.9, 3.3]	0.17
			Min, Max	1.9, 5.5	1.9, 3.9	
	変化量	24 週	n	14	17	
			Mean±SD	-0.8±0.8	-0.9±0.5	0.54
			Median [Q1, Q3]	-1.0 [-1.4, -0.1]	-0.8 [-1.1, -0.6]	0.86
			Min, Max	-1.8, 1.3	-2.0, 0.0	
			One-sample t-test	0.005	<0.001	
			Wilcoxon signed-rank test	0.007	<0.001	
	変化率	24 週	n	14	17	
			Mean±SD	-20.4±20.8	-26.3±13.5	0.35
			Median [Q1, Q3]	-25.6 [-32.7, -3.1]	-30.2 [-34.5, -17.6]	0.49
			Min, Max	-44.1, 30.9	-46.5, 0.0	
			One-sample t-test	0.003	<0.001	
			Wilcoxon signed-rank test	0.005	<0.001	
血中尿酸値 (mg/dL)	測定値	0 週	n	16	18	
			Mean±SD	8.5±1.3	8.5±1.3	0.97

2.3. ベースライン時 EF45%未満の部分集団での FMD、8-OHdG、XOR 活性、尿酸

			Median [Q1, Q3]	8.4 [7.2, 9.6]	8.5 [7.6, 8.8]	0.97
			Min, Max	6.9, 10.6	6.7, 11.6	
		12 週	n	16	17	
			Mean±SD	5.9±1.2	6.0±0.9	0.78
			Median [Q1, Q3]	6.0 [5.3, 6.7]	6.0 [5.7, 6.4]	0.76
			Min, Max	3.9, 8.7	4.5, 7.4	
		24 週	n	14	17	
			Mean±SD	6.1±1.3	6.0±0.9	0.74
			Median [Q1, Q3]	6.3 [4.7, 7.2]	6.0 [5.3, 6.4]	0.80
			Min, Max	4.5, 7.8	4.5, 7.9	
	変化量	12 週	n	16	17	
			Mean±SD	−2.6±1.2	−2.3±0.9	0.46
			Median [Q1, Q3]	−2.3 [−3.4, −1.7]	−2.3 [−2.7, −1.8]	0.68
			Min, Max	−5.3, −1.2	−4.4, −1.0	
			One-sample t-test	<0.001	<0.001	
			Wilcoxon signed-rank test	<0.001	<0.001	
		24 週	n	14	17	
			Mean±SD	−2.3±1.3	−2.3±1.0	0.97
			Median [Q1, Q3]	−2.6 [−2.8, −1.7]	−2.2 [−3.0, −1.7]	0.86
			Min, Max	−4.6, 0.0	−4.4, −0.4	
			One-sample t-test	<0.001	<0.001	
			Wilcoxon signed-rank test	<0.001	<0.001	

2.4. ベースライン時 EF50%以上の部分集団での FMD、8-OHdG、XOR 活性、尿酸

表 2.4. [PPS] ベースライン時 EF50%以上の部分集団での FMD、8-OHdG、XOR 活性、尿酸

変数		観察ポイント	統計量	トピロキソスタット群	アロプリノール群	群間比較 P 値
FMD(%)	測定値	0 週	n	30	27	
			Mean±SD	4.53±2.18	4.36±1.95	0.75
			Median [Q1, Q3]	4.60 [3.00, 5.50]	4.50 [2.90, 5.20]	0.94
			Min, Max	1.10, 10.50	1.30, 10.80	
		24 週	n	30	26	
			Mean±SD	4.60±2.26	3.79±1.34	0.12
			Median [Q1, Q3]	3.95 [3.00, 5.60]	3.80 [2.90, 4.70]	0.30
			Min, Max	1.30, 10.70	1.20, 6.20	
	変化量	24 週	n	30	26	
			Mean±SD	0.07±1.96	-0.65±1.43	0.13
			Median [Q1, Q3]	0.25 [-0.90, 1.20]	-0.50 [-0.90, 0.10]	0.06
			Min, Max	-6.60, 4.40	-5.60, 1.30	
			One-sample t-test	0.85	0.030	
			Wilcoxon signed-rank test	0.53	0.020	
8-OHdG (ng/mg・Cr)	測定値	0 週	n	42	42	
			Mean±SD	7.4±3.4	6.9±3.4	0.44
			Median [Q1, Q3]	6.9 [5.4, 8.3]	6.1 [4.5, 8.3]	0.32
			Min, Max	2.9, 22.3	2.1, 19.9	
		12 週	n	39	41	
			Mean±SD	9.1±4.1	10.6±6.2	0.22
			Median [Q1, Q3]	8.2 [6.5, 11.0]	9.1 [7.0, 12.3]	0.21
			Min, Max	4.1, 20.7	4.6, 37.9	
		24 週	n	41	40	
			Mean±SD	8.7±4.5	10.1±4.7	0.17
			Median [Q1, Q3]	8.3 [5.7, 9.5]	8.7 [7.4, 12.1]	0.12
			Min, Max	1.5, 24.9	3.6, 25.6	
	変化量	12 週	n	39	41	
			Mean±SD	1.5±3.5	3.7±4.7	0.022
			Median [Q1, Q3]	1.1 [-0.2, 2.9]	2.6 [0.9, 4.7]	0.006
			Min, Max	-6.5, 12.7	-2.6, 27.3	
			One-sample t-test	0.013	<0.001	
			Wilcoxon signed-rank test	0.004	<0.001	
		24 週	n	41	40	
			Mean±SD	1.2±3.4	3.4±3.2	0.003
			Median [Q1, Q3]	1.1 [-0.4, 2.5]	3.2 [1.4, 5.4]	0.002
			Min, Max	-7.3, 11.9	-1.8, 10.6	
			One-sample t-test	0.031	<0.001	
			Wilcoxon signed-rank test	0.017	<0.001	
	変化率	12 週	n	39	41	
			Mean±SD	26.9±47.1	64.6±70.5	0.007
			Median [Q1, Q3]	16.1 [-3.0, 50.9]	49.0 [13.2, 85.7]	0.006
			Min, Max	-46.7, 158.8	-27.1, 275.0	
			One-sample t-test	<0.001	<0.001	

			Wilcoxon signed-rank test	<0.001	<0.001	
		24 週	n	41	40	
			Mean±SD	20.4±45.1	64.0±64.1	<0.001
			Median [Q1, Q3]	13.2 [-5.5, 39.8]	42.9 [20.5, 109.0]	0.002
			Min, Max	-83.0, 151.2	-25.0, 195.6	
			One-sample t-test	0.006	<0.001	
			Wilcoxon signed-rank test	0.006	<0.001	
XOR 活性 (pmol/h/mL plasma)	測定値	0 週	n	42	42	
			Mean±SD	60.5±60.0	79.0±156.1	0.48
			Median [Q1, Q3]	40.9 [27.1, 61.5]	29.2 [17.8, 57.5]	0.11
			Min, Max	16.3, 342.0	7.5, 946.0	
		24 週	n	41	40	
			Mean±SD	19.7±19.2	29.1±41.5	0.20
			Median [Q1, Q3]	9.7 [6.7, 27.4]	13.5 [8.8, 22.8]	0.21
			Min, Max	6.7, 83.4	6.7, 210.0	
	変化量	24 週	n	41	40	
			Mean±SD	-40.9±58.1	-53.0±141.1	0.61
			Median [Q1, Q3]	-21.9 [-45.7, -14.1]	-18.0 [-46.0, -8.2]	0.31
			Min, Max	-334.9, 15.9	-868.1, 65.0	
			One-sample t-test	<0.001	0.022	
			Wilcoxon signed-rank test	<0.001	<0.001	
	変化率	24 週	n	41	40	
			Mean±SD	-58.2±34.6	-43.0±47.6	0.10
			Median [Q1, Q3]	-69.2 [-82.0, -52.3]	-52.6 [-72.0, -36.5]	0.036
			Min, Max	-97.9, 59.6	-91.8, 130.0	
			One-sample t-test	<0.001	<0.001	
			Wilcoxon signed-rank test	<0.001	<0.001	
対数変換 XOR 活性(ln (pmol/h/mL plasma))	測定値	0 週	n	42	42	
			Mean±SD	3.8±0.7	3.6±1.1	0.33
			Median [Q1, Q3]	3.7 [3.3, 4.1]	3.4 [2.9, 4.1]	0.11
			Min, Max	2.8, 5.8	2.0, 6.9	
		24 週	n	41	40	
			Mean±SD	2.6±0.8	2.8±0.9	0.26
			Median [Q1, Q3]	2.3 [1.9, 3.3]	2.6 [2.2, 3.1]	0.21
			Min, Max	1.9, 4.4	1.9, 5.3	
	変化量	24 週	n	41	40	
			Mean±SD	-1.2±0.9	-0.8±0.7	0.045
			Median [Q1, Q3]	-1.2 [-1.7, -0.7]	-0.7 [-1.3, -0.5]	0.036
			Min, Max	-3.9, 0.5	-2.5, 0.8	
			One-sample t-test	<0.001	<0.001	
			Wilcoxon signed-rank test	<0.001	<0.001	
	変化率	24 週	n	41	40	
			Mean±SD	-30.4±19.2	-20.9±18.4	0.027
			Median [Q1, Q3]	-34.1 [-44.0, -17.6]	-23.2 [-33.3, -13.7]	0.025
			Min, Max	-66.3, 14.2	-55.8, 26.6	
			One-sample t-test	<0.001	<0.001	

			Wilcoxon signed-rank test	<0.001	<0.001	
血中尿酸値 (mg/dL)	測定値	0 週	n	41	41	
			Mean±SD	8.2±1.4	8.2±1.5	0.93
			Median [Q1, Q3]	8.0 [7.2, 9.1]	8.0 [7.3, 8.7]	0.93
			Min, Max	5.7, 11.6	5.4, 12.4	
		12 週	n	41	42	
			Mean±SD	5.2±1.0	5.9±1.2	0.003
			Median [Q1, Q3]	5.4 [4.7, 5.8]	5.9 [5.2, 6.5]	0.006
			Min, Max	3.2, 6.9	3.6, 9.0	
		24 週	n	41	40	
			Mean±SD	5.2±1.1	6.0±1.2	0.005
			Median [Q1, Q3]	5.2 [4.6, 6.0]	6.0 [5.1, 6.4]	0.009
			Min, Max	3.1, 7.5	3.6, 9.7	
	変化量	12 週	n	40	41	
			Mean±SD	−3.0±1.2	−2.2±1.4	0.011
			Median [Q1, Q3]	−2.9 [−3.8, −2.2]	−2.0 [−2.9, −1.4]	0.008
			Min, Max	−5.5, −0.9	−6.2, 0.7	
			One-sample t-test	<0.001	<0.001	
			Wilcoxon signed-rank test	<0.001	<0.001	
		24 週	n	40	39	
			Mean±SD	−2.9±1.5	−2.2±1.3	0.028
			Median [Q1, Q3]	−3.2 [−3.9, −1.9]	−1.9 [−3.2, −1.3]	0.012
			Min, Max	−6.6, 0.6	−5.7, 0.5	
			One-sample t-test	<0.001	<0.001	
			Wilcoxon signed-rank test	<0.001	<0.001	
