# Supplementary material for: Topiroxostat versus allopurinol in patients with chronic heart failure complicated by hyperuricemia: A prospective, randomized, open-label, blinded-end-point clinical trial
Source: PLoS One. 2022 Jan 25;17(1):e0261445. doi: 10.1371/journal.pone.0261445 (PMC8789120; doi:10.1371/journal.pone.0261445)
Supplement: S3 Appendix — (PDF) [file pone.0261445.s010.pdf]

高尿酸血症を合併した心不全患者における
トピロキソスタットとアロプリノールのランダム化比較試験

The **E**ffect of **X**anthine Oxidase Inhibitor
in **C**hron**I**c heart failure patients
complica**TED** with hyper-**U**ricemi**A**

Excited-UA study

追加解析報告書
Ver 1.2

研究代表者 ：
　獨協医科大学病院 心臓･血管内科 教授　井上 晃男

統計解析責任者 ：
　株式会社 総合医科学研究所 医薬臨床研究支援事業部　山田 博万

追加解析報告書の作成･変更履歴

日付	版番号	変更点
2019/09/06	1.0	初版作成
2019/10/01	1.1	全解析から心房細動症例を除外して再解析
2019/11/05	1.2	相関解析の追加

目次

1. FAS から心房細動症例を除外した集団 1

1.1. トロポニン I 1
表 1.1.1. [FAS] トロポニン I 1

1.2. FMD 3
表 1.2.1. [FAS] FMD 3
表 1.2.2. [FAS] FMD の変化量と EndoPAT の変化量の相関 3

1.3. ベースライン時 EF45%未満の部分集団での FMD、8-OHdG、XOR 活性、尿酸 4
表 1.3.1. [FAS] ベースライン時 EF45%未満の部分集団での FMD、8-OHdG、XOR 活性、尿酸 4
表 1.3.2. [FAS] ベースライン時 EF45%未満の部分集団での FMD の変化量と EndoPAT の変化量の相関 6

1.4. ベースライン時 EF50%以上の部分集団での FMD、8-OHdG、XOR 活性、尿酸 7
表 1.4.1. [FAS] ベースライン時 EF50%以上の部分集団での FMD、8-OHdG、XOR 活性、尿酸 7
表 1.4.2. [FAS] ベースライン時 EF50%以上の部分集団での FMD の変化量と EndoPAT の変化量の相関 9

2. PPS から心房細動除症例を除外した集団 10

2.1. トロポニン I 10
表 2.1.1. [PPS] トロポニン I 10

2.2. FMD 12
表 2.2.1. [PPS] FMD 12
表 2.2.2. [PPS] FMD の変化量と EndoPAT の変化量の相関 12

2.3. ベースライン時 EF45%未満の部分集団での FMD、8-OHdG、XOR 活性、尿酸 13
表 2.3.1. [PPS] ベースライン時 EF45%未満の部分集団での FMD、8-OHdG、XOR 活性、尿酸 13
表 2.3.2. [PPS] ベースライン時 EF45%未満の部分集団での FMD の変化量と EndoPAT の変化量の相関 15

2.4. ベースライン時 EF50%以上の部分集団での FMD、8-OHdG、XOR 活性、尿酸 16
表 2.4.1. [PPS] ベースライン時 EF50%以上の部分集団での FMD、8-OHdG、XOR 活性、尿酸 16
表 2.4.2. [PPS] ベースライン時 EF50%以上の部分集団での FMD の変化量と EndoPAT の変化量の相関 18

1. FAS から心房細動症例を除外した集団

除外した心房細動症例の定義は、心不全の原疾患もしくは合併症に心房細動があり、かつ登録時の心電図検査でも心房細動の所見があった症例、とした。

1.1. トロポニン I

表 1.1.1. [FAS] トロポニン I

変数		観察ポイント	統計量	トピロキソスタット群	アロプリノール群	群間比較 P 値
hs トロポニン I (pg/mL)	測定値	0 週	n	40	45	
			Mean±SD	27.5±41.9	17.9±23.7	0.19
			Median [Q1, Q3]	8.5 [4.9, 29.4]	7.9 [6.4, 17.9]	1.00
			Min, Max	1.5, 152.7	1.6, 132.3	
		12 週	n	39	43	
			Mean±SD	34.0±58.6	21.6±34.5	0.24
			Median [Q1, Q3]	9.6 [4.6, 29.2]	9.4 [5.1, 21.9]	1.00
			Min, Max	2.0, 294.8	1.1, 197.9	
		24 週	n	38	42	
			Mean±SD	26.3±38.2	22.7±57.6	0.75
			Median [Q1, Q3]	9.2 [5.7, 24.8]	7.7 [4.5, 21.1]	0.48
			Min, Max	1.8, 145.0	1.5, 375.3	
	変化量	12 週	n	39	43	
			Mean±SD	9.2±46.5	3.7±30.5	0.52
			Median [Q1, Q3]	0.3 [-1.0, 3.8]	0.1 [-1.7, 2.0]	0.30
			Min, Max	-17.9, 287.7	-40.7, 190.4	
			One-sample t-test	0.23	0.43	
			Wilcoxon signed-rank test	0.14	0.90	
		24 週	n	38	42	
			Mean±SD	1.7±15.6	7.1±53.1	0.55
			Median [Q1, Q3]	1.2 [-0.6, 3.3]	-0.5 [-2.9, 0.8]	0.012
			Min, Max	-68.9, 42.3	-18.4, 340.7	
			One-sample t-test	0.50	0.39	
			Wilcoxon signed-rank test	0.038	0.13	
	変化率	12 週	n	39	43	
			Mean±SD	115.0±647.9	62.6±388.0	0.65
			Median [Q1, Q3]	6.4 [-11.5, 27.1]	1.9 [-18.9, 20.5]	0.22
			Min, Max	-55.9, 4052.1	-44.9, 2538.7	
			One-sample t-test	0.27	0.30	
			Wilcoxon signed-rank test	0.06	0.70	
		24 週	n	38	42	
			Mean±SD	22.0±39.8	20.6±156.3	0.96
			Median [Q1, Q3]	20.1 [-5.0, 43.2]	-9.2 [-23.0, 18.2]	0.003
			Min, Max	-49.4, 126.9	-53.6, 984.7	
			One-sample t-test	0.002	0.40	
			Wilcoxon signed-rank test	0.001	0.37	
対数変換 hs トロポニン I (ln (pg/mL))	測定値	0 週	n	40	45	
			Mean±SD	2.46±1.27	2.37±0.97	0.72
			Median [Q1, Q3]	2.14 [1.59, 3.37]	2.07 [1.86, 2.88]	1.00

			Min, Max	0.41, 5.03	0.47, 4.89	
		12 週	n	39	43	
			Mean±SD	2.55±1.33	2.43±1.09	0.64
			Median [Q1, Q3]	2.26 [1.53, 3.37]	2.24 [1.63, 3.09]	1.00
			Min, Max	0.69, 5.69	0.10, 5.29	
		24 週	n	38	42	
			Mean±SD	2.51±1.17	2.30±1.10	0.41
			Median [Q1, Q3]	2.22 [1.74, 3.21]	2.03 [1.50, 3.05]	0.48
			Min, Max	0.59, 4.98	0.41, 5.93	
	変化量	12 週	n	39	43	
			Mean±SD	0.16±0.66	0.07±0.58	0.51
			Median [Q1, Q3]	0.06 [-0.12, 0.24]	0.02 [-0.21, 0.19]	0.22
			Min, Max	-0.82, 3.73	-0.60, 3.27	
			One-sample t-test	0.14	0.45	
			Wilcoxon signed-rank test	0.11	0.99	
		24 週	n	38	42	
			Mean±SD	0.15±0.34	-0.03±0.51	0.08
			Median [Q1, Q3]	0.18 [-0.05, 0.36]	-0.10 [-0.26, 0.17]	0.003
			Min, Max	-0.68, 0.82	-0.77, 2.38	
			One-sample t-test	0.011	0.71	
			Wilcoxon signed-rank test	0.005	0.17	
	変化率	12 週	n	39	43	
			Mean±SD	11.85±39.33	2.89±31.08	0.25
			Median [Q1, Q3]	2.00 [-4.79, 9.10]	1.09 [-7.81, 7.22]	0.32
			Min, Max	-42.69, 190.10	-82.04, 162.43	
			One-sample t-test	0.07	0.55	
			Wilcoxon signed-rank test	0.16	0.81	
		24 週	n	38	42	
			Mean±SD	12.16±24.17	-1.52±21.16	0.009
			Median [Q1, Q3]	7.63 [-1.11, 15.54]	-4.70 [-12.96, 8.85]	0.004
			Min, Max	-29.32, 85.76	-44.54, 67.27	
			One-sample t-test	0.004	0.64	
			Wilcoxon signed-rank test	0.004	0.26	

1.2. FMD

表 1.2.1. [FAS] FMD

変数		観察ポイント	統計量	トピロキソスタット群	アロプリノール群	群間比較 P 値
FMD（%）	測定値	0 週	n	32	33	
			Mean±SD	4.88±2.38	4.73±2.29	0.80
			Median [Q1, Q3]	4.65 [3.00, 6.00]	4.70 [3.40, 5.70]	0.94
			Min, Max	1.70, 11.50	1.10, 10.80	
		24 週	n	31	31	
			Mean±SD	4.98±2.45	4.53±1.71	0.40
			Median [Q1, Q3]	4.00 [3.50, 6.10]	4.20 [3.40, 5.60]	0.69
			Min, Max	1.50, 12.60	1.60, 9.20	
	変化量	24 週	n	30	30	
			Mean±SD	−0.01±1.40	−0.22±1.53	0.59
			Median [Q1, Q3]	0.10 [−0.80, 1.00]	−0.10 [−0.90, 0.60]	0.52
			Min, Max	−4.30, 3.00	−5.60, 2.40	
			One-sample t-test	0.97	0.45	
			Wilcoxon signed-rank test	0.83	0.53	

表 1.2.2. [FAS] FMD の変化量と EndoPAT の変化量の相関

変数 1	変数 2	n	Pearson		Spearman	
			相関係数 (95%CI)	P 値	相関係数 (95%CI)	P 値
FMD（%） 24 週変化量	EndoPAT（RHI 指数） 24 週変化量	60	0.25 (−0.01, 0.47)	0.06	0.26 (0.00, 0.48)	0.048

1.3. ベースライン時 EF45%未満の部分集団での FMD、8-OHdG、XOR 活性、尿酸

表 1.3.1. [FAS] ベースライン時 EF45%未満の部分集団での FMD、8-OHdG、XOR 活性、尿酸

変数		観察ポイント	統計量	トピロキソスタット群	アロプリノール群	群間比較 P 値
FMD(%)	測定値	0 週	n	12	13	
			Mean±SD	5.28±3.09	5.40±2.31	0.91
			Median [Q1, Q3]	4.30 [2.90, 7.55]	4.70 [4.20, 6.70]	0.61
			Min, Max	1.90, 11.50	1.10, 9.80	
		24 週	n	11	13	
			Mean±SD	6.06±3.08	5.09±1.91	0.36
			Median [Q1, Q3]	5.90 [4.00, 8.70]	4.20 [3.60, 6.00]	0.42
			Min, Max	1.50, 12.60	3.40, 9.20	
	変化量	24 週	n	10	12	
			Mean±SD	0.47±1.22	0.14±1.41	0.57
			Median [Q1, Q3]	0.65 [-0.40, 1.10]	-0.10 [-0.95, 1.30]	0.47
			Min, Max	-1.30, 3.00	-1.60, 2.40	
			One-sample t-test	0.25	0.73	
			Wilcoxon signed-rank test	0.31	0.85	
8-OHdG (ng/mg・Cr)	測定値	0 週	n	14	16	
			Mean±SD	8.4±3.7	7.5±3.0	0.43
			Median [Q1, Q3]	7.6 [5.4, 11.2]	6.7 [5.8, 8.5]	0.56
			Min, Max	3.4, 15.7	2.8, 13.5	
		12 週	n	13	16	
			Mean±SD	9.0±3.8	8.2±2.5	0.50
			Median [Q1, Q3]	8.0 [7.1, 10.3]	8.6 [5.8, 10.6]	0.88
			Min, Max	3.6, 16.8	4.3, 11.5	
		24 週	n	12	15	
			Mean±SD	8.7±4.3	10.8±1.5	0.10
			Median [Q1, Q3]	8.5 [5.9, 9.6]	11.1 [10.0, 12.1]	0.016
			Min, Max	4.1, 20.1	7.3, 12.7	
	変化量	12 週	n	13	16	
			Mean±SD	0.4±2.7	0.8±3.2	0.78
			Median [Q1, Q3]	1.7 [-1.0, 2.2]	0.8 [-1.3, 3.7]	0.71
			Min, Max	-5.8, 3.6	-5.5, 5.4	
			One-sample t-test	0.56	0.35	
			Wilcoxon signed-rank test	0.37	0.36	
		24 週	n	12	15	
			Mean±SD	0.2±4.1	3.2±3.3	0.047
			Median [Q1, Q3]	1.0 [-1.5, 2.5]	3.4 [1.2, 6.0]	0.05
			Min, Max	-9.9, 6.2	-2.3, 8.3	
			One-sample t-test	0.85	0.002	
			Wilcoxon signed-rank test	0.56	0.003	
	変化率	12 週	n	13	16	
			Mean±SD	14.4±38.1	23.1±49.7	0.60
			Median [Q1, Q3]	20.0 [-12.0, 31.5]	12.5 [-18.6, 72.1]	0.78
			Min, Max	-61.7, 81.8	-43.0, 94.7	
			One-sample t-test	0.20	0.08	

			Wilcoxon signed-rank test	0.15	0.16	
		24 週	n	12	15	
			Mean±SD	17.5±60.8	68.4±84.5	0.09
			Median [Q1, Q3]	13.4 [-14.6, 28.8]	55.9 [16.2, 89.6]	0.023
			Min, Max	-70.7, 182.4	-18.0, 296.4	
			One-sample t-test	0.34	0.007	
			Wilcoxon signed-rank test	0.34	0.001	
XOR 活性 (pmol/h/mL plasma)	測定値	0 週	n	14	16	
			Mean±SD	50.3±43.6	47.4±51.6	0.87
			Median [Q1, Q3]	36.4 [22.7, 51.3]	30.9 [16.7, 53.3]	0.37
			Min, Max	13.6, 170.0	6.7, 201.0	
		24 週	n	12	15	
			Mean±SD	38.5±68.4	31.4±61.0	0.78
			Median [Q1, Q3]	14.9 [9.2, 30.3]	9.2 [6.7, 27.8]	0.26
			Min, Max	6.7, 250.0	6.7, 246.0	
	変化量	24 週	n	12	15	
			Mean±SD	-15.1±71.8	-18.8±60.8	0.89
			Median [Q1, Q3]	-18.5 [-33.9, -5.1]	-19.2 [-28.8, -4.5]	0.88
			Min, Max	-110.2, 182.2	-174.1, 138.0	
			One-sample t-test	0.48	0.25	
			Wilcoxon signed-rank test	0.034	0.010	
	変化率	24 週	n	12	15	
			Mean±SD	-28.8±96.9	-43.2±53.7	0.63
			Median [Q1, Q3]	-61.4 [-71.2, -29.9]	-63.1 [-73.5, -32.3]	0.90
			Min, Max	-84.0, 268.7	-86.6, 127.8	
			One-sample t-test	0.32	0.008	
			Wilcoxon signed-rank test	0.034	0.013	
対数変換 XOR 活性(ln (pmol/h/mL plasma))	測定値	0 週	n	14	16	
			Mean±SD	3.7±0.7	3.4±1.0	0.42
			Median [Q1, Q3]	3.6 [3.1, 3.9]	3.4 [2.8, 3.9]	0.37
			Min, Max	2.6, 5.1	1.9, 5.3	
		24 週	n	12	15	
			Mean±SD	2.9±1.1	2.7±1.1	0.53
			Median [Q1, Q3]	2.7 [2.2, 3.4]	2.2 [1.9, 3.3]	0.26
			Min, Max	1.9, 5.5	1.9, 5.5	
	変化量	24 週	n	12	15	
			Mean±SD	-0.8±0.9	-0.8±0.7	0.83
			Median [Q1, Q3]	-1.0 [-1.3, -0.4]	-1.0 [-1.3, -0.4]	0.90
			Min, Max	-1.8, 1.3	-2.0, 0.8	
			One-sample t-test	0.009	<0.001	
			Wilcoxon signed-rank test	0.016	0.001	
	変化率	24 週	n	12	15	
			Mean±SD	-21.3±21.2	-24.4±18.3	0.69
			Median [Q1, Q3]	-25.6 [-34.7, -11.7]	-31.5 [-35.7, -9.3]	0.61
			Min, Max	-44.1, 30.9	-46.5, 17.6	
			One-sample t-test	0.005	<0.001	
			Wilcoxon signed-rank test	0.012	<0.001	

血中尿酸値 (mg/dL)	測定値	0 週	n	14	16	
			Mean±SD	8.8±1.6	8.6±1.5	0.75
			Median [Q1, Q3]	8.7 [7.2, 10.2]	8.5 [7.6, 9.2]	0.85
			Min, Max	6.9, 11.9	6.7, 11.6	
		12 週	n	13	16	
			Mean±SD	6.0±1.3	6.0±0.9	0.95
			Median [Q1, Q3]	6.1 [5.3, 6.8]	6.0 [5.5, 6.6]	1.00
			Min, Max	3.9, 8.7	4.5, 7.4	
		24 週	n	12	15	
			Mean±SD	6.2±1.3	6.0±0.9	0.62
			Median [Q1, Q3]	6.6 [4.8, 7.4]	6.0 [5.2, 6.5]	0.73
			Min, Max	4.5, 7.8	4.7, 7.9	
	変化量	12 週	n	13	16	
			Mean±SD	-2.6±1.2	-2.6±1.3	0.95
			Median [Q1, Q3]	-2.2 [-3.3, -1.7]	-2.3 [-3.5, -1.8]	0.86
			Min, Max	-5.3, -1.2	-5.0, -1.0	
			One-sample t-test	<0.001	<0.001	
			Wilcoxon signed-rank test	<0.001	<0.001	
		24 週	n	12	15	
			Mean±SD	-2.2±1.3	-2.4±1.3	0.58
			Median [Q1, Q3]	-2.6 [-2.8, -1.3]	-2.2 [-3.1, -1.7]	0.86
			Min, Max	-4.6, 0.0	-5.7, -0.4	
			One-sample t-test	<0.001	<0.001	
			Wilcoxon signed-rank test	<0.001	<0.001	

表 1.3.2. [FAS] ベースライン時 EF45%未満の部分集団での FMD の変化量と EndoPAT の変化量の相関

変数 1	変数 2	n	Pearson		Spearman	
			相関係数 (95%CI)	P 値	相関係数 (95%CI)	P 値
FMD(%) 24 週変化量	EndoPAT(RHI 指数) 24 週変化量	22	0.26 (-0.19, 0.61)	0.25	0.36 (-0.08, 0.67)	0.10

1.4. ベースライン時 EF50%以上の部分集団での FMD、8-OHdG、XOR 活性、尿酸

表 1.4.1. [FAS] ベースライン時 EF50%以上の部分集団での FMD、8-OHdG、XOR 活性、尿酸

変数		観察ポイント	統計量	トピロキソスタット群	アロプリノール群	群間比較 P 値
FMD(%)	測定値	0 週	n	17	14	
			Mean±SD	4.54±2.00	4.53±2.41	0.99
			Median [Q1, Q3]	4.60 [3.30, 5.50]	4.75 [2.40, 5.50]	1.00
			Min, Max	1.70, 9.20	1.30, 10.80	
		24 週	n	17	12	
			Mean±SD	4.39±1.96	4.10±1.61	0.67
			Median [Q1, Q3]	3.70 [3.00, 5.60]	4.15 [2.85, 5.40]	0.96
			Min, Max	1.70, 9.80	1.60, 6.20	
	変化量	24 週	n	17	12	
			Mean±SD	-0.14±1.54	-0.89±1.77	0.24
			Median [Q1, Q3]	0.30 [-0.90, 1.00]	-0.60 [-1.35, 0.05]	0.22
			Min, Max	-4.30, 2.10	-5.60, 1.30	
			One-sample t-test	0.71	0.11	
			Wilcoxon signed-rank test	0.97	0.11	
8-OHdG (ng/mg・Cr)	測定値	0 週	n	22	23	
			Mean±SD	7.6±2.5	7.2±3.8	0.73
			Median [Q1, Q3]	7.3 [5.6, 9.4]	6.1 [4.8, 9.2]	0.33
			Min, Max	4.3, 13.0	2.6, 19.9	
		12 週	n	22	21	
			Mean±SD	9.1±3.8	11.9±8.1	0.15
			Median [Q1, Q3]	8.4 [6.5, 11.1]	9.2 [7.0, 14.3]	0.32
			Min, Max	4.1, 19.4	4.9, 37.9	
		24 週	n	22	21	
			Mean±SD	9.2±4.7	11.4±5.7	0.17
			Median [Q1, Q3]	8.4 [5.6, 10.5]	10.0 [7.3, 14.7]	0.11
			Min, Max	4.6, 24.9	3.6, 25.6	
	変化量	12 週	n	22	21	
			Mean±SD	1.5±3.4	4.3±6.0	0.06
			Median [Q1, Q3]	1.0 [-0.4, 3.2]	2.6 [0.5, 5.6]	0.044
			Min, Max	-5.7, 9.6	-1.1, 27.3	
			One-sample t-test	0.048	0.003	
			Wilcoxon signed-rank test	0.039	<0.001	
		24 週	n	22	21	
			Mean±SD	1.6±3.7	3.9±3.3	0.037
			Median [Q1, Q3]	1.0 [-0.4, 3.9]	3.8 [1.8, 5.9]	0.022
			Min, Max	-3.9, 11.9	-1.7, 10.6	
			One-sample t-test	0.0495	<0.001	
			Wilcoxon signed-rank test	0.08	<0.001	
	変化率	12 週	n	22	21	
			Mean±SD	26.0±52.7	59.2±66.4	0.08
			Median [Q1, Q3]	17.0 [-5.9, 38.1]	47.4 [9.1, 79.3]	0.042
			Min, Max	-46.7, 158.1	-13.9, 257.5	
			One-sample t-test	0.031	<0.001	

			Wilcoxon signed-rank test	0.036	<0.001	
		24 週	n	22	21	
			Mean±SD	25.0±50.8	60.4±56.4	0.036
			Median [Q1, Q3]	11.6 [-5.5, 39.8]	41.9 [29.0, 84.4]	0.016
			Min, Max	-32.0, 151.2	-25.0, 194.7	
			One-sample t-test	0.032	<0.001	
			Wilcoxon signed-rank test	0.06	<0.001	
XOR 活性 (pmol/h/mL plasma)	測定値	0 週	n	22	23	
			Mean±SD	51.2±36.0	53.5±76.4	0.90
			Median [Q1, Q3]	40.5 [29.3, 54.8]	23.8 [17.3, 49.9]	0.047
			Min, Max	16.3, 152.0	7.5, 329.0	
		24 週	n	22	21	
			Mean±SD	21.8±19.1	23.1±31.4	0.87
			Median [Q1, Q3]	13.8 [7.2, 34.0]	12.7 [6.7, 20.8]	0.72
			Min, Max	6.7, 65.7	6.7, 135.0	
	変化量	24 週	n	22	21	
			Mean±SD	-29.4±31.5	-33.4±54.6	0.77
			Median [Q1, Q3]	-19.8 [-41.5, -13.2]	-12.6 [-30.5, -8.2]	0.36
			Min, Max	-124.6, 14.6	-241.9, 7.6	
			One-sample t-test	<0.001	0.011	
			Wilcoxon signed-rank test	<0.001	<0.001	
	変化率	24 週	n	22	21	
			Mean±SD	-53.3±32.6	-45.9±34.3	0.47
			Median [Q1, Q3]	-58.2 [-80.0, -48.1]	-51.5 [-64.9, -39.3]	0.19
			Min, Max	-92.8, 28.6	-90.9, 81.4	
			One-sample t-test	<0.001	<0.001	
			Wilcoxon signed-rank test	<0.001	<0.001	
対数変換 XOR 活性(ln (pmol/h/mL plasma))	測定値	0 週	n	22	23	
			Mean±SD	3.8±0.6	3.4±1.0	0.17
			Median [Q1, Q3]	3.7 [3.4, 4.0]	3.2 [2.9, 3.9]	0.047
			Min, Max	2.8, 5.0	2.0, 5.8	
		24 週	n	22	21	
			Mean±SD	2.8±0.8	2.7±0.9	0.76
			Median [Q1, Q3]	2.6 [2.0, 3.5]	2.5 [1.9, 3.0]	0.72
			Min, Max	1.9, 4.2	1.9, 4.9	
	変化量	24 週	n	22	21	
			Mean±SD	-1.0±0.7	-0.8±0.6	0.25
			Median [Q1, Q3]	-0.9 [-1.6, -0.7]	-0.7 [-1.0, -0.5]	0.19
			Min, Max	-2.6, 0.3	-2.4, 0.6	
			One-sample t-test	<0.001	<0.001	
			Wilcoxon signed-rank test	<0.001	<0.001	
	変化率	24 週	n	22	21	
			Mean±SD	-26.3±18.0	-20.9±15.4	0.30
			Median [Q1, Q3]	-24.8 [-41.9, -15.7]	-22.2 [-24.2, -16.9]	0.34
			Min, Max	-58.1, 6.4	-55.8, 26.6	
			One-sample t-test	<0.001	<0.001	

			Wilcoxon signed-rank test	<0.001	<0.001	
血中尿酸値 (mg/dL)	測定値	0 週	n	21	23	
			Mean±SD	8.2±1.5	8.2±1.7	0.91
			Median [Q1, Q3]	8.1 [7.2, 9.2]	7.9 [7.0, 9.1]	0.79
			Min, Max	5.7, 11.6	6.0, 12.4	
		12 週	n	22	23	
			Mean±SD	5.4±0.9	5.7±1.3	0.42
			Median [Q1, Q3]	5.5 [5.0, 5.8]	5.7 [4.9, 6.0]	0.52
			Min, Max	3.8, 7.6	3.6, 9.0	
		24 週	n	22	22	
			Mean±SD	5.4±1.1	5.9±1.5	0.19
			Median [Q1, Q3]	5.5 [4.8, 6.0]	5.9 [5.0, 6.4]	0.26
			Min, Max	3.6, 7.4	3.1, 9.7	
	変化量	12 週	n	21	23	
			Mean±SD	-2.8±1.3	-2.5±1.6	0.44
			Median [Q1, Q3]	-2.4 [-3.8, -2.0]	-2.3 [-3.2, -1.6]	0.43
			Min, Max	-5.5, -0.9	-6.2, 0.7	
			One-sample t-test	<0.001	<0.001	
			Wilcoxon signed-rank test	<0.001	<0.001	
		24 週	n	21	22	
			Mean±SD	-2.8±1.7	-2.3±1.4	0.25
			Median [Q1, Q3]	-2.3 [-3.4, -1.8]	-1.8 [-3.2, -1.2]	0.13
			Min, Max	-6.6, 0.6	-5.7, -0.1	
			One-sample t-test	<0.001	<0.001	
			Wilcoxon signed-rank test	<0.001	<0.001	

表 1.4.2. [FAS] ベースライン時 EF50%以上の部分集団での FMD の変化量と EndoPAT の変化量の相関

変数 1	変数 2	n	Pearson		Spearman	
			相関係数 (95%CI)	P 値	相関係数 (95%CI)	P 値
FMD(%) 24 週変化量	EndoPAT(RHI 指数) 24 週変化量	29	0.27 (-0.11, 0.58)	0.15	0.25 (-0.14, 0.56)	0.20

2. PPS から心房細動除症例を除外した集団

除外した心房細動症例の定義は、心不全の原疾患もしくは合併症に心房細動があり、かつ登録時の心電図検査でも心房細動の所見があった症例、とした。

2.1. トロポニン I

表 2.1.1. [PPS] トロポニン I

変数		観察ポイント	統計量	トピロキソスタット群	アロプリノール群	群間比較 P 値
hs トロポニン I (pg/mL)	測定値	0 週	n	37	43	
			Mean±SD	25.7±39.7	18.1±24.2	0.30
			Median [Q1, Q3]	8.4 [4.9, 24.9]	7.9 [5.9, 19.5]	0.98
			Min, Max	1.5, 152.7	1.6, 132.3	
		12 週	n	37	41	
			Mean±SD	35.0±60.0	19.9±33.5	0.17
			Median [Q1, Q3]	9.6 [4.6, 29.2]	8.9 [5.1, 21.0]	0.79
			Min, Max	2.0, 294.8	1.1, 197.9	
		24 週	n	36	40	
			Mean±SD	27.1±39.0	23.4±58.9	0.74
			Median [Q1, Q3]	9.2 [5.4, 25.1]	7.7 [4.5, 21.2]	0.47
			Min, Max	1.8, 145.0	1.5, 375.3	
	変化量	12 週	n	37	41	
			Mean±SD	9.4±47.7	4.8±30.4	0.61
			Median [Q1, Q3]	0.3 [-1.0, 3.3]	0.1 [-1.5, 1.1]	0.39
			Min, Max	-17.9, 287.7	-18.9, 190.4	
			One-sample t-test	0.24	0.32	
			Wilcoxon signed-rank test	0.20	0.82	
		24 週	n	36	40	
			Mean±SD	1.7±16.0	7.5±54.4	0.54
			Median [Q1, Q3]	1.2 [-0.7, 3.5]	-0.5 [-2.8, 0.9]	0.018
			Min, Max	-68.9, 42.3	-18.4, 340.7	
			One-sample t-test	0.52	0.39	
			Wilcoxon signed-rank test	0.045	0.19	
	変化率	12 週	n	37	41	
			Mean±SD	119.6±665.2	66.1±397.3	0.66
			Median [Q1, Q3]	6.4 [-8.8, 26.7]	1.9 [-18.5, 20.5]	0.29
			Min, Max	-55.9, 4052.1	-44.9, 2538.7	
			One-sample t-test	0.28	0.29	
			Wilcoxon signed-rank test	0.08	0.62	
		24 週	n	36	40	
			Mean±SD	19.8±36.5	22.3±160.1	0.93
			Median [Q1, Q3]	20.1 [-7.1, 42.7]	-8.7 [-24.2, 19.3]	0.008
			Min, Max	-49.4, 126.5	-53.6, 984.7	
			One-sample t-test	0.003	0.38	
			Wilcoxon signed-rank test	0.002	0.47	
対数変換 hs トロポニン I(ln (pg/mL))	測定値	0 週	n	37	43	
			Mean±SD	2.42±1.23	2.37±0.99	0.83
			Median [Q1, Q3]	2.13 [1.59, 3.21]	2.07 [1.77, 2.97]	0.98

			Min, Max	0.41, 5.03	0.47, 4.89	
		12 週	n	37	41	
			Mean±SD	2.58±1.33	2.36±1.06	0.42
			Median [Q1, Q3]	2.26 [1.53, 3.37]	2.19 [1.63, 3.04]	0.79
			Min, Max	0.69, 5.69	0.10, 5.29	
		24 週	n	36	40	
			Mean±SD	2.52±1.20	2.30±1.12	0.41
			Median [Q1, Q3]	2.22 [1.67, 3.22]	2.03 [1.49, 3.05]	0.47
			Min, Max	0.59, 4.98	0.41, 5.93	
	変化量	12 週	n	37	41	
			Mean±SD	0.16±0.67	0.08±0.59	0.58
			Median [Q1, Q3]	0.06 [-0.09, 0.24]	0.02 [-0.20, 0.19]	0.29
			Min, Max	-0.82, 3.73	-0.60, 3.27	
			One-sample t-test	0.17	0.41	
			Wilcoxon signed-rank test	0.14	0.88	
		24 週	n	36	40	
			Mean±SD	0.13±0.33	-0.02±0.53	0.13
			Median [Q1, Q3]	0.18 [-0.07, 0.36]	-0.09 [-0.28, 0.18]	0.008
			Min, Max	-0.68, 0.82	-0.77, 2.38	
			One-sample t-test	0.020	0.77	
			Wilcoxon signed-rank test	0.008	0.24	
	変化率	12 週	n	37	41	
			Mean±SD	12.32±40.17	3.11±31.80	0.26
			Median [Q1, Q3]	2.00 [-3.98, 8.68]	1.09 [-7.81, 7.22]	0.34
			Min, Max	-42.69, 190.10	-82.04, 162.43	
			One-sample t-test	0.07	0.53	
			Wilcoxon signed-rank test	0.15	0.76	
		24 週	n	36	40	
			Mean±SD	10.48±21.33	-1.29±21.67	0.020
			Median [Q1, Q3]	7.63 [-1.53, 15.22]	-4.26 [-13.35, 11.23]	0.011
			Min, Max	-29.32, 66.80	-44.54, 67.27	
			One-sample t-test	0.006	0.71	
			Wilcoxon signed-rank test	0.006	0.33	

2.2. FMD

表 2.2.1. [PPS] FMD

変数		観察ポイント	統計量	トピロキソスタット群	アロプリノール群	群間比較 P 値
FMD（%）	測定値	0 週	n	29	31	
			Mean±SD	4.84±2.38	4.86±2.27	0.97
			Median [Q1, Q3]	4.60 [3.10, 5.80]	4.80 [3.40, 5.80]	0.78
			Min, Max	1.70, 11.50	1.10, 10.80	
		24 週	n	29	29	
			Mean±SD	4.99±2.51	4.54±1.72	0.42
			Median [Q1, Q3]	4.00 [3.50, 6.10]	4.20 [3.40, 5.30]	0.77
			Min, Max	1.50, 12.60	1.60, 9.20	
	変化量	24 週	n	28	28	
			Mean±SD	0.13±1.18	−0.35±1.49	0.19
			Median [Q1, Q3]	0.10 [−0.70, 1.00]	−0.35 [−0.95, 0.40]	0.22
			Min, Max	−1.90, 3.00	−5.60, 2.40	
			One-sample t-test	0.57	0.22	
			Wilcoxon signed-rank test	0.63	0.20	

表 2.2.2. [PPS] FMD の変化量と EndoPAT の変化量の相関

変数 1	変数 2	n	Pearson		Spearman	
			相関係数 (95%CI)	P 値	相関係数 (95%CI)	P 値
FMD（%） 24 週変化量	EndoPAT（RHI 指数） 24 週変化量	56	0.18 (−0.09, 0.42)	0.18	0.21 (−0.06, 0.45)	0.12

2.3. ベースライン時 EF45%未満の部分集団での FMD、8-OHdG、XOR 活性、尿酸

表 2.3.1. [PPS] ベースライン時 EF45%未満の部分集団での FMD、8-OHdG、XOR 活性、尿酸

変数		観察ポイント	統計量	トピロキソスタット群	アロプリノール群	群間比較 P 値
FMD(%)	測定値	0 週	n	11	12	
			Mean±SD	5.55±3.07	5.50±2.39	0.96
			Median [Q1, Q3]	5.50 [2.90, 7.60]	5.20 [4.10, 6.75]	0.78
			Min, Max	1.90, 11.50	1.10, 9.80	
		24 週	n	11	12	
			Mean±SD	6.06±3.08	5.02±1.98	0.34
			Median [Q1, Q3]	5.90 [4.00, 8.70]	4.15 [3.60, 5.85]	0.37
			Min, Max	1.50, 12.60	3.40, 9.20	
	変化量	24 週	n	10	11	
			Mean±SD	0.47±1.22	-0.01±1.37	0.41
			Median [Q1, Q3]	0.65 [-0.40, 1.10]	-0.20 [-1.00, 0.80]	0.29
			Min, Max	-1.30, 3.00	-1.60, 2.40	
			One-sample t-test	0.25	0.98	
			Wilcoxon signed-rank test	0.31	0.79	
8-OHdG (ng/mg・Cr)	測定値	0 週	n	13	15	
			Mean±SD	8.6±3.9	7.5±3.1	0.43
			Median [Q1, Q3]	8.3 [5.4, 11.2]	6.7 [5.7, 9.2]	0.58
			Min, Max	3.4, 15.7	2.8, 13.5	
		12 週	n	13	14	
			Mean±SD	9.0±3.8	8.7±2.4	0.77
			Median [Q1, Q3]	8.0 [7.1, 10.3]	8.8 [7.3, 10.9]	0.75
			Min, Max	3.6, 16.8	4.3, 11.5	
		24 週	n	12	14	
			Mean±SD	8.7±4.3	10.8±1.6	0.11
			Median [Q1, Q3]	8.5 [5.9, 9.6]	11.4 [10.0, 12.1]	0.019
			Min, Max	4.1, 20.1	7.3, 12.7	
	変化量	12 週	n	13	14	
			Mean±SD	0.4±2.7	1.0±3.3	0.61
			Median [Q1, Q3]	1.7 [-1.0, 2.2]	2.0 [-1.2, 3.8]	0.45
			Min, Max	-5.8, 3.6	-5.5, 5.4	
			One-sample t-test	0.56	0.26	
			Wilcoxon signed-rank test	0.37	0.27	
		24 週	n	12	14	
			Mean±SD	0.2±4.1	3.2±3.4	0.06
			Median [Q1, Q3]	1.0 [-1.5, 2.5]	3.4 [1.2, 6.0]	0.07
			Min, Max	-9.9, 6.2	-2.3, 8.3	
			One-sample t-test	0.85	0.004	
			Wilcoxon signed-rank test	0.56	0.005	
	変化率	12 週	n	13	14	
			Mean±SD	14.4±38.1	29.1±50.4	0.40
			Median [Q1, Q3]	20.0 [-12.0, 31.5]	31.1 [-16.0, 73.1]	0.45
			Min, Max	-61.7, 81.8	-43.0, 94.7	
			One-sample t-test	0.20	0.0496	

			Wilcoxon signed-rank test	0.15	0.09	
		24 週	n	12	14	
			Mean±SD	17.5±60.8	69.0±87.7	0.10
			Median [Q1, Q3]	13.4 [-14.6, 28.8]	53.7 [16.2, 89.6]	0.033
			Min, Max	-70.7, 182.4	-18.0, 296.4	
			One-sample t-test	0.34	0.011	
			Wilcoxon signed-rank test	0.34	0.002	
XOR 活性 (pmol/h/mL plasma)	測定値	0 週	n	13	15	
			Mean±SD	51.1±45.3	43.4±50.7	0.68
			Median [Q1, Q3]	33.5 [22.7, 51.3]	28.3 [15.3, 41.1]	0.29
			Min, Max	13.6, 170.0	6.7, 201.0	
		24 週	n	12	14	
			Mean±SD	38.5±68.4	16.1±14.6	0.24
			Median [Q1, Q3]	14.9 [9.2, 30.3]	8.0 [6.7, 26.9]	0.15
			Min, Max	6.7, 250.0	6.7, 47.8	
	変化量	24 週	n	12	14	
			Mean±SD	-15.1±71.8	-30.0±44.3	0.52
			Median [Q1, Q3]	-18.5 [-33.9, -5.1]	-20.1 [-28.8, -8.6]	0.96
			Min, Max	-110.2, 182.2	-174.1, 0.0	
			One-sample t-test	0.48	0.025	
			Wilcoxon signed-rank test	0.034	<0.001	
	変化率	24 週	n	12	14	
			Mean±SD	-28.8±96.9	-55.4±26.3	0.33
			Median [Q1, Q3]	-61.4 [-71.2, -29.9]	-63.1 [-73.5, -40.4]	0.70
			Min, Max	-84.0, 268.7	-86.6, 0.0	
			One-sample t-test	0.32	<0.001	
			Wilcoxon signed-rank test	0.034	<0.001	
対数変換 XOR 活性(ln (pmol/h/mL plasma))	測定値	0 週	n	13	15	
			Mean±SD	3.7±0.7	3.3±0.9	0.30
			Median [Q1, Q3]	3.5 [3.1, 3.9]	3.3 [2.7, 3.7]	0.29
			Min, Max	2.6, 5.1	1.9, 5.3	
		24 週	n	12	14	
			Mean±SD	2.9±1.1	2.5±0.8	0.20
			Median [Q1, Q3]	2.7 [2.2, 3.4]	2.1 [1.9, 3.3]	0.15
			Min, Max	1.9, 5.5	1.9, 3.9	
	変化量	24 週	n	12	14	
			Mean±SD	-0.8±0.9	-1.0±0.6	0.52
			Median [Q1, Q3]	-1.0 [-1.3, -0.4]	-1.0 [-1.3, -0.5]	0.70
			Min, Max	-1.8, 1.3	-2.0, 0.0	
			One-sample t-test	0.009	<0.001	
			Wilcoxon signed-rank test	0.016	<0.001	
	変化率	24 週	n	12	14	
			Mean±SD	-21.3±21.2	-27.4±14.7	0.40
			Median [Q1, Q3]	-25.6 [-34.7, -11.7]	-32.6 [-35.7, -17.4]	0.43
			Min, Max	-44.1, 30.9	-46.5, 0.0	
			One-sample t-test	0.005	<0.001	
			Wilcoxon signed-rank test	0.012	<0.001	

血中尿酸値 (mg/dL)	測定値	0 週	n	13	15	
			Mean±SD	8.6±1.4	8.5±1.4	0.86
			Median [Q1, Q3]	8.4 [7.2, 10.1]	8.5 [7.5, 8.8]	0.89
			Min, Max	6.9, 10.6	6.7, 11.6	
		12 週	n	13	14	
			Mean±SD	6.0±1.3	6.0±1.0	0.98
			Median [Q1, Q3]	6.1 [5.3, 6.8]	5.9 [5.3, 6.8]	0.98
			Min, Max	3.9, 8.7	4.5, 7.4	
		24 週	n	12	14	
			Mean±SD	6.2±1.3	6.1±0.9	0.72
			Median [Q1, Q3]	6.6 [4.8, 7.4]	6.2 [5.3, 6.5]	0.80
			Min, Max	4.5, 7.8	4.7, 7.9	
	変化量	12 週	n	13	14	
			Mean±SD	-2.6±1.2	-2.3±1.0	0.53
			Median [Q1, Q3]	-2.2 [-3.3, -1.7]	-2.2 [-2.7, -1.8]	0.77
			Min, Max	-5.3, -1.2	-4.4, -1.0	
			One-sample t-test	<0.001	<0.001	
			Wilcoxon signed-rank test	<0.001	<0.001	
		24 週	n	12	14	
			Mean±SD	-2.2±1.3	-2.2±0.9	0.93
			Median [Q1, Q3]	-2.6 [-2.8, -1.3]	-2.2 [-3.0, -1.7]	0.94
			Min, Max	-4.6, 0.0	-3.7, -0.4	
			One-sample t-test	<0.001	<0.001	
			Wilcoxon signed-rank test	<0.001	<0.001	

表 2.3.2. [PPS] ベースライン時 EF45%未満の部分集団での FMD の変化量と EndoPAT の変化量の相関

変数 1	変数 2	n	Pearson		Spearman	
			相関係数 (95%CI)	P 値	相関係数 (95%CI)	P 値
FMD(%) 24 週変化量	EndoPAT(RHI 指数) 24 週変化量	21	0.20 (-0.26, 0.58)	0.39	0.29 (-0.16, 0.64)	0.20

2.4. ベースライン時 EF50%以上の部分集団での FMD、8-OHdG、XOR 活性、尿酸

表 2.4.1. [PPS] ベースライン時 EF50%以上の部分集団での FMD、8-OHdG、XOR 活性、尿酸

変数		観察ポイント	統計量	トピロキソスタット群	アロプリノール群	群間比較 P 値
FMD(%)	測定値	0 週	n	15	13	
			Mean±SD	4.23±1.89	4.78±2.32	0.50
			Median [Q1, Q3]	4.00 [2.30, 5.20]	4.80 [3.40, 5.50]	0.45
			Min, Max	1.70, 9.20	1.60, 10.80	
		24 週	n	15	12	
			Mean±SD	4.33±2.04	4.10±1.61	0.75
			Median [Q1, Q3]	3.70 [2.80, 5.60]	4.15 [2.85, 5.40]	0.96
			Min, Max	1.70, 9.80	1.60, 6.20	
	変化量	24 週	n	15	12	
			Mean±SD	0.10±1.19	-0.89±1.77	0.09
			Median [Q1, Q3]	0.30 [-0.90, 1.00]	-0.60 [-1.35, 0.05]	0.16
			Min, Max	-1.90, 2.10	-5.60, 1.30	
			One-sample t-test	0.75	0.11	
			Wilcoxon signed-rank test	0.73	0.11	
8-OHdG (ng/mg・Cr)	測定値	0 週	n	20	22	
			Mean±SD	7.9±2.5	7.5±3.8	0.68
			Median [Q1, Q3]	7.6 [6.3, 9.6]	6.5 [5.0, 9.2]	0.26
			Min, Max	4.3, 13.0	3.3, 19.9	
		12 週	n	20	21	
			Mean±SD	9.1±3.9	11.9±8.1	0.18
			Median [Q1, Q3]	8.4 [6.5, 11.1]	9.2 [7.0, 14.3]	0.35
			Min, Max	4.1, 19.4	4.9, 37.9	
		24 週	n	20	21	
			Mean±SD	9.2±4.9	11.4±5.7	0.19
			Median [Q1, Q3]	8.4 [5.5, 10.7]	10.0 [7.3, 14.7]	0.11
			Min, Max	4.6, 24.9	3.6, 25.6	
	変化量	12 週	n	20	21	
			Mean±SD	1.3±3.3	4.3±6.0	0.05
			Median [Q1, Q3]	1.0 [-0.5, 2.8]	2.6 [0.5, 5.6]	0.024
			Min, Max	-5.7, 9.6	-1.1, 27.3	
			One-sample t-test	0.10	0.003	
			Wilcoxon signed-rank test	0.10	<0.001	
		24 週	n	20	21	
			Mean±SD	1.4±3.7	3.9±3.3	0.024
			Median [Q1, Q3]	0.7 [-1.0, 3.0]	3.8 [1.8, 5.9]	0.011
			Min, Max	-3.9, 11.9	-1.7, 10.6	
			One-sample t-test	0.12	<0.001	
			Wilcoxon signed-rank test	0.21	<0.001	
	変化率	12 週	n	20	21	
			Mean±SD	19.8±46.1	59.2±66.4	0.034
			Median [Q1, Q3]	14.2 [-6.9, 36.7]	47.4 [9.1, 79.3]	0.020
			Min, Max	-46.7, 158.1	-13.9, 257.5	
			One-sample t-test	0.07	<0.001	

			Wilcoxon signed-rank test	0.11	<0.001	
		24 週	n	20	21	
			Mean±SD	17.7±46.0	60.4±56.4	0.012
			Median [Q1, Q3]	11.0 [-14.7, 34.6]	41.9 [29.0, 84.4]	0.004
			Min, Max	-32.0, 151.2	-25.0, 194.7	
			One-sample t-test	0.10	<0.001	
			Wilcoxon signed-rank test	0.18	<0.001	
XOR 活性 (pmol/h/mL plasma)	測定値	0 週	n	20	22	
			Mean±SD	53.6±36.9	54.9±77.8	0.94
			Median [Q1, Q3]	40.9 [30.4, 58.2]	24.4 [17.3, 49.9]	0.045
			Min, Max	16.3, 152.0	7.5, 329.0	
		24 週	n	20	21	
			Mean±SD	23.1±19.6	23.1±31.4	1.00
			Median [Q1, Q3]	16.2 [7.8, 34.4]	12.7 [6.7, 20.8]	0.52
			Min, Max	6.7, 65.7	6.7, 135.0	
	変化量	24 週	n	20	21	
			Mean±SD	-30.5±32.8	-33.4±54.6	0.84
			Median [Q1, Q3]	-19.8 [-42.9, -14.6]	-12.6 [-30.5, -8.2]	0.33
			Min, Max	-124.6, 14.6	-241.9, 7.6	
			One-sample t-test	<0.001	0.011	
			Wilcoxon signed-rank test	<0.001	<0.001	
	変化率	24 週	n	20	21	
			Mean±SD	-52.2±33.7	-45.9±34.3	0.55
			Median [Q1, Q3]	-58.2 [-80.0, -43.0]	-51.5 [-64.9, -39.3]	0.24
			Min, Max	-92.8, 28.6	-90.9, 81.4	
			One-sample t-test	<0.001	<0.001	
			Wilcoxon signed-rank test	<0.001	<0.001	
対数変換 XOR 活性(ln (pmol/h/mL plasma))	測定値	0 週	n	20	22	
			Mean±SD	3.8±0.6	3.4±1.0	0.16
			Median [Q1, Q3]	3.7 [3.4, 4.1]	3.2 [2.9, 3.9]	0.045
			Min, Max	2.8, 5.0	2.0, 5.8	
		24 週	n	20	21	
			Mean±SD	2.8±0.8	2.7±0.9	0.59
			Median [Q1, Q3]	2.8 [2.1, 3.5]	2.5 [1.9, 3.0]	0.52
			Min, Max	1.9, 4.2	1.9, 4.9	
	変化量	24 週	n	20	21	
			Mean±SD	-1.0±0.7	-0.8±0.6	0.30
			Median [Q1, Q3]	-0.9 [-1.6, -0.6]	-0.7 [-1.0, -0.5]	0.24
			Min, Max	-2.6, 0.3	-2.4, 0.6	
			One-sample t-test	<0.001	<0.001	
			Wilcoxon signed-rank test	<0.001	<0.001	
	変化率	24 週	n	20	21	
			Mean±SD	-25.5±18.3	-20.9±15.4	0.39
			Median [Q1, Q3]	-24.8 [-40.5, -13.3]	-22.2 [-24.2, -16.9]	0.43
			Min, Max	-58.1, 6.4	-55.8, 26.6	
			One-sample t-test	<0.001	<0.001	

			Wilcoxon signed-rank test	<0.001	<0.001	
血中尿酸値 (mg/dL)	測定値	0 週	n	19	22	
			Mean±SD	8.2±1.5	8.2±1.7	0.98
			Median [Q1, Q3]	8.1 [7.2, 9.2]	8.0 [7.0, 9.1]	0.85
			Min, Max	5.7, 11.6	6.0, 12.4	
		12 週	n	20	22	
			Mean±SD	5.3±0.8	5.8±1.3	0.20
			Median [Q1, Q3]	5.5 [4.9, 5.8]	5.8 [5.0, 6.0]	0.31
			Min, Max	3.8, 6.7	3.6, 9.0	
		24 週	n	20	21	
			Mean±SD	5.3±1.0	6.0±1.4	0.05
			Median [Q1, Q3]	5.4 [4.7, 6.0]	5.9 [5.2, 6.4]	0.09
			Min, Max	3.6, 7.3	3.6, 9.7	
	変化量	12 週	n	19	22	
			Mean±SD	-2.9±1.3	-2.4±1.6	0.32
			Median [Q1, Q3]	-2.6 [-3.8, -2.0]	-2.2 [-3.2, -1.6]	0.27
			Min, Max	-5.5, -0.9	-6.2, 0.7	
			One-sample t-test	<0.001	<0.001	
			Wilcoxon signed-rank test	<0.001	<0.001	
		24 週	n	19	21	
			Mean±SD	-2.9±1.8	-2.2±1.4	0.17
			Median [Q1, Q3]	-2.7 [-4.2, -1.8]	-1.7 [-3.2, -1.2]	0.08
			Min, Max	-6.6, 0.6	-5.7, -0.1	
			One-sample t-test	<0.001	<0.001	
			Wilcoxon signed-rank test	<0.001	<0.001	

表 2.4.2. [PPS] ベースライン時 EF50%以上の部分集団での FMD の変化量と EndoPAT の変化量の相関

変数 1	変数 2	n	Pearson		Spearman	
			相関係数 (95%CI)	P 値	相関係数 (95%CI)	P 値
FMD (%) 24 週変化量	EndoPAT (RHI 指数) 24 週変化量	27	0.20 (-0.19, 0.54)	0.31	0.20 (-0.20, 0.53)	0.33
