# Supplementary figures and images for: Topiroxostat versus allopurinol in patients with chronic heart failure complicated by hyperuricemia: A prospective, randomized, open-label, blinded-end-point clinical trial
Source: PLoS One. 2022 Jan 25;17(1):e0261445. doi: 10.1371/journal.pone.0261445 (PMC8789120; doi:10.1371/journal.pone.0261445)

# ANCOVA

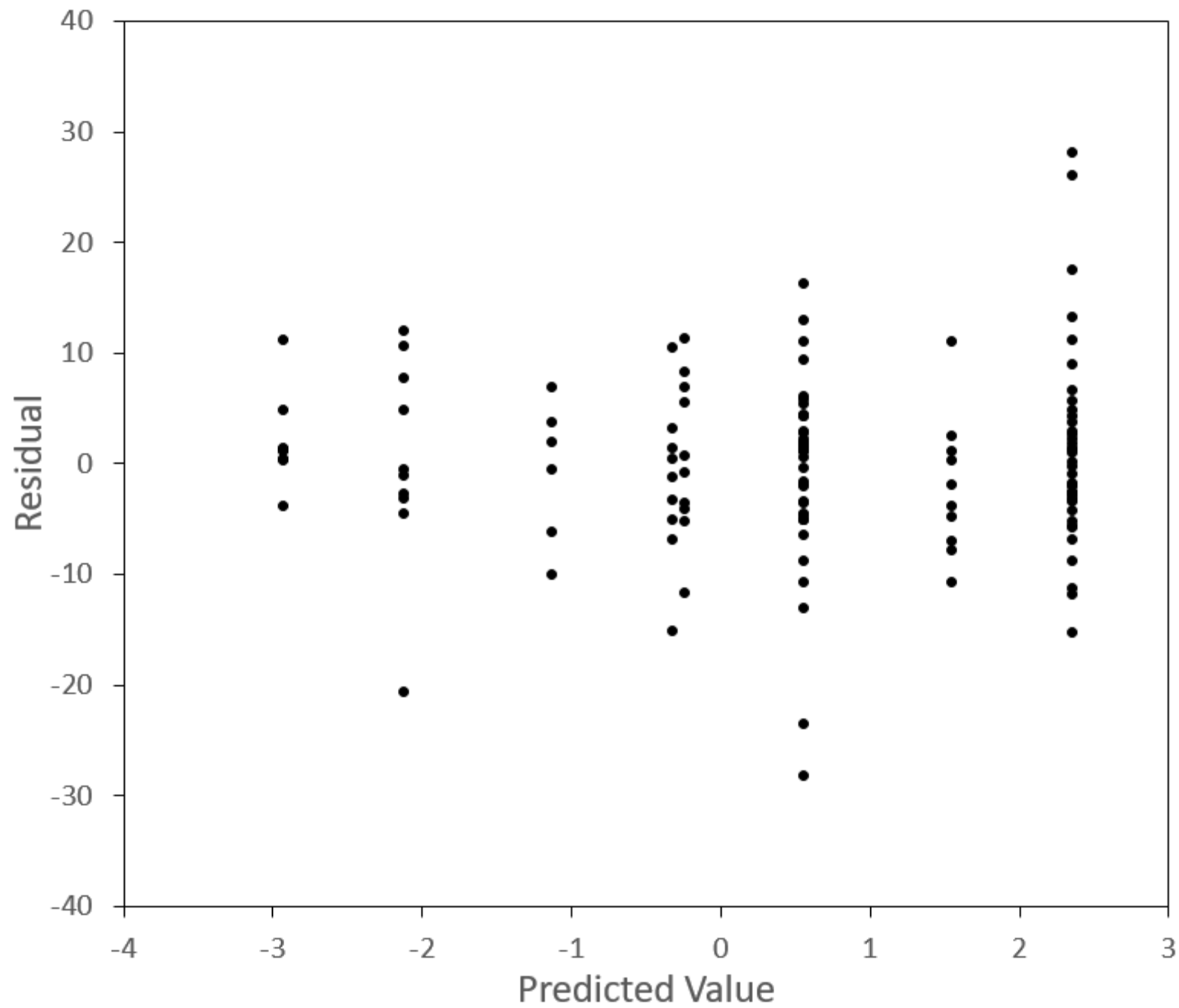

# MMRM

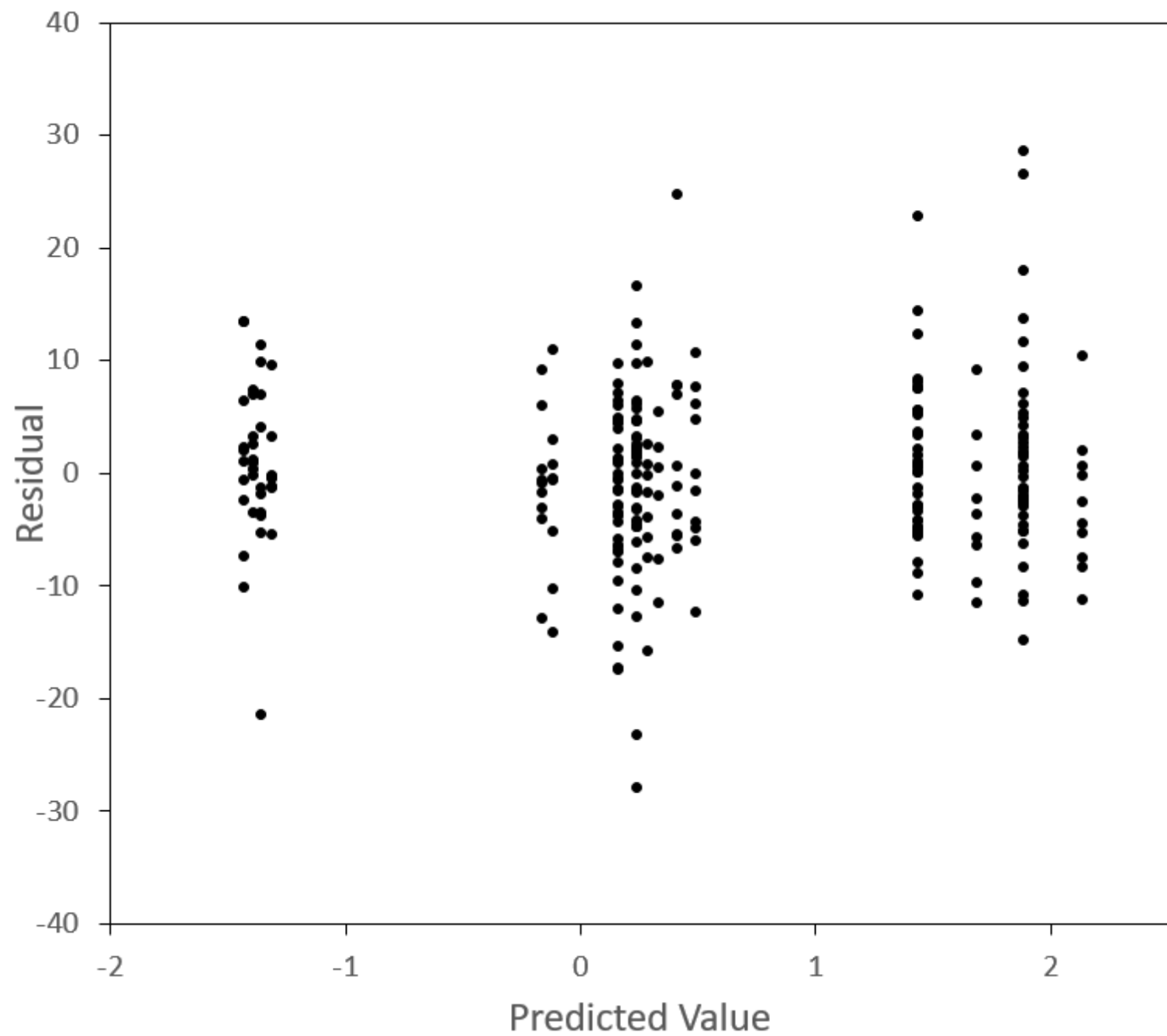

Supplement: S4 Appendix — (PDF) [file pone.0261445.s011.pdf]
